# Nucleophilic Gallyl Anions Enable Ga─Au Bimetallic Ammonia Activation

**DOI:** 10.1002/anie.7183298

**Published:** 2026-05-11

**Authors:** Hellen Videa, Keelan M. Byrne, Tobias Krämer, Antonio J. Martínez‐Martínez

**Affiliations:** ^1^ Department of Chemistry and Center for Research in Sustainable Chemistry (CIQSO), University of Huelva Campus El Carmen Huelva Spain; ^2^ Department of Chemistry Maynooth University Maynooth Co. Kildare Ireland; ^3^ School of Chemistry, Trinity College Dublin College Green Dublin 2 Ireland

**Keywords:** ammonia activation, bimetallic complexes, cooperative reactivity, gallium, gold

## Abstract

Controlling the reactivity of low‐valent main‐group nucleophiles in small‐molecule activation remains a significant challenge. Here, we report the synthesis of a family of charge‐delocalized β‐diketiminate gallyl anions, accessible across the Li–Cs series, whose aggregation and ion pairing can be tuned to furnish a cation‐gated gallium(I) nucleophilic platform. Only the cesium crown‐encapsulated derivative forms a truly separated ion pair (SIP) that exhibits clean nucleophilic reactivity toward (IPr)AuCl to generate an unsupported Ga─Au bimetallic complex, isolable, and structurally authenticated. Coordination at Au enhances the Lewis acidity at gallium(I), enabling cooperative N─H and O─H σ‐bond activation under mild conditions via a substrate‐assisted proton‐shuttle pathway. This reactivity, exemplified by NH_3_ and H_2_O, and extended to MeNH_2_ and MeOH, is inaccessible to the free gallyl anions. These findings define a new gallium‐based nucleophilic platform for exploiting bimetallic cooperation in small‐molecule bond activation.

## Graphical abstract

Unlocking gallyl nucleophiles: Counterion control enables formation of an unsupported gallyl─gold complex. Gold coordination induces a polarity switch at gallium, enabling cooperative activation of NH_3_ and related N─H/O─H substrates under mild conditions.

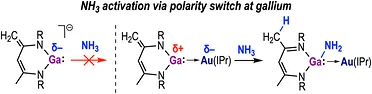

## 1 Introduction

The activation of small molecules and strong σ‐bonds remains a significant challenge in modern synthesis. While transition‐metal complexes have traditionally dominated this area, low‐valent main‐group compounds, when paired with tailored ligand and counterion environments, can deliver comparable reactivity [1, 2, 3, 4, 5, 6]. Among these, carbene‐analogue species across Groups 13–15 (borylenes, silylenes, and heavier pnictogen analogues) have emerged as versatile platforms [7]. By exploiting ambiphilic or strongly nucleophilic behavior, they mediate transformations that are formally analogous to oxidative addition or σ‐bond metathesis, thereby enabling selective cleavage of otherwise inert bonds under mild conditions.

**FIGURE 1:**
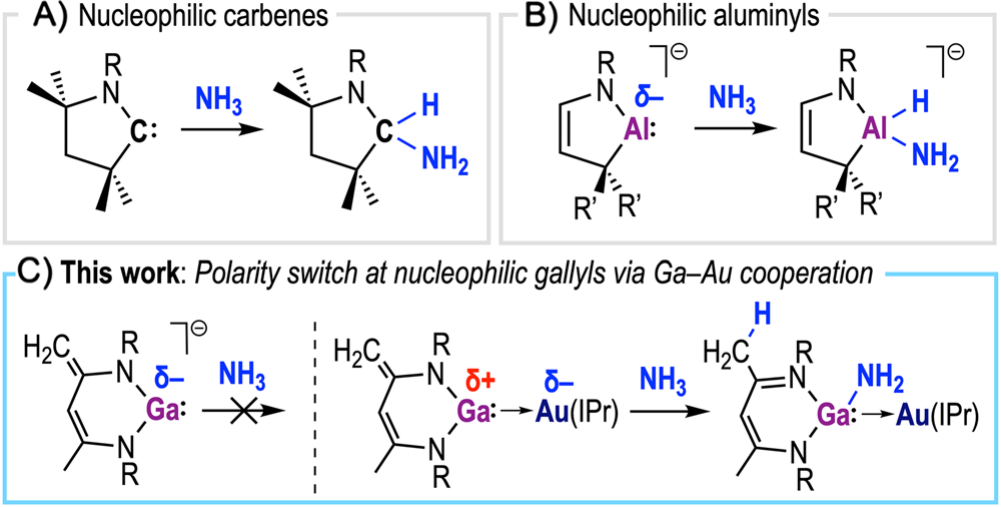
Representative main‐group strategies for NH_3_ activation: (A) Nucleophilic carbenes; (B) Nucleophilic aluminyl anions; (C) This work: polarity switch at nucleophilic gallyl anions via Ga─Au cooperation.

In this context, ammonia (NH_3_) and water (H_2_O) are stringent benchmarks for polar σ‐bond activation [8, 9]. In NH_3_, strong N─H bonds (100.3 kcal mol^−1^) and the formation of robust Werner‐type coordination frustrate productive scission, while H_2_O presents similarly strong O─H bonds (113.0 kcal mol^−1^) and demanding proton‐transfer requirements [10, 11, 12]. Main‐group strategies for E─H bond activation broadly fall into three canonical modes. In oxidative‐addition‐type processes, nucleophilic carbenes can split NH_3_ at a carbon center (Figure 1A) [13, 14], and geometrically constrained phosphorus(III) compounds cleave NH_3_ and H_2_O under mild conditions [15, 16, 17, 18]. Cooperative Lewis acid/base approaches are exemplified by Al–C ambiphiles, in which a Lewis‐acidic aluminum center and a Lewis‐basic carbon site act in concert to add across N─H bonds [19]. In heterolytic/deprotonation regimes, silylene–borane ambiphiles achieve H_2_O dehydrogenation [20], while bismuth(II) systems employ single‐electron transfer to cleave N─H/O─H bonds [21]. Within Group 13, nucleophilic aluminyl anions can split NH_3_ oxidatively (Figure 1B) [22] and even effect dual dehydrogenation of NH_3_ with H_2_ evolution [23].

The heavier congener gallium is an attractive target for main‐group‐mediated bond activation chemistry. Gallium(I) is softer and more polarizable than aluminum(I), and neutral gallylenes are established carbene analogues that engage in diverse bond‐activation chemistry [24, 25, 26, 27, 28, 29, 30]. Anionic gallyl systems likewise have clear precedent [31, 32, 33, 34, 35, 36, 37, 38], including group 11 metal–gallyl complexes [39]. Complementary Au–Ga structural precedent has also been accessed from a neutral Ga(I) manifold [40]. However, compared with aluminyl platforms [41, 42, 43, 44, 45, 46, 47, 48, 49, 50, 51, 52, 53, 54, 55, 56, 57], the use of gallyl anions as controllable nucleophiles for bond activation remains challenging, in part because access to reactive yet controllable gallyl species is constrained by Ga─Ga aggregation, ligand redistribution, and disproportionation [58, 59, 60, 61].

In this study, we report a charge‐delocalized β‐diketiminate gallyl platform (Figure 1C) with programmable nucleophilic function across the Li–Cs series. We show that aggregation and ion pairing gate access to an operationally “naked” gallium nucleophile, with the cesium/18‐crown‐6 encapsulated derivative undergoing clean Au─Cl substitution with (IPr)AuCl to generate an unsupported Ga─Au bimetallic species that is isolable and structurally authenticated. Embedding this Ga(I) nucleophile within a Ga─Au bond enhances its Lewis acidity, enabling cooperative N─H and O─H activation under mild conditions via a ligand‐assisted proton‐shuttling pathway. Beyond NH_3_ and H_2_O, this reactivity extends to MeNH_2_ and MeOH, whereas the free gallyl anions either remain unreactive toward amines or undergo protic quenching. These findings establish a cation‐gated nucleophilic gallyl platform as a new entry point to cooperative bond activation at low‐valent gallium.

## 2 Results and Discussion

### 2.1 Synthesis and Counterion‐Programmable Speciation

**SCHEME 1:**
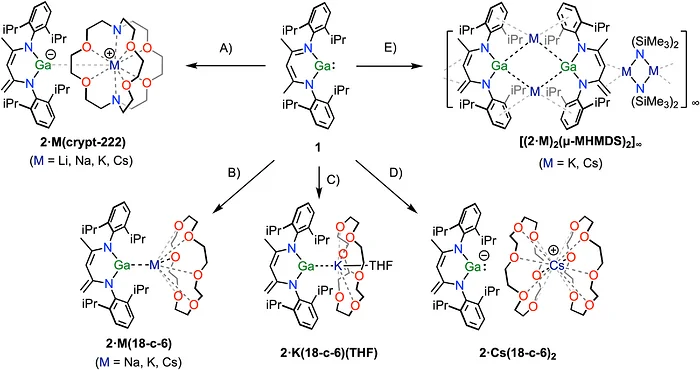
Synthesis of alkali‐metal gallyl complexes from gallylene **1**: (A) **2·M(crypt‐222)**: MHMDS, crypt‐222, benzene or toluene, rt; (B) **2·M(18‐c‐6)**: MHMDS, 18‐crown‐6, benzene or toluene, rt; (C) **2·M(18‐c‐6)(THF)**: KHMDS, 18‐crown‐6, benzene/THF, rt; (D) **2·Cs(18‐c‐6)_2_**: CsHMDS, 18‐crown‐6 (2 equiv), benzene, rt; (E) **[(2·M)_2_(μ‐MHMDS)_2_]_∞_**: MHMDS (2 equiv), benzene or toluene, 50°C.

Selective deprotonation of the β‐diketiminate (BDI) backbone in the neutral gallylene **1** [62] with MHMDS (M = Li, Na, K, and Cs) in the presence of cryptand[2.2.2] (crypt‐222) cleanly furnishes a complete Li^+^–Cs^+^ series of charge‐separated gallyl salts **2·M(crypt‐222)** in benzene or toluene at room temperature (Scheme 1A). In this transformation, the amide base removes a backbone methyl proton to generate the gallyl anion **2^–^** supported by a dianionic BDI’ scaffold. Encapsulation of M^+^ by crypt‐222 then stabilizes **2^–^** as a separated ion pair (SIP). Replacing crypt‐222 with 18‐crown‐6 modulates aggregation in a predictable fashion. With Na^+^, K^+^ and Cs^+^, one equivalent of crown ether gives contact‐ion‐pair (CIP) monomers **2·M(18‐c‐6)** (Scheme 1B), while in the presence of coordinating THF the adduct **2·K(18‐c‐6)(THF)** is isolated (Scheme 1C). Increasing ligand saturation to two equivalents of 18‐crown‐6 with Cs^+^ promotes complete ion separation, affording **2·Cs(18‐c‐6)_2_** (Scheme 1D), which mirrors the crypt‐encapsulated analogue **2·M(crypt‐222)**.

In the absence of donor ligands, deprotonation of **1** at room temperature is unsuccessful; however, gentle heating to 50°C enables the isolation of the donor‐free polymeric assemblies **[(2·M)_2_(μ‐MHMDS)_2_]_∞_** (M = K, Cs; Scheme 1E). Unlike the cryptand/crown‐stabilized monomers, these derivatives incorporate bridging (MHMDS)_2_ units and form polymeric arrays in the solid state, a structural motif not encountered in related alkali‐metal aluminyl dimers (Al·M)_2_ [45, 51, 52]. When substoichiometric amounts of MHMDS were employed, only the polymeric **[(2·M)_2_(μ‐MHMDS)_2_]_∞_** species were obtained, albeit in reduced yield, with no evidence for discrete donor‐free gallyl dimers **(2·K)_2_** or **(2·Cs)_2_**, underscoring the strong propensity of the donor‐free **(2·M)_2_** units to bind additional amide and propagate polymerization via (MHMDS)_2_ bridges.

This amide‐driven backbone deprotonation contrasts sharply with the behavior of **1** toward organo‐alkali bases. Whereas KCH(SiMe_3_)_2_ induces Ga─Ga bond formation to the cyclopentagallene dianion [Ga_5_R_5_]^2−^ rather than a gallyl [63], the analogous aluminylene system forms aluminyl dimers (Al·K)_2_ [45, 51], highlighting a chemically orthogonal outcome. We also note that the gallyls **2·M(donor)** are also thermally robust in benzene and toluene, in contrast to many low‐valent gallium systems that cluster or disproportionate [64], and to related aluminyls, which activate aromatic solvents at room temperature [45, 52].

To delineate how the alkali‐metal cation and donor ligands govern Ga···M contacts and aggregation, we combined single‐crystal X‐ray diffraction, multinuclear NMR, and diffusion‐ordered (DOSY) NMR spectroscopy. Formation of the gallyl anion **2^−^** is evident in the ^1^H NMR spectra by disappearance of the backbone methyl resonance of **1** and emergence of two diastereotopic methylene signals at δ ∼2.7 and 1.9 ppm across the Li–Cs series, consistent with installation of a conjugated CH_2_═C fragment on the BDI’ backbone. Concomitantly, the Dipp (2,6‐iPr_2_C_6_H_3_) substituents become magnetically inequivalent, giving two distinct sets of ^1^H/^13^C resonances, consistent with the lowered symmetry of the dianionic BDI’ framework in **2^−^**.

**FIGURE 2:**
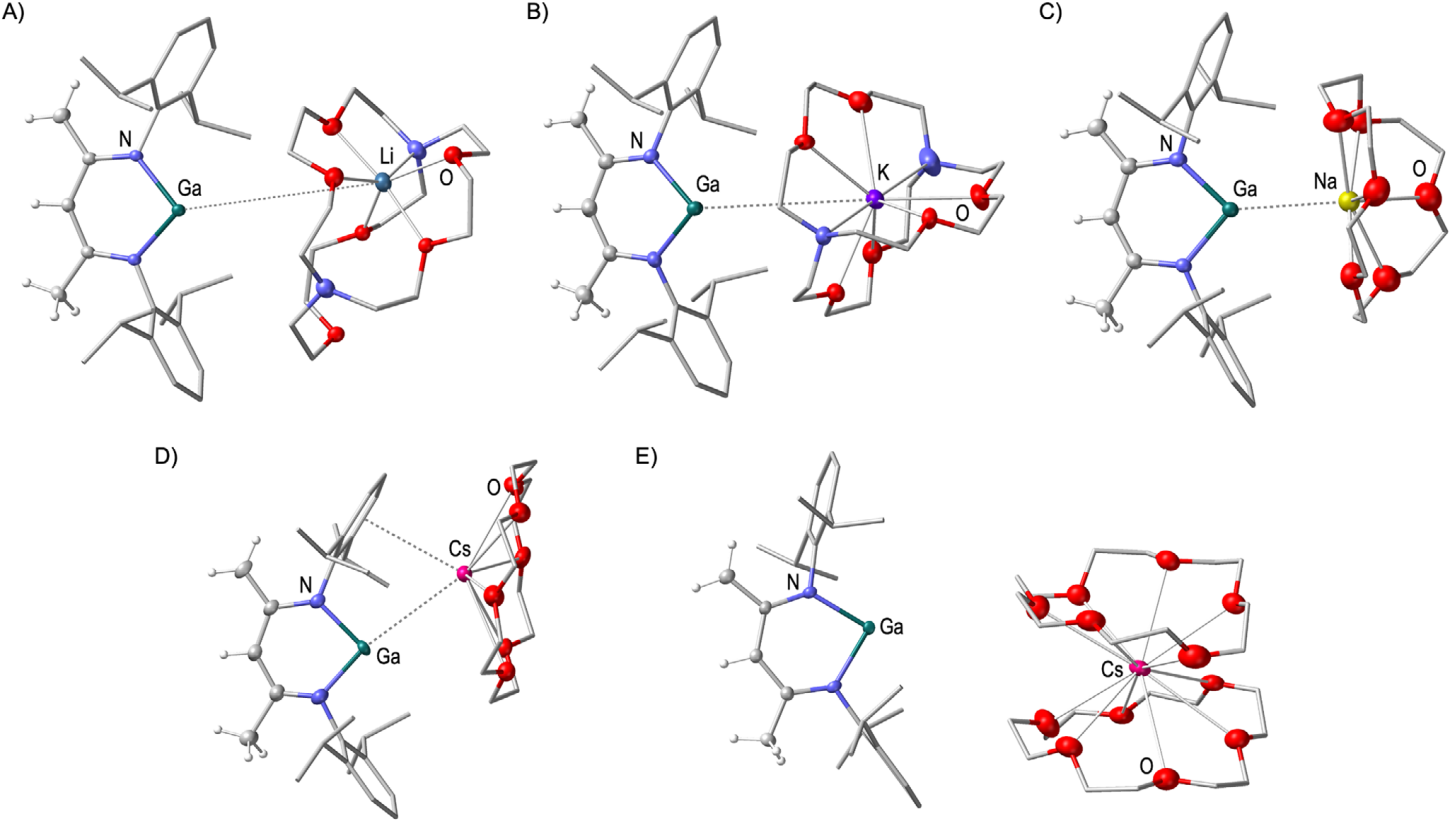
Crystal structures of monomeric gallyl complexes: (A) **2·Li(crypt‐222)**: Ga···Li 5.769(2) Å; (B) **2·K(crypt‐222)**: Ga···K 5.1539(15) Å; (C) **2·Na(18‐c‐6)**: Ga···Na 3.3372(16) Å; (D) **2·Cs(18‐c‐6)**: Ga···Cs 3.7076(17), Cs···π(Dipp) 3.556(7) Å; and (E) **2·Cs(18‐c‐6)_2_**: Ga···Cs >8 Å. Most hydrogen atoms omitted, and Dipp (2,6‐iPr_2_C_6_H_3_) substituents shown in wireframe format for clarity; thermal ellipsoids at 35%.

Single‐crystal X‐ray structures of **2·M(crypt‐222)** (M = Li, K, Cs; Figures 2A,B and S57) show SIPs with no short Ga···M contacts (Ga···M >5.1 Å). In solution, DOSY NMR shows co‐diffusion of **2^−^** and the crypt‐cations within each salt, with hydrodynamic diameters of 16–18 Å (*D* = 5.19–6.82 × 10^−10^ m^2^ s^−1^), indicating that anion and cation move together on the NMR timescale, despite the absence of direct Ga···M interactions in the solid state. We attribute this behavior to ion‐pair association in the low‐dielectric medium, even when M^+^ is encapsulated by crypt‐222.

Replacing crypt‐222 with 18‐crown‐6 contracts the Ga···M contacts and affords CIP monomers **2·M(18‐c‐6)** (M = Na, K, Cs; Figures 2C,D and S60). The resulting Ga···M distances (Na 3.3372(16), K 3.4872(13), Cs 3.7076(17) Å) lie close to the corresponding sums of covalent radii (d/Σ_r,cov_ = 1.02–1.07) [65], consistent with substantive cation–anion interaction when M^+^ is only partially encapsulated by the crown ether. In **2·Cs(18‐c‐6)**, additional Cs^+^···π(Dipp) engagement is evident (3.556(7) Å), reflecting the low charge density of Cs^+^ and the high polarizability at Ga(I). In solution, DOSY NMR data show codiffusion of **2^−^** and the crown‐bound cation for **2·M(18‐c‐6)** (*D* = 4.49–5.95 × 10^−10^ m^2^ s^−1^), corresponding to hydrodynamic diameters of ∼15 Å, consistent with associated ion pairing. By contrast, the fully encapsulated salt **2·Cs(18‐c‐6)_2_** displays distinct diffusion coefficients for the cation (*D* = 7.18 × 10^−10^ m^2^ s^−1^) and anion (*D* = 4.90 × 10^−10^ m^2^ s^−1^), evidencing true separation in solution. This behavior mirrors the >8 Å Ga···Cs separation observed crystallographically (Figure 2E) and confirms that **2·Cs(18‐c‐6)_2_** behaves as a genuine SIP.

**FIGURE 3:**
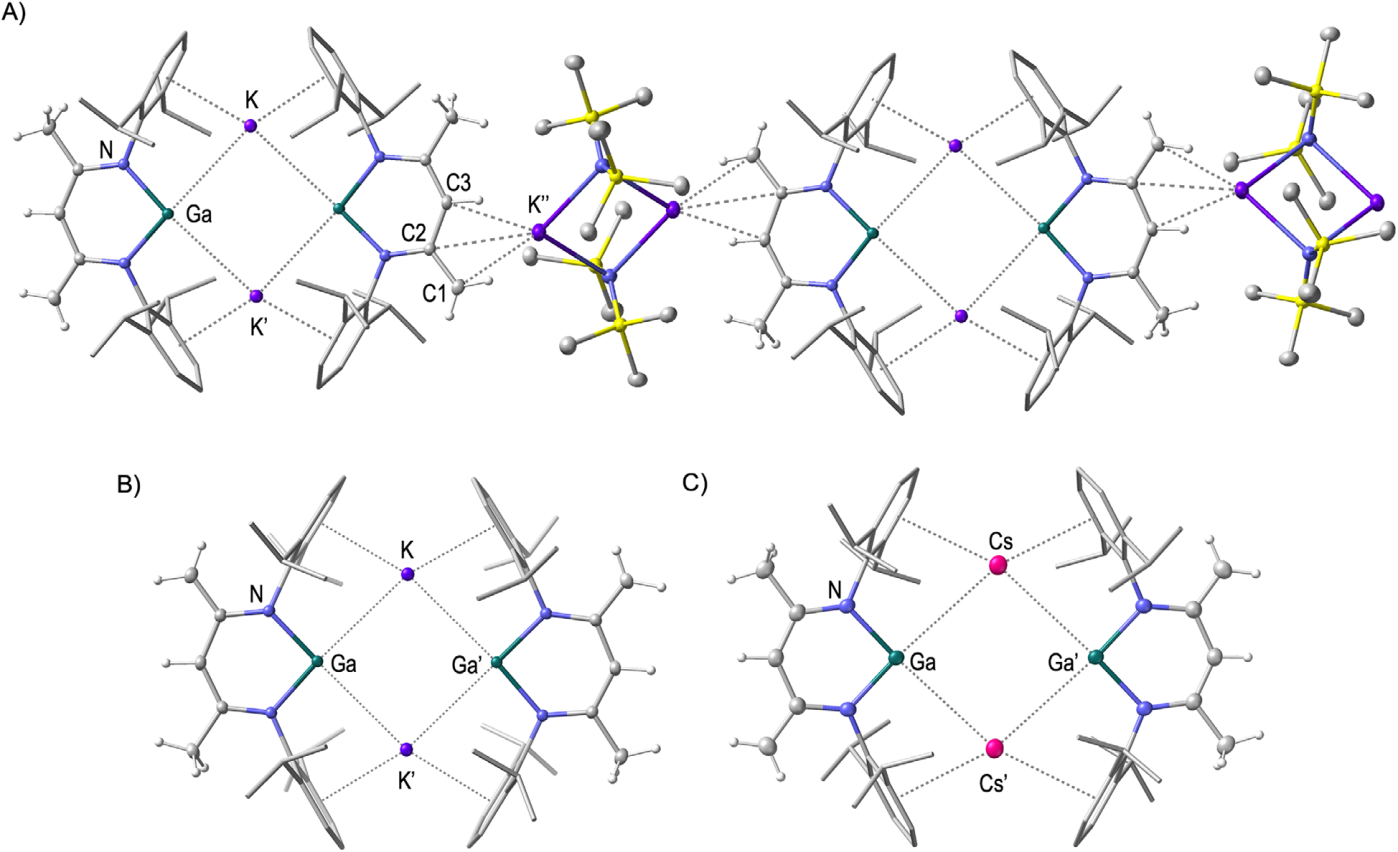
Crystal structures of the polymeric **[(2·M)_2_(μ‐MHMDS)_2_]_∞_** gallyl assemblies: (A) Section of the polymeric chain in **[(2·K)_2_(μ‐KHMDS)_2_]_∞_** showing propagation through bridging (μ‐MHMDS)_2_ units and K'···π(backbone) interactions (C1 3.4889(15), C2 3.340(2), C3 3.2405(16) Å); (B) Dimeric **(2·K)_2_** repeat unit extracted from **[(2·K)_2_(μ‐KHMDS)_2_]_∞_** with Ga···K (K‘ 3.4679(5), K’ 3.4778(5) Å) and K···π(Dipp) interactions (K' 2.8207(7), K' 2.8940(7) Å); symmetry operation (') = ‐x,y,1/2‐z; (C) Dimeric **(2·Cs)_2_** repeat unit extracted from **[(2·Cs)_2_(μ‐CsHMDS)_2_]_∞_** with Ga···Cs (Cs 3.8481(6), Cs' 3.7860(6) Å) and Cs···π(Dipp) interactions (Cs 3.2539(11), Cs' 3.2829(13) Å); symmetry operation (') = 1‐x,2‐y,1‐z. Most hydrogen atoms omitted, and Dipp (2,6‐iPr_2_C_6_H_3_) substituents shown in wireframe format for clarity; thermal ellipsoids at 35%.

In the absence of external donors, only the polymeric species **[(2·M)_2_(μ‐MHMDS)_2_]_∞_** (M = K, Cs; Figures 3A and S73) were obtained crystallographically. These 1D assemblies are built from dimeric Ga_2_M_2_ repeat units (Figure 3B,C), linked by bridging (μ‑MHMDS)_2_ units through M^+^···π(backbone) contacts. The Ga···M distances remain close to, though slightly longer than, the corresponding sums of covalent radii (d/Σ_r,cov_ = 1.07 for K and 1.05 for Cs) [65]. Together with pronounced M^+^···π(Dipp) interactions (K 2.8940(7)/2.9207(7), Cs 3.2539(11)/3.2839(13) Å), these metrics support substantial cation–anion association within the dimeric cores.

Despite the polymeric solid‐state structures of **[(2·M)_2_(μ‐MHMDS)_2_]_∞_**, DOSY NMR in the presence of THF indicates disassembly into (MHMDS)_2_ units and discrete **(2·M)_2_** dimers, the latter with hydrodynamic diameters of ∼17 Å fully consistent with free Ga_2_M_2_ cores (see Supporting Information). Thus, the donor‐free polymeric chains collapse to strongly associated dimers rather than fully dissociated monomeric ion pairs, even under coordinating conditions. This behavior contrasts with **2·Cs(18‐c‐6)_2_**, which behaves as a SIP in solution, and with related aluminyl dimers (Al·M)_2_, which are more labile and can equilibrate between dimeric and monomeric species [52]. This distinction will prove important when considering the nucleophilicity of these donor‐free gallyl systems in subsequent reactivity studies (*vide infra*).

### 2.2 Bonding and Charge‐Delocalization

**FIGURE 4:**
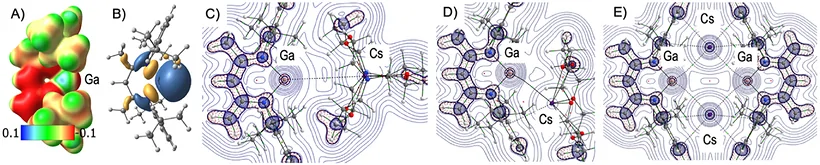
(A) Molecular electrostatic potential map for **2^−^** (isovalue 0.025 a.u.); (B) Kohn‐Sham orbital representation of the Ga‐centered lone‐pair (HOMO–1) orbital in **2^−^**. QTAIM graphs and negative Laplacian (–∇^2^ *ρ*(*r*)) contour plots in planes containing the Ga and N atoms for (C) **2·Cs(crypt‐222)**, (D) **2·Cs(18‐c‐6)**, and (E) **(2·Cs)_2_**. Bond‐critical points (green dots), ring‐critical points (red dots), and bond paths (solid/dotted black lines) are shown.

To gain further insight into the electronic structure of the gallyl anion **2^−^** in its different aggregation states, we carried out DFT calculations (PBE0‐D3(BJ)/def2‐QZVP//PBE/def2‐TZVP/def2‐SV(P)) [66, 67, 68]. The gas‐phase optimized geometries reproduce the salient features of the solid‐state structures. In all cases, **2^−^** is highly polarized, with substantial electron density delocalized over the deprotonated BDI' backbone (Figures 4A and S107). QTAIM analysis identifies polar Ga─N bonds with bond‐critical‐points (BCP) electron densities 𝜌(*r*) = 0.081–0.089 ea_0_^−3^ and positive Laplacians ∇^2^𝜌(*r*) = 0.228–0.265 ea_0_^−5^, consistent with strong donor–acceptor N→Ga interactions rather than purely covalent Ga─N bonding [69]. The corresponding NBO second‐order perturbation energies for N(lp)→Ga(acceptor) interactions (Δ*E* ^(2)^ = 5.7–7.5 kcal mol^−1^) likewise support the view that the BDI' ligand is a key stabilizing donor to the Ga(I) center.

The frontier orbital picture (Figure 4B) further underscores the ligand‐centered nature of the charge distribution. In **2^−^**, the HOMO is predominantly located on the BDI' framework, reflecting backbone‐driven charge delocalization, whereas the Ga‐centered lone pair resides in the HOMO–1 and the LUMO is largely p‐type at Ga(I). Laplacian maps show a compact valence‐shell charge concentration at Ga (Figure 4C–E), in line with an s‐rich Ga lone pair, and the NBO hybrid analysis estimates ∼90–95% s‐character across the series, compared with ∼80–85% typically obtained for aluminyl analogues [46, 48, 70]. Despite this more s‐character‐rich lone pair, the calculated HOMO–LUMO gaps for the gallyl series (4.1–4.5 eV) are comparable to the reported values for aluminyl anions (3.4–4.1 eV) [46, 48, 70], indicating that **2^−^** retains a similarly accessible frontier‐orbital manifold. Taken together, the ligand‐delocalized charge distribution, compact Ga(I)‐centered lone pair, and accessible Ga‐based LUMO render **2^−^** well‐suited to nucleophilic reactivity.

For the cryptand‐encapsulated salts **2·M(crypt‐222)**, QTAIM analysis reveals no significant Ga⋯M BCPs, except for a very weak contact in **2·Cs(crypt‐222)** (𝜌(*r*) = 0.001 ea_0_^−3^, ∇^2^𝜌(*r*) = 0.003 ea_0_^−5^, Figure 4C). Replacing crypt‐222 with 18‐crown‐6 strengthens Ga···M association, giving BCPs consistent with more substantive, predominantly ionic contacts (𝜌(*r*) = 0.008–0.010 ea_0_^−3^ and ∇^2^𝜌(*r*) = 0.018–0.028 ea_0_^−5^). Noncovalent interaction (NCI) surfaces corroborate this picture, showing minimal cation–anion engagement in the cryptand derivatives and a moderate increase for the crown‐stabilized species (Figure S108). NBO second‐order perturbation energies for Ga(lp)→M(σ*) donation decrease down the group for the 18‐crown‐6 salts (Δ*E* ^(2)^ = 20.8, 16.0, 14.5 kcal mol^−1^ for Na, K, and Cs), reflecting weaker charge transfer to the larger and more diffuse cations. In **2·Cs(18‐c‐6)**, an additional BCP between Cs^+^ and a Dipp π‐surface of the BDI' ligand is also found, in line with the pronounced cation···π propensity of Cs^+^ [71].

For the donor‐free dimers **(2·K)_2_** and **(2·Cs)_2_**, the Ga···M interactions likewise exhibit low‐density BCPs, consistent with predominantly ionic closed‐shell cation–anion association within the Ga_2_M_2_ cores (𝜌(*r*) = 0.008–0.009 ea_0_^−3^, ∇^2^𝜌(*r*) = 0.016–0.019 ea_0_^−5^, Figure 4E). Additional M^+^⋯π BCPs to both Dipp rings further consolidate the dimers in the absence of external donors, and NCI surfaces show diffuse weakly attractive envelopes around these cation⋯π regions. A very shallow Ga···Ga feature appears for **(2·K)_2_** at a separation of 5.09 Å, which we ascribe to long‐range dispersive proximity rather than any covalent Ga─Ga interaction (𝜌(*r*) = 0.003 ea_0_^−3^, ∇^2^𝜌(*r*) = 0.004 ea_0_^−5^); no analogous feature is found for **(2·Cs)_2_** with a Ga···Ga distance of 5.71 Å. NBO second‐order stabilization energies for Ga(lp)→M(σ*) donation again decrease down the group (Δ*E* ^(2)^ = 18.5 kcal mol^−1^ for K and 8.3 kcal mol^−1^ for Cs), consistent with greater charge‐transfer stabilization for the smaller, more charge‐dense K^+^ cation. Together with the stronger cation⋯π engagement for K^+^, these trends rationalize the enhanced association observed experimentally for donor‑free **(2·M)_2_** aggregates even in coordinating media (*vide supra*).

Energy decomposition analysis (EDA) attributes the **2**···M interactions primarily to electrostatics (∼70%), with a smaller orbital (∼28%) and minor dispersion (∼2%) contributions, mirroring trends reported for related aluminyls [72]. The corresponding interaction energies are substantial (–350 kcal mol^−1^ for K and –328 kcal mol^−1^ for Cs), reflecting formal ion pairing reinforced by multiple M^+^···π contacts within the Ga_2_M_2_ cores. EDA‐NOCV identifies a dominant Ga(lp)→M(acceptor) channel, with secondary M^+^···π(Dipp) contributions. Thus, a predominantly electrostatic scaffold, combined with non‑negligible charge transfer, accounts for the persistence of these dimeric Ga_2_M_2_ aggregates even in coordinating media such as THF.

### 2.3 Nucleophilic Gallyls and Gallyl─Gold Bimetallic Reactivity

**SCHEME 2:**
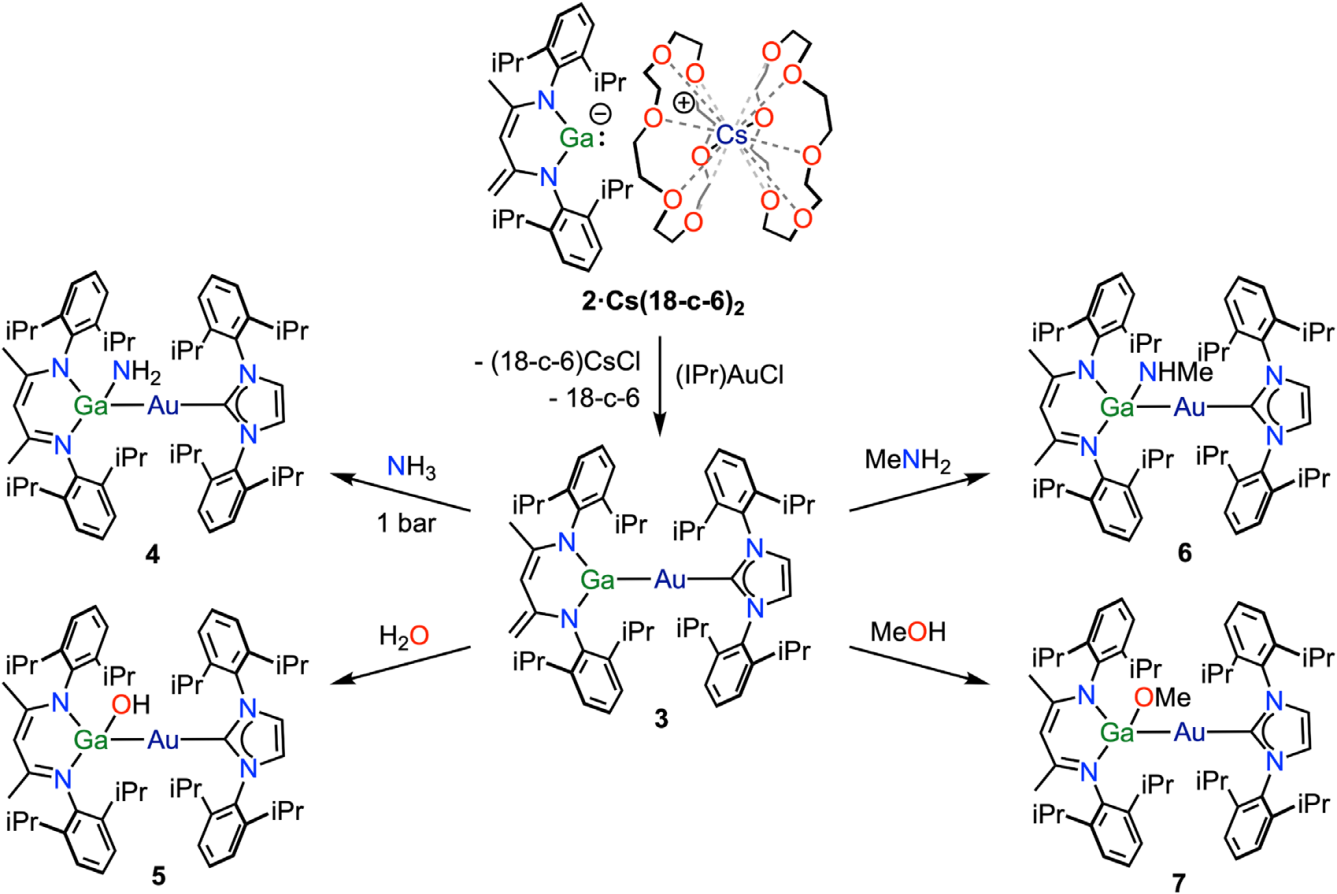
Formation of the gallyl─gold complex **3** from the separated gallyl salt **2·Cs(18‐c‐6)_2_** and (IPr)AuCl, and its subsequent cooperative N─H and O─H activation chemistry with NH_3_, H_2_O, MeNH_2_, and MeOH to give **4**–**7**, respectively.

Having established the solution speciation of the gallyls **2·M(L)** as a function of alkali‐metal cation and donor ligand, we next examined their reactivity toward a benchmark Au electrophile, (IPr)AuCl (IPr = 1,3‐bis(2,6‐diisopropylphenyl)imidazol‐2‐ylidene). Under otherwise identical conditions in toluene at room temperature (1:1 stoichiometry), the monomeric species **2·M(crypt‐222)** (M = Li–Cs), **2·M(18‐c‐6)** (M = Na, K, Cs), **2·K(18‐c‐6)(THF)**, and the donor‐free polymers **[(2·M)_2_(μ‐MHMDS)_2_]_∞_** (M = K, Cs) leave (IPr)AuCl unchanged even after prolonged reaction times. In sharp contrast, only the separated cesium gallyl **2·Cs(18‐c‐6)_2_** reacts cleanly with (IPr)AuCl to give the Ga─Au species **3** (Scheme 2). This marked counterion dependence shows that effective exposure of the Ga lone pair in solution is decisive. Full encapsulation of the large, soft Cs^+^ cation by two 18‐crown‐6 ligands attenuates both Ga···Cs and Cs^+^···π interactions, allowing **2·Cs(18‐c‐6)_2_** to behave as an operationally “naked” gallyl nucleophile in solution, paralleling aluminyl nucleophiles [73].

**FIGURE 5:**
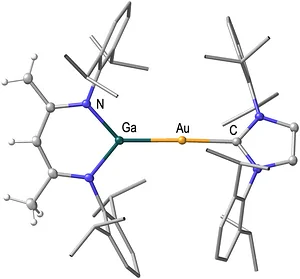
Crystal structure of the isolated gallyl─gold complex **3**, showing an unsupported Ga─Au unit (Ga─Au 2.3874(8) Å) and a near‐linear carbene–Au–Ga angle of 178.27(16)°. Cocrystallized toluene and most hydrogen atoms omitted, and Dipp (2,6‐iPr_2_C_6_H_3_) substituents shown in wireframe format for clarity; thermal ellipsoids at 35%.

Complex **3** was isolated as a yellow crystalline material. Its ^1^H NMR spectrum shows an IPr backbone CH signal at δ 6.27 ppm (cf. (IPr)AuCl, δ 6.29 ppm), a shift of the BDI' γ‐CH resonance from δ 5.31 in **2·Cs(18‐c‐6)_2_** to 5.17 ppm, and retention of the exocyclic CH_2_═C resonances at δ 3.65 and 2.83 ppm. Single‐crystal X‐ray diffraction of **3** (Figure 5) confirms an unsupported Ga─Au core, with a Ga─Au distance of 2.3874(8) Å, comparable to reported Ga─Au bond lengths [39, 40, 74, 75], and a near‐linear Ga─Au–carbene angle of 178.27(16)°. The Ga center is essentially trigonal planar, with a sum of angles of ∼360°. An independent second crystal form displays closely comparable Ga─Au core metrics. Although small amounts of THF aided crystallization of **3**, neither crystal form contains coordinated THF, and DOSY NMR does not support a persistent THF adduct in solution (see Supporting Information).

**FIGURE 6:**
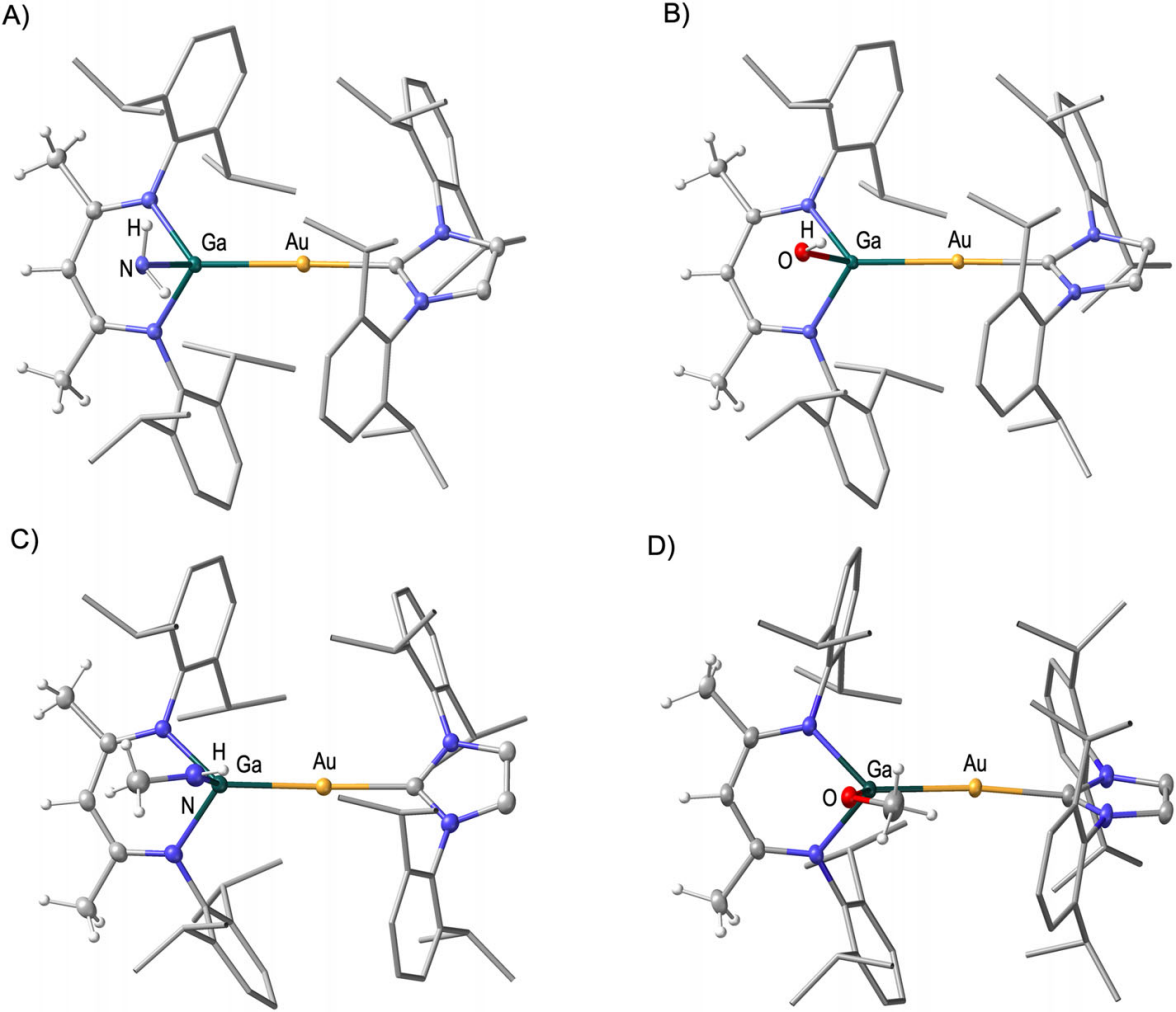
Crystal structures of N─H and O─H activation products **4**–**7**. (A) **4**: Ga–NH_2_ 1.920(2) Å, Ga─Au 2.4052(3) Å. (B) **5**: Ga─OH 1.8768(13) Å, Ga─Au 2.4045(2) Å. (C) **6**: Ga–NHMe 1.883(2) Å, Ga─Au 2.4203(3) Å. (D) **7**: Ga─OMe 1.875(2) Å, Ga─Au 2.4165(4) Å. For **4** and **6**, the N─H hydrogen atoms, and for **5**, the O─H hydrogen atom, were located in the difference map and freely refined. Most hydrogen atoms omitted, and Dipp (2,6‐iPr_2_C_6_H_3_) substituents shown in wireframe format for clarity; thermal ellipsoids at 35%.

With **3** in hand, we first examined its reactivity toward NH_3_. Exposure of a toluene solution of **3** to NH_3_ (1 bar) at room temperature instantaneously gives **4** (Scheme 2). In the ^1^H NMR spectrum, a broad singlet at δ –0.07 ppm (2H) is assigned to a new Ga–NH_2_ group, while singlets at δ 1.54 and 4.68 ppm correspond to the β‐Me and γ‐CH resonances of the protonated BDI' backbone. The IPr resonances remain sharp and shift only slightly, indicating retention of the Au(IPr) fragment. The crystal structure of **4** (Figure 6A) shows a four‐coordinate Ga center bearing the Ga–NH_2_ group (1.920(2) Å) within a slightly elongated Ga─Au unit (2.4052(3) Å). The β‐diketiminate backbone exhibits two C─Me bonds (1.512(3) and 1.518(3) Å), consistent with protonation of the BDI' skeleton. These data support the formal N─H activation of NH_3_ at the Ga center, with the BDI' ligand acting as the proton acceptor in **3**.

Likewise, exposure of **3** to a moist atmosphere affords **5** (Scheme 2), consistent with O─H activation of H_2_O at **3**. The ^1^H NMR spectrum of **5** shows a broad Ga─OH resonance at δ –0.26 ppm together with singlets at δ 1.56 ppm and 4.75 ppm, again indicative of protonation of the BDI' backbone. Single‐crystal X‐ray diffraction of **5** (Figure 6B) establishes a Ga─OH bond (1.8768(13) Å) on the Ga─Au core (2.4045(2) Å).

To probe whether this activation manifold operates beyond NH_3_ and H_2_O, we next examined the reactivity of **3** toward MeNH_2_ and MeOH under otherwise analogous conditions. In both cases, clean conversion afforded **6** and **7**, respectively (Scheme 2). In the ^1^H NMR spectrum of **6**, a broad quartet at δ –0.18 (1H) together with a doublet at δ 3.07 (3H) is consistent with a Ga–NHMe fragment, while **7** shows a singlet at δ 3.69 for the Ga─OMe group; both compounds display the same protonated BDI' pattern observed for **4** and **5**. The crystal structures of **6** and **7** (Figure 6C,D) confirm Ga–NHMe and Ga─OMe groups with distances of 1.882(2) and 1.875(2) Å on their Ga─Au units, with Ga─Au distances of 2.4203(3) and 2.4165(4) Å, respectively. The resulting amido and alkoxy products, **6** and **7**, therefore parallel **4** and **5**, consistent with a common activation manifold.

Control experiments support that clean N─H and O─H bond activations are intrinsic to the Ga─Au bimetallic platform **3** (see Supporting Information). Under the same conditions, **2·Cs(18‐c‐6)_2_** does not react cleanly with NH_3_ or MeNH_2_, and no defined products are obtained when other gallyl salts **2·M(L)** are treated with these amines. Likewise, exposure of **2·Cs(18‐c‐6)_2_**, or other representative gallyl salts **2·M(L)**, to H_2_O or MeOH causes rapid protic quenching and complex mixtures containing, at most, transient amounts attributable to **1**; no clean formation of hydroxo or alkoxy products analogous to **5** or **7** is observed. (IPr)AuCl remains inert toward these substrates under the same conditions. The amido products **4** and **6** also convert cleanly to **5** upon exposure to a moist atmosphere, consistent with hydrolytic replacement of the Ga–NRH unit by Ga─OH. Thus, generation of the Ga─Au species **3** is required for clean cooperative N─H/O─H bond activation.

Mechanistic DFT calculations provide a coherent picture linking the counterion‐controlled nucleophilicity of the gallyl anion **2^−^** to the cooperative reactivity displayed by the Ga─Au species **3**. EDA–NOCV analysis of the Ga─Au bond in **3** reveals a strongly attractive interaction (–168 kcal mol^−1^) dominated by donation from the gallyl lone pair into Au (–99 kcal mol^−1^), consistent with viewing **2^−^** as a nucleophilic metalloligand. Coordination of Au(IPr) increases the NPA charge at Ga from +0.72 e in **2^−^** to +1.29 e in **3** and stabilizes the Ga‐centered p‐type LUMO (Figures S82 and S87), reflecting migration of electron density from Ga into the Au–carbene manifold. In the free gallyl anion **2^−^**, this LUMO lies 1.8 eV higher, NH_3_ binds only weakly to Ga, and the computed Δ*G* ^‡^ barrier for N─H activation is >25 kcal mol^−1^, exceeding those found for **3**, accompanied by pronounced distortion and partial decoordination of a BDI' N‐donor arm (Figures S112 and S113). These features account for the lack of observed NH_3_ reactivity of the gallyls **2·M(L)**.

**FIGURE 7:**
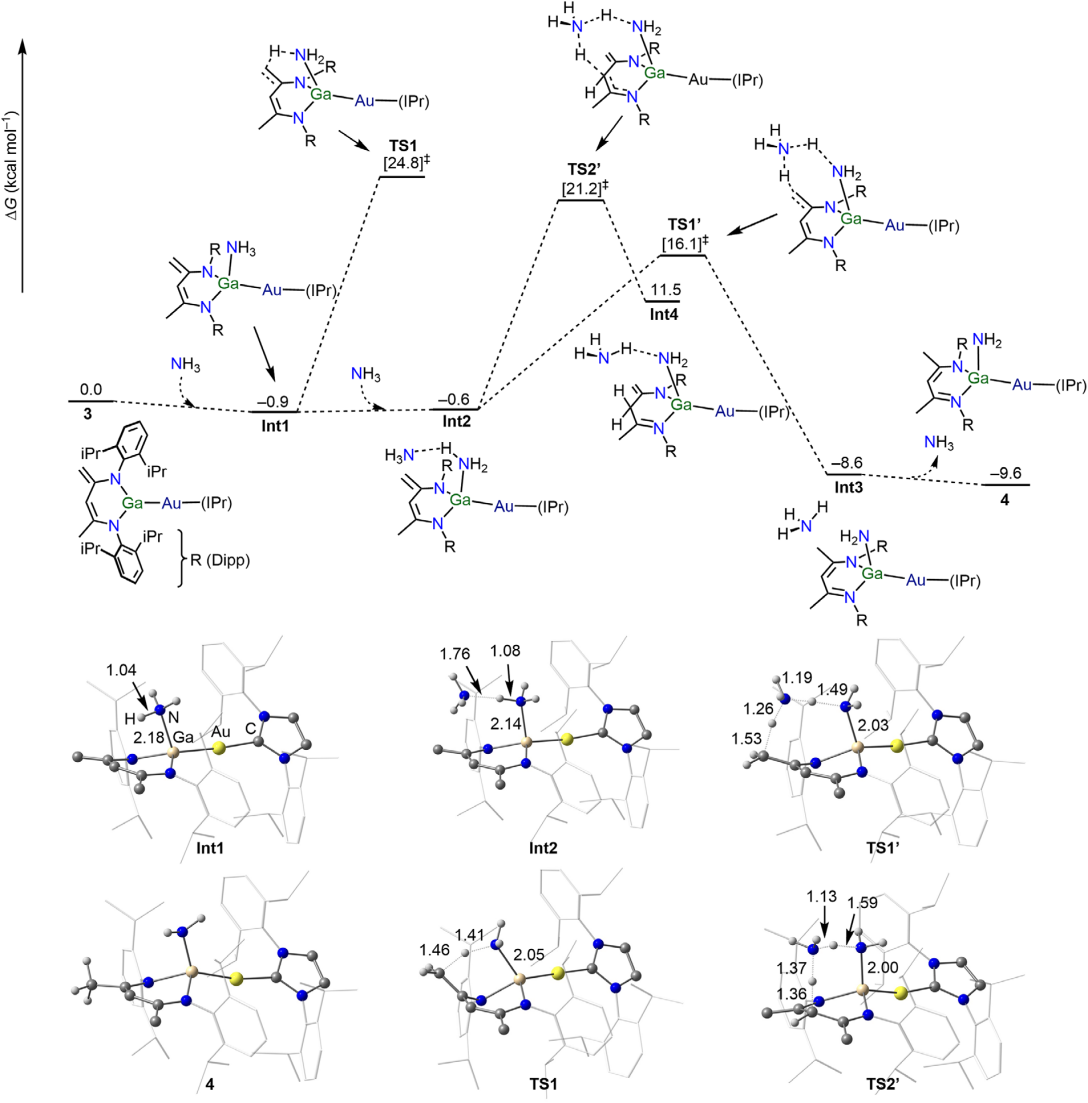
Calculated Gibbs free‐energy reaction profile (298.15 K, in kcal mol^−1^) for NH_3_ activation at **3** at the PBE0‐D3(BJ)/def2‐TZVP//PBE‐D3(BJ)/def2‐TZVP/def2‐SV(P) level of theory, corrected for benzene solvent. Bond distances are given in Å for key optimized intermediates and transition states.

For **3**, the lowest‐energy pathway for NH_3_ activation proceeds by an NH_3_‐assisted proton‐shuttle mechanism (Figure 7). Initial coordination of NH_3_ to Ga in **3** is slightly exergonic (**Int1,** Δ*G* = –0.9 kcal mol^−1^). A second NH_3_ molecule then relays proton transfer from the Ga‐bound NH_3_ to the exocyclic CH_2_═C group via **TS1'** (Δ*G* ^‡^ = 16.1 kcal mol^−1^), furnishing **4** with an overall driving force of –9.6 kcal mol^−1^. The alternative γ‐CH protonation pathway is less favorable (**TS2'**, Δ*G* ^‡^ = 21.2 kcal mol^−1^) and leads to a high‐energy intermediate (**Int4,** Δ*G* = 11.5 kcal mol^−1^) that is neither observed experimentally nor productive.

Single‐substrate pathways are also substantially higher in energy (e.g. **TS1**, Δ*G* ^‡^ ∼ 25 kcal mol^−1^) and are therefore noncompetitive. The close structural analogy among **4**–**7** is consistent with a common Ga‐centered cooperative activation manifold. Overall, the calculations therefore support a two‐substrate proton‐shuttle mechanism at the Ga─Au framework, in which a second NH_3_ molecule acts as a transient Brønsted base to lower the barrier, closely echoing proton‐shuttling mechanisms in related aluminyl systems [23]. In this manifold, Au coordination transforms **2^−^** from a nucleophilic metalloligand into a cooperative Ga─Au species **3** in which Ga acts as the substrate‐binding Lewis‐acidic site for binding the substrate and promoting N─H/O─H bond cleavage via a BDI′ backbone proton acceptor. In this sense, the **2^−^** → **3** sequence constitutes a formal donor‐to‐acceptor polarity switch at Ga within a coinage‐metal bimetallic framework.

## 3 Conclusion

We have established a charge‐delocalized β‐diketiminate gallyl platform in which aggregation and ion pairing across the Li–Cs series control access to nucleophilic gallium(I) reactivity. Only the fully separated cesium derivative behaves as an operationally “naked” gallyl nucleophile, providing isolable access to an unsupported gallyl─gold complex. Embedding the gallyl fragment within this Ga─Au bimetallic platform switches gallium from a nucleophilic donor to a more Lewis‐acidic site, enabling cooperative N─H and O─H activation of NH_3_ and H_2_O, and more generally, of MeNH_2_ and MeOH to give the corresponding amido and alkoxy products, reactivity that is not observed for the alkali‐metal gallyls alone. Combined experimental and computational data support a substrate‐assisted proton‐shuttle pathway at gallium in which the β‐diketiminate ligand acts as the proton acceptor. These findings demonstrate a donor‐to‐acceptor polarity switch at gallium within a low‐valent Ga─Au bimetallic platform and establish a new entry point to cooperative small‐molecule E─H bond activation.

## 4 Experimental Section

Synthetic, characterization and computational data for all compounds, together with spectra and crystallographic details are included in the Supporting Information [76].

## Author Contributions

**Hellen Videa**: investigation, data curation, formal analysis, visualization, writing ‐ review and editing. **Keelan M. Byrne**: software, formal analysis, visualization, investigation, data curation. **Tobias Krämer**: supervision, funding acquisition, formal analysis, writing ‐ review and editing, resources, validation, methodology. **Antonio J. Martínez‐Martínez**: conceptualization, supervision, project administration, funding acquisition, writing ‐ original draft, writing ‐ review and editing, resources, methodology, validation.

## Conflicts of Interest

The authors declare no conflicts of interest.

## Supporting information




**Supporting File 1**: anie72587‐sup‐0001‐SuppMat.Pdf.

**Supporting File 2**: anie72587‐sup‐0002‐cif.Zip.

## Data Availability

The data that support the findings of this study are available in the supporting information of this article.
