# Supplementary material for: Nucleophilic Gallyl Anions Enable Ga─Au Bimetallic Ammonia Activation
Source: Angew Chem Int Ed Engl. 2026 May 11;65(35):e7183298. doi: 10.1002/anie.7183298 (PMC13502605; doi:10.1002/anie.7183298)
Supplement: Supplementary file 1 [file ANIE-65-e7183298-s001.pdf]

Supporting Information

Nucleophilic Gallyl Anions Enable Ga–Au Bimetallic Ammonia Activation

Hellen Videa,[a] Keelan M. Byrne,[b] Tobias Krämer*[b,c] and Antonio J. Martínez-Martínez*[a]

[a] Department of Chemistry and Center for Research in Sustainable Chemistry (CIQSO), Campus El Carmen, University of Huelva, Huelva (Spain)

[b] Department of Chemistry, Maynooth University, Maynooth, Co. Kildare, Ireland

[c] School of Chemistry, Trinity College Dublin, College Green, Dublin 2, Ireland

** E-mail: kraemert@tcd.ie, antonio.martinez@dqcm.uhu.es*

Table of Contents

S1 Materials and General Methods 4
S2 Synthetic Procedures and Characterization Data 6
S2.1 Synthesis and characterization of 2·Li(crypt-222) 6
S2.2 Synthesis and characterization of 2·Na(crypt-222) 9
S2.3 Synthesis and characterization of 2·K(crypt-222) 11
S2.4 Synthesis and characterization of 2·Cs(crypt-222) 13
S2.5 Synthesis and characterization of 2·Na(18-c-6) 15
S2.6 Synthesis and characterization of 2·K(18-c-6) 17
S2.7 Synthesis and characterization of 2·K(18-c-6)(THF) 19
S2.8 Synthesis and characterization of 2·Cs(18-c-6) 20
S2.9 Synthesis and characterization of 2·Cs(18-c-6)$_2$ 23
S2.10 Synthesis and characterization of [(2·K)$_2$(KHMDS)$_2$]$_\infty$ 26
S2.11 Synthesis and characterization of [(2·Cs)$_2$(CsHMDS)$_2$]$_\infty$ 28
S3 Nucleophilic Substitution Reactivity 31
S3.1 Synthesis and characterization of 3 31
S3.2 Synthesis and characterization of 4 33
S3.3 Synthesis and characterization of 5 35
S3.4 Synthesis and characterization of 6 37
S3.5 Synthesis and characterization of 7 40
S3.6 Control experiments supporting the gallyl–gold reactivity 42
S4 ^{1}H Diffusion-Ordered Spectroscopy (DOSY) NMR Studies 43
S4.1 ^{1}H DOSY NMR and hydrodynamic radii analyses 43
S4.1.1 ^{1}H DOSY NMR spectroscopic study of 2·Li(crypt-222) 47
S4.1.2 ^{1}H DOSY NMR spectroscopic study of 2·Na(crypt-222) 49
S4.1.3 ^{1}H DOSY NMR spectroscopic study of 2·K(crypt-222) 51
S4.1.4 ^{1}H DOSY NMR spectroscopic study of 2·Cs(crypt-222) 53
S4.1.5 ^{1}H DOSY NMR spectroscopic study of 2·Na(18-c-6) 55
S4.1.6 ^{1}H DOSY NMR spectroscopic study of 2·K(18-c-6) 57
S4.1.7 ^{1}H DOSY NMR spectroscopic study of 2·Cs(18-c-6) 59
S4.1.8 ^{1}H DOSY NMR spectroscopic study of 2·Cs(18-c-6)$_2$ 61
S4.1.9 ^{1}H DOSY NMR spectroscopic study of [(2·K)$_2$(KHMDS)$_2$]$_\infty$ 63
S4.1.10 ^{1}H DOSY NMR spectroscopic study of [(2·Cs)$_2$(CsHMDS)$_2$]$_\infty$ 66
S4.1.11 ^{1}H DOSY NMR spectroscopic study of 3 with added THF 68
S5 Crystal Structure Determinations 69
S5.1 Crystal data and structure refinement for 2·Li(crypt-222) (CCDC 2391227) 70
S5.2 Crystal data and structure refinement for 2·K(crypt-222) (CCDC 2391228) 72
S5.3 Crystal data and structure refinement for 2·Cs(crypt-222) (CCDC 2391229) 74
S5.4 Crystal data and structure refinement for 2·Na(18-c-6) (CCDC 2391231) 76
S5.5 Crystal data and structure refinement for 2·K(18-c-6) (CCDC 2391232) 78
S5.6 Crystal data and structure refinement for 2·K(18-c-6)(THF) (CCDC 2391234) 84
S5.7 Crystal data and structure refinement for 2·Cs(18-c-6) (CCDC 2391235) 86
S5.8 Crystal data and structure refinement for 2·Cs(18-c-6)$_2$ (CCDC 2391230) 88
S5.9 Crystal data and structure refinement for [(2·K)$_2$(KHMDS)$_2$]$_\infty$ (CCDC 2391236) 90
S5.10 Crystal data and structure refinement for [(2·Cs)$_2$(CsHMDS)$_2$]$_\infty$ (CCDC 2391237) 93
S5.11 Crystal data and structure refinement for 3·(toluene)$_{0.5}$ (CCDC 2546880) 96
S5.12 Crystal data and structure refinement for 3·benzene (CCDC 2546879) 98
S5.13 Crystal data and structure refinement for 4 (CCDC 2502503) 100
S5.14 Crystal data and structure refinement for 5 (CCDC 2502504) 102
S5.15 Crystal data and structure refinement for 6 (CCDC 2546881) 104
S5.16 Crystal data and structure refinement for 7 (CCDC 2546882) 106
S6 Computational Methods 109
S6.1 Optimized geometries 111
S6.1.1 Canonical Kohn-Sham Molecule orbital analysis 116
S6.1.2 Natural bond orbital (NBO) analysis 119
S6.1.3 QTAIM analysis 129
S6.1.4 Energy decomposition analysis (EDA) 141

S6.1.5 Molecular Electrostatic Potential Maps 146
S6.1.6 Non-Covalent Interactions 147
S7 References 150

S1 Materials and General Methods

Experiments were conducted under an inert nitrogen atmosphere using either a glove box (MBraun LabMaster SP or Vigor SG1800/750TS-F) or standard Schlenk techniques. Benzene, *n*-hexane, and toluene were dried over sodium benzophenone and stored under a nitrogen atmosphere over a potassium mirror. Deuterated benzene (C_6D_6) and toluene (Tol-d_8) were dried with calcium hydride (CaH_2), distilled at low pressures (< 1 × 10^{-2} mbar), and similarly stored over a potassium mirror. THF-d_8 was sourced from Cambridge Isotopes or Merck and stored under nitrogen over 3 Å molecular sieves. Methanol was dried by distillation over magnesium methoxide (prepared *in situ* from Mg turnings and I_2).

Ga ingot (99.99%) and sodium bis(trimethylsilyl)amide (NaHMDS) (95%) were purchased from Alfa Aesar, stored in a glove box, and used as received. 4,7,13,16,21,24-hexaoxa-1,10-diazabicyclo[8.8.8]hexacosane (2.2.2-crypt, also abbreviated as crypt) was purchased from Merck, distilled, and stored in a glove box. 1,4,7,10,13,16-hexaoxacyclooctadecane (18-c-6), bis(trimethylsilyl)amine HMDS(H), *n*-BuLi, methylamine (2 M solution in THF), CsF were purchased from Merck and used as received. Bottle of dry ammonia 10L, 6 bar, 99.96% (H_2O ≤100 ppm) from Air Liquide was used as received.

2-((2,6-diisopropylphenl)imido)-2-penten-4-one (abbreviated as BDI(H)),[1] β-diketiminate gallium(I) (BDI)Ga **1**,[2] LiHMDS,[3] KHMDS[3], CsHMDS[4], and (IPr)AuCl[5] were prepared according to previously reported procedures.

NMR experiments were conducted in J. Young tap NMR tubes prepared and sealed under a nitrogen atmosphere. Multinuclear NMR spectra were collected on either a Bruker AVIIIHD 400 nanobay (equipped with a 9.4 tesla magnet) or Bruker AVIII 500 (equipped with 11.7 tesla magnet and a Prodigy cryoprobe) spectrometer at 298 K unless otherwise stated. The non-deuterated solvents (fluorobenzene) were locked to an external standard sample of 10% fluorobenzene in C_6D_6. ^{7}Li and ^{133}Cs NMR data were referenced to external standard solutions of LiCl and $CsNO_3$ in D_2O (0.1 M). Assignments of NMR resonance signals in the ^{1}H and ^{13}C{^{1}H} NMR spectra were made with the help of two-dimensional NMR experiments (^{1}H,^{1}H-COSY, ^{1}H,^{13}C-HSQC, ^{1}H,^{13}C-HMBC). Chemical shifts (δ) are reported in ppm and referenced to residual solvent resonances. The coupling constants (J) were expressed in hertz. The signal multiplicities are denoted as s (singlet), d (doublet), sept (septet), m (multiplet), and br (broad).

CHN elemental microanalyses were performed at the elemental analysis services of the Metropolitan University of London using a Thermo Flash 2000 Elemental Analyzer by manipulating and preparing the samples under an inert atmosphere of pure and dry nitrogen gas inside a glove box before analysis. This was followed by the immediate transfer of the sample to the analyzer to prevent environmental exposure. Both crystalline and bulk materials of the highly reactive and air-sensitive complexes were provided in sealed glass ampoules, ensuring complete protection from atmospheric

conditions, and were analyzed in several attempts. To aid the complete combustion of specific complex samples, V_2O_5 was also incorporated. Although both crystalline and bulk forms of the highly reactive, air-sensitive complexes were analyzed multiple times, consistent results were challenging to obtain because of the inherent reactivity of these systems. This high reactivity, underscored by the small hydrolysis observed in both crystalline and bulk samples in solution by NMR spectroscopy, contributed to the challenges in obtaining meaningful elemental analysis data. Consequently, obtaining accurate elemental analysis data for such reactive complexes proved difficult, primarily because of sample decomposition and suboptimal combustion efficiency. Representative values of the organometallic species are presented in the characterization data. Despite efforts to maintain the bulk purity of the compounds, some failed to meet the ± 0.4% accuracy criteria. The complications associated with CHN combustion analysis, especially for reactive organometallic species, are well-documented and understood in the literature.[6-8]

Infrared (IR) spectra were obtained using Nujol mulls between KBr discs inside a nitrogen glove box on a Bruker Alpha FT-IR spectrometer.

S2 Synthetic Procedures and Characterization Data

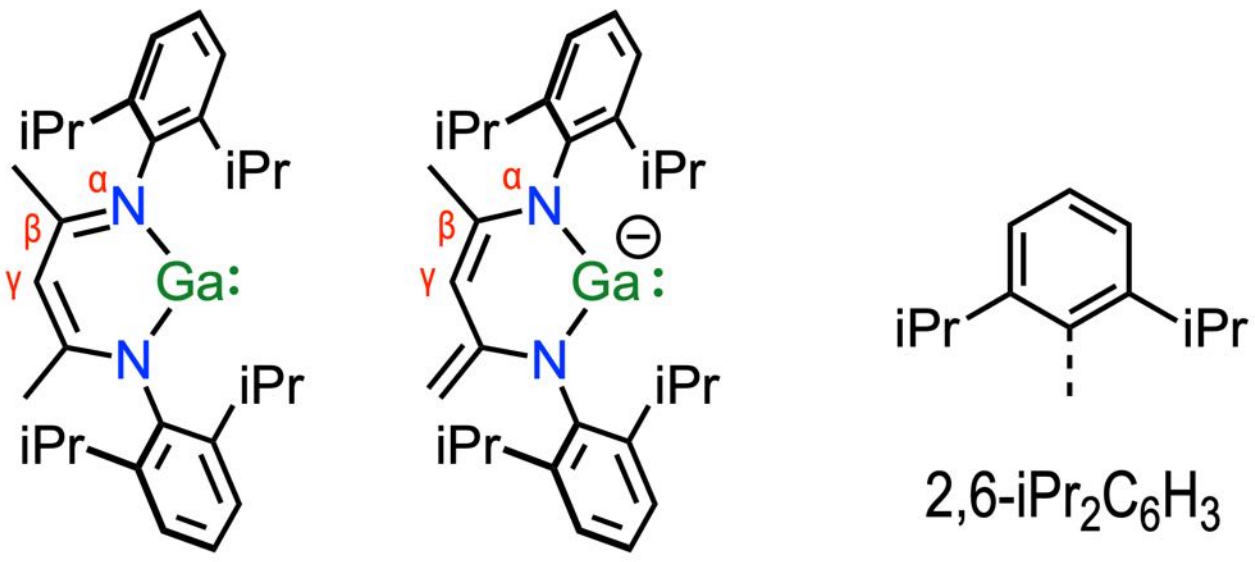

1 $\quad$ 2^{-} $\quad$ Dipp

Chart S1. Notation and abbreviations.

S2.1 Synthesis and characterization of 2·Li(crypt-222)

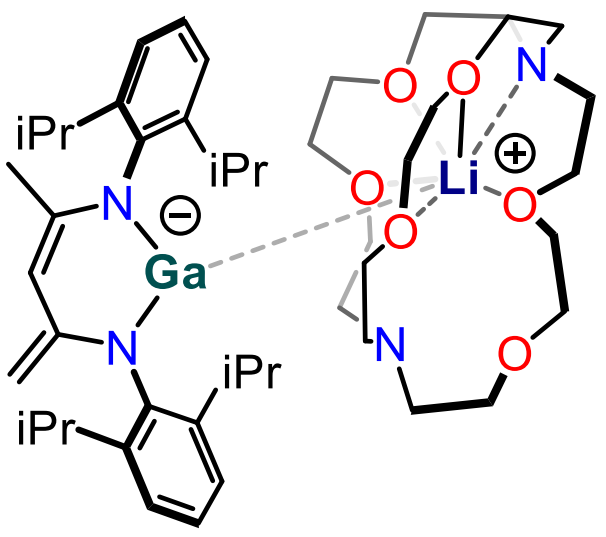

In a J. Young's ampoule, **1** (45 mg, 0.092 mmol), LiHMDS (15 mg, 0.089 mmol), and crypt (34 mg, 0.090 mmol) were combined and dissolved in toluene (1 mL). Vigorous stirring for 10 minutes yielded a pale-yellow solution alongside an orange oil. The solution was then decanted, isolating the orange oil, which upon drying under high vacuum resulted in **2·Li(crypt-222)** as an oily compound (60 mg, 0.068 mmol, 74%). This material was taken up in 0.6 mL of THF-d_8 and verified as the desired product by ^{1}H, ^{7}Li and ^{13}C{^{1}H} NMR analyses. Attempts to crystallize this compound from various solvent mixtures involving THF, *n*-hexane, benzene, toluene, and fluorobenzene were met with challenges. However, replicating the synthesis in benzene and allowing the oily compound to stand in benzene at room temperature for two days furnished a small crop of crystals suitable for single-crystal X-ray diffraction.

Due to the oily consistency of **2·Li(crypt-222)** and the difficulties in crystallizing it, conducting accurate elemental analyses was hampered, making it challenging to obtain precise compositional data. Nevertheless, the NMR and X-ray diffraction data supports the chemical identity of **2·Li(crypt-222)**.

<u>Note on NMR data</u>: The NMR spectroscopic data of **2·Li(crypt-222)** in solution, obtained using ^{1}H, ^{7}Li, ^{13}C{^{1}H} and DOSY, is consistent (identical), regardless of its physical form, either orange oil or orange prism-shaped crystals, of **2·Li(crypt-222)**. The ^{7}Li NMR spectrum of **2·Li(crypt-222)** displays two broad resonances at δ 1.75 and 0.52, indicating dynamic behavior and mobility of the Li^+ within the puckered crypt cavity at room temperature.

^{1}H NMR (500 MHz, THF-d_8, 298 K): *δ* 6.95 (d, 2 H, J = 7.6 Hz, Ar), 6.93 (d, 2 H, J = 7.6 Hz, Ar), 6.81 (t, 2 H, J = 7.5 Hz, Ar), 4.52 (s, 1 H, γ-CH), 3.70 (m, 4 H, CH iPr), 3.59 (s, 12 H, crypt), 3.54 (br s, 12 H, crypt), 2.68 (d, 1 H, J = 2.7 Hz, CH=C), 2.61 (br s, 12 H, crypt), 1.94 (d, 1 H, J = 2.7 Hz, CH=C), 1.40 (s, 3 H, β-Me), 1.18 (m, 24 H, Me iPr).

$^{13}C\{^1H\}$ NMR (125.6 MHz, THF-d_8, 298 K): δ 157.3 (C_q, *C*=CH_2), 151.0 (C_q, Ar), 147.6 (C_q, Ar),147.2 (C_q, Ar), 143.4 (β-*C*-Me), 142.6 (C_q, Ar), 122.8 (CH, Ar), 122.7 (CH, Ar), 122.6 (CH, Ar),122.4 (CH, Ar), 100.4 (*γ*-CH), 71.8 (br s, crypt), 70.8 (br s, crypt), 69.6 (CH_2=C), 69.0 (br s, crypt), 57.6 (br s, crypt), 54.2 (br s, crypt), 28.2 (CH, iPr), 28.1 (CH, iPr), 26.6 (Me, iPr), 26.6 (Me, iPr), 25.8 (Me, iPr), 24.2 (β-Me).

$^7Li\{^1H\}$ NMR (194.2 MHz, THF-d_8, 298 K): δ 1.75 (s), 0.52 (s).

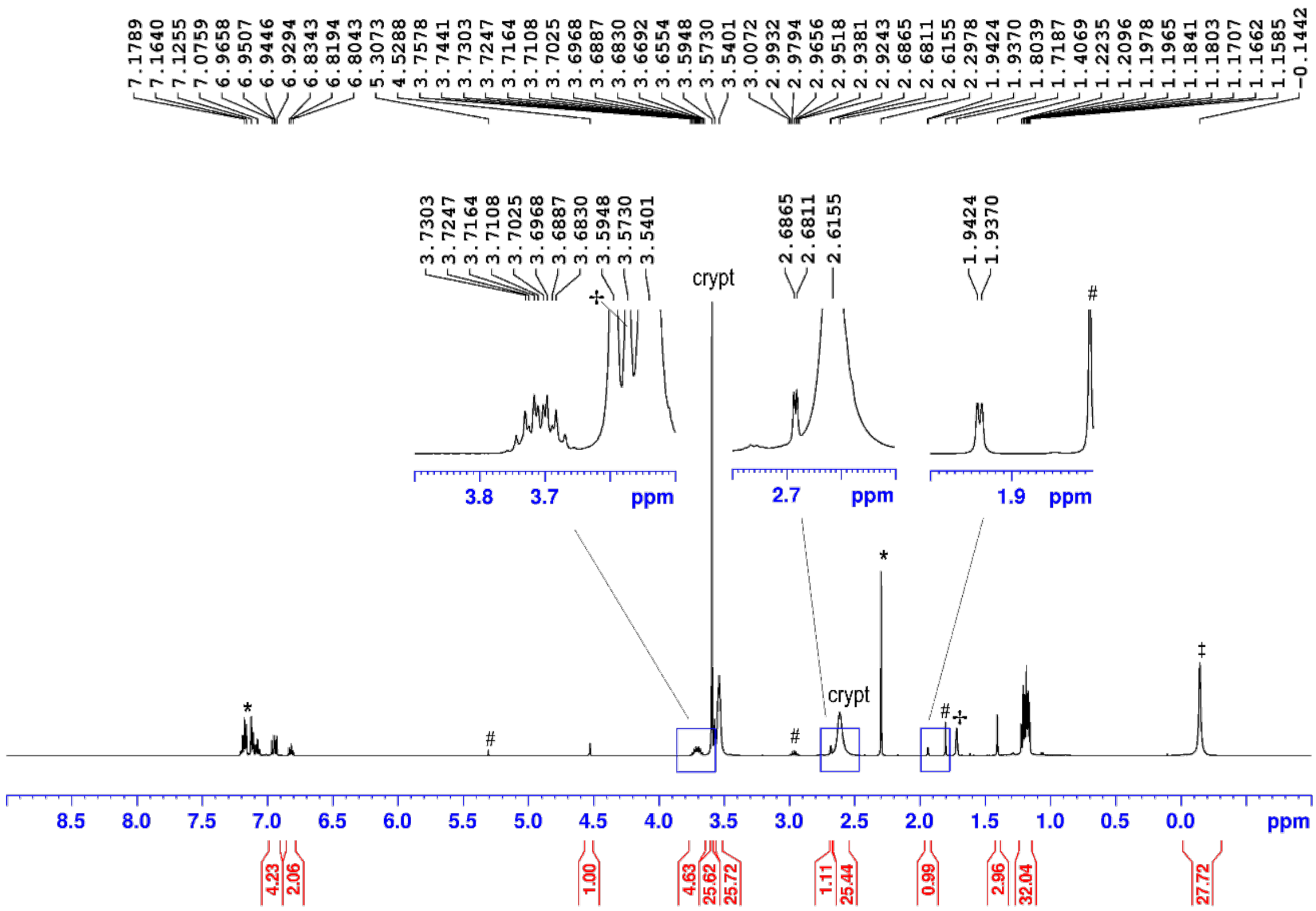

Figure S1. ^{1}H NMR (500 MHz, THF-d_8, 298 K) spectrum of **2·Li(crypt-222)**. The symbols (*) and (∔) denote residual C_6D_6 and THF-d_8 solvent peaks, respectively. The symbol (#) represents traces of **1** resulting from unavoidable hydrolysis processes in solution. Additionally, (‡) represents traces of HMDS(H) occluded within the oily compound.

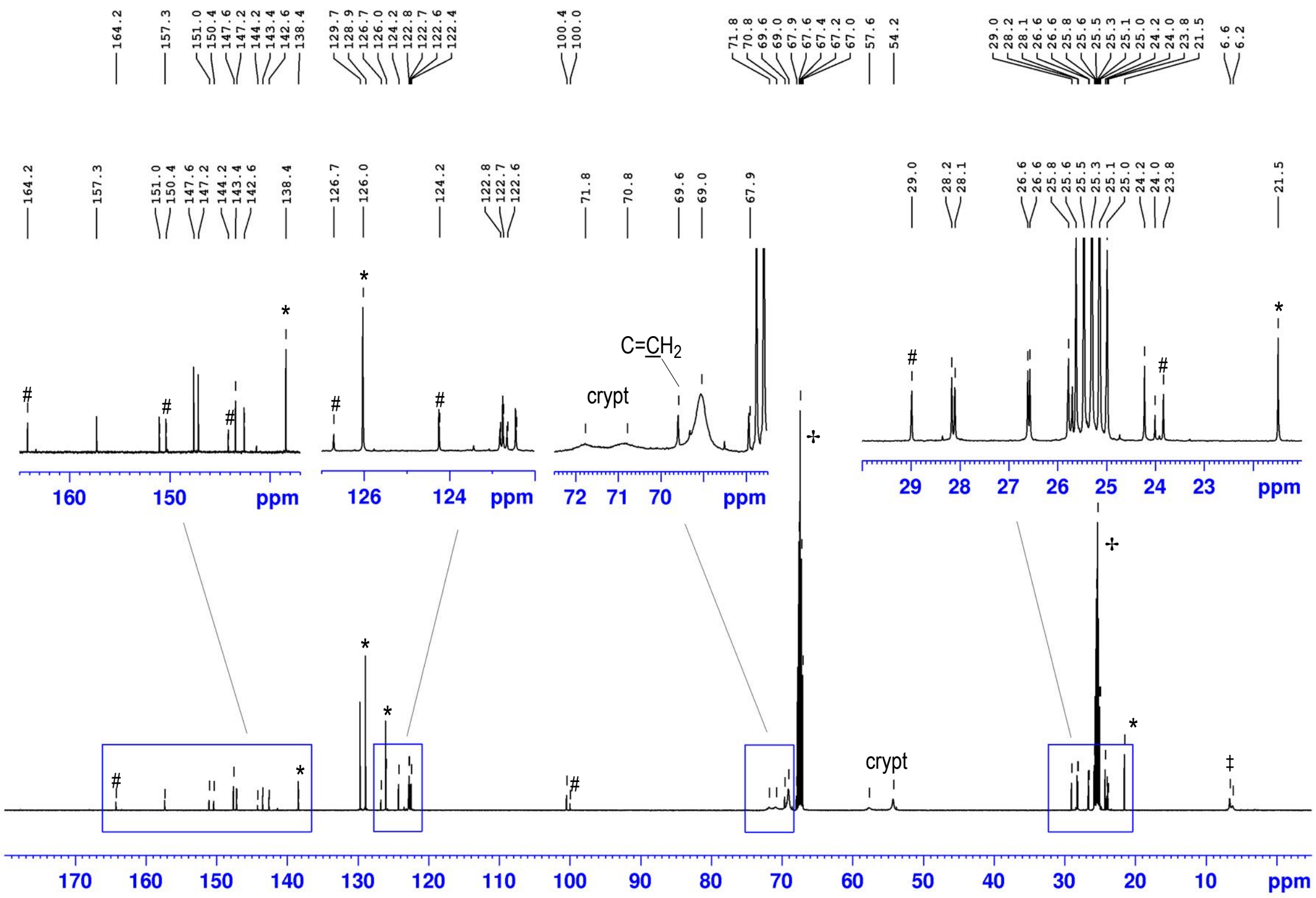

Figure S2. $^{13}C\{^{1}H\}$ NMR (125.6 MHz, THF-d_8, 298 K) spectrum of **2·Li(crypt-222)**. The symbols (*) and (+) denote residual C_6D_6 and THF-d_8 solvent peaks, respectively. ‡ Represents traces of HMDS(H) occluded within the oily compound, and # represents traces of **1** resulting from unavoidable hydrolysis processes in solution.

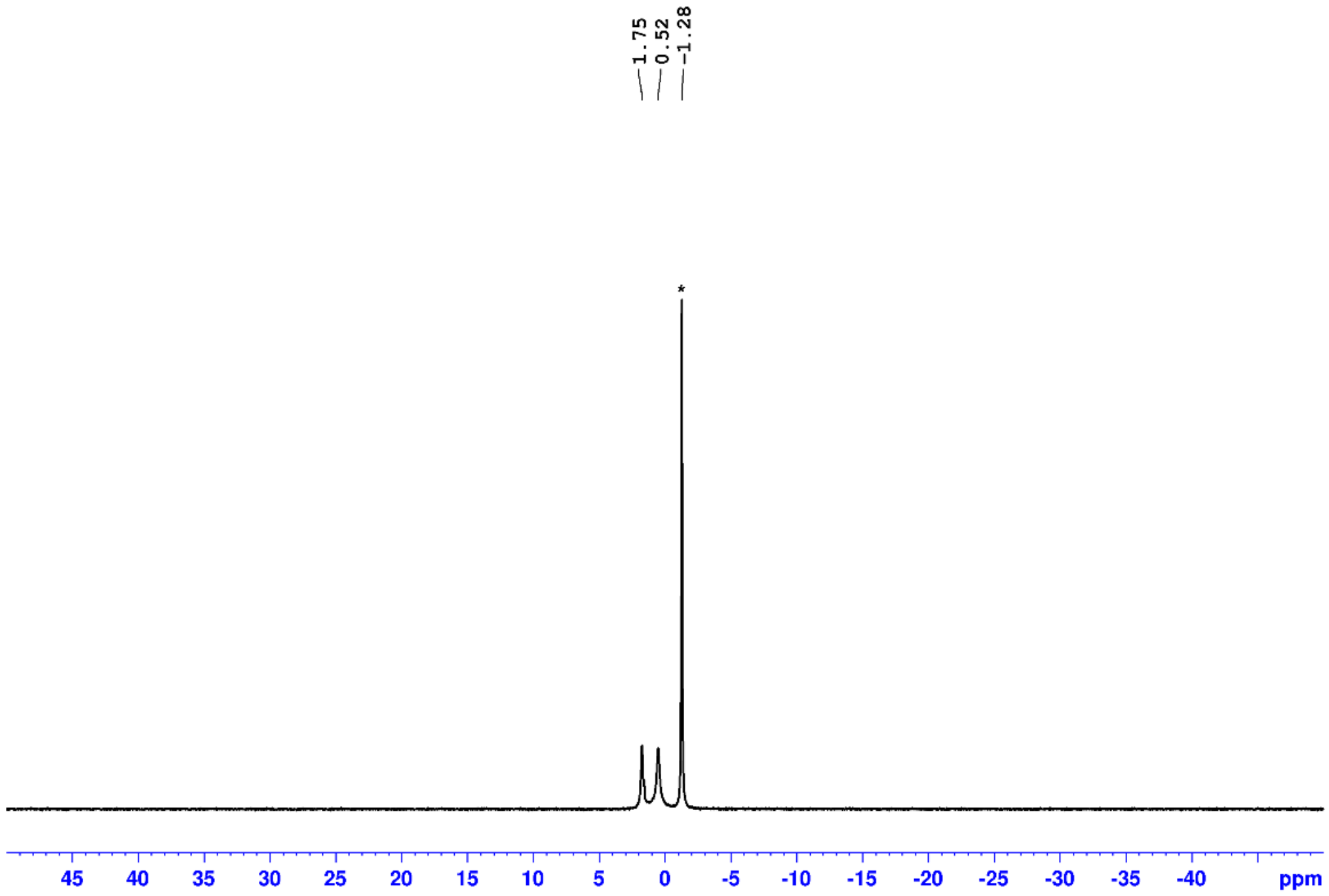

Figure S3. $^{7}Li\{^{1}H\}$ NMR (194.2 MHz, THF-d_8, 298 K) spectrum of **2·Li(crypt-222)**. The resonance marked with (*) accounts for residual amounts of LiHMDS occluded within the oily compound.

S2.2 Synthesis and characterization of 2·Na(crypt-222)

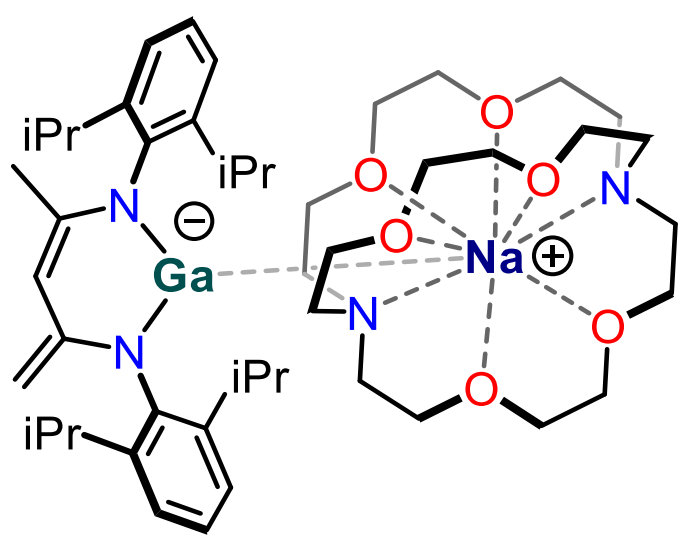

In a J. Young's ampoule, **1** (76 mg, 0.156 mmol), NaHMDS (60 mg, 0.327 mmol), and crypt (56 mg, 0.149 mmol) were combined and dissolved in benzene (1.5 mL). After stirring vigorously for 10 minutes, a yellow solution accompanied by an orange oil was observed. Tetrahydrofuran (0.6 mL) was then added, resulting in an orange solution. The solution volume was reduced until crystallization incipient crystallization. Orange crystals of **2·Na(crypt-222)** were subsequently isolated by filtration, dried under vacuum (< 10^{-2} mbar) for 2 hours and isolated (30 mg, 0.034 mmol, 22%). Crystals suitable for single-crystal X-ray diffraction were prepared by slowly diffusing *n*-hexane (1 mL) into a benzene solution (0.5 mL) containing **2·Na(crypt-222)**.

^{1}H NMR (500 MHz, 4:1 C_6D_6:THF-d_8, 298 K): δ 7.34 (d, 2 H, J = 7.6 Hz, Ar), 7.24 (d, 2 H, J = 7.6 Hz, Ar), 7.16 (t overlapped with solvent peak, 1 H, Ar), 7.10 (t, 1 H, J = 7.5 Hz, Ar), 5.26 (s, 1 H, γ-CH), 4.28 (sept, 2 H, J = 6.9 Hz, CH iPr), 4.17 (sept, 2 H, J = 6.9 Hz, CH iPr), 3.46 (d, 1 H, J = 2.6 Hz, CH=C), 3.05 (s, 12 H, crypt), 3.00 (t, 12 H, J_1 = 4.8 Hz, crypt), 2.71 (d, 1 H, J = 2.6 Hz, CH=C), 2.04 (t, 12 H, J_1 = 4.8 Hz, crypt), 1.88 (s, 3 H, β-Me), 1.58 (m, 2 H, Me iPr), 1.53 (d, 6 H, J = 6.9 Hz, Me iPr), 1.41 (d, 6 H, J = 6.9 Hz, Me iPr).

^{13}C{^{1}H} NMR (125.6 MHz, 4:1 C_6D_6:THF-d_8, 298 K): δ 157.4 (C_q, *C*=CH_2), 151.0 (C_q, Ar), 150.1 (C_q, Ar), 147.7 (C_q, Ar),147.1 (C_q, Ar), 143.2 (β-*C*-Me), 122.9 (CH, Ar), 122.8 (CH, Ar), 122.8 (CH, Ar),122.5 (CH, Ar), 100.6 (*γ*-CH), 70.3 (*CH_2*=C), 68.6 (crypt), 67.7 (crypt), 53.0 (crypt), 28.1 (CH, iPr), 28.1 (CH, iPr), 26.7 (Me iPr), 26.7 (Me iPr), 25.9 (Me iPr), 25.2 (β-Me), 24.2 (Me iPr).

Elemental analysis found (calc.) for $C_{47}H_{76}GaNaN_4O_6$: C 63.26 (63.73), H 8.81 (8.65), N 6.49 (6.32).

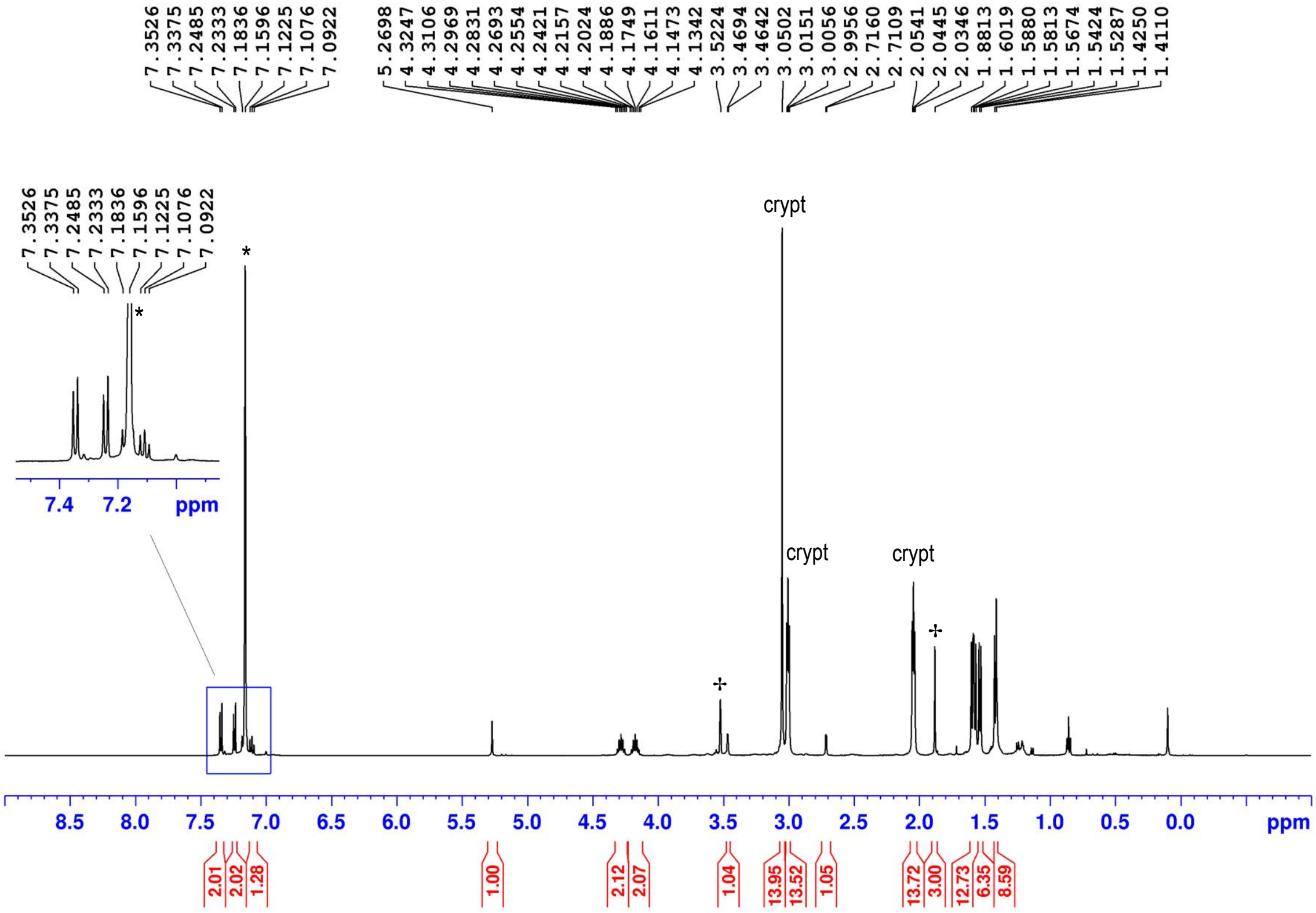

Figure S4. ^{1}H NMR (500 MHz, C_6D_6:THF-d_8, 298 K) spectrum of **2·Na(crypt-222)**. The symbols (*) and (+) denote residual C_6D_6 and THF-d_8 solvent peaks, respectively.

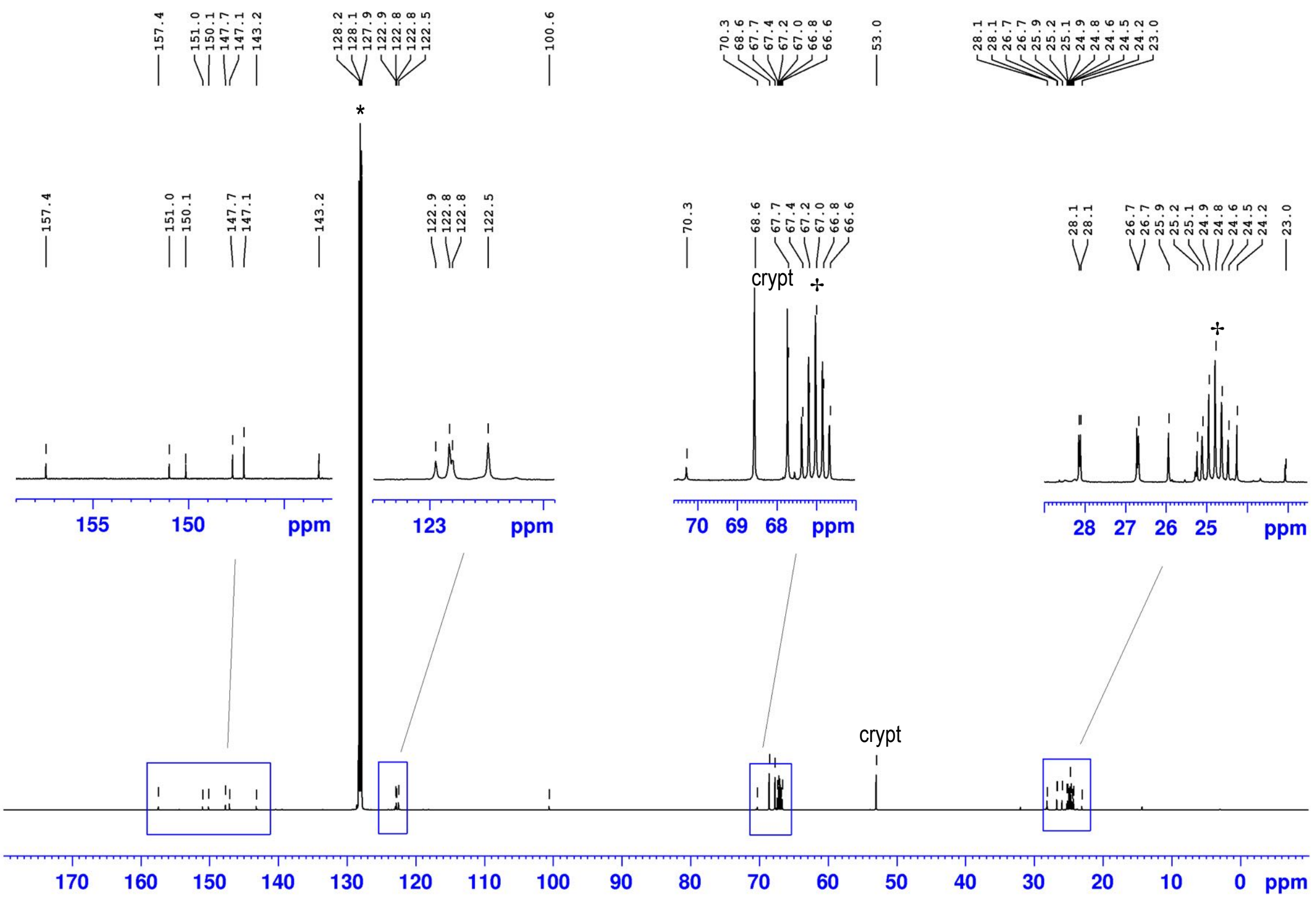

Figure S5. ^{13}C{^{1}H} NMR (125.6 MHz, C_6D_6:THF-d_8, 298 K) spectrum of **2·Na(crypt-222)**. The symbols (*) and (+) denote residual C_6D_6 and THF-d_8 solvent peaks, respectively.

S2.3 Synthesis and characterization of 2·K(crypt-222)

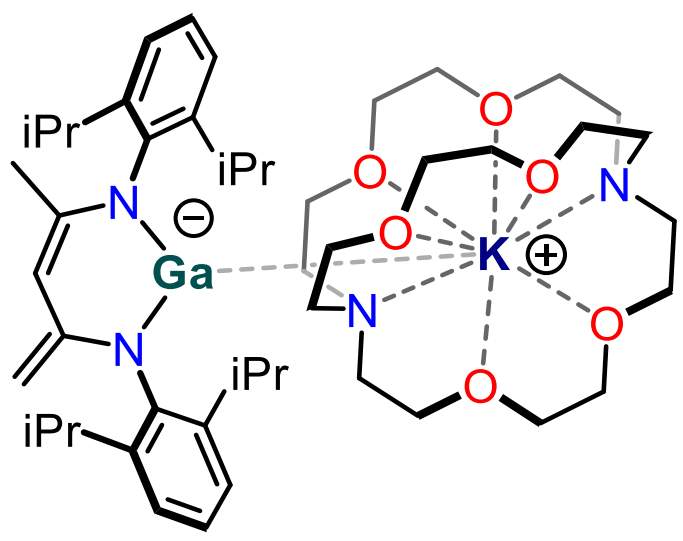

In a J. Young's ampoule, **1** (56 mg, 0.115 mmol), KHMDS (33 mg, 0.165 mmol), and crypt (40 mg, 0.106 mmol) were combined and dissolved in dry toluene (1 mL). After stirring vigorously for 10 minutes at room temperature, left-standing orange prism-shaped crystals were obtained after 2 hours. These crystals were filtered with a cannula and dried under vacuum (< 10^{-2} mbar) for 2 hours, affording **2·K(crypt-222)** orange prism-shaped crystals (35 mg, 0.040 mmol, 35%). These crystals were suitable for single-crystal X-ray diffraction.

^{1}H NMR (500 MHz, 4:1 C_6D_6:THF-*d*$_8$, 298 K): *δ* 7.32 (d, 2 H, J = 7.6 Hz, Ar), 7.21 (d, 2 H, J = 7.6 Hz, Ar), 7.14 (t overlapped with C_6D_6 residual solvent peak, 1 H, J = 7.6 Hz, Ar), 7.08 (t, 1 H, J = 7.6 Hz, Ar), 5.25 (s, 1 H, γ-CH), 4.27 (sept, 2 H, J = 6.9 Hz, CH iPr), 4.16 (sept, 2 H, J = 6.9 Hz, CH iPr), 3.45 (d, 1 H, J = 2.6 Hz, CH=C), 3.07 (s, 12 H, crypt), 3.01 (t, 12 H, J = 4.6 Hz, crypt), 2.69 (d, 1 H, J = 2.6 Hz, CH=C), 2.03 (t, 12 H, J = 4.6 Hz, crypt), 1.86 (s, 3 H, β-Me), 1.57 (d, 6 H, J = 6.9 Hz, Me iPr), 1.55 (d, 6 H, J = 6.9 Hz, Me iPr), 1.51 (d, 6 H, J = 6.9 Hz, Me iPr), 1.40 (d, 6 H, J = 6.9 Hz, Me iPr).

^{13}C{^{1}H} NMR (125.6 MHz, 4:1 C_6D_6:THF-*d*$_8$, 298 K): *δ* 157.4 (C_q, *C*=CH_2), 151.1 (C_q, Ar), 150.3 (C_q, Ar), 147.7 (C_q, Ar),147.1 (C_q, Ar), 143.1 (C_q, β-*C*-Me), 122.9 (CH, Ar), 122.8 (CH, Ar), 122.8 (CH, Ar), 122.4 (CH, Ar), 100.6 (*γ*-CH), 70.5 (crypt), 70.3 (*CH_2*=C), 67.3 (crypt), 53.8 (crypt), 28.1 (CH, iPr), 28.1 (CH, iPr), 26.7 (Me, iPr), 26.6 (Me, iPr), 25.9 (Me, iPr), 25.2 (β-Me), 24.6 (Me, iPr).

Elemental analysis found (calc.) for $C_{47}H_{76}GaKN_4O_6$: C 58.23 (62.59), H 8.82 (8.49), N 6.04 (6.21).

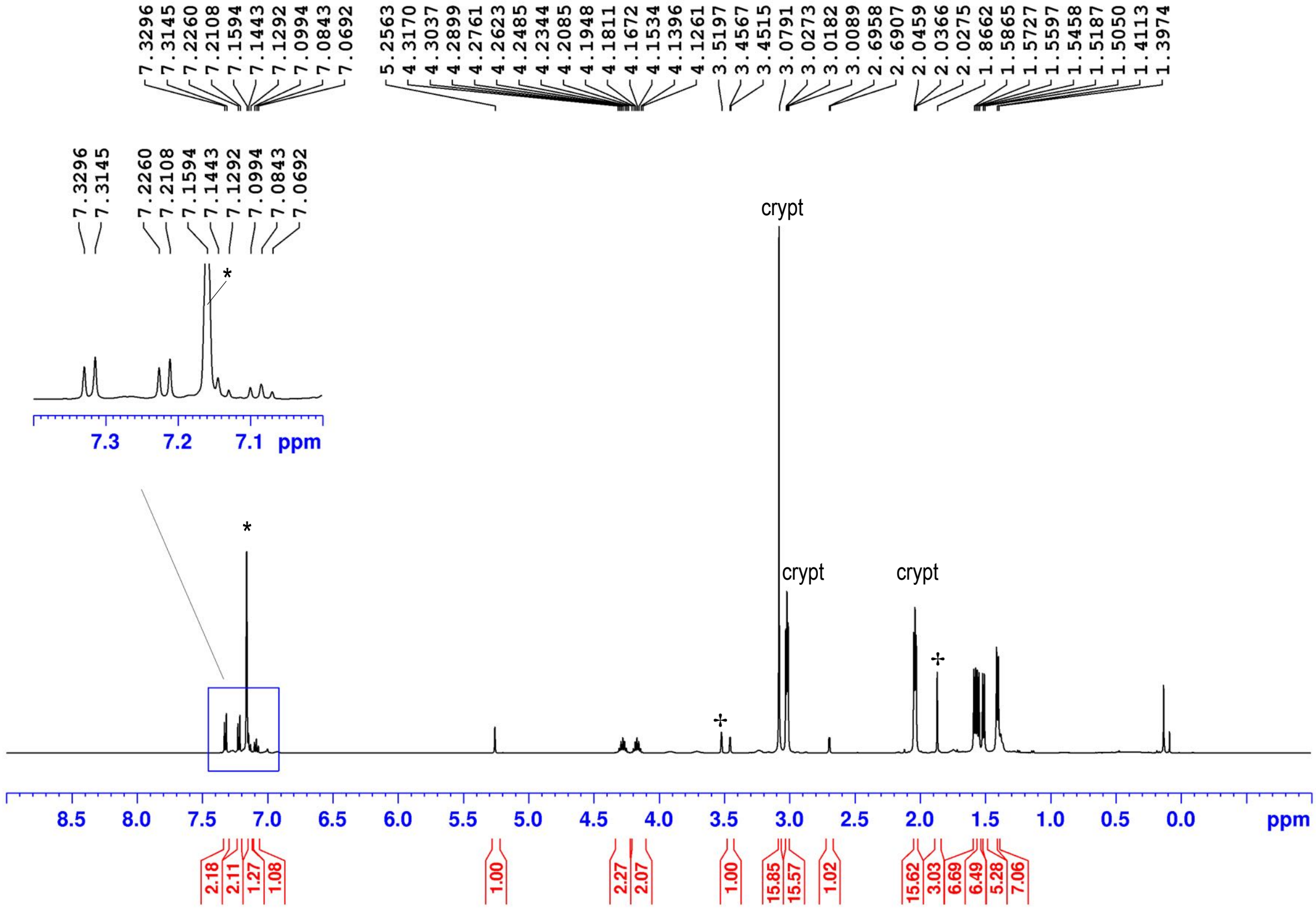

Figure S6. ^{1}H NMR (500 MHz, C_6D_6:THF-d_8, 298 K) spectrum of **2·K(crypt-222)**. The symbols (*) and (∔) denote residual C_6D_6 and THF-d_8 solvent peaks, respectively.

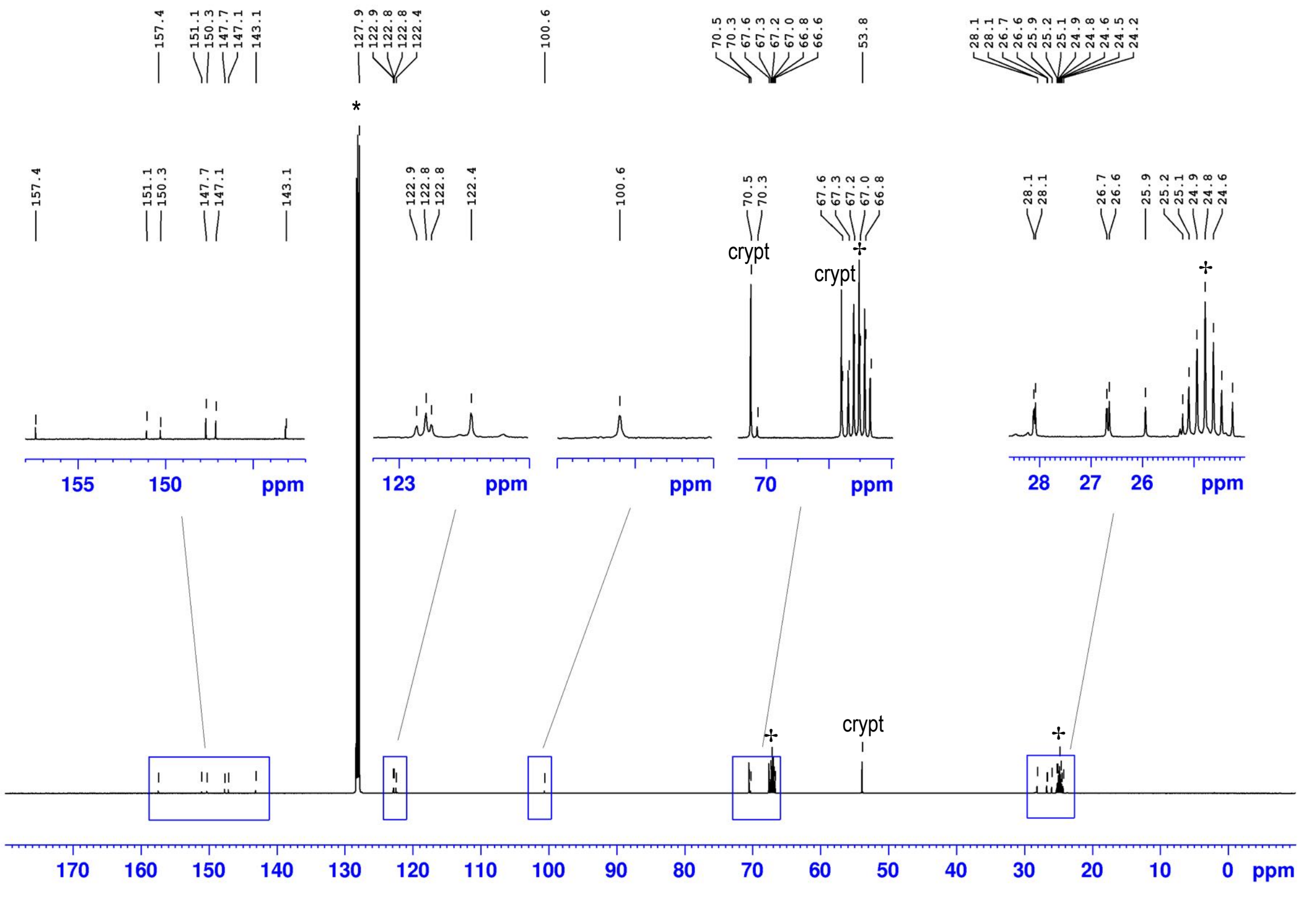

Figure S7. $^{13}C\{^{1}H\}$ NMR (125.6 MHz, C_6D_6:THF-d_8, 298 K) spectrum of **2·K(crypt-222)**. The symbols (*) and (∔) denote residual C_6D_6 and THF-d_8 solvent peaks, respectively.

S2.4 Synthesis and characterization of 2·Cs(crypt-222)

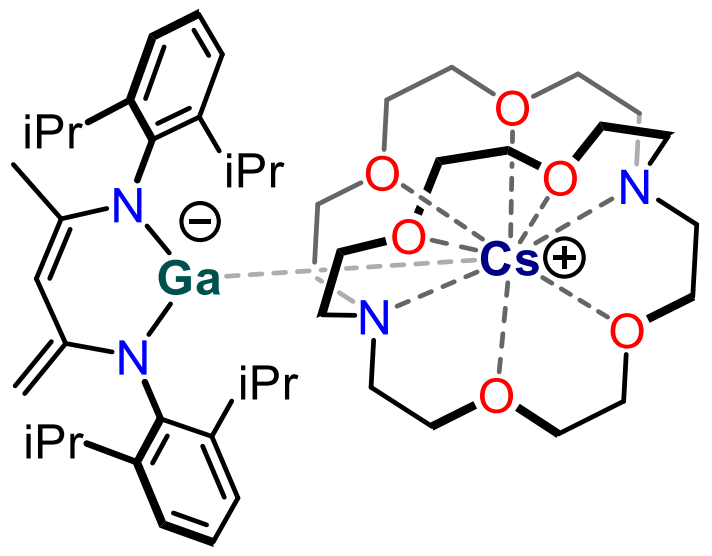

In a J. Young's ampoule, **1** (156 mg, 0.320 mmol), CsHMDS (116 mg, 0.395 mmol), and crypt (130 mg, 0.345 mmol) were combined and dissolved in benzene (1.5 mL). The mixture was stirred at room temperature for 5 hours. The resulting yellow solution was then filtered via cannula and layered with *n*-hexane (5 mL). After two days, orange plate-shaped crystals formed. These crystals were dried under vacuum (< 10^{-2} mbar) for 1 hour, affording **2·Cs(crypt-222)** (200 mg, 0.226 mmol, 71%). These crystals were suitable for single-crystal X-ray diffraction.

^{1}H NMR (500 MHz, 4:1 C_6D_6:THF-d_8, 298 K): *δ* 7.19 (d, 2 H, J = 7.5 Hz, Ar), 7.11 (d, 2 H, J = 7.5 Hz, Ar), 7.02 (t, 1 H, J_1 = 7.5 Hz, Ar), 6.98 (t, 1 H, J = 7.5 Hz, Ar), 5.11 (s, 1 H, γ-CH), 4.11 (sept, 2 H, J = 6.9 Hz, CH iPr), 4.03 (sept, 2 H, J = 6.9 Hz, CH iPr), 3.34 (d, 1 H, J = 2.5 Hz, CH=C), 3.16 (s, 12 H, crypt), 3.07 (t, 12 H, J = 4.6 Hz, crypt), 2.54 (d, 1 H, J = 2.5 Hz, CH=C), 2.14 (t, 12 H, J = 4.6 Hz, crypt), 1.73 (s, 3 H, β-Me), 1.44 (d, 6 H, J = 6.9 Hz, Me iPr), 1.39 (d, 6 H, J = 6.9 Hz, Me iPr), 1.37 (d, 6 H, J = 6.9 Hz, Me iPr), 1.31 (d, 6 H, J = 6.9 Hz, Me iPr).

^{13}C{^{1}H} NMR (125.6 MHz, 4:1 C_6D_6:THF-d_8, 298 K): *δ* 157.0 (C_q, *C*=CH_2), 151.2 (C_q, Ar), 150.2 (C_q, Ar), 147.9 (C_q, Ar),147.2 (C_q, Ar), 142.9 (β-*C*-Me), 122.9 (CH, Ar), 122.8 (CH, Ar), 122.7 (CH, Ar), 122.3 (CH, Ar), 100.6 (*γ*-CH), 70.6 (*CH_2*=C and crypt, overlapped signals), 68.7 (crypt), 55.6 (crypt), 28.0 (CH, iPr), 26.4 (Me, iPr), 25.7 (Me, iPr), 25.3 (β-Me), 24.1 (Me, iPr).

^{133}Cs{^{1}H} NMR (52.4 MHz, 4:1 C_6D_6:THF-d_8, 298 K): δ 193.4 (br s, Δν = 80 Hz).

Elemental analysis found (calc.) for $C_{47}H_{76}CsGaN_4O_6$: C 55.54 (56.69), H 7.83 (7.69), N 5.48 (5.63).

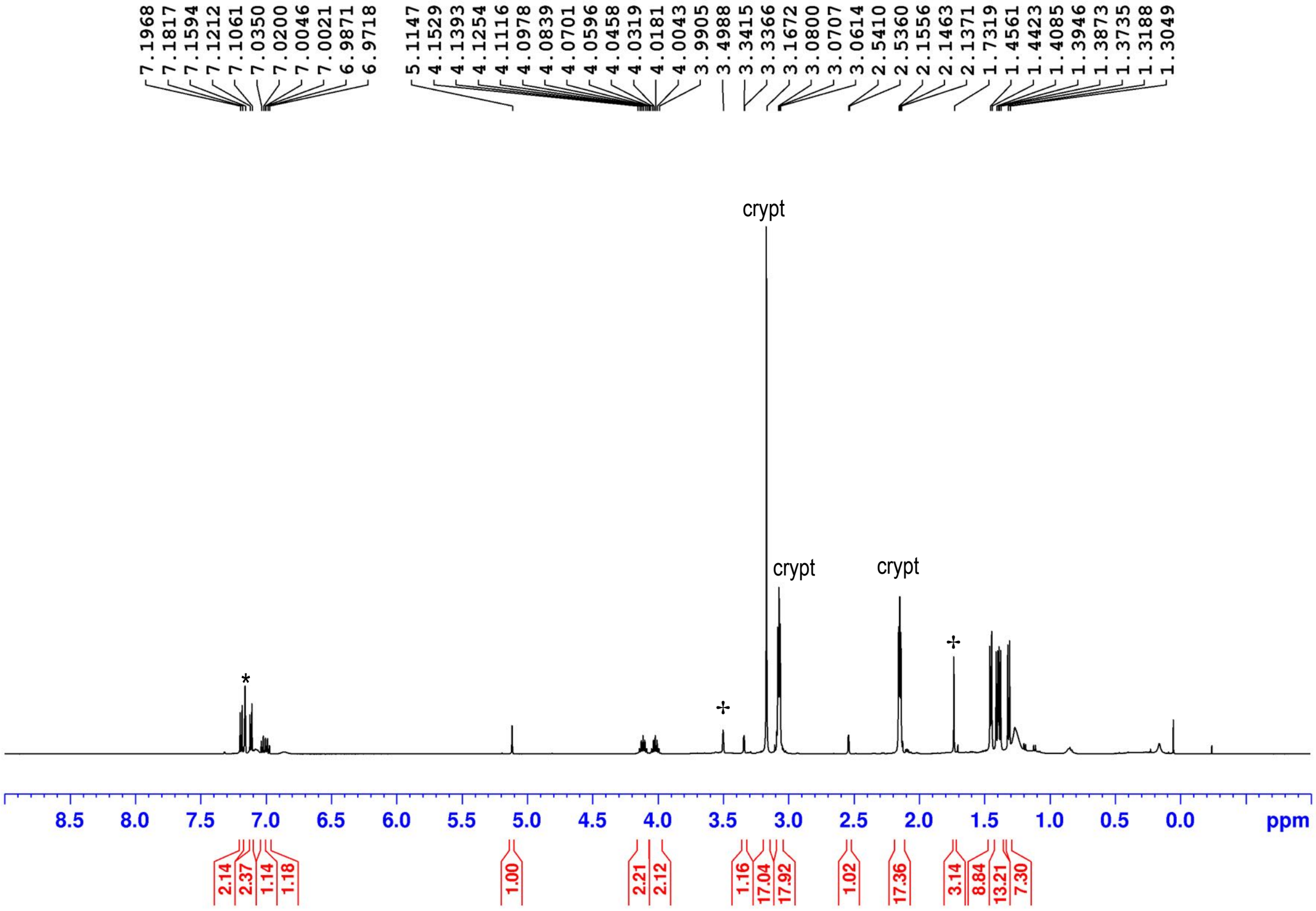

Figure S8. ^{1}H NMR (500 MHz, C_6D_6:THF-d_8, 298 K) spectrum of **2·Cs(crypt-222)**. The symbols (*) and (✢) denote residual C_6D_6 and THF-d_8 solvent peaks, respectively.

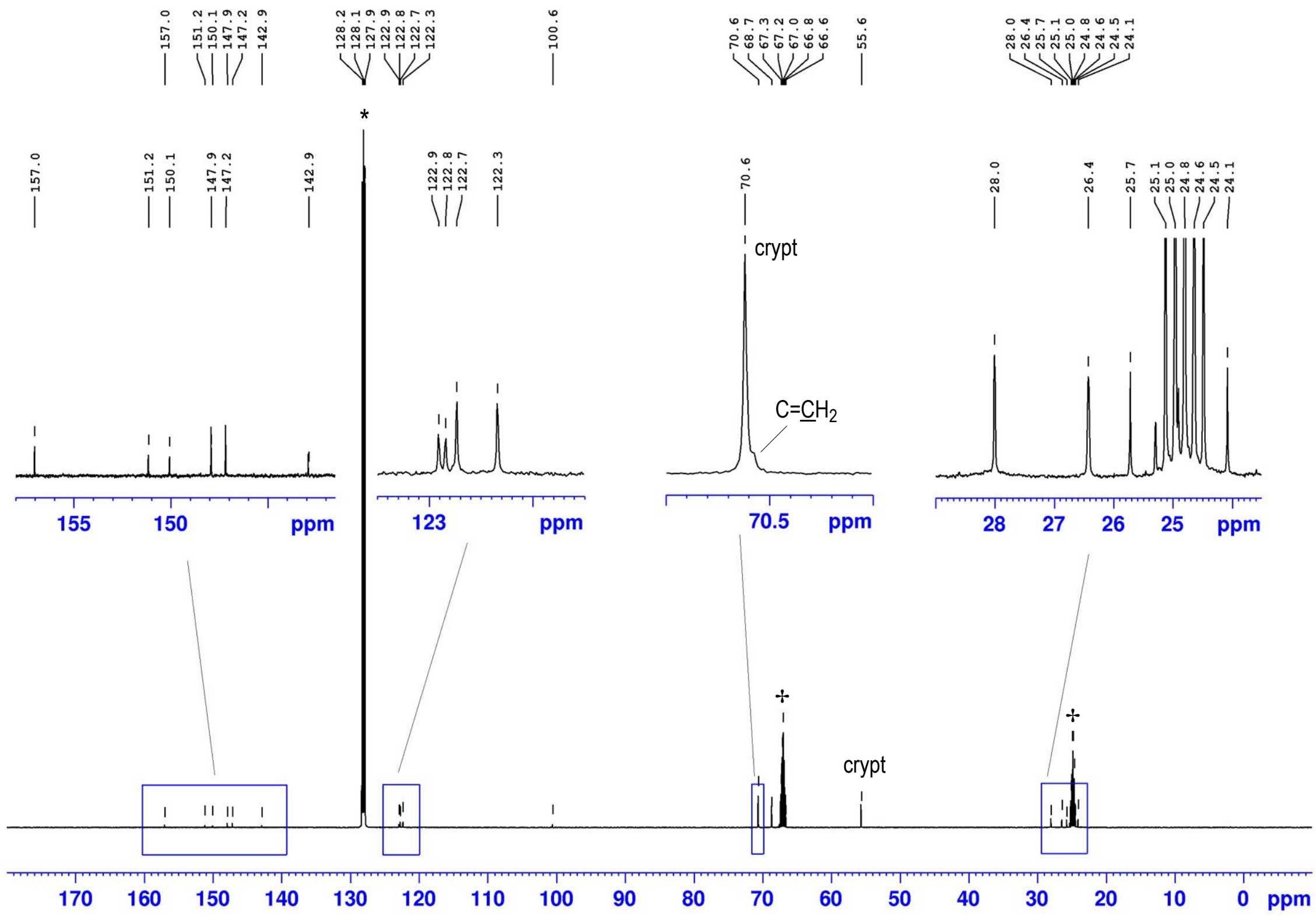

Figure S9. ^{13}C{^{1}H} NMR (125.6 MHz, C_6D_6:THF-d_8, 298 K) spectrum of **2·Cs(crypt-222)**. The symbols (*) and (✢) denote residual C_6D_6 and THF-d_8 solvent peaks, respectively.

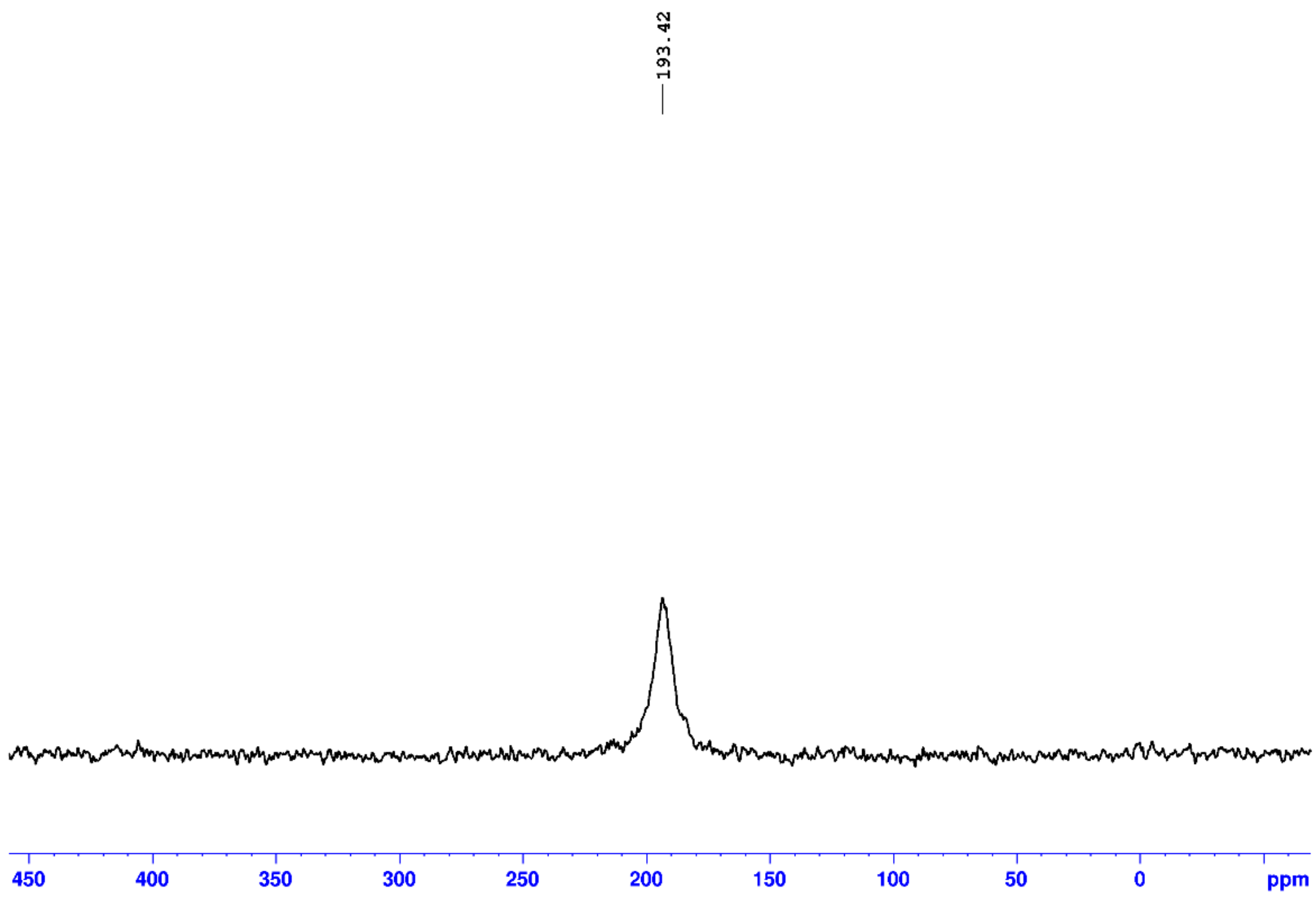

Figure S10. ^{133}Cs NMR (52.4 MHz, C_6D_6:THF-d_8, 298K) spectrum of **2·Cs(crypt-222)**.

S2.5 Synthesis and characterization of 2·Na(18-c-6)

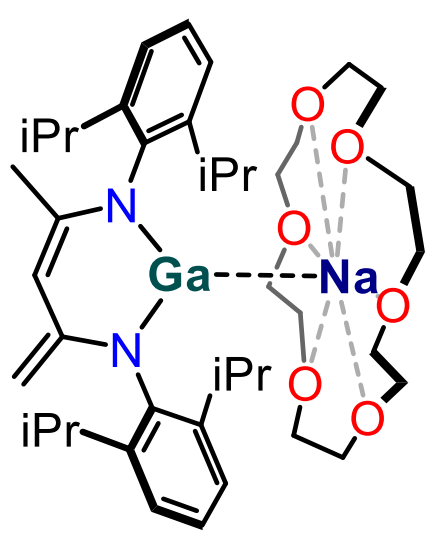

In a J. Young's ampoule, **1** (77 mg, 0.158 mmol), NaHMDS (50 mg, 0.273 mmol), and 18-crown-6 (40 mg, 0.151 mmol) were combined and dissolved in benzene (2 mL). The mixture was stirred at room temperature. Progress of the reaction was monitored by ^{1}H NMR, and after 2 hours, the reaction was complete. The resulting yellow solution was filtered via cannula followed. Layering with *n*-hexane (5 mL) gave orange prism-shaped crystals after two days at room temperature. These crystals were dried under vacuum (< 10^{-2} mbar) for 1 hour, affording **2·Na(18-c-6)** (40 mg, 0.052 mmol, 33%). These crystals were suitable for single-crystal X-ray diffraction.

^{1}H NMR (400 MHz, C_6D_6, 298 K): δ 7.29 (d, 2 H, J = 7.6 Hz, Ar), 7.13 (m overlapped with solvent peak, 3 H, Ar), 7.02 (t, 1 H, J = 7.2 Hz, Ar), 5.60 (s, 1 H, γ-CH), 4.28 (sept, 2 H, J = 6.9 Hz, CH iPr), 4.11 (sept, 2 H, J = 6.9 Hz, CH iPr), 3.93 (d, 1 H, J = 2 Hz, CH=C), 3.00 (d, 1 H, J = 2 Hz, CH=C), 2.92 (s, 24 H,18-cr-6), 1.88 (s, 3 H, β-Me), 1.65 (d, 6 H, J = 6.9 Hz, Me iPr), 1.39 (m, 18 H, Me iPr).

^{13}C{^{1}H} NMR NMR (100.6 MHz, C_6D_6, 298 K): δ 156.6 (C_q, *C*=CH_2), 150.3 (C_q, Ar), 148.1 (C_q, Ar), 147.7 (C_q, Ar), 143.0 (β-*C*-Me), 124.0 (CH, Ar), 123.2 (CH, Ar), 122.6 (CH, Ar), 101.8 (*γ*-CH), 73.6 (*CH_2*=C), 69.1 (18-c-6), 28.1 (CH, iPr), 28.1 (CH, iPr), 26.1 (Me, ^{i}Pr), 25.9 (Me, ^{i}Pr), 24.1 (β-Me).

Elemental analysis found (calc.) for $C_{41}H_{64}GaNaN_2O_6$: C 62.81 (63.65), H 8.50 (8.34), N 3.62 (3.62).

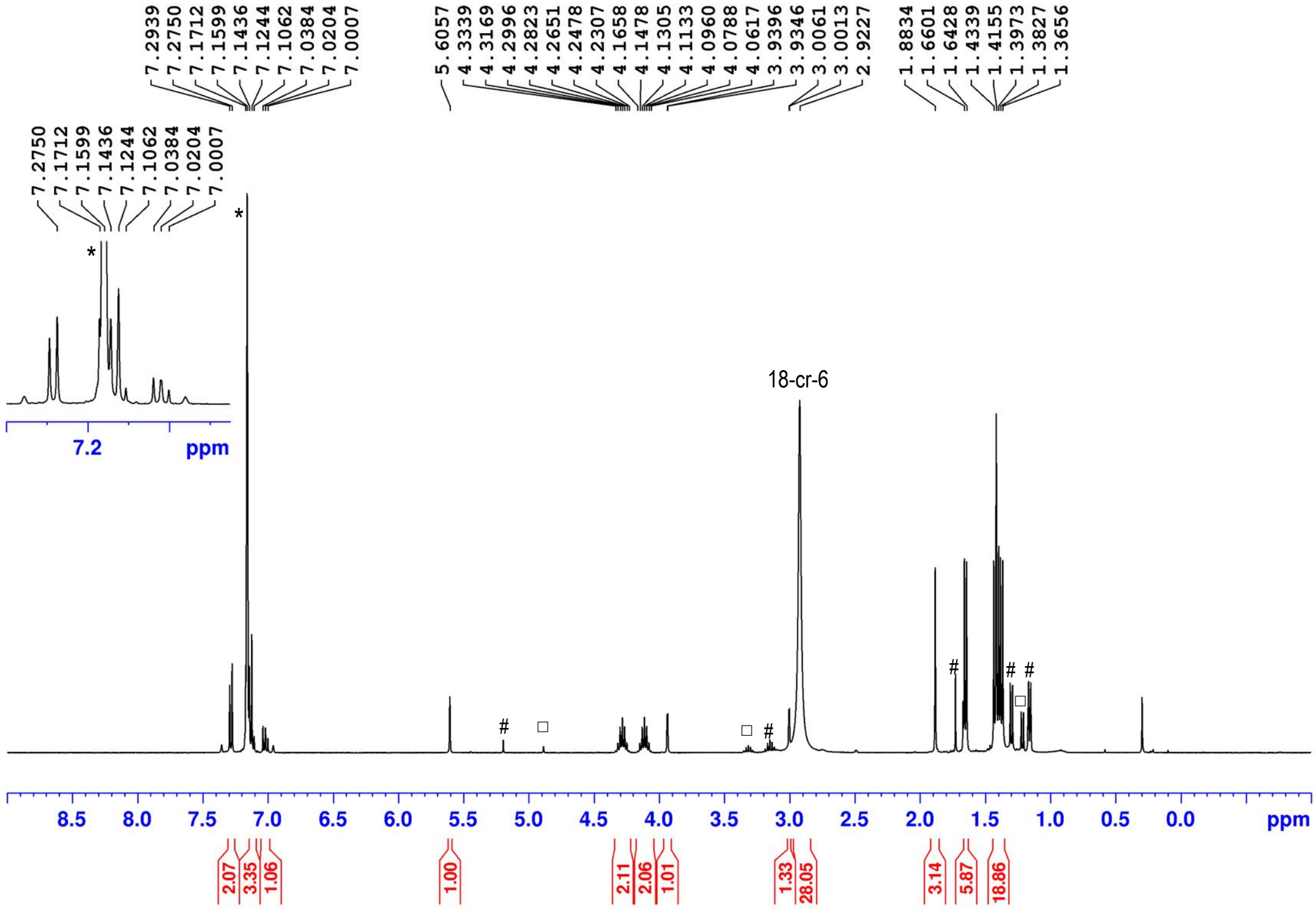

Figure S11. ^{1}H NMR (400 MHz, C_6D_6, 298 K) of **2·Na(18-c-6)**. The symbol (#) denotes the minor unavoidable hydrolysis product **1**, (□) for BDI(H), and (*) for the residual C_6D_6 solvent peak.

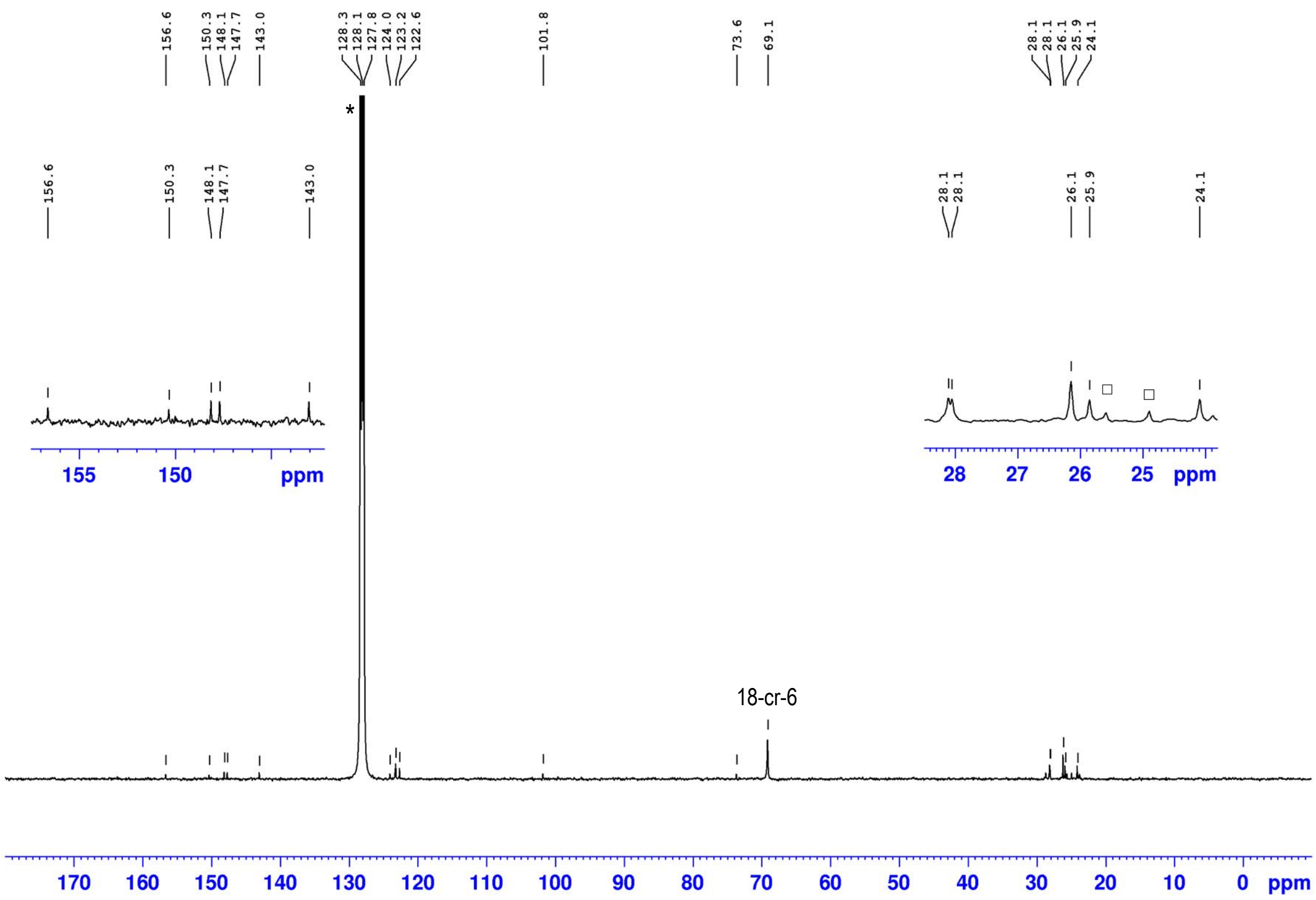

Figure S12. $^{13}C\{^{1}H\}$ NMR (125.6 MHz, C_6D_6, 298 K) spectrum of **2·Na(18-c-6)**. The symbol (*) represents the residual C_6D_6 solvent peak.

S2.6 Synthesis and characterization of 2·K(18-c-6)

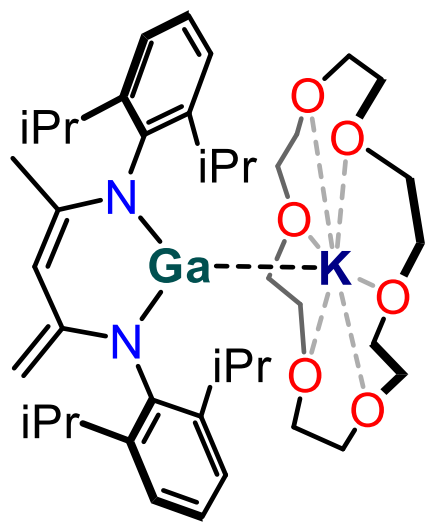

In a J. Young's ampoule, **1** (179 mg, 0.367 mmol), KHMDS (108 mg, 0.541 mmol), and 18-crown-6 (100 mg, 0.378 mmol) were combined and dissolved in toluene (2 mL). The mixture was stirred at room temperature for 2 hours. Afterward, the resulting yellow suspension was filtered using a cannula. The isolated yellow solid was dried under a high vacuum (< 10^{-2} mbar) for 2 hours to afford **2·K(18-c-6)** as a green-yellow solid (165 mg, 0.209 mmol, 57%). Crystals suitable for single-crystal X-ray diffraction of **2·K(18-c-6)** were achieved by slowly diffusing *n*-hexane (1 mL) into its benzene solution (0.5 mL). Under these conditions, two distinct crystallographic polymorphs of **2·K(18-c-6)** were isolated: one crystallized as orange prisms (dimer) and the other as orange needles (trimer). Further details can be found in S5.

Note on NMR data: Regardless of the crystallized polymorph type, the ^{1}H, ^{13}C{^{1}H}, and DOSY NMR data for **2·K(18-c-6)** in solution were identical. The only discrepancy was observed with benzene, which was incorporated within the trimer polymorph as a solvent molecule, while the dimer existed in a solvent-free arrangement. For a comprehensive discussion, refer to S5.

^{1}H NMR (500 MHz, C_6D_6, 298 K): *δ* 7.29 (d, 2 H, J = 7.6 Hz, Ar), 7.14 (m overlapped with solvent peak, 3 H, Ar), 7.03 (t, 1 H, J = 7.5 Hz, Ar), 5.57 (s, 1 H, γ-CH), 4.31 (sept, 2 H, J = 6.9 Hz, CH iPr), 4.13 (sept, 2 H, J = 6.9 Hz, CH iPr), 3.93 (d, 1 H, J = 2.0 Hz, CH=C), 3.04 (d, 1 H, J = 2 Hz, CH=C), 2.87 (s, 24 H, 18-c-6), 1.94 (s, 3 H, β-Me), 1.66 (d, 6 H, J = 6.9 Hz, Me iPr), 1.42 (d, 6 H, J = 6.7 Hz, Me iPr), 1.42 (d, 6 H, J = 6.7 Hz, Me iPr), 1.37 (d, 6H, J = 6.9 Hz, Me iPr).

^{13}C{^{1}H} NMR (125.6 MHz, C_6D_6, 298 K): *δ* 156.7 (C_q, *C*=CH_2), 150.4 (C_q, Ar), 150.0 (C_q, Ar), 148.0 (C_q, Ar), 147.6 (C_q, Ar), 143.0 (C_q, β-*C*-Me), 123.0 (CH, Ar), 122.9 (CH, Ar), 122.9 (CH, Ar), 122.4 (CH, Ar), 101.2 (*γ*-CH), 72.8 (*CH_2*=C), 69.8 (18-c-6), 28.1(CH, iPr), 28.0 (CH, iPr), 26.4 (Me, iPr), 26.0 (Me, iPr), 24.9 (β-Me), 24.2 (Me, iPr).

Elemental analysis found (calc.) for $C_{41}H_{64}GaKN_2O_6$: C 60.86 (62.35), H 7.97 (8.17), N 3.40 (3.55).

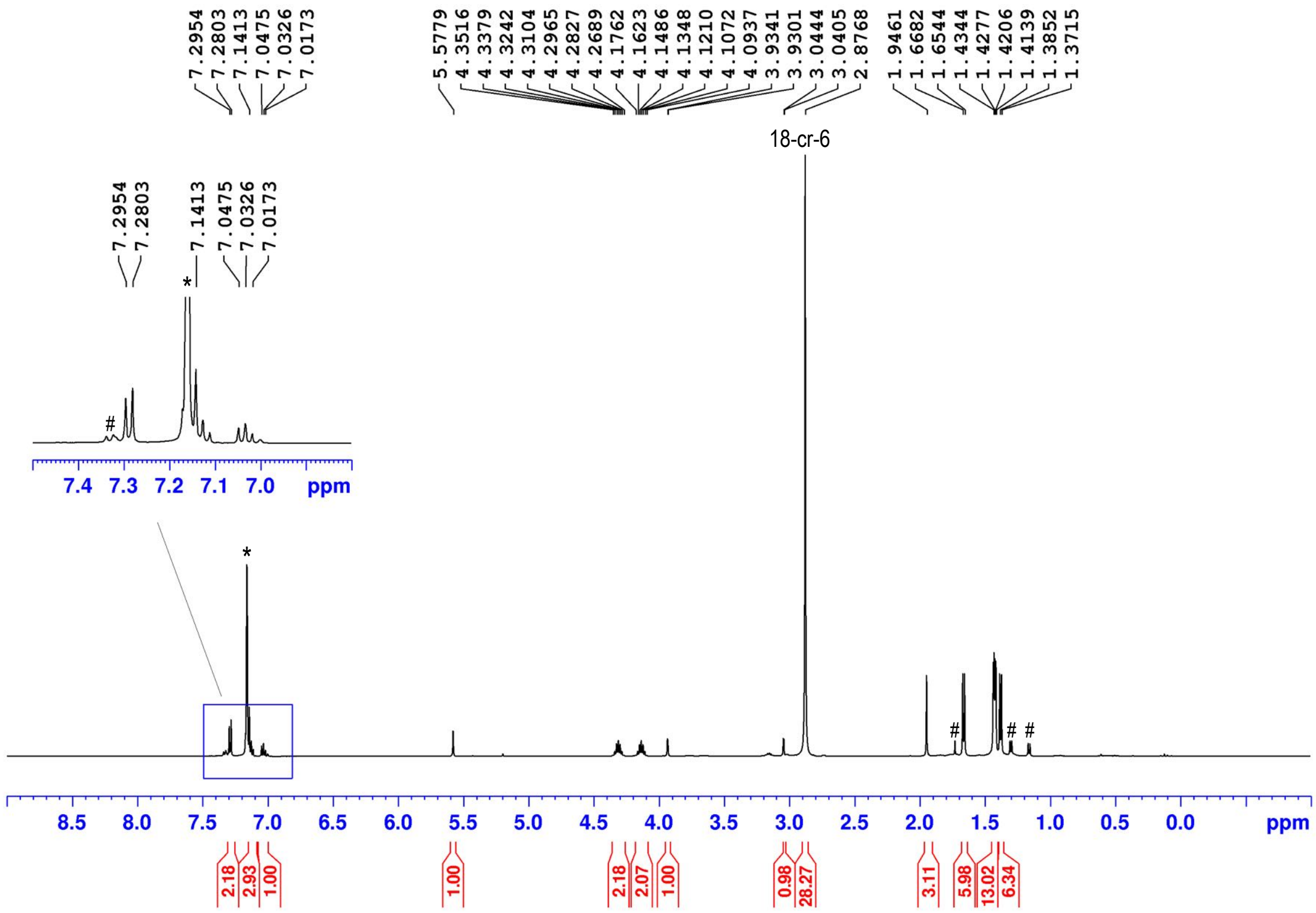

Figure S13. ^{1}H NMR (500 MHz, C_6D_6, 298K) spectrum of **2·K(18-c-6)**. The symbol (#) denotes trace amounts of the unavoidable hydrolysis product **1**.

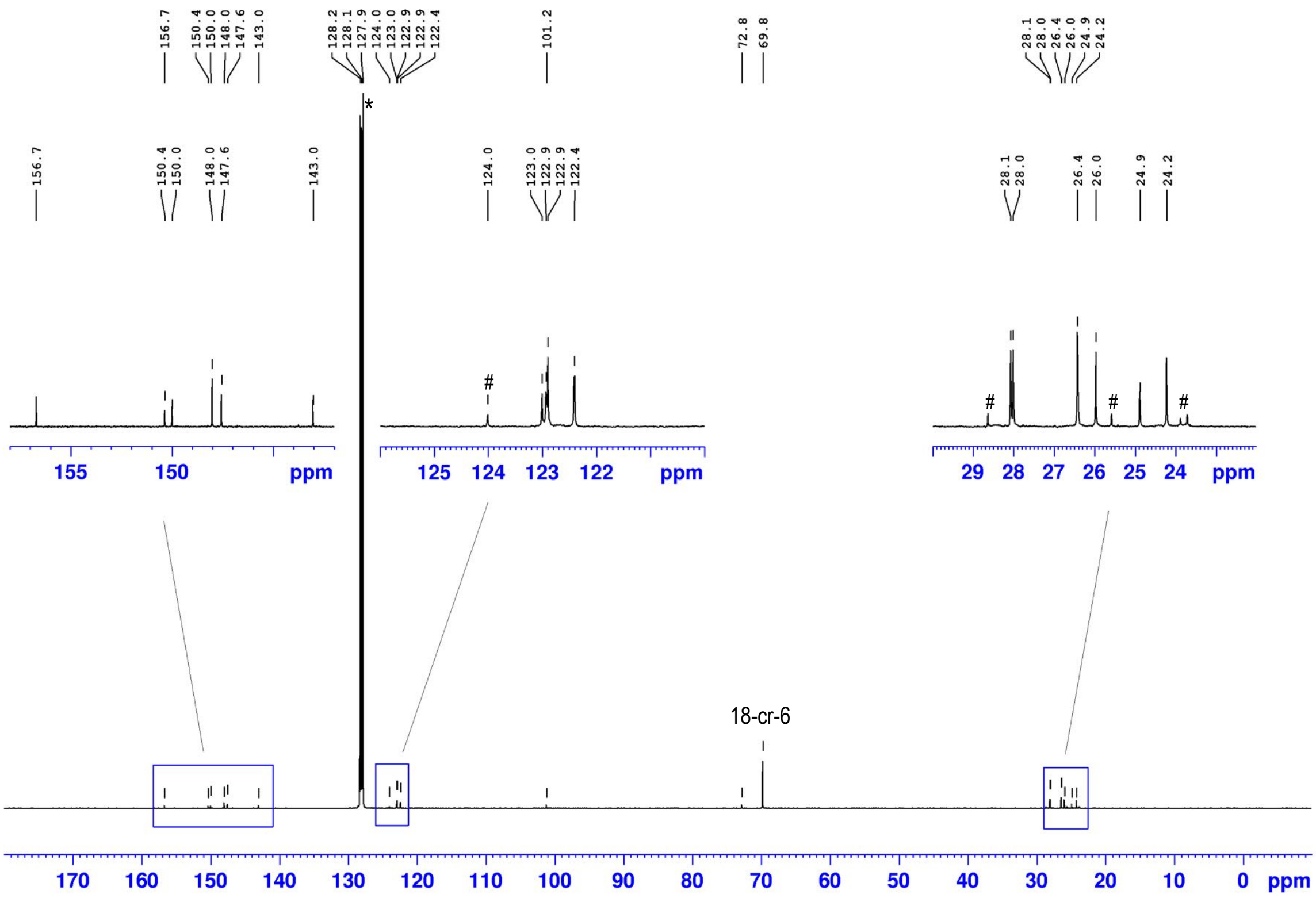

Figure S14. $^{13}C\{^{1}H\}$ NMR (125.6 MHz, C_6D_6, 298K) spectrum of **2·K(18-c-6)**. The symbol (#) denotes trace amounts of the unavoidable hydrolysis product **1**.

S2.7 Synthesis and characterization of 2·K(18-c-6)(THF)

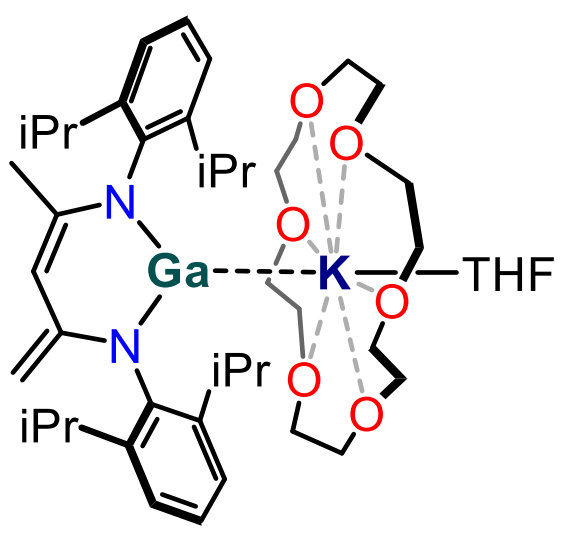

A J. Young's NMR tube was loaded with crystals of **2**·K(18-c-6) (7 mg, 8.8 µmol). Upon dissolving these crystals in a 1:1 mixture of C_6D_6/THF-*d*$_8$ (0.5 mL), a yellow solution was obtained. After standing at room temperature for 12 hours, orange prism-shaped crystals of **2·K(18-c-6)(THF)** formed in nearly quantitative yield (5 mg, 5.8 µmol, 66%), which were dried under vacuum (< 10^{-2} mbar) for 2 hours and filtered. These crystals were suitable for single-crystal X-ray diffraction.

^{1}H NMR (500 MHz, C_6D_6, 298 K): δ 7.18-7.06 (m, 6 H, Ar overlapped with solvent peaks), 5.36 (s, 1 H, γ-CH), 4.16 (sept, 2 H, J = 7.0 Hz, CH iPr), 4.04 (sept, 2 H, J = 6.9 Hz, CH iPr), 3.67 (d, 1 H, J = 2.0 Hz, CH=C), 3.04 (24 H, 18-c-6), 2.80 (d, 1 H, J = 2 Hz, CH=C), 1.82 (s, 3 H, β-Me), 1.53 (d, 6 H, J = 6.9 Hz, Me iPr), 1.33 (m, 12 H, J = 6.7 Hz, Me iPr), 1.22 (d, 6 H, J = 6.7 Hz, Me iPr).

Note on NMR data: Only ^{1}H NMR data was collected in THF-*d*$_8$ due to the limited solubility of the compound, hampering ^{13}C{^{1}H} or DOSY NMR characterization in solution.

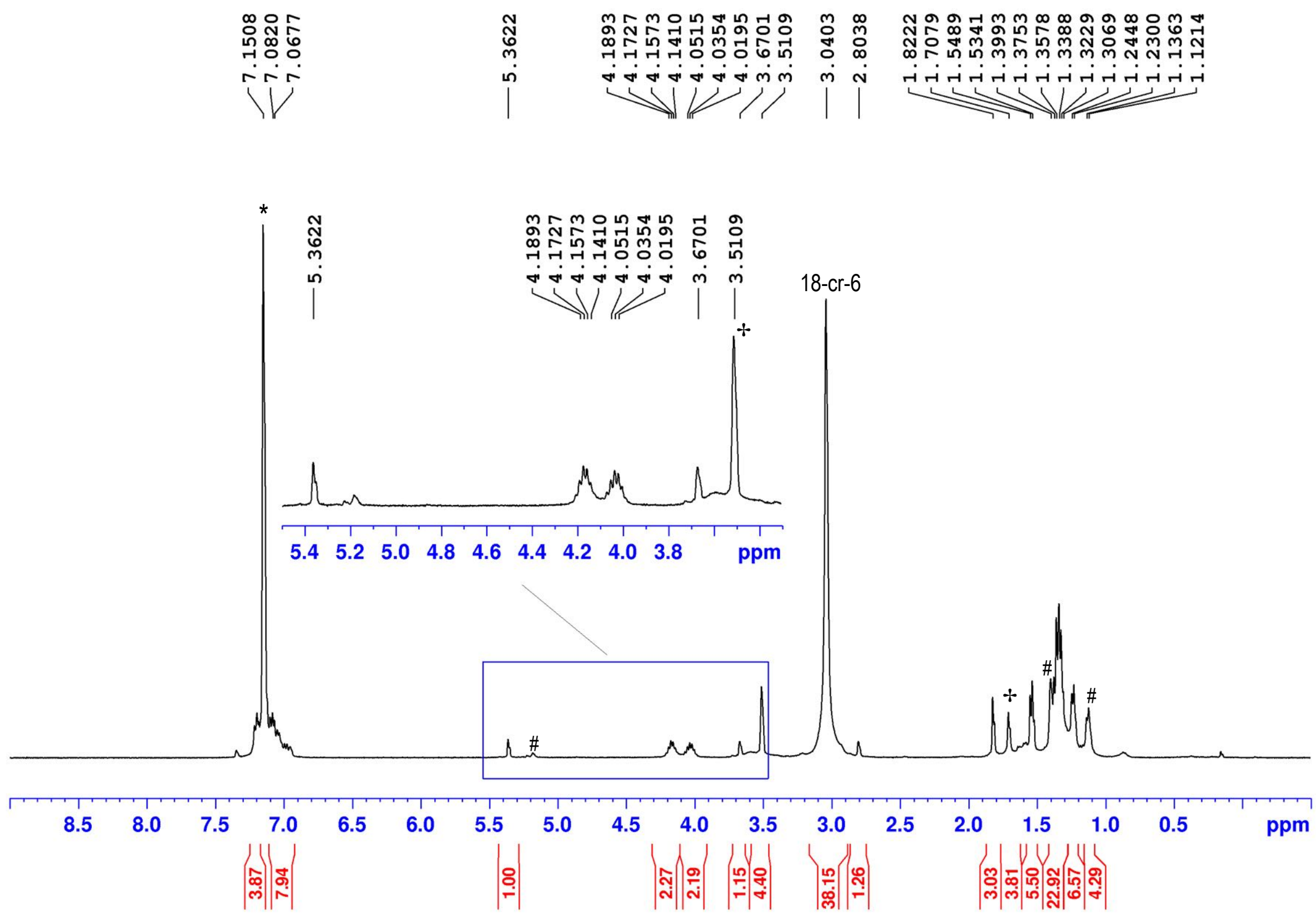

Figure S15. ^{1}H NMR (400 MHz, C_6D_6:THF-*d*$_8$, 298 K) spectrum of **2·K(18-c-6)(THF)**. The symbol (#) denotes trace amounts of the unavoidable hydrolysis product **1**.

S2.8 Synthesis and characterization of 2·Cs(18-c-6)

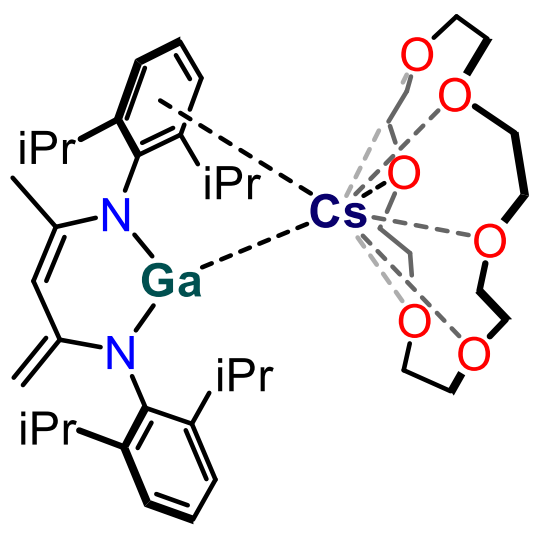

In a J. Young's ampoule, **1** (156 mg, 0.320 mmol), CsHMDS (116 mg, 0.395 mmol), and 18-crown-6 (85 mg, 0.322 mmol) were combined and dissolved in benzene (1.5 mL). The mixture was stirred at room temperature for 5 hours. The resulting yellow solution was then filtered via cannula and layered with *n*-hexane (5 mL). After two days, orange prism-shaped crystals formed. These crystals were dried under vacuum (< 10^{-2} mbar) for 1 hour, affording **2·Cs(18-c-6)** (200 mg, 0.226 mmol, 71%). These crystals were suitable for single-crystal X-ray diffraction.

^{1}H NMR (400 MHz, C_6D_6, 298 K): δ 7.32 (d, 2 H, J = 7.5 Hz, Ar), 7.26 (d, 2 H, J = 7.5 Hz; Ar), 7.12 (m overlapped with solvent peak, 2 H, Ar), 5.51 (s, 1 H, γ-CH), 4.37 (sept, 2 H, J = 6.9 Hz, CH iPr), 4.21 (sept, 2 H, J = 6.9 Hz, CH iPr), 3.81 (d, 1 H, J = 2.0 Hz, CH=C), 2.98 (s, 24 H, 18-c-6), 2.94 (d, 1 H, J = 2.0 Hz, CH=C), 1.95 (s, 3 H, β-Me), 1.64 (d, 6 H, J = 6.9 Hz, Me iPr), 1.53 (d, 12 H, J = 6.8 Hz, Me iPr), 1.44 (d, 6 H, J = 6.9 Hz, Me iPr).

^{13}C{^{1}H} NMR (100.5 MHz, C_6D_6, 298 K): δ 156.9 (C_q, *C*=CH_2), 151.8 (C_q, Ar), 149.6 (C_q, Ar), 148.7 (C_q, Ar),147.3 (C_q, Ar), 143.6 (β-*C*-Me), 123.6 (CH, Ar), 123.1 (CH, Ar), 122.6 (CH, Ar), 100.9 (*γ*-CH), 71.7 (*CH_2*=C), 69.8 (18-c-6), 28.2 (CH, iPr), 28.1 (CH, iPr), 26.6 (Me, iPr), 26.4 (Me, iPr), 25.8 (Me, iPr), 24.9 (β-Me), 24.1 (Me, iPr).

^{133}Cs{^{1}H} NMR (52.4 MHz, C_6D_6, 298 K): δ 31.1 (br s, Δν = 80 Hz).

Elemental analysis found (calc.) for $C_{41}H_{64}GaCsN_2O_6$: C 53.96 (55.73), H 7.51 (7.30), N 2.12 (3.17).

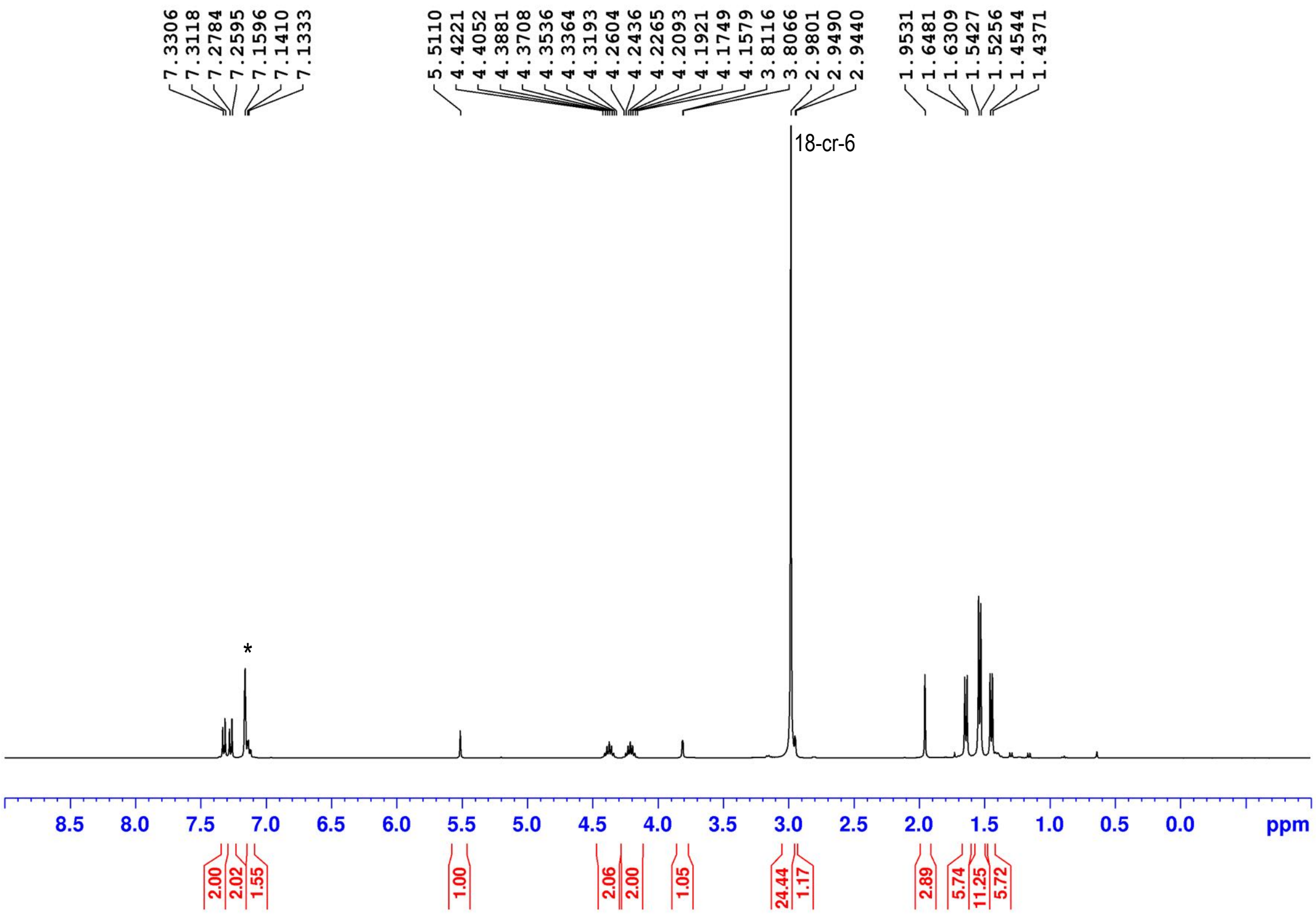

Figure S16. ^{1}H NMR (400 MHz, C_6D_6, 298K) spectrum of **2·Cs(18-c-6)**. The symbol (*) denotes the C_6D_6 residual solvent peak.

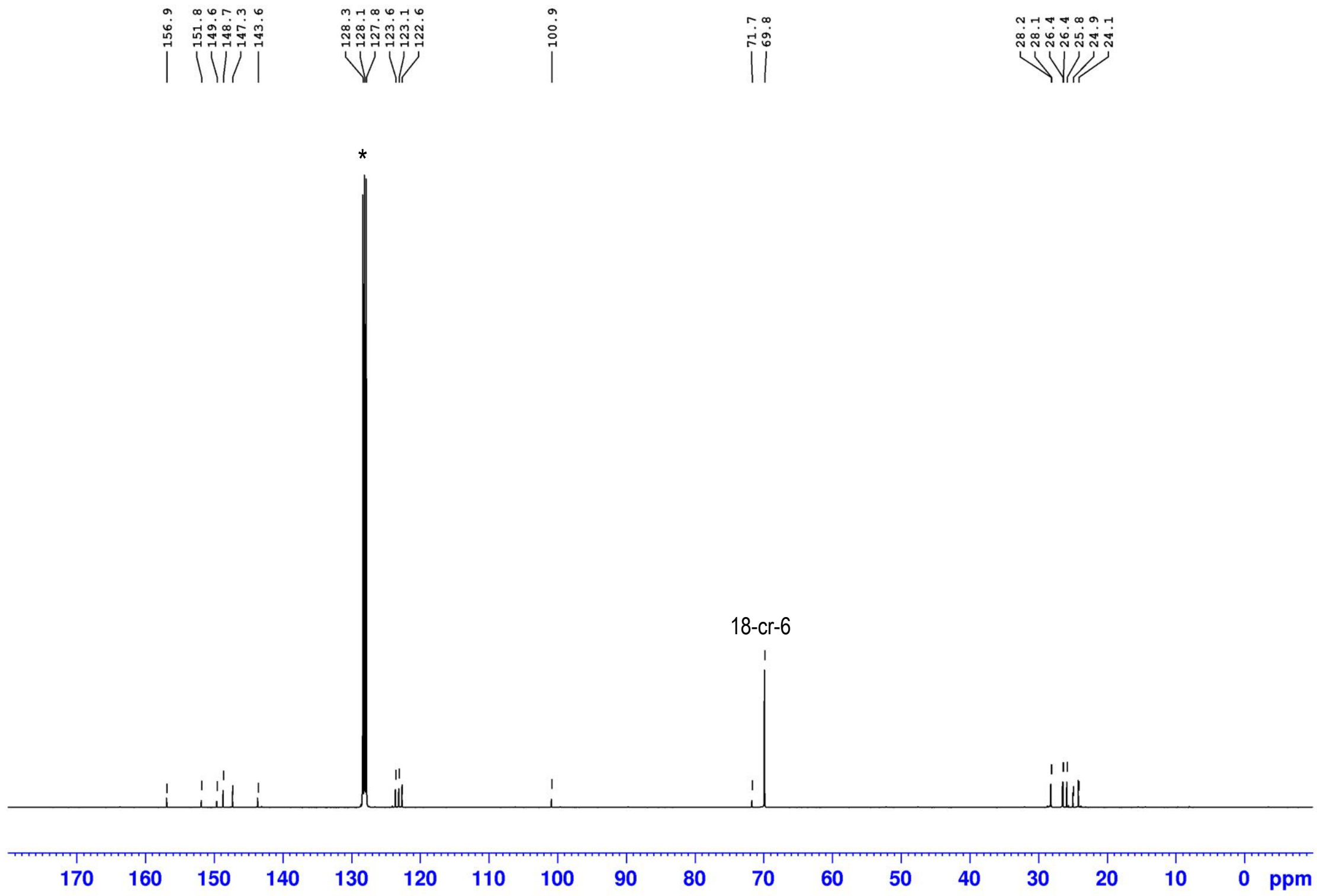

Figure S17. $^{13}C\{^{1}H\}$ NMR (100.6 MHz, C_6D_6, 298 K) spectrum of **2·Cs(18-c-6)**. The symbol (*) denotes the C_6D_6 residual solvent peak.

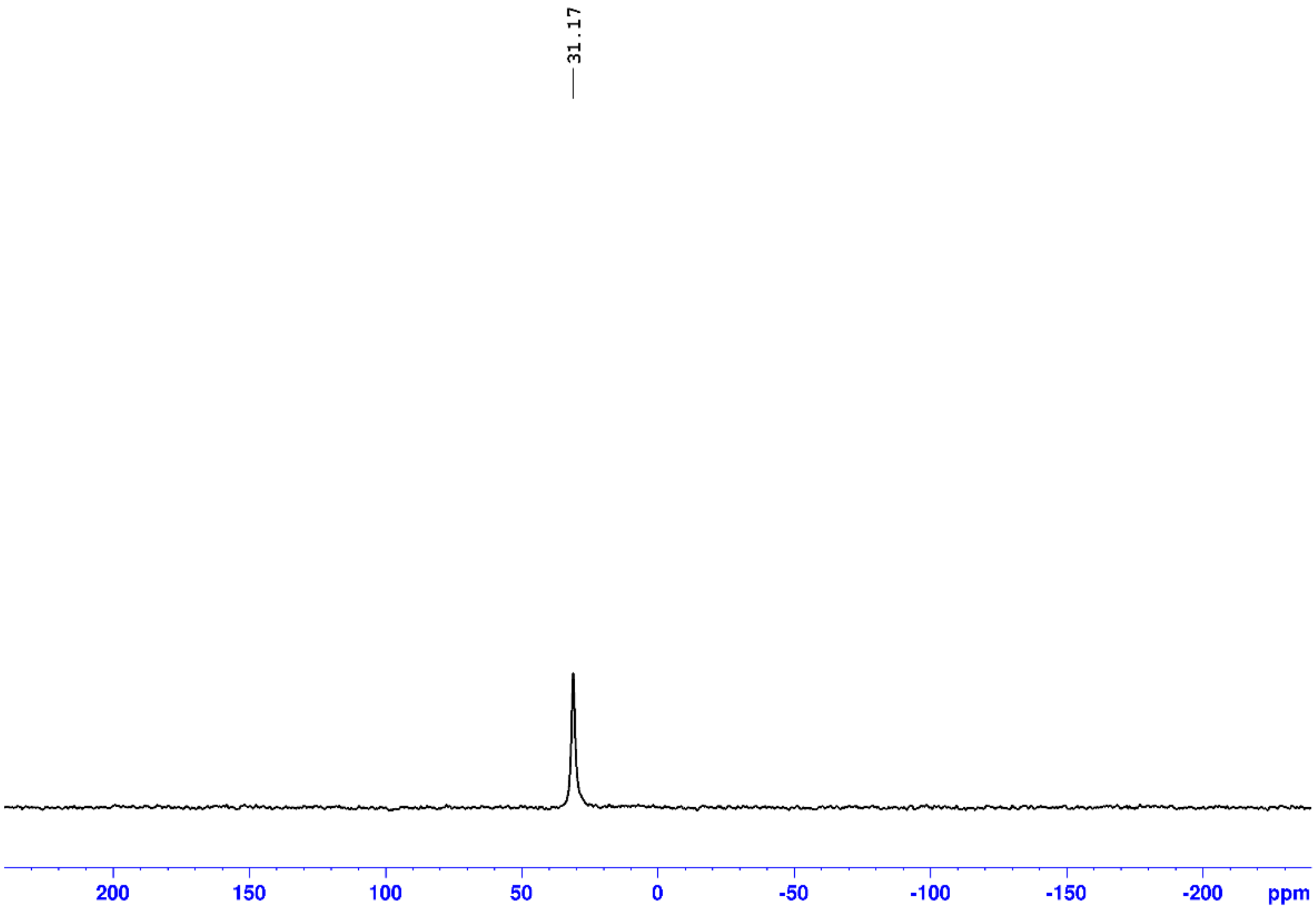

Figure S18. ^{133}Cs NMR (52.4 MHz, C_6D_6, 298K) spectrum of **2·Cs(18-c-6)**.

S2.9 Synthesis and characterization of 2·Cs(18-c-6)$_2$

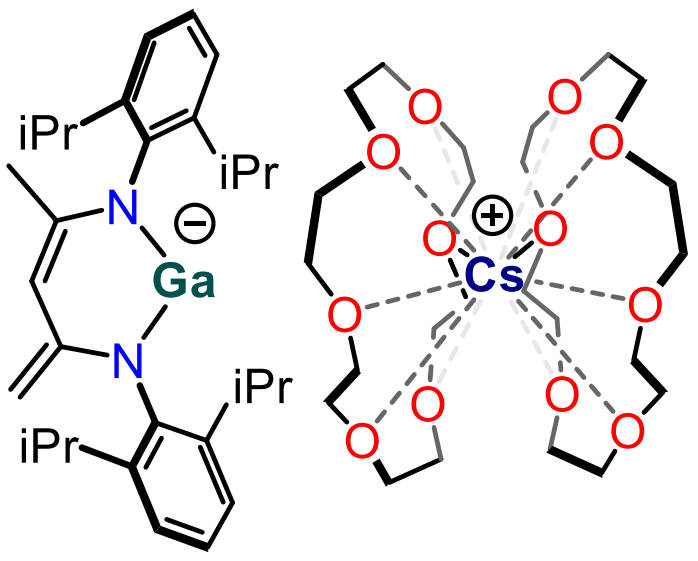

In a J. Young's ampoule, **1** (100 mg, 0.205 mmol), CsHMDS (66 mg, 0.225 mmol), and 18-crown-6 (108 mg, 0.410 mmol) were weighed and dissolved in benzene (1.5 mL). The solution was mixed and allowed to stir at room temperature for a duration of 5 hours. Subsequently, the resulting yellow suspension was processed through cannula filtration. The residual solid was then thoroughly washed with *n*-hexane (2 x 3 mL) and subjected to drying under vacuum (< 10^{-2} mbar) for a period of 2 hours, resulting with **2·Cs(18-c-6)$_2$** as a yellow solid (176 mg, 0.148 mmol, 72% yield). Crystals suitable for single-crystal X-ray diffraction were obtained by layering a solution of **2·Cs(18-c-6)$_2$** in benzene with *n*-hexane.

^{1}H NMR (400 MHz, Tol-*d*$_8$, 298 K): *δ* 7.26 (d, 2 H, J = 7.5 Hz, Ar), 7.20 (d, 2 H, J = 7.5 Hz; Ar), 7.08 (m overlapped with solvent peak, 2 H, Ar), 5.31 (s, 1 H, γ-CH), 4.25 (sept, 2 H, J = 6.7 Hz, CH iPr), 4.13 (sept, 2 H, J = 6.8 Hz, CH iPr), 3.59 (s, 1 H, CH=C), 3.26 (s, 48 H, 18-c-6), 2.75 (s, 1 H, CH=C), 1.86 (s, 3 H, β-Me), 1.56 (d, 6 H, J = 6.9 Hz, Me iPr), 1.47 (m, 12 H, Me iPr), 1.41 (d, 6 H, Me iPr).

^{13}C{^{1}H} NMR (125.6 MHz, Tol-*d*$_8$, 298 K): *δ* 156.7 (C_q, *C*=CH_2), 151.7 (C_q, Ar), 149.7 (C_q, Ar), 148.5 (C_q, Ar),147.2 (C_q, Ar), 143.2 (β-*C*-Me), 123.3 (CH, Ar), 123.3 (CH, Ar), 122.9 (CH, Ar), 122.5 (CH, Ar), 101.0 (*γ*-CH), 71.7 (*CH_2*=C), 70.4 (18-c-6), 28.1 (CH, iPr), 28.1 (CH, iPr), 26.5 (Me, iPr), 26.4 (Me, iPr), 25.8 (Me, iPr), 24.9 (β-Me), 24.1 (Me, iPr).

^{133}Cs{^{1}H} NMR (52.4 MHz, Tol-*d*$_8$, 298 K): δ 15.6 (br s, Δν = 270 Hz).

Elemental analysis found (calc.) for $C_{53}H_{88}CsGaN_2O_{12}$: C 54.64 (55.46), H 7.19 (7.73), N 2.21 (2.44).

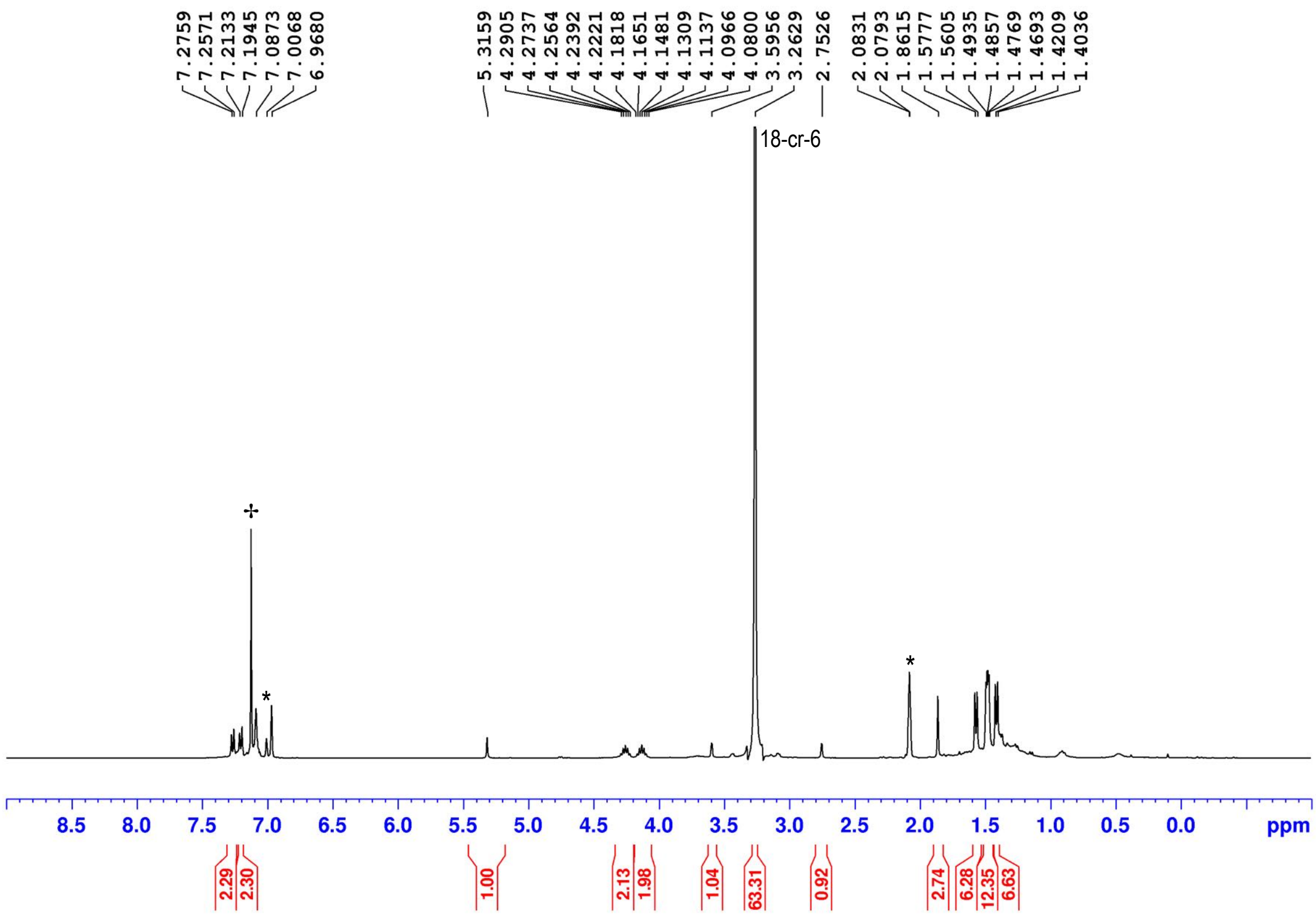

Figure S19. ^{1}H NMR (400 MHz, Tol-d_8, 298 K) spectrum of **2·Cs(18-c-6)$_2$**. The symbols (*) and (+) denote residual Tol-d_8 and occluded C_6H_6 solvent peaks, respectively.

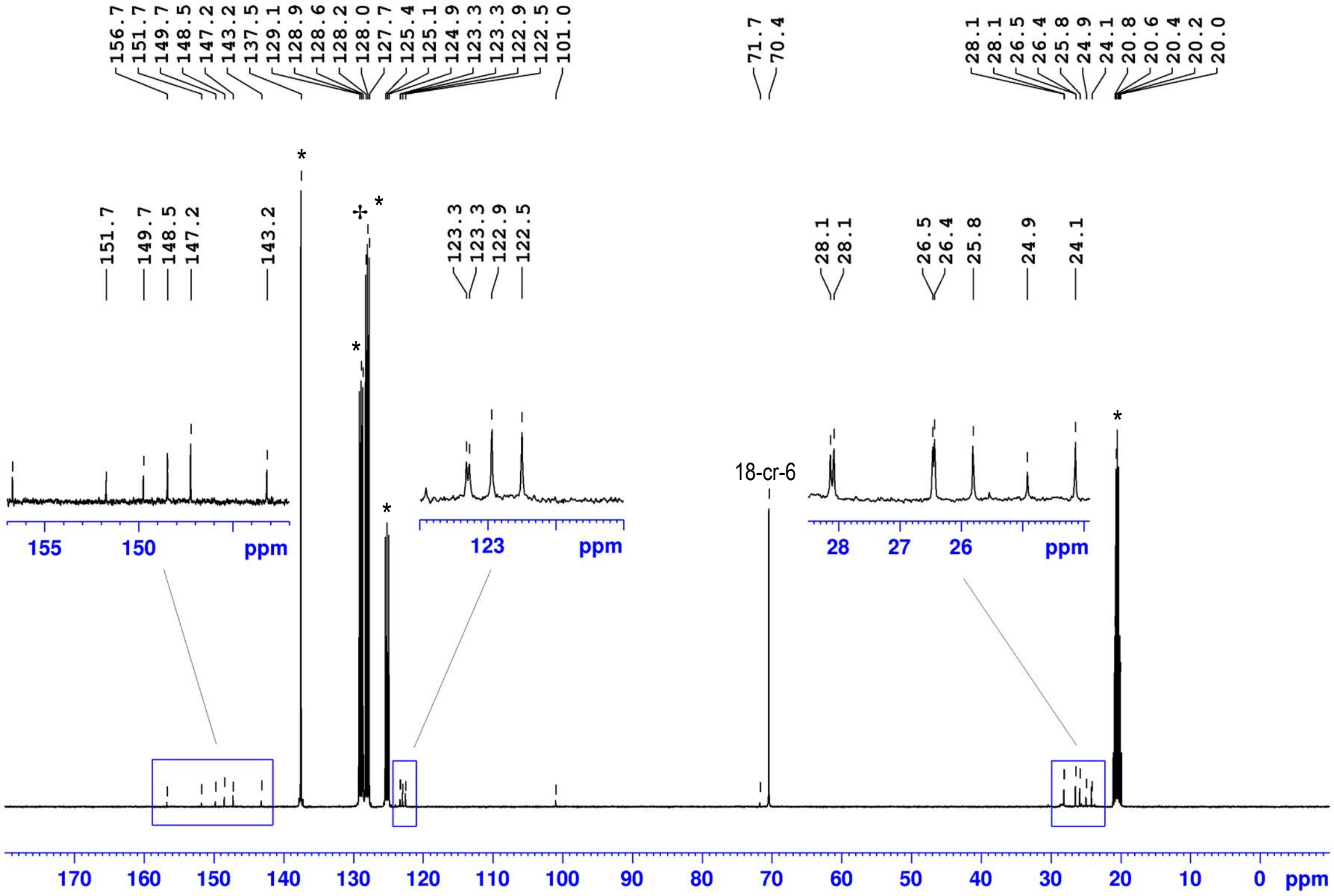

Figure S20. ^{13}C{^{1}H} NMR (100.6 MHz, Tol-d_8, 298 K) spectrum of **2·Cs(18-c-6)$_2$**. The symbols (*) and (+) denote residual Tol-d_8 and occluded C_6H_6 solvent peaks, respectively.

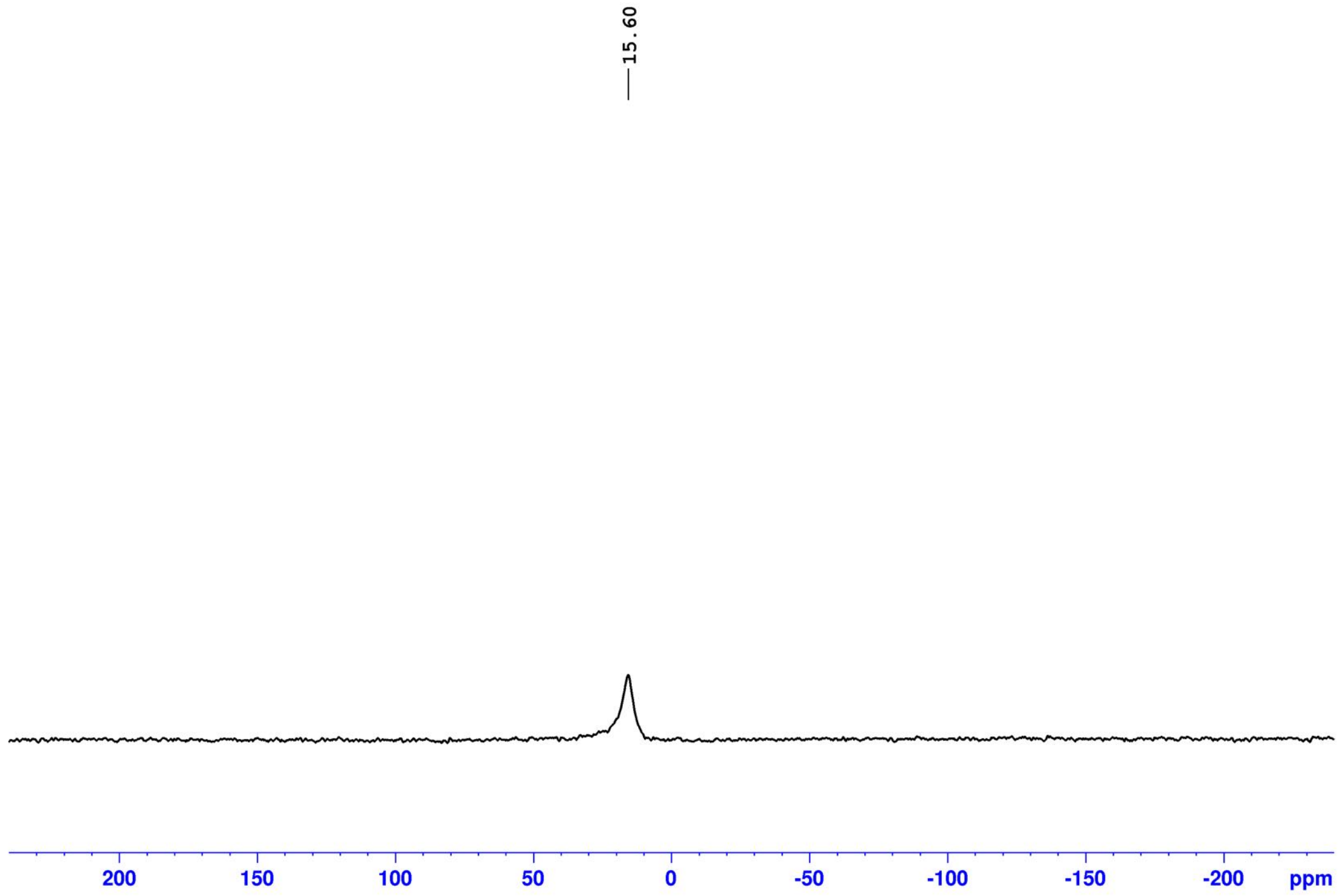

Figure S21. ^{133}Cs NMR (52.4 MHz, Tol-d_8, 298K) spectrum of **2·Cs(18-c-6)$_2$**.

S2.10 Synthesis and characterization of [(2·K)$_2$(KHMDS)$_2$]$_\infty$

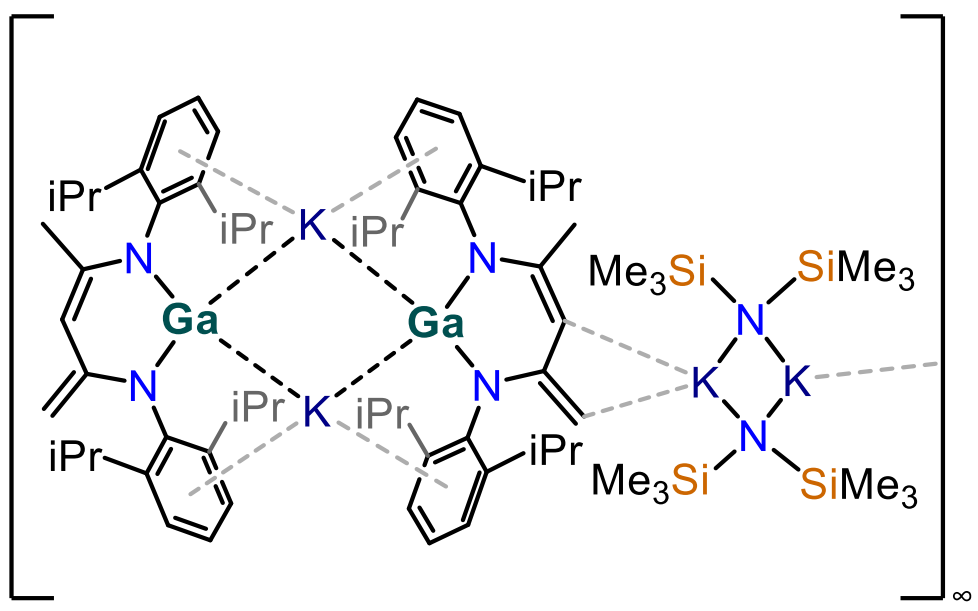

A J. Young's ampoule was charged with **1** (212 mg, 0.435 mmol) and KHMDS (198 mg, 0.992 mmol). Benzene (3 mL) was then added, and the mixture was stirred at 50ºC for 2 days. The resulting brown-green suspension was filtered via a cannula. The solid residue was washed with *n*-hexane (2 x 5 mL) and dried under high vacuum (< 10^{-2} mbar) for 2 hours, yielding [**(2·K)$_2$(KHMDS)$_2$**]$_\infty$ as a dark yellow solid (50 mg, 0.034 mmol, 32%). Orange block-shaped crystals of [**(2·K)$_2$(KHMDS)$_2$**]$_\infty$ suitable for single-crystal X-ray diffraction were obtained by placing the reaction mixture in a hot water bath at 50ºC and allowing it to stand for 2 days.

Note: Reducing the amount of KHMDS to 1 equiv produced [**(2·K)$_2$(KHMDS)$_2$**]$_\infty$ with a comparable isolated yield. The NMR spectroscopic data of [**(2·K)$_2$(KHMDS)$_2$**]$_\infty$ in solution, obtained using ^{1}H, ^{13}C{^{1}H} and DOSY, is consistent (identical), regardless of its physical form (dark yellow powder or orange block-shaped crystals of [**(2·K)$_2$(KHMDS)$_2$**]$_\infty$.

^{1}H NMR (400 MHz, 4:1 C_6D_6:THF-*d*$_8$, 298 K): δ 7.17-7.11 (m overlapped with residual solvent peak from C_6D_6, 4 H, Ar), 7.00 (m, 2 H, Ar), 5.18 (s, 1 H, γ-CH), 4.05 (sept, 2 H, J = 6.9 Hz, CH iPr), 3.96 (sept, 2 H, J = 6.9 Hz, CH iPr), 3.44 (s, 1 H, CH=C), 2.60 (s, 1 H, CH=C), 1.70 (s overlapped with residual solvent peak from THF-d_8, 3 H. β-Me), 1.42 (m, 6 H, Me iPr), 1.31 (m, 18 H, Me iPr), 0.22 (s, 18 H, $SiMe_3$).

^{13}C{^{1}H} NMR (125.6 MHz, 4:1 C_6D_6:THF-*d*$_8$, 298 K): *δ* 156.7 (C_q, *C*=CH_2), 150.7 (C_q, Ar), 148.9 (C_q, Ar), 147.3 (C_q, β-*C*-Me), 147.2 (C_q, Ar), 123.6 (CH, Ar), 123.2 (CH, Ar), 123.0 (CH, Ar), 122.6 (CH, Ar), 100.4 (*γ*-CH), 71.4 (*CH_2*=C), 28.0 (CH, iPr), 27.8 (CH, iPr), 26.2 (Me, iPr), 26.5 (Me, iPr), 25.4 (Me, ^{i}Pr), 24.1 (Me, ^{i}Pr), 23.8 (β-Me), 7.0 (Me, $SiMe_3$).

Elemental analysis found (calc.) for $C_{70}H_{116}Ga_2K_4N_6Si_4$: C 56.48 (57.99), H 7.50 (8.06), N 5.33 (5.80).

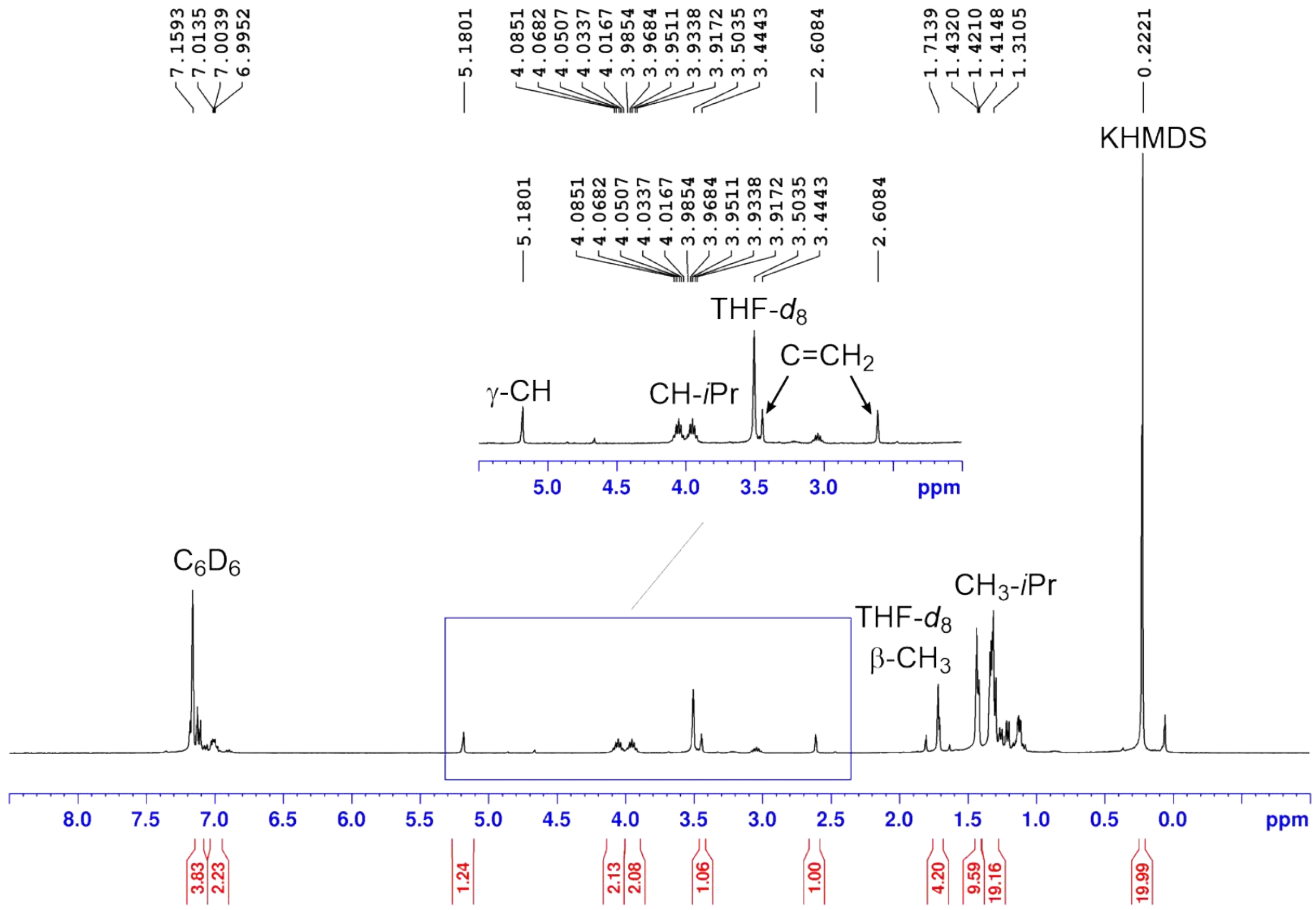

Figure S22. ^{1}H NMR (400 MHz, C_6D_6:THF-d_8, 298 K) of [**(2·K)$_2$(KHMDS)$_2$**]$_\infty$.

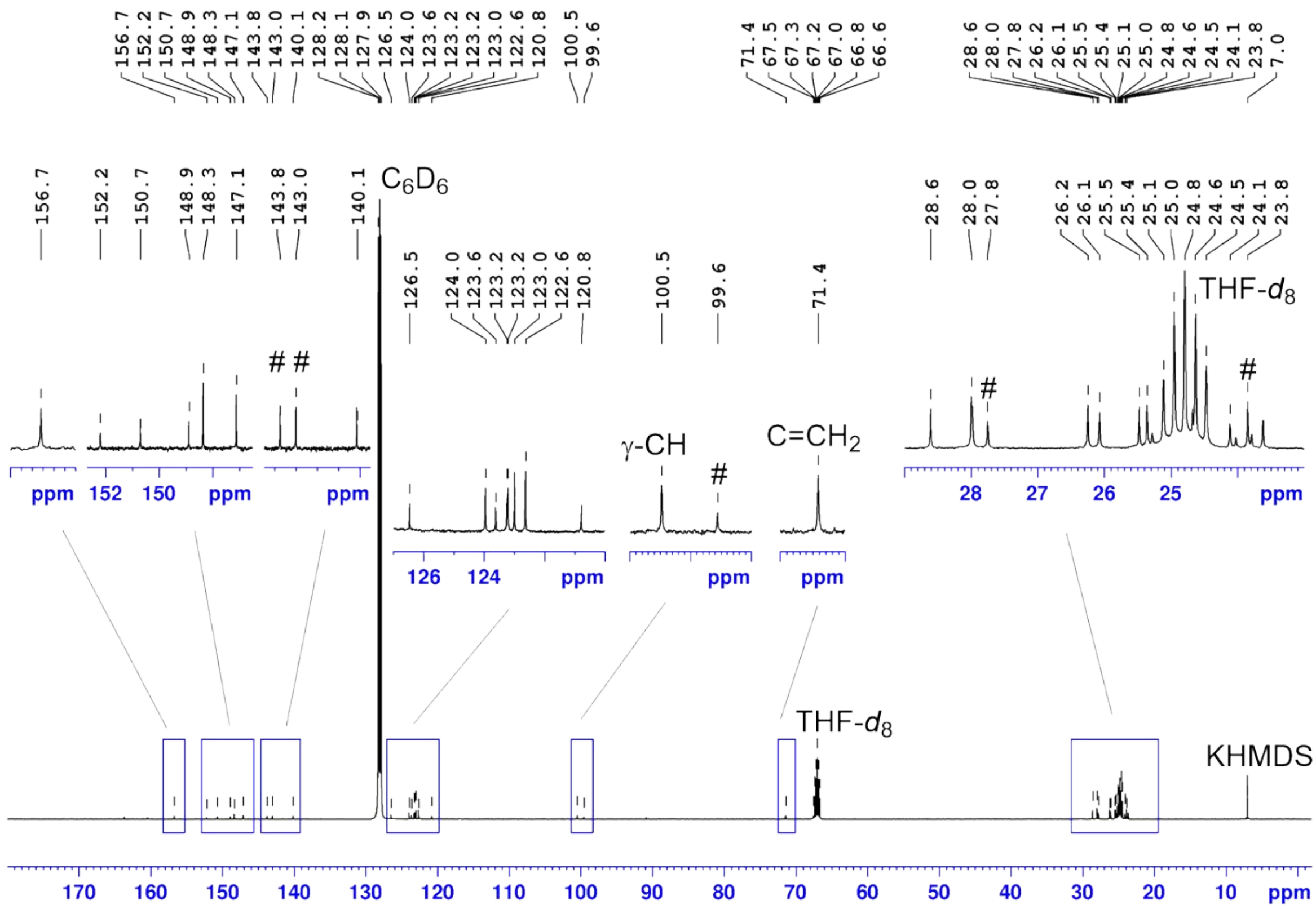

Figure S23. ^{13}C{^{1}H} NMR (125.6 MHz, C_6D_6:THF-d_8, 298 K) spectrum of [**(2·K)$_2$(KHMDS)$_2$**]$_\infty$. The symbol (#) represents traces of **1** resulting from unavoidable hydrolysis in solution.

S2.11 Synthesis and characterization of [(**2·Cs**)$_2$(**CsHMDS**)$_2$]$_\infty$

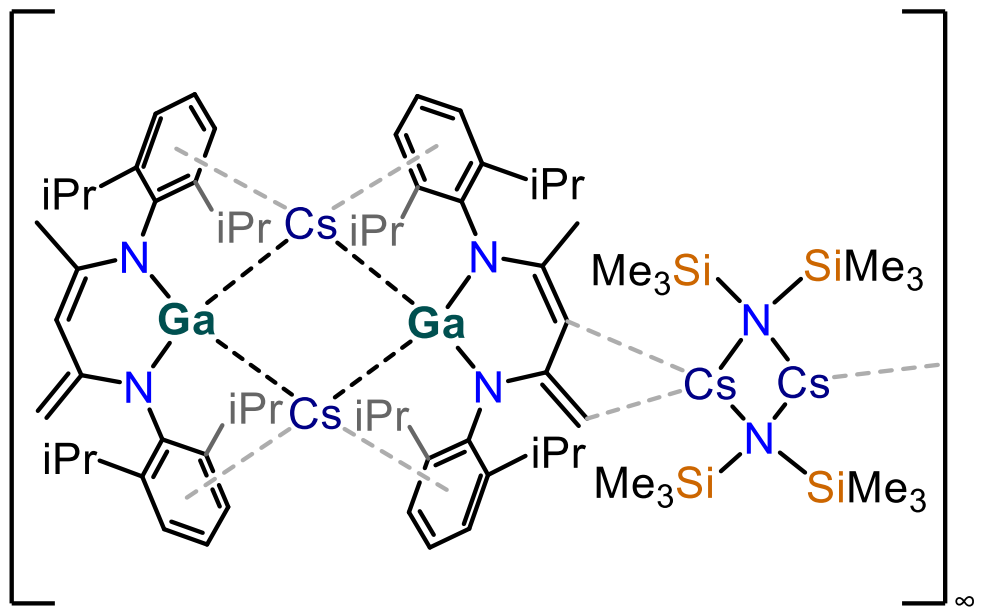

In a J. Young's ampoule, **1** (225 mg, 0.462 mmol) and CsHMDS (272 mg, 0.924mmol) were combined. Toluene (4 mL) was added, and the mixture was stirred at room temperature until a homogeneous solution formed (approximately 20 minutes). The yellow solution was then heated and maintained at 45ºC for 16 hours. Subsequent cooling resulted in a brown-green suspension, which was filtered via cannula. The solid was washed with *n*-hexane (2 x 3 mL) and dried under vacuum (< 10^{-2} mbar) for 2 hours, affording [(**2·Cs**)$_2$(**CsHMDS**)$_2$]$_\infty$ as a yellow solid (80 mg, 0.088 mmol, 20%). This protocol was replicated, with the reaction mixture heated in a water bath at 45ºC, then allowed to cool gradually to room temperature over 2 days, giving crystals suitable for single-crystal X-ray diffraction.

Note: Reducing the amount of CsHMDS to 1 equiv produced [(**2·Cs**)$_2$(**CsHMDS**)$_2$]$_\infty$ with a comparable isolated yield.

^{1}H NMR (400 MHz, 4:1 C_6D_6:THF-*d*$_8$, 298 K): δ 7.13 (d, 2 H, J = 7.6 Hz, Ar), 7.07 (d, 2 H, J = 7.6 Hz, Ar), 6.97 (m, 2 H, Ar), 5.08 (s, 1 H, γ-CH), 3.98 (sept, 2 H, J = 6.9 Hz, CH iPr), 3.87 (sept, 2 H, J = 6.9 Hz, CH iPr), 3.35 (d, 1 H, J = 1.8 Hz, CH=C), 2.51 (d, 1 H, J = 1.8 Hz, CH=C), 1.65 (s, 3 H. β-Me), 1.36 (d, 3 H, J = 6.9 Hz, CH_3 iPr), 1.31 (dd, 12 H, J_1 = 7.2 Hz, J_1 = 1.2 Hz, CH_3 iPr), 1.25 (d, 6 H, J = 6.9 Hz, CH_3 iPr), 0.09 (s, 18 H, $SiMe_3$).

^{13}C{^{1}H} NMR (100.6 MHz, 4:1 C_6D_6:THF-*d*$_8$, 298 K): *δ* 156.3 (C_q, *C*=CH_2), 151.5 (C_q, Ar), 149.8 (C_q, Ar), 148.7 (C_q, Ar),147.5 (C_q, Ar), 143.2 (β-*C*-Me), 123.7 (CH, Ar), 123.7 (CH, Ar), 123.5 (CH, Ar),122.9 (CH, Ar), 100.7 (*γ*-CH), 72.0 (*CH$_2$*=C), 28.0 (CH, iPr), 28.0 (CH, iPr), 26.1 (Me, iPr), 25.9 (Me, iPr), 25.2 (Me, iPr), 23.8 (β-Me), 6.9 ($SiMe_3$).

^{133}Cs{^{1}H} NMR (52.4 MHz, 4:1 C_6D_6:THF-*d*$_8$, 298 K): *δ* 60.9 (br s, $\Delta\nu$ = 240 Hz).

Elemental analysis found (calc.) for $C_{70}H_{116}Ga_2Cs_4N_6Si_4$: C 42.28 (46.07), H 5.47 (6.41), N 3.83 (4.60).

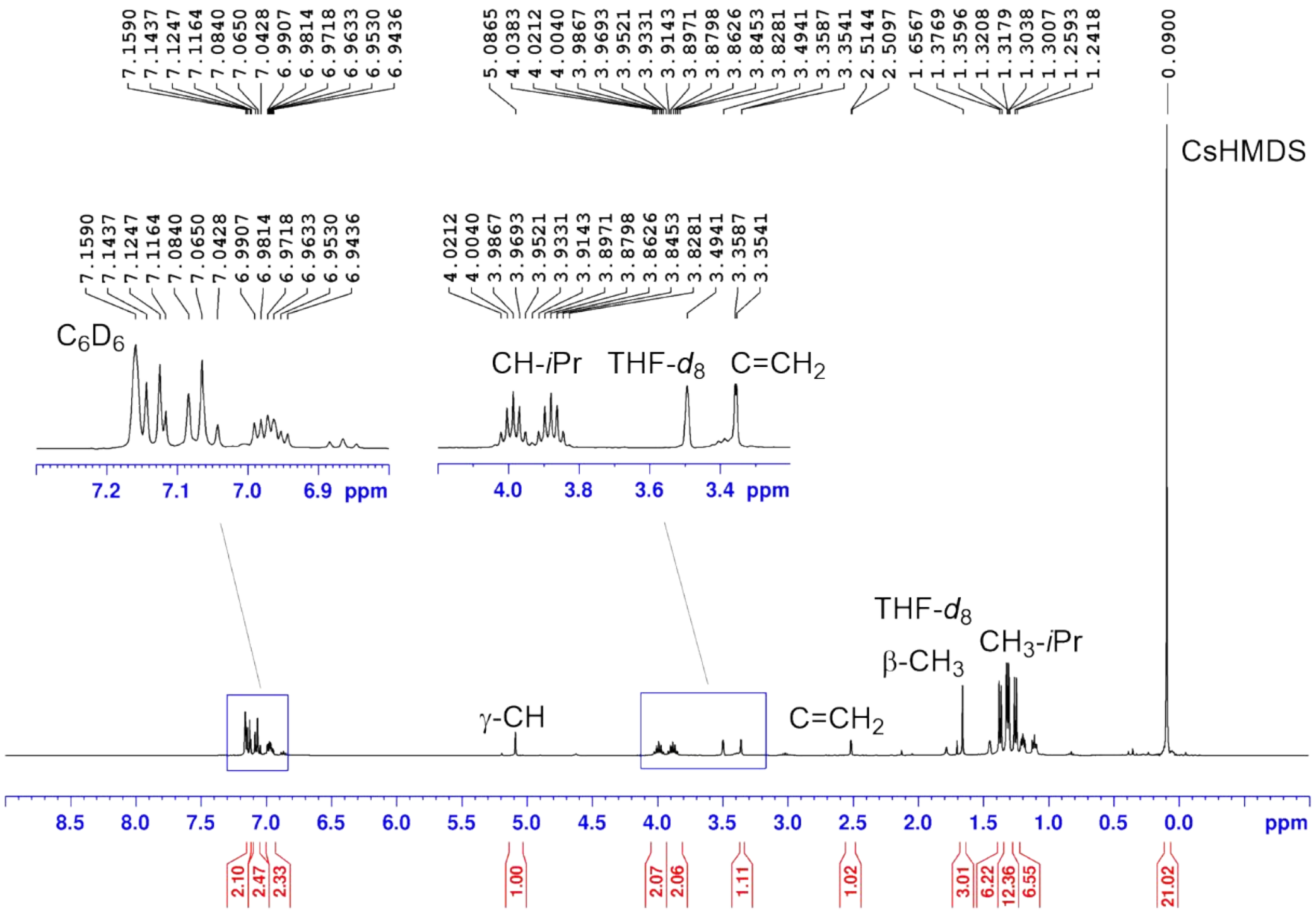

Figure S24. ^{1}H NMR (400 MHz, C_6D_6:THF-d_8, 298 K) of [(**2·Cs**)$_2$(**CsHMDS**)$_2$]$_\infty$.

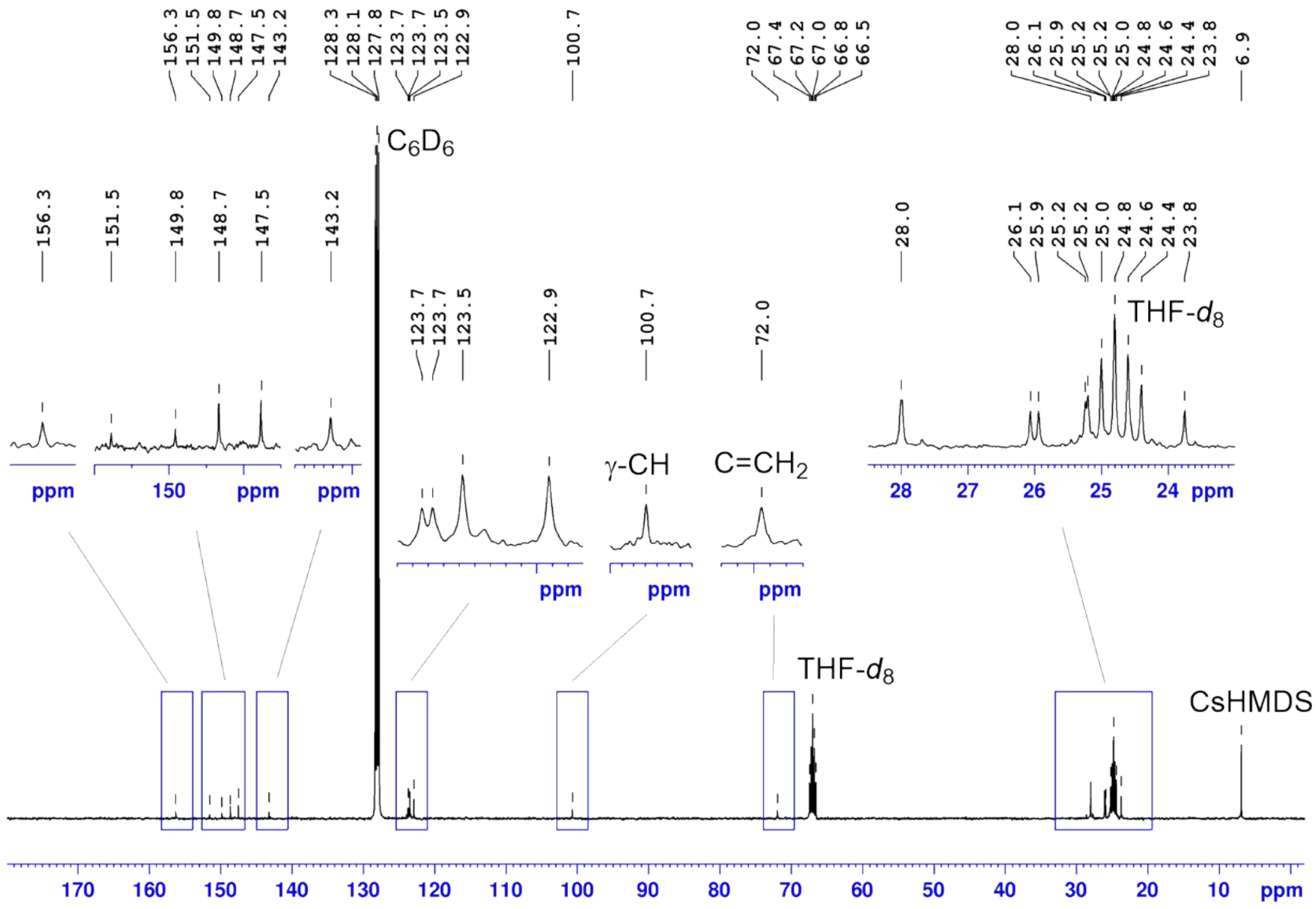

Figure S25. $^{13}C\{^{1}H\}$ NMR (125.6 MHz, C_6D_6:THF-d_8, 298 K) spectrum of [(**2·Cs**)$_2$(**CsHMDS**)$_2$]$_\infty$.

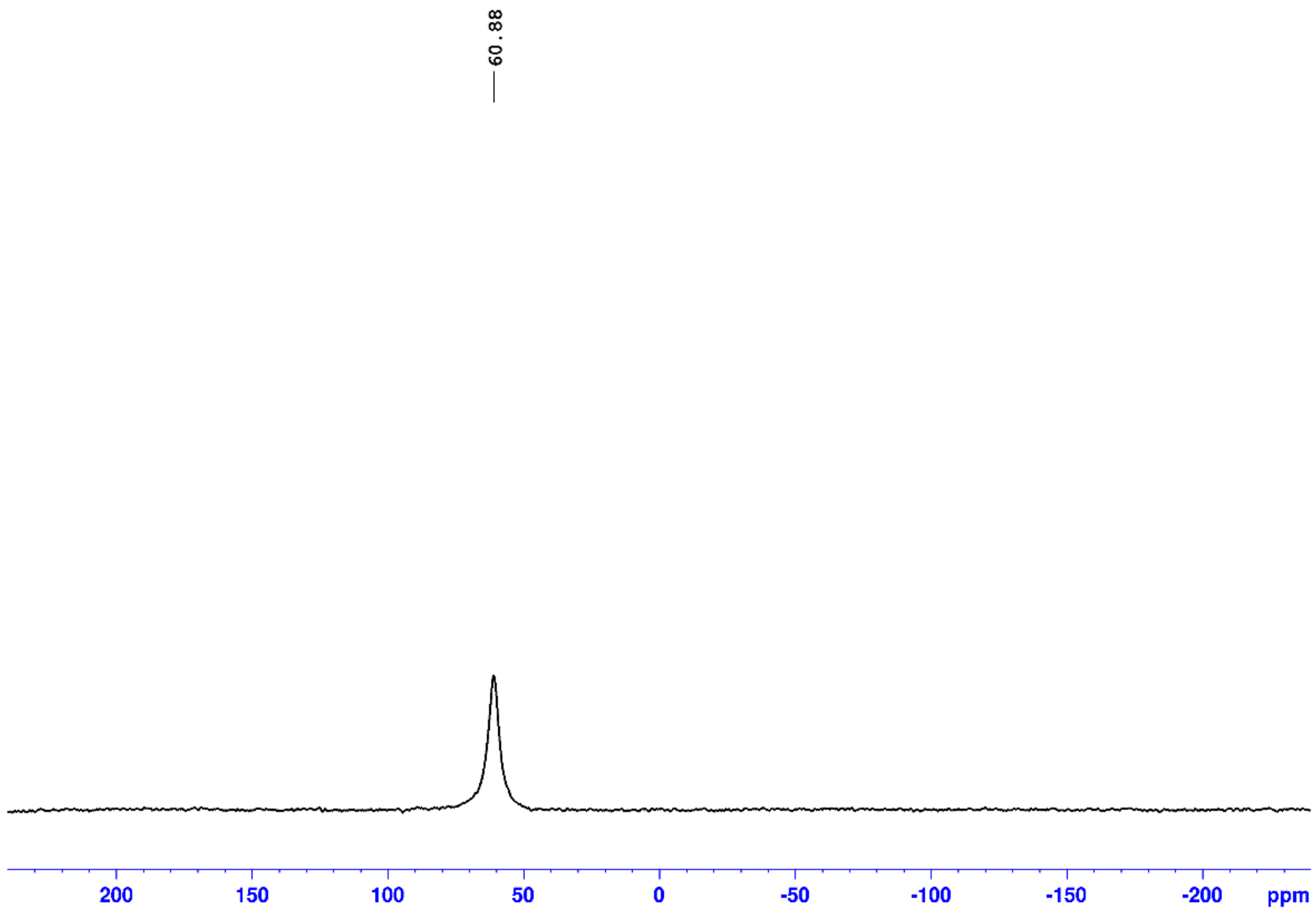

Figure S26. [133]Cs NMR (52.4 MHz, C_6D_6:THF-d_8, 298 K) spectrum of [(**2·Cs**)$_2$(**CsHMDS**)$_2$]$_\infty$.

S3 Nucleophilic Substitution Reactivity

S3.1 Synthesis and characterization of 3

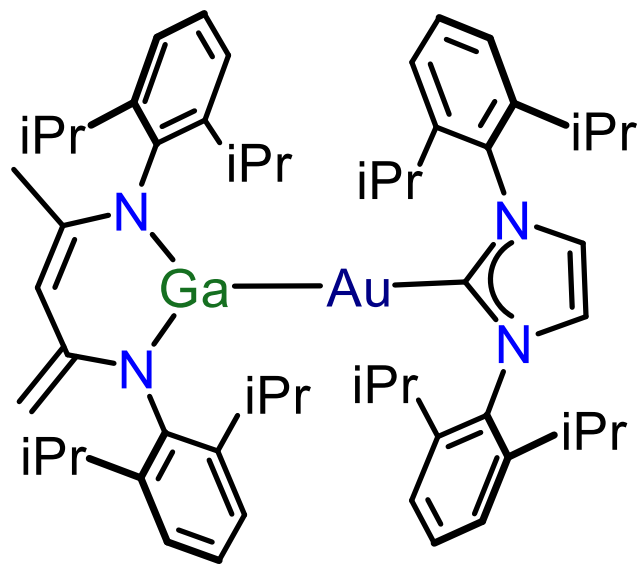

In a glovebox, a J. Young ampoule was charged with **2·Cs(18-c-6)$_2$** (173 mg, 0.150 mmol), (IPr)AuCl (93.2 mg, 0.150 mmol) and toluene (2 mL). The reaction mixture was stirred at room temperature for 1 h to give a yellow suspension. The suspension was filtered, concentrated (1 mL), then THF (0.1 mL) and *n*-hexane (2.5 mL) were added. Slow evaporation at room temperature afforded **3** as yellow prism-like crystals (85 mg, 0.079 mmol, 53%) suitable for single-crystal X-ray diffraction (Figure S74). An analogous crystallization carried out from benzene rather than toluene under otherwise identical conditions gave a second independent crystal form of **3** in comparable yield, which was also suitable for single-crystal X-ray diffraction (Figure S75). Neither crystal form contains coordinated THF.

Formation of **3** was additionally monitored by in situ ^{1}H NMR spectroscopy, which showed complete consumption of **2·Cs(18-c-6)$_2$** and (IPr)AuCl and clean formation of **3** within 2 min in either Tol-d_8 or C_6D_6 at room temperature. The reaction is accompanied by precipitation of an insoluble caesium chloride/crown ether salt and release of free 18-crown-6 in solution.

Identification of the precipitated byproduct as (18-c-6)CsCl: The insoluble precipitate formed during generation of **3** was collected, washed with toluene, and dissolved in D_2O for NMR analysis. It showed a single ^{133}Cs NMR resonance (δ 10.5 ppm, br s, 52.4 MHz) and a ^{1}H NMR (400.1 MHz) signal at δ 3.66 ppm (s) consistent with 18-crown-6. An independently prepared mixture of CsCl and 18-crown-6 in D_2O gave identical ^{1}H and ^{133}Cs NMR spectra, supporting assignment of the byproduct as (18-c-6)CsCl.[9-10]

^{1}H NMR (500 MHz, Tol-d_8/THF-d_8 (15:1), 298 K): δ 7.21 (t, 2 H, J = 7.8 Hz, Ar), 7.12-6.95 (m overlapped with residual solvent peak from Tol-d_8, 10 H, Ar), 6.27 (s, 2 H, CH-imidazole), 5.16 (s, 1 H, γ-CH), 3.67 (sept, 2 H, J = 6.9 Hz, CH-^{i}Pr), 3.65 (s, 1 H, C=CH$_2$), 3.54 (sept, 2 H, J = 6.9 Hz, CH-^{i}Pr), 2.83 (s, 1 H, C=CH$_2$), 2.20 (sept, 4 H, J = 6.9 Hz, CH-IPr), 1.48 (s, 3 H. β-Me), 1.35 (d, 6 H, J = 6.8 Hz, Me-^{i}Pr), 1.20 (d, 6 H, J = 6.9 Hz, Me-^{i}Pr), 0.98 (m, 18 H, Me-^{i}Pr and Me-IPr), 0.91 (m, 18 H, Me-^{i}Pr and Me-IPr).

^{13}C{^{1}H} NMR (125.6 MHz, Tol-d_8/THF-d_8 (15:1), 298 K): *δ* 206.7 (C, carbene IPr), 155.4 (C$_q$, *C*=CH$_2$), 146.9, 146.9, 146.0, 145.6 (C$_q$, Ar), 145.4 (C$_q$, *C*-Me), 144.4, 144.3, 143.6 (C$_q$, Ar), 134.4 (C$_q$, IPr), 134.4, 130.2 (Ar, IPr), 124.2 (CH, Ar), 123.5 (CH, Ar), 122.9 (CH, Ar), 122.9 (HC=CH, IPr), 102.0 (*γ*-CH), 78.1 (*CH$_2$*=C), 28.9 (CH-IPr), 28.0 (CH-^{i}Pr), 27.8 (Me-^{i}Pr), 26.6 (CH-^{i}Pr), 26.4 (Me-^{i}Pr), 25.0 (Me-IPr), 24.8 (Me-^{i}Pr), 23.8 (β-Me), 23.4 (Me-IPr).

Elemental analysis found (calc.) for $C_{56}H_{76}AuGaN_4$: C 62.94 (62.75), H 7.29 (7.15), N 4.94 (5.23).

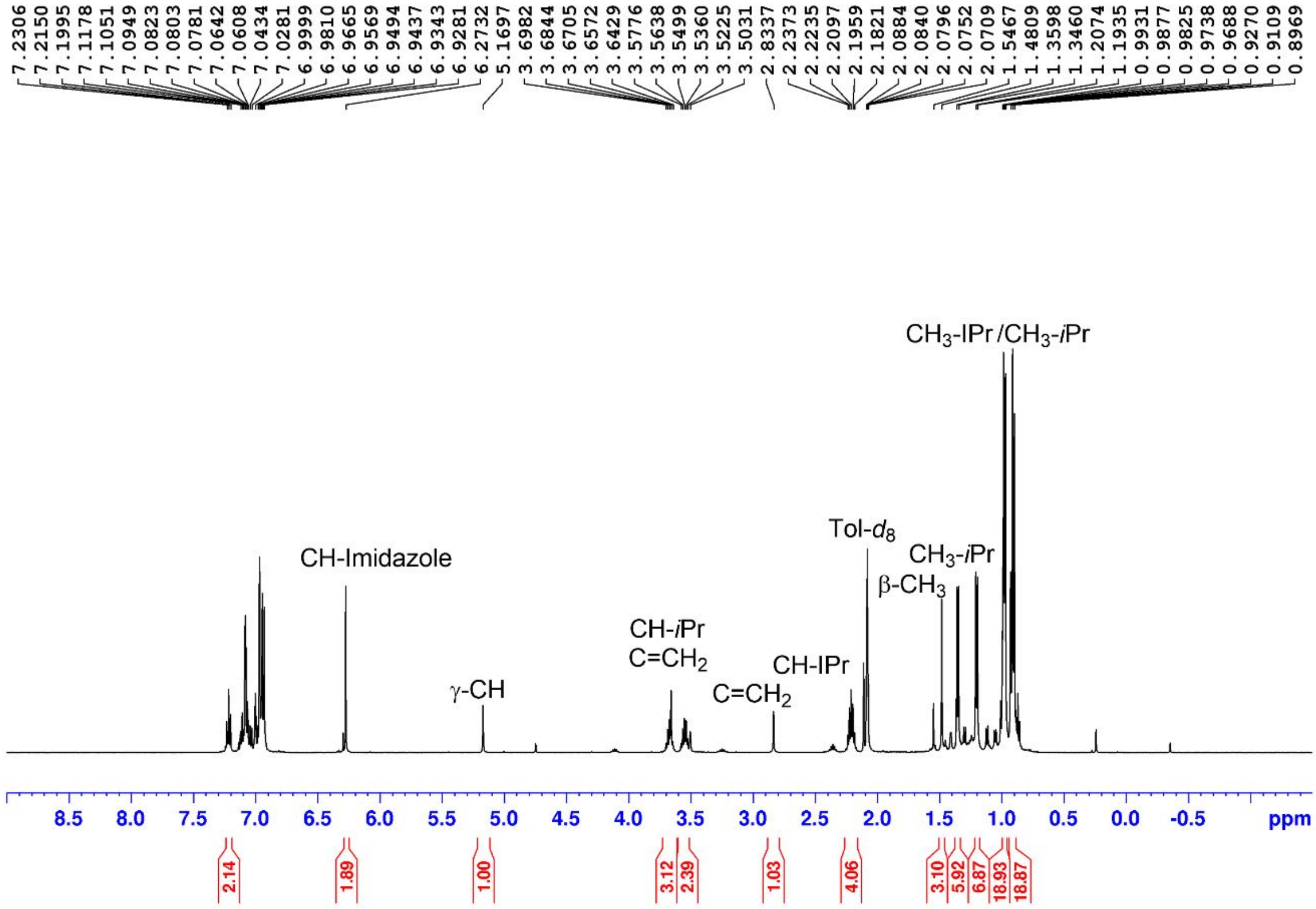

Figure S27. ^{1}H NMR (500 MHz, Tol-d_8/THF-d_8 (15:1), 298 K) spectrum of **3**.

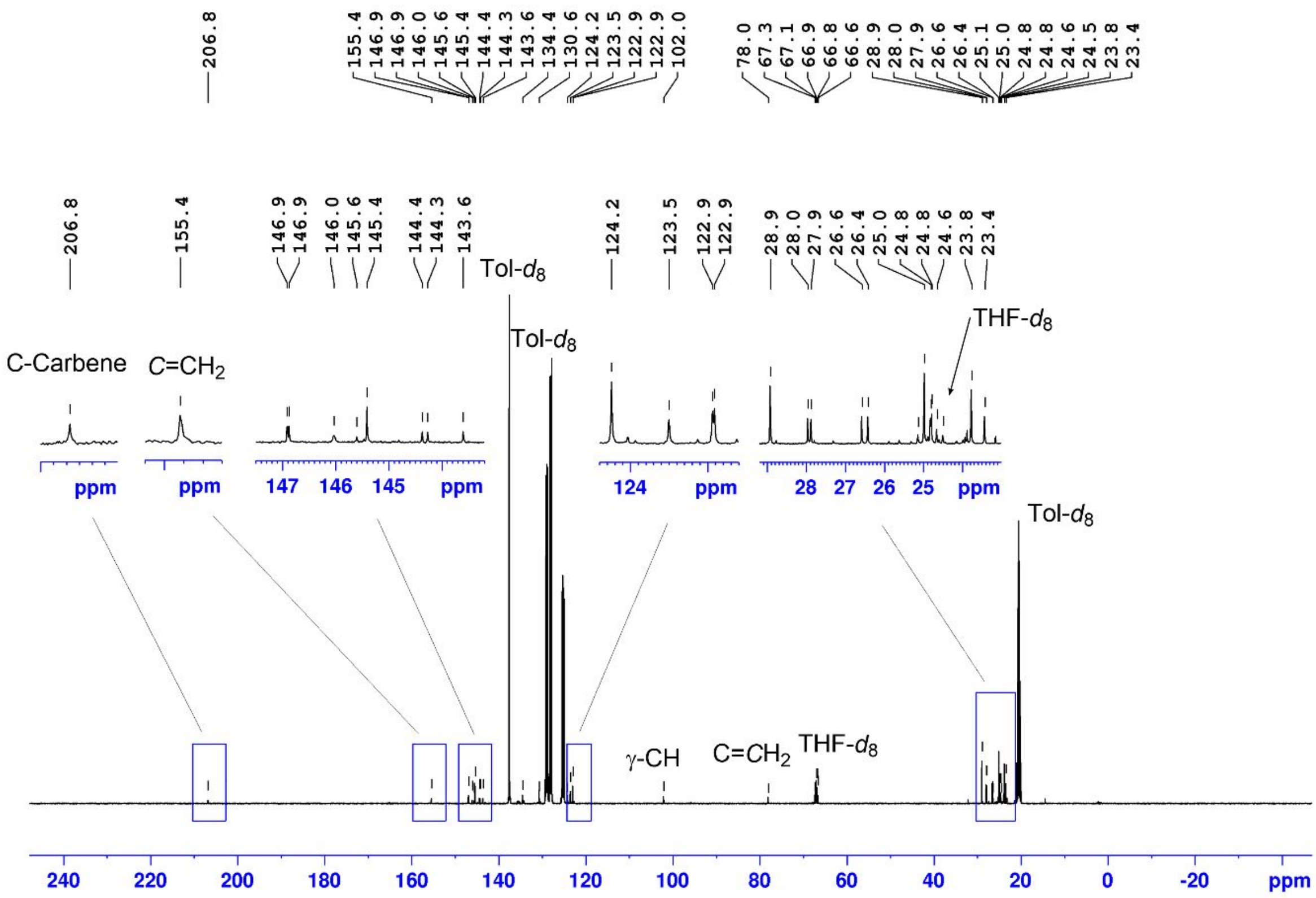

Figure S28. $^{13}C\{^{1}H\}$ NMR (125.6 MHz, Tol-d_8/THF-d_8 (15:1), 298 K) spectrum of **3**.

S3.2 Synthesis and characterization of 4

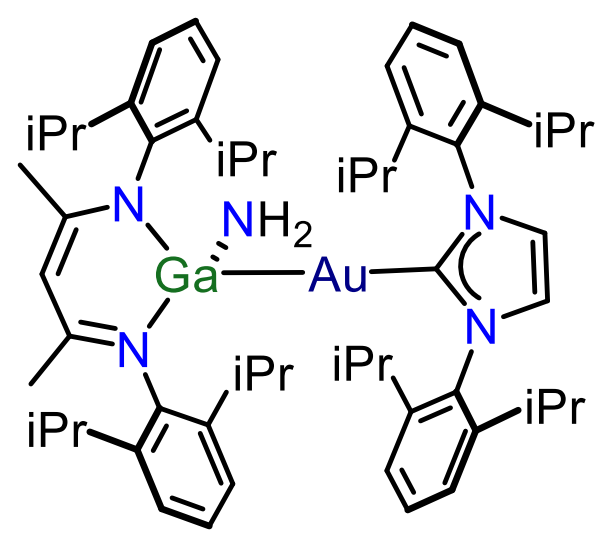

In a glovebox, a J. Young tube was charged with **3** (20 mg, 0.019 mmol) in Tol-d_8/THF-d_8 (15:1, 0.5 mL) at room temperature. The resulting solution was degassed by three freeze–pump–thaw cycles, charged with NH_3 (1.0 bar) and kept at room temperature. *In situ* ^{1}H NMR spectroscopy showed complete conversion to **4** within 2 min. The reaction solution was then layered with *n*-hexane (1 mL) under 1.0 bar of NH_3 and left to stand at room temperature, affording **4** as colourless plate-like crystals suitable for single-crystal X-ray diffraction (9 mg, 0.008 mmol, 43%).

^{1}H NMR (400 MHz, Tol-*d*₈/THF-*d*₈ (15:1), 298 K): *δ* 7.23 (t, 4 H, J = 7.8 Hz, Ar), 7.14-6.90 (m overlapped with residual solvent peak from Tol-d_8, 8 H, Ar), 6.25 (s, 2 H, CH-imidazole), 4.68 (s, 1 H, γ-CH), 3.97 (sept, 2 H, J = 6.7 Hz, CH-^{i}Pr), 3.36 (sept, 2 H, J = 6.9 Hz, CH-^{i}Pr), 2.38 (sept, 4 H, J = 6.8 Hz, CH-IPr), 1.54 (s, 6 H, β-Me), 1.31 (d, 6 H, J = 6.9 Hz, Me-^{i}Pr), 1.12 (d, 6 H, J = 6.9 Hz, Me-^{i}Pr), 1.08 (d, 6 H, J = 6.6 Hz, Me-^{i}Pr), 1.01 (d, 24 H, J = 6.5 Hz, Me-IPr), 0.95 (d, 6 H, J = 6.9 Hz, Me-^{i}Pr), –0.07 (br s, 2H, NH_2).

^{13}C{^{1}H} NMR (100.5 MHz, Tol-d_8/THF-d_8 (15:1), 298 K): *δ* 164.7 (C, carbene IPr), 145.6 (IPr), 145.4 (C_q, Ar), 145.2 (C_q, *C*-Me), 143.0 (C_q, Ar), 135.1 (C_q, IPr), 130.2 (Ar, IPr), 124.0 (CH, Ar), 123.8 (CH, Ar), 123.2 (CH, Ar), 122.5 (HC=CH, IPr), 94.8 (*γ*-CH), 28.9 (CH-IPr), 28.8 (CH-^{i}Pr), 28.3 (Me-^{i}Pr), 27.6 (CH-^{i}Pr), 25.4 (Me-^{i}Pr), 24.8 (Me-IPr), 24.8 (Me-^{i}Pr), 24.0 (Me-^{i}Pr), 24.0 (β-Me), 24.0 (Me-IPr).

Bulk isolation of **4** remains challenging outside an NH_3 atmosphere because of its pronounced sensitivity towards adventitious moisture, which leads to rapid conversion to **5**. Reliable elemental analyses could therefore not be obtained.

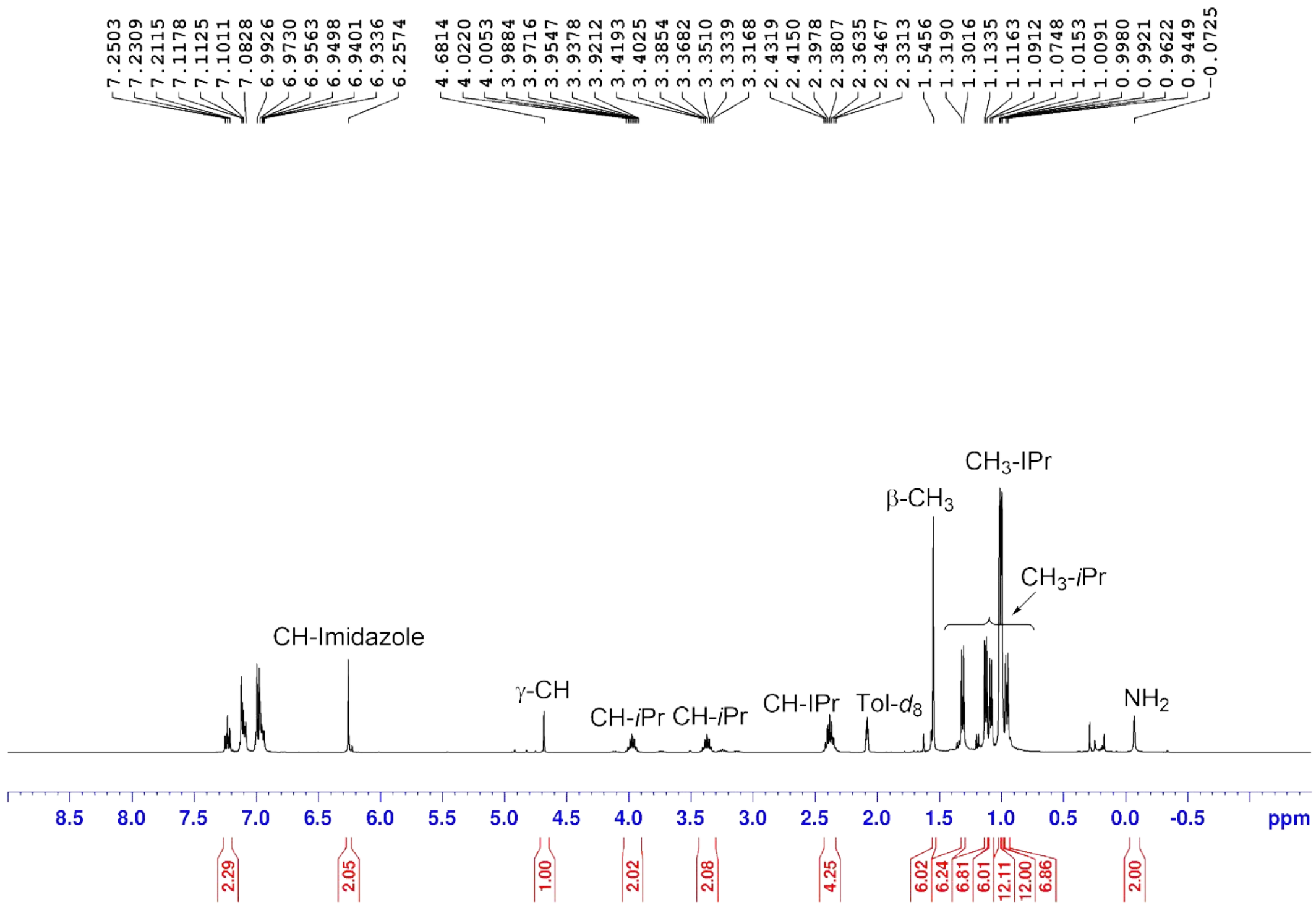

Figure S29. ^{1}H NMR (400 MHz, Tol-d_8/THF-d_8 (15:1), 298 K) spectrum of **4**.

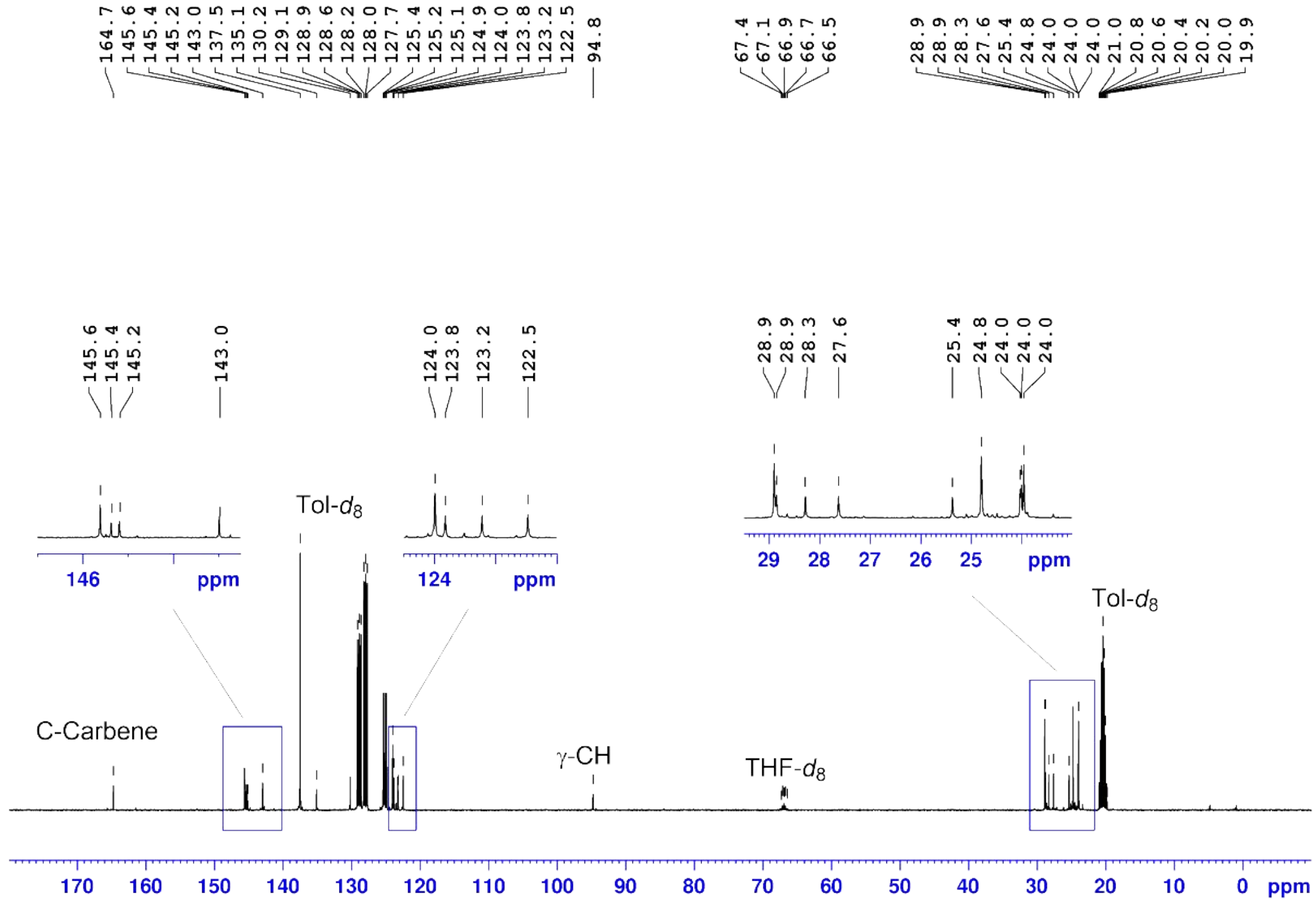

Figure S30. ^{13}C{^{1}H} NMR (100.5 MHz, Tol-d_8/THF-d_8 (15:1), 298 K) spectrum of **4**.

S3.3 Synthesis and characterization of 5

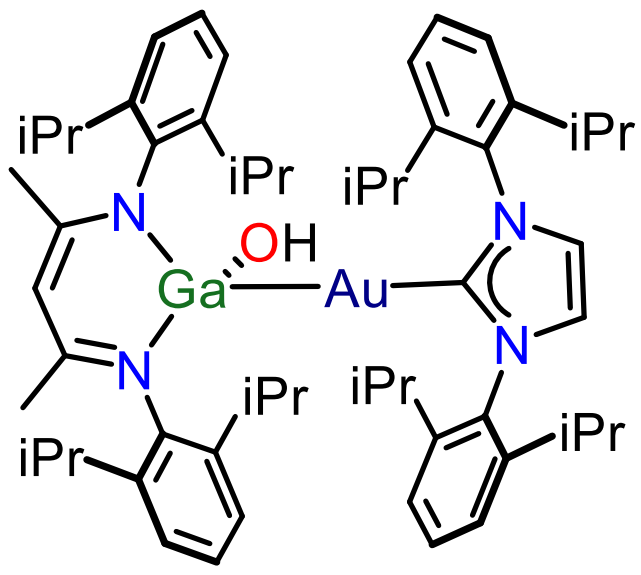

In a glovebox, a J. Young ampoule was charged with **3** (172 mg, 0.161 mmol) and toluene (5 mL). The reaction mixture was then briefly exposed to ambient atmosphere at room temperature for 2 min, after which the solution was concentrated under reduced pressure (2.5 mL) and layered with *n*-hexane (10 mL). After standing at room temperature for 16 h, **5** was obtained as colourless block-like crystals suitable for single-crystal X-ray diffraction (54 mg, 0.050 mmol, 31%).

An analogous reaction monitored by *in situ* ^{1}H NMR spectroscopy in Tol-d_8 showed clean conversion of **3** to **5** within 2 min upon brief exposure to ambient atmosphere.

^{1}H NMR (400 MHz, Tol-d_8, 298 K): δ 7.22 (t, 2 H, J = 7.7 Hz, Ar-IPr), 7.12-6.95 (m overlapped with residual solvent peak from Tol-d_8, 10 H, Ar), 6.19 (s, 2 H, CH-imidazole), 4.75 (s, 1 H, γ-CH), 4.11 (sept, 2 H, J = 6.8 Hz, CH-^{i}Pr), 3.25 (sept, 2 H, J = 6.8 Hz, CH-^{i}Pr), 2.35 (sept, 4 H, J = 6.8 Hz, CH-IPr), 1.56 (s, 6 H. β-Me), 1.33 (d, 6 H, J = 6.7 Hz, Me-^{i}Pr), 1.13 (d, 6 H, J = 6.9 Hz, Me-^{i}Pr), 1.07 (d, 6 H, J = 6.6 Hz, Me-^{i}Pr), 1.00 (m, 24 H, Me-IPr), 0.94 (d, 6 H, J = 6.8 Hz, Me-^{i}Pr), –0.26 (s, 1H, OH).

^{13}C{^{1}H} NMR (125.6 MHz, Tol-d_8, 298 K): *δ* 165.2 (C, carbene IPr), 145.6 (IPr), 145.5 (C_q, Ar), 144.7 (C_q, *C*-Me), 143.0 (C_q, Ar), 134.9 (C_q, IPr), 130.3 (Ar, IPr), 124.0 (CH, Ar), 124.0 (CH, Ar), 123.1 (CH, Ar), 122.5 (HC=CH, IPr), 95.7 (*γ*-CH), 28.9 (CH-IPr), 28.8 (CH-^{i}Pr), 27.8 (Me-^{i}Pr), 27.3 (CH-^{i}Pr), 25.6 (Me-^{i}Pr), 24.9 (Me-IPr), 24.6 (Me-^{i}Pr), 24.1 (Me-^{i}Pr), 23.9 (β-Me), 23.9 (Me-IPr).

Elemental analysis found (calc.) for $C_{56}H_{78}AuGaN_4O$: C 61.69 (61.71), H 7.14 (7.21), N 4.81 (5.14).

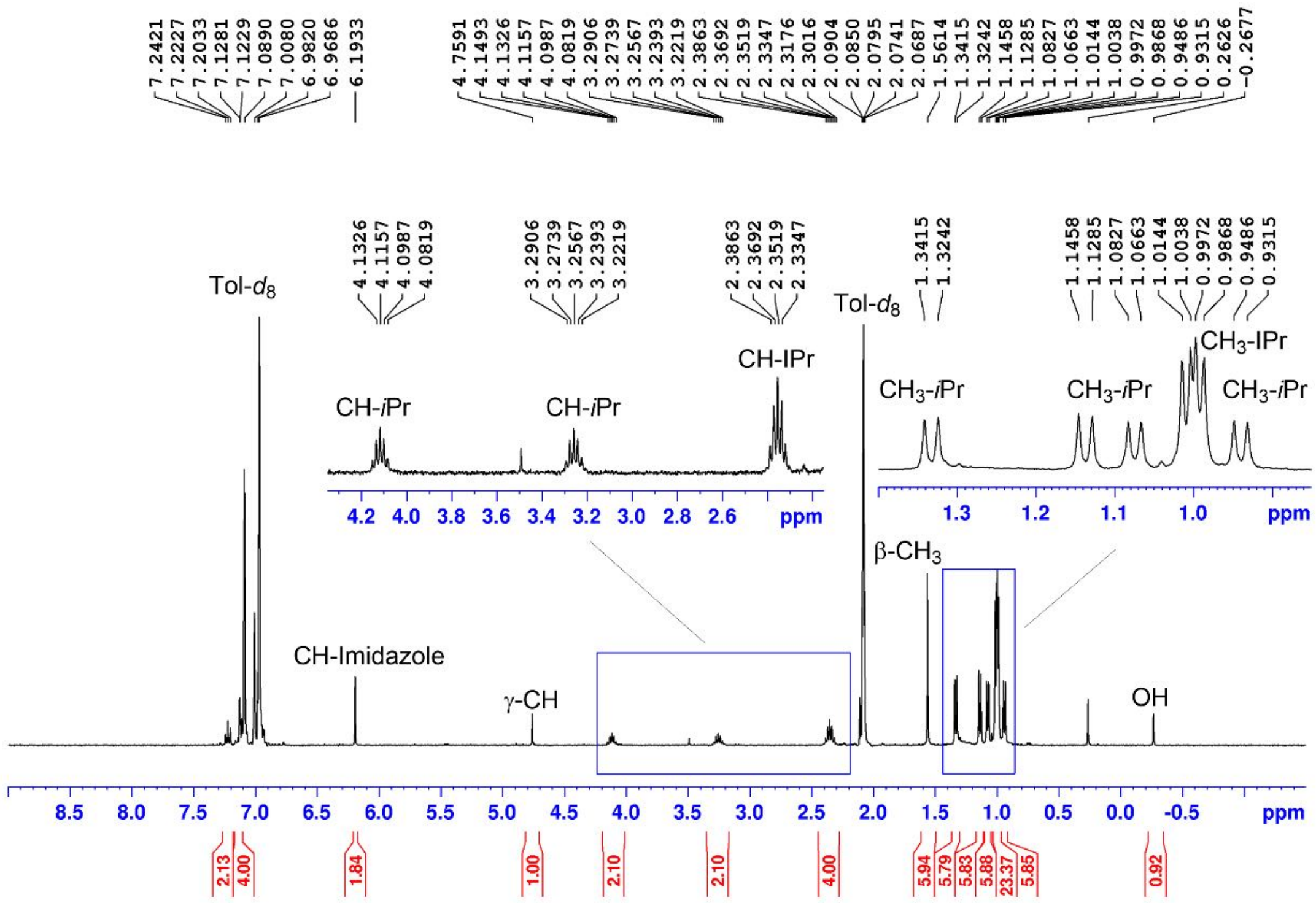

Figure S31. ^{1}H NMR (400 MHz, Tol-d_8, 298 K) spectrum of **5**.

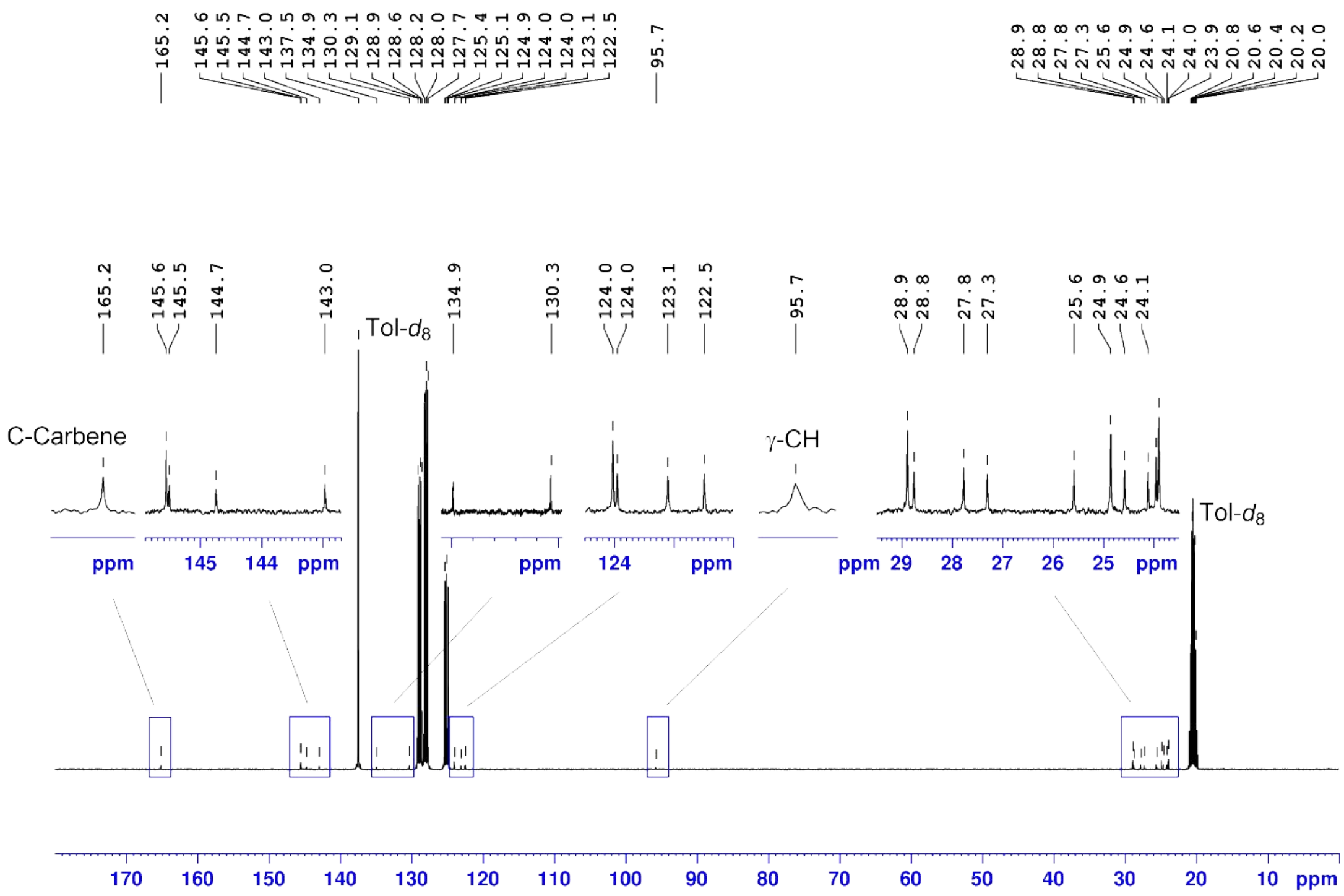

Figure S32. ^{13}C{^{1}H} NMR (125.6 MHz, Tol-d_8, 298 K) spectrum of **5**.

S3.4 Synthesis and characterization of 6

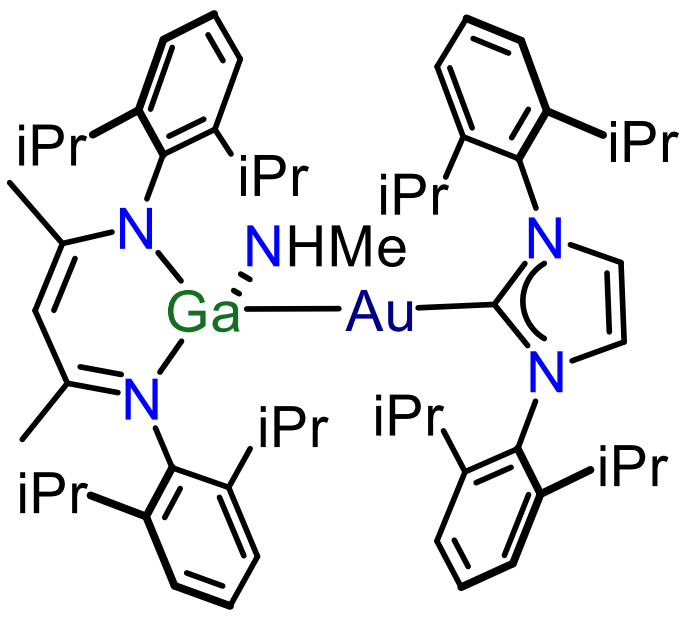

In a glovebox, a J. Young ampoule was charged with **3** (55 mg, 0.05 mmol) and toluene (1 mL). A commercial solution of $MeNH_2$ in THF (2.0 M, 35 µL, 0.070 mmol) was then added. The reaction mixture was stirred at room temperature for 2 min to give a colourless solution. Volatiles were removed under reduced pressure to afford **6** as a colourless solid, which was washed with *n*-hexane (1 mL) and dried under vacuum (20 mg, 0.018 mmol, 36%). Prism-like single crystals suitable for single-crystal X-ray diffraction were obtained from a solution of **6** in toluene/THF/n-hexane by slow evaporation at room temperature over 24 h.

^{1}H NMR (400 MHz, Tol-*d*$_8$, 298 K): δ 7.23 (t, 4 H, J = 7.8 Hz, Ar), 7.17-6.98 (m overlapped with residual solvent peak from Tol-*d*$_8$, 10 H, Ar), 6.20 (s, 2 H, CH-imidazole), 4.67 (s, 1 H, γ-CH), 3.87 (sept, 2 H, J = 6.6 Hz, CH-^{i}Pr), 3.33 (sept, 2 H, J = 6.9 Hz, CH-^{i}Pr), 3.07 (d, 3 H, J = 6.8 Hz, *Me*NH), 2.37 (sept, 4 H, J = 6.8 Hz, CH-IPr), 1.57 (s, 6 H. β-Me), 1.38 (d, 6 H, J = 6.9 Hz, Me-^{i}Pr), 1.21 (d, 6 H, J = 6.5 Hz, Me-^{i}Pr), 1.15 (d, 6 H, J = 6.8 Hz, Me-^{i}Pr), 0.99 (m, 24 H, Me-IPr), 0.94 (d, 6 H, J = 6.9 Hz, Me-^{i}Pr), –0.18 (br q, 1 H, MeN*H*).

^{13}C{^{1}H} NMR (125.6 MHz, Tol-*d*$_8$, 298 K): *δ* 164.4 (C, carbene IPr), 145.6 (IPr), 145.5 (C_q, Ar), 145.4 (C_q, *C*-Me), 143.1 (C_q, Ar), 135.1 (C_q, IPr), 130.2 (Ar, IPr), 124.0 (CH, Ar), 123.9 (CH, Ar), 123.3 (CH, Ar), 123.3 (CH, Ar), 122.4 (HC=CH, IPr), 95.5 (*γ*-CH), 35.9 (*Me*NH), 28.9 (CH-IPr), 28.8 (CH-^{i}Pr), 27.2 (Me-^{i}Pr), 27.2 (CH-^{i}Pr), 25.7 (Me-^{i}Pr), 24.8 (Me-IPr), 24.7 (Me-^{i}Pr), 24.6 (Me-^{i}Pr), 24.0 (Me-IPr), 23.9 (β-Me).

Elemental analysis found (calc.) for $C_{57}H_{81}AuGaN_5$: C 62.17 (62.07), H 7.35 (7.40), N 5.90 (6.35).

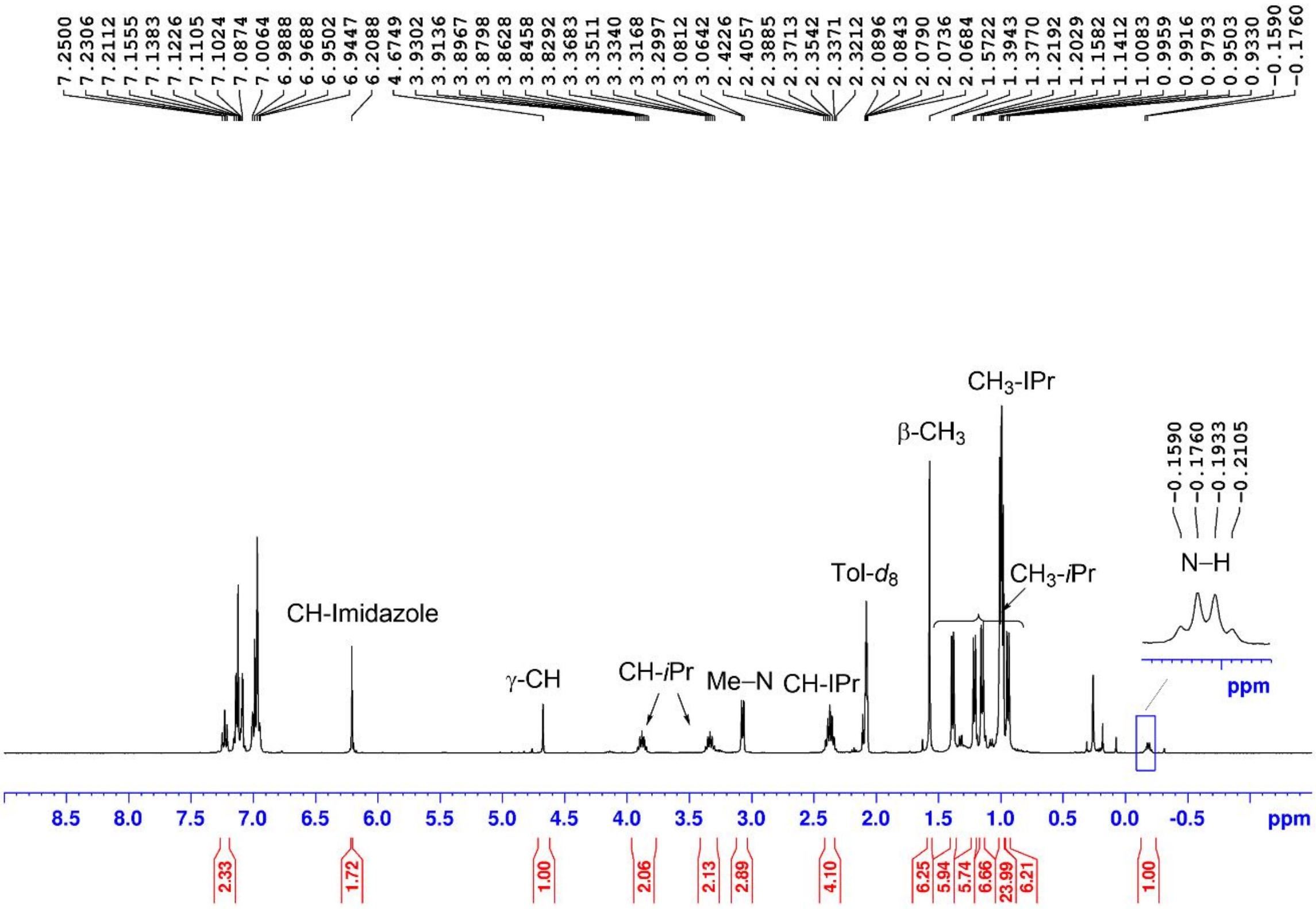

Figure S33. ^{1}H NMR (400 MHz, Tol-d_8, 298 K) spectrum of **6**.

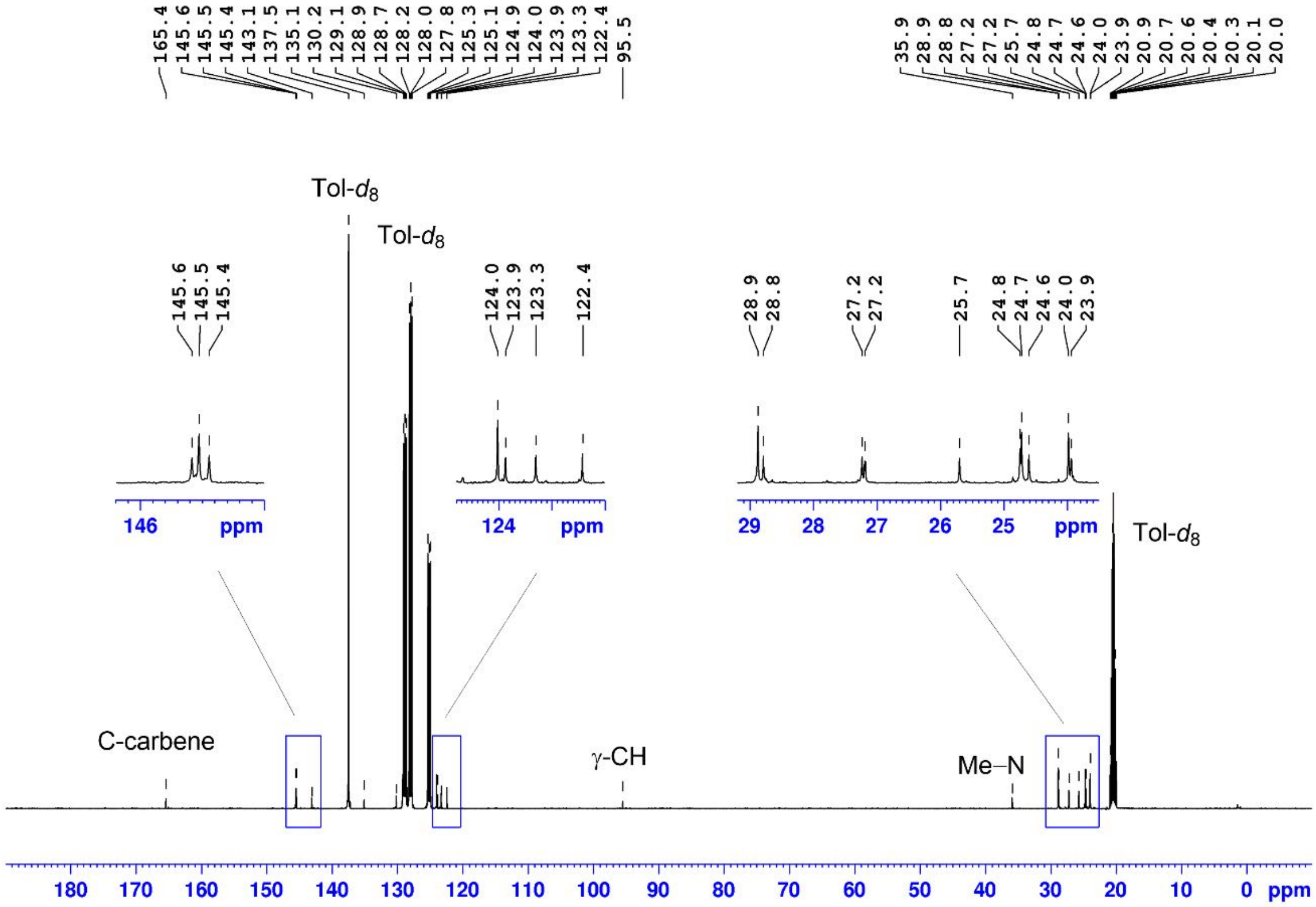

Figure S34. $^{13}C\{^{1}H\}$ NMR (125.6 MHz, Tol-d_8, 298 K) spectrum of **6**.

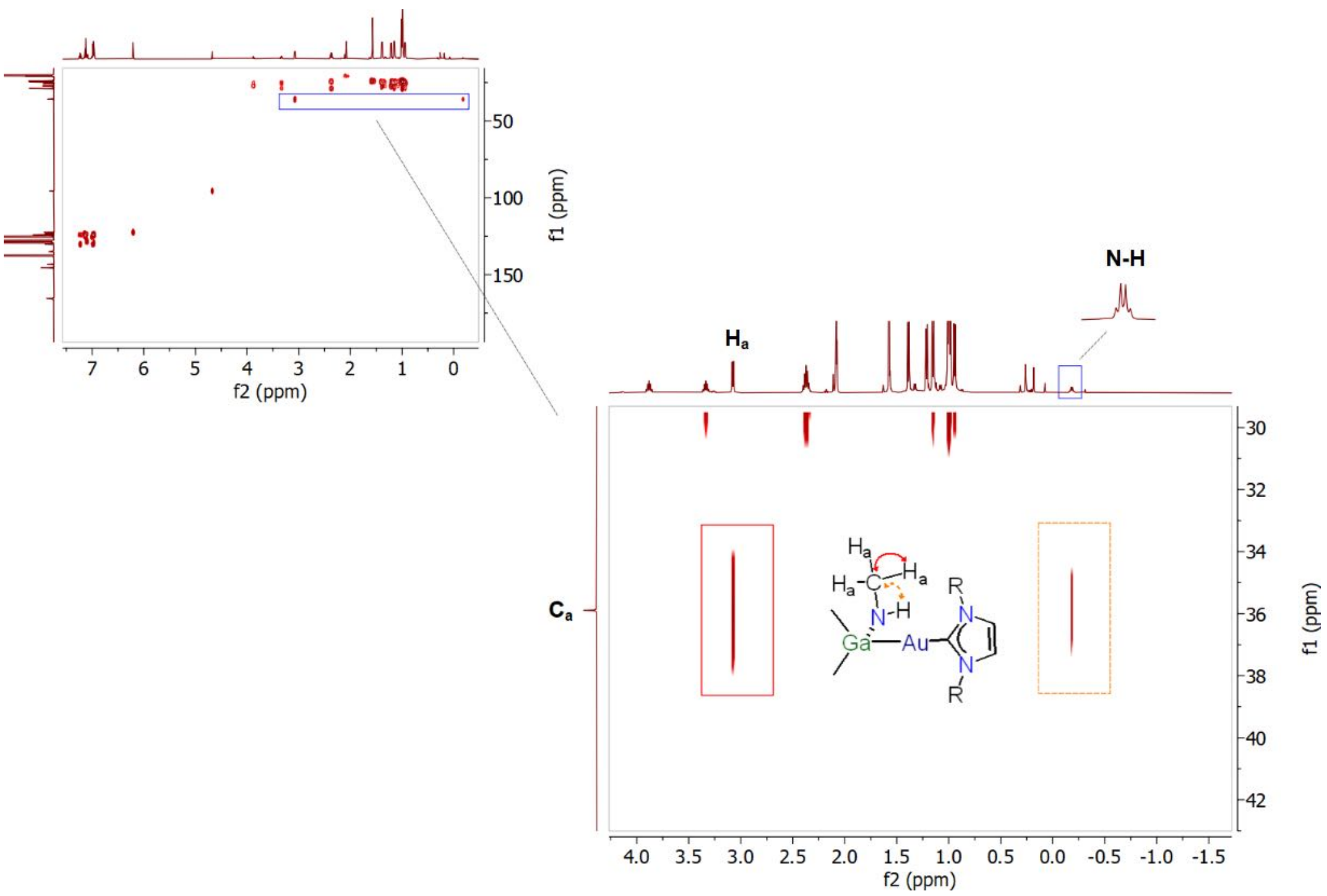

Figure S35. ^{1}H–^{13}C HSQC-TOCSY NMR (500 MHz, Tol-d_8, 298 K) spectrum of **6**.

S3.5 Synthesis and characterization of 7

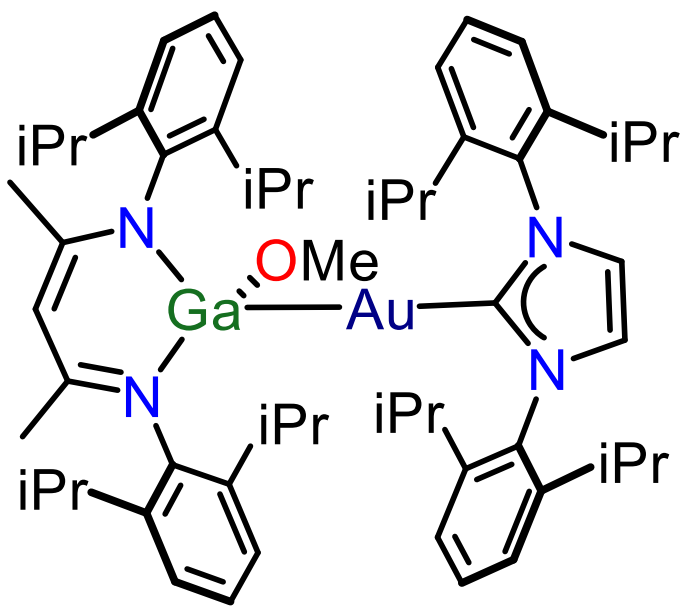

In a glovebox, a J. Young ampoule was charged with **3** (55 mg, 0.050 mmol) and toluene (1 mL). Dry MeOH (40 µL, 1 mmol) was then added, and the reaction mixture was stirred at room temperature for 2 min to give a colourless solution. Volatiles were removed under reduced pressure to afford a white solid, which was washed with n-hexane (1 mL) and dried under vacuum to give **7** (30 mg, 0.027 mmol, 54%). Needle-like single crystals suitable for single-crystal X-ray diffraction were obtained from a solution of **7** in toluene/n-hexane after storage at −33°C for 24 h.

^{1}H NMR (400 MHz, Tol-d_8, 298 K): δ 7.22 (t, 2 H, J = 7.8 Hz, Ar-IPr), 7.17-6.91 (m overlapped with residual solvent peak from Tol-d_8, 10 H, Ar), 6.22 (s, 2 H, CH-imidazole), 4.76 (s, 1 H, γ-CH), 4.05 (sept, 2 H, J = 6.8 Hz, CH-^{i}Pr), 3.69 (s, 3 H, *Me*O), 3.22 (sept, 2 H, J = 6.8 Hz, CH-^{i}Pr), 2.39 (sept, 4 H, J = 6.9 Hz, CH-IPr), 1.53 (s, 6 H. β-Me), 1.34 (d, 6 H, J = 6.9 Hz, Me-^{i}Pr), 1.20 (d, 6 H, J = 6.6 Hz, Me-^{i}Pr), 1.13 (d, 6 H, J = 6.9 Hz, Me-^{i}Pr), 0.99 (m, 24 H, Me-IPr), 0.92 (d, 6 H, J = 6.9 Hz, Me-^{i}Pr).

^{13}C{^{1}H} NMR (100.5 MHz, Tol-d_8, 298 K): *δ* 165.5 (C, carbene IPr), 145.6 (IPr), 145.6 (C_q, Ar), 145.4 (C_q, Ar), 145.2 (C_q, *C*-Me), 143.1 (C_q, Ar), 135.0 (C_q, IPr), 130.2 (Ar, IPr), 124.1 (CH, Ar), 124.0 (CH, Ar), 123.2 (CH, Ar), 122.6 (HC=CH, IPr), 96.8 (*γ*-CH), 55.2 (MeO), 28.9 (CH-IPr), 28.7 (CH-^{i}Pr), 27.6 (Me-^{i}Pr), 27.3 (CH-^{i}Pr), 25.8 (Me-^{i}Pr), 24.6 (Me-IPr), 24.6 (Me-^{i}Pr), 24.1 (Me-^{i}Pr), 24.1 (β-Me).

Elemental analysis found (calc.) for $C_{57}H_{80}AuGaN_4O\cdot(C_7H_8)_{0.75}$: C 63.76 (63.74), H 7.29 (7.39), N 4.99 (4.78).

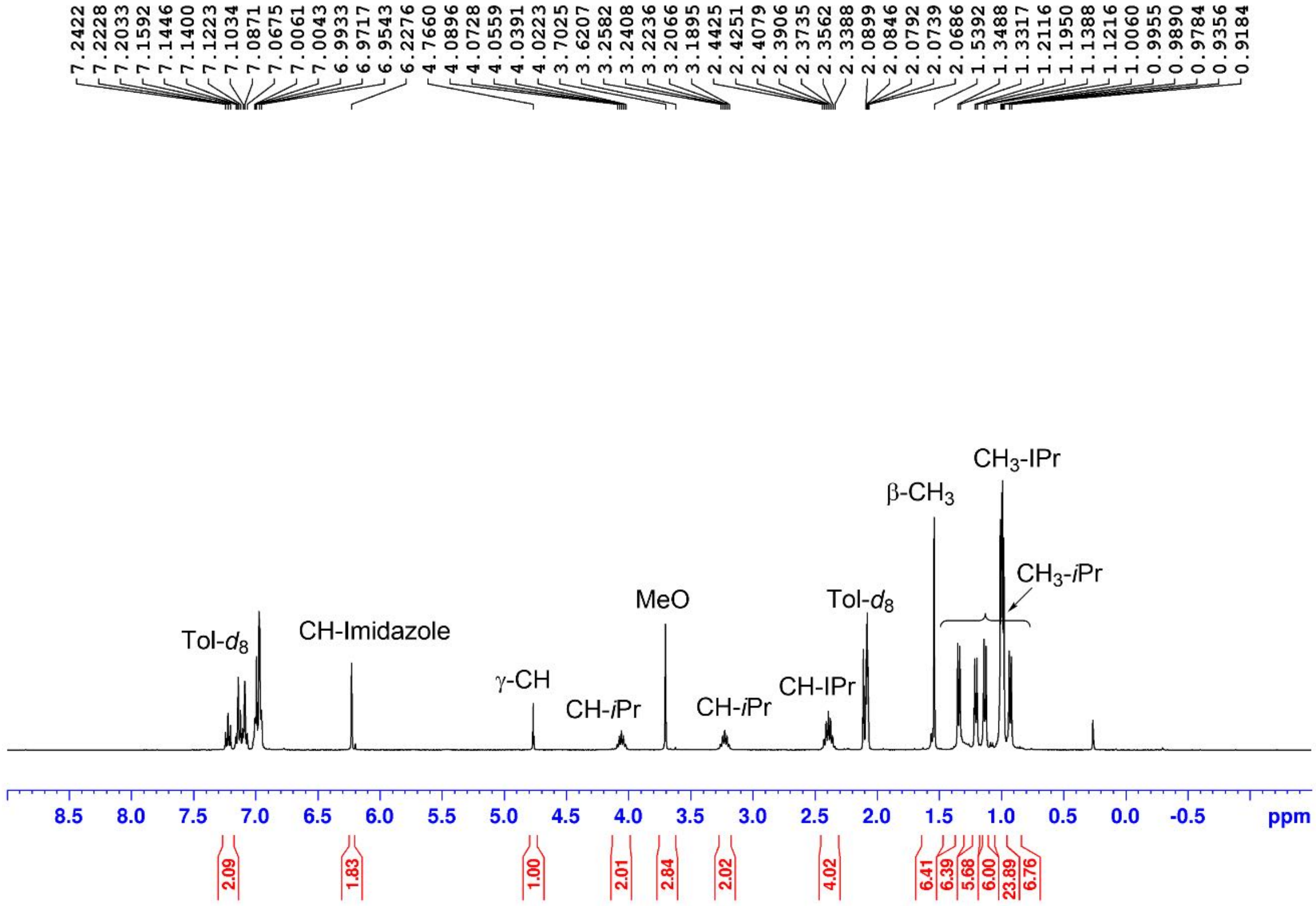

Figure S36. ^{1}H NMR (400 MHz, Tol-d_8, 298 K) spectrum of **7**.

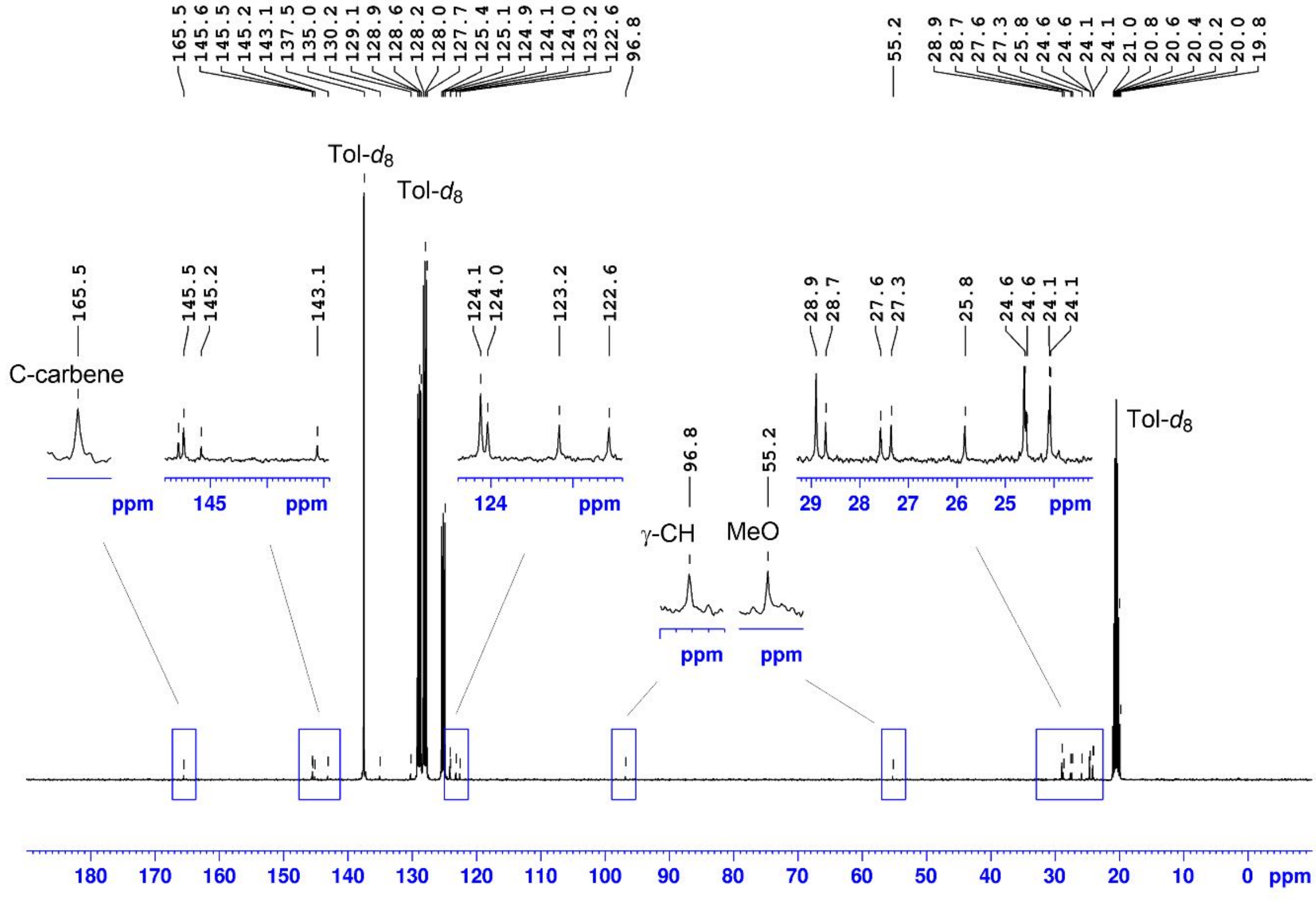

Figure S37. ^{13}C{^{1}H} NMR (100.5 MHz, Tol-d_8, 298 K) spectrum of **7**.

S3.6 Control experiments supporting the gallyl–gold reactivity

To distinguish the cooperative N–H/O–H activation reactivity of **3** from protic quenching of the free gallyl anions or independent reactivity of the Au(IPr) fragment, comparative NMR-scale control reactions were carried out under otherwise similar conditions to those used for the formation of **4**–**7**. In a typical experiment, a J. Young NMR tube was charged in the glovebox with the organometallic precursor and Tol-d_8 or Tol-d_8/THF-d_8. The substrate was then introduced as NH_3 gas, $MeNH_2$ solution in THF, dry MeOH, or by brief exposure to a moist atmosphere. Reaction progress was monitored by ^{1}H NMR spectroscopy at 298 K. The results summarized in Table S1 show that only the Ga–Au complex **3** gives clean conversion to the defined Ga–NH_2, Ga–NHMe, Ga–OH, and Ga–OMe products **4**–**7**. Free gallyl salts do not form defined amido products with NH_3 or $MeNH_2$, while H_2O and MeOH rapidly quench the gallyl resonances to give complex mixtures containing, at most, initial minor amounts of the reprotonated gallylene **1**. The Au precursor (IPr)AuCl remains unchanged under the same conditions.

Table S1. Summary of control reactions.

Entry	Precursor	Substrate	Conditions	Observation by ^{1}H NMR
**1**	**3**	NH_3	1 bar NH_3, Tol-d_8/THF-d_8, 298 K	Clean conversion to **4**, diagnostic Ga–NH_2 resonance observed
**2**	**3**	$MeNH_2$	$MeNH_2$ solution in THF, Tol-d_8, 298 K	Clean conversion to **6**, diagnostic Ga–NHMe resonance observed
**3**	**3**	H_2O	moist-atmosphere exposure 2 min, 298 K	Clean conversion to **5**, diagnostic Ga–OH resonance observed
**4**	**3**	MeOH	dry MeOH, Tol-d_8, 298 K	Clean conversion to **7**, diagnostic Ga–OMe resonance observed
**5**	**2·Cs(18-c-6)$_2$**	NH_3 or $MeNH_2$	conditions matched to entry 1 or 2	No defined **4**- or **6**-type products, complex reaction mixtures
**6**	representative **2·M(L)** salts[a]	NH_3 or $MeNH_2$	conditions matched to entries 1 or 2	No defined **4**- or **6**-type products, complex reaction mixtures
**7**	**2·Cs(18-c-6)$_2$**	H_2O	conditions matched to entry 3	Loss of gallyl resonances; complex mixture with transient minor signals attributable to **1**
**8**	representative **2·M(L)** salts[a]	MeOH	conditions matched to entry 4	Loss of gallyl resonances; complex mixture with transient minor signals attributable to **1**
**9**	(IPr)AuCl	NH_3, $MeNH_2$, H_2O, or MeOH	conditions matched to entries 1–4	(IPr)AuCl remains unchanged
**10**	**4** or **6**	H_2O	moist-atmosphere exposure 2 min, Tol-d_8 or Tol-d_8/THF-d_8, 298 K	Clean conversion to **5**

[a] Representative salts tested: **2·Na(crypt-222), 2·Cs(crypt-222)**, **2·K(18-c-6)**, **2·Cs(18-c-6)** and **[(2·K)$_2$(μ-KHMDS)$_2$]$_\infty$**.

S4 ^{1}H Diffusion-Ordered Spectroscopy (DOSY) NMR Studies

S4.1 ^{1}H DOSY NMR and hydrodynamic radii analyses

Diffusion-Ordered Spectroscopy (DOSY) NMR experiments were carried out on a Bruker AVIIIHD 9.4 Tesla Nanobay (400 MHz for ^{1}H) equipped with a BBF/19F-1H/D-5.0-z probe, or a Bruker AVIII 11.7 Tesla equipped with a BBO/19F-1H/D-5.0-z Prodigy cryoprobe. All experiments were conducted at 298 K using TopSpin software (version 4.1, Bruker Biospin, Karlsruhe), on systems with an actively shielded z-gradient coil capable of providing a maximum gradient strength of 5.05 G mm^{-1} at 10 A. Data for diffusion-ordered NMR were collected using the Bruker pulse program ledgpgp2s1d with a double stimulated echo. Sine-shaped gradient pulses with a duration of D21 of 5 ms were used, coupled with a diffusion period D20 of 100 ms, bipolar gradient pulses P30 of 650 µsec, and two spoiling gradients P19 of 600 µsec. Following the application of each gradient pulse, a gradient recovery delay of 200 µs was implemented. Data were systematically compiled by linearly varying the diffusion encoding gradients from 5% to 95% of the maximum for 32 gradient increment values. The pulse program ledgpgp2s was employed for data acquisition of this pseudo-2D experiment. The signal decay dimension on the pseudo-2D data was generated by Fourier transformation of the time-domain data.

DOSY plots were generated using the DOSY processing with the T1/T2 software package module of TopSpin (version 4.1, Bruker Biospin, Karlsruhe), fitting area data (integration of all peaks of interest of the sample) of diffusion decays. Parameters were empirically optimized to achieve the highest quality of data presentation. Diffusion coefficients (D_x) were calculated by fitting intensity data to the Stejskal-Tanner expression and then normalized ($D_{x,norm}$) using adamantane as an external standard to fit the reference fixed diffusion coefficients ($D_{ref,fix}$) standardized by Stalke.[11-12] External calibration for DOSY NMR studies was performed due to the high reactivity of the compounds being analyzed, using diffusion coefficients of external standards (1,2,3,4-tetraphenylnaphthalene, 1-phenylnaphthalene, adamantane, and tetramethylsilane, 15 mM each in either Tol-d_8, C_6D_6 or THF-d_8). Linear calibration graphs were obtained by plotting $log D_{x,norm}$ versus *log*MW. By utilizing the normalized diffusion coefficients for the corresponding compound's signals ($D_{x,norm}$, analyte), an estimate of the molecular weight (MW) of the species present in the solution was obtained (OEC, own external calibration method). Our external calibration method was compared with that reported by Stalke,[11-12] resulting in comparable MW values. In addition, a correction factor was applied to MW that scales incorporating heavier elements from van-der-Waals molecular densities when appropriate.[13] Air-sensitive samples were prepared in a J. Young NMR tube sealed under a nitrogen gas atmosphere containing the corresponding compound to be analyzed (typically, 7-14 mg) in an appropriate deuterated solvent or solvent mixture (0.5 mL).

The diameter of the diffusing species in solution for the corresponding compounds was estimated using the Stokes-Einstein equation: $D = K_BT/3\eta\pi d$. Here, D denotes the diffusion coefficient ($m^2\cdot s^{-1}$), π stands for 3.1415926, K_B represents Boltzmann's constant (1.3806505 J·K), T is the absolute temperature (298 K), and η signifies the solvent viscosity (not the solution viscosity) at 298 K (0.56×10^{-3} pa·s for toluene, 0.456×10^{-3} pa·s for tetrahydrofuran).[14]

According to this equation, the hydrodynamic diameter (D_{hyd}) of the diffusing species in solution is $D_{hyd} = K_BT/3\eta\pi D$ (m). From the diffusion coefficients ($D_{x,norm}$, $m^2\cdot s^{-1}$) of the external standards used (1,2,3,4-tetraphenylnaphthalene, 1-phenylnaphthalene, adamantane and tetramethylsilane, 15 mM each in either Tol-d_8 or THF-d_8), linear calibration graphs were obtained by plotting $log D_{x,norm}$ versus $log D_{X\text{-}ray}$, where $D_{X\text{-}ray}$ is an estimate of the radius of the compounds extracted from their corresponding molecular structures (5 Å for tetramethylsilane,[15] 6 Å for adamantane,[16] 8 Å for 1-phenylnaphthalene[17] and 11.5 Å for 1,2,3,4-tetraphenylnaphthalene).[18] $D_{X\text{-}ray}$ (Å) was estimated by calculating an average value from the corresponding molecular structures. This calculation utilized a centered spheric probe with a given radius (Å) in conjunction with a space-filling model that uses van der Waals radii reported by S. Álvarez for all atoms.[19] The correlation between the estimated diameter values extracted from the molecular structures and calculated from the solution showed good agreement (6.4 Å for adamantane calculated from thermodynamic solution studies[17] and 5 Å calculated from reported[20] DOSY studies). From the diffusion coefficients of the external standards used (1,2,3,4-tetraphenylnaphthalene, 1-phenylnaphthalene, adamantane and tetramethylsilane, 15 mM each in either Tol-d_8 or THF-d_8), linear calibration graphs were obtained by plotting $log D_{x,norm}$ versus $log D_{X\text{-}ray}$. Furthermore, using the diffusion coefficients for the signals from the corresponding standard, an estimate of the diffusing species present in the solution was achieved, which showed excellent agreement with the estimated $D_{X\text{-}ray}$ values extracted from the molecular structures. Finally, by using the diffusion coefficients for the signals from the corresponding compound, an estimate of the D_{hyd} of the species present in the solution was obtained, which was compared with that estimated $D_{X\text{-}ray}$ values extracted from the molecular structures (see Figure S38, Figure S39, Figure S40 and Figure S41).

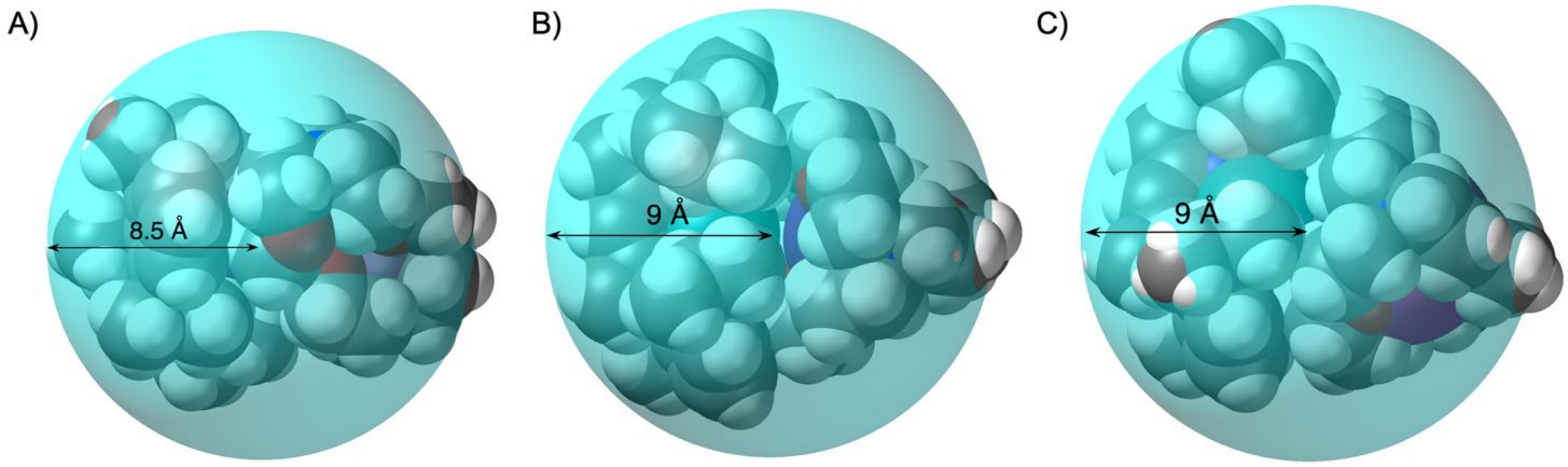

Figure S38. Estimated molecular diameters ($D_{X\text{-}ray}$, Å) for A) **2·Li(crypt-222)** (17 Å), B) **2·K(crypt-222)** (18 Å) and C) **2·Cs(crypt-222)** (18 Å) obtained from their molecular structures. These estimations were calculated using a geometrically centered spherical probe of the given radii (Å) within space-filling models, utilizing van der Waals radii for all atoms as reported by S. Álvarez.[19]

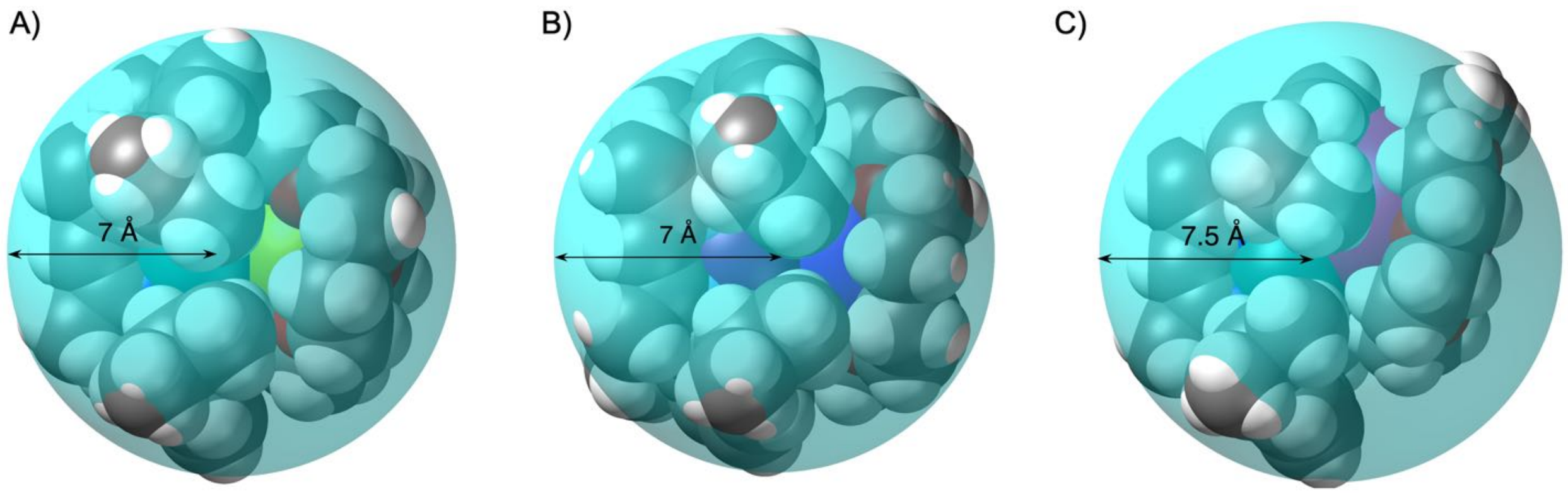

Figure S39. Estimated molecular diameters ($D_{X\text{-}ray}$, Å) for A) **2·Na(18-c-6)** (14 Å), B) **2·K(18-c-6)** (14 Å) and C) **2·Cs(18-c-6)** (15 Å), obtained from their molecular structures. These estimations were calculated using a geometrically centered spherical probe of the given radii (Å) within space-filling models, utilizing van der Waals radii for all atoms as reported by S. Álvarez.[19]

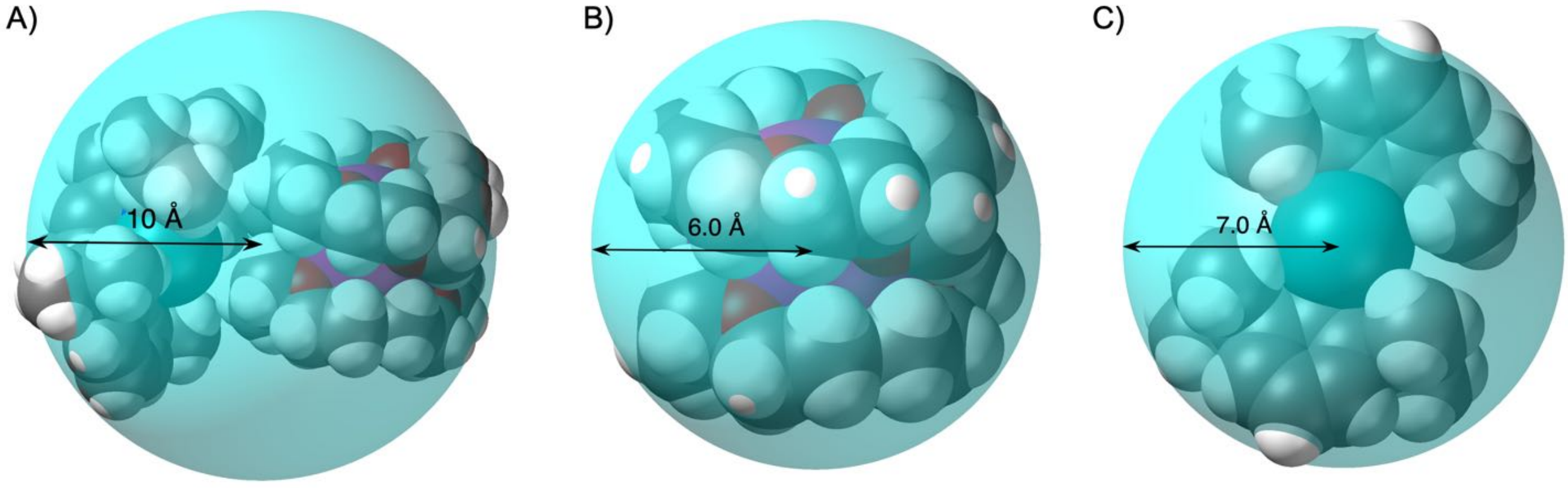

Figure S40. Estimated molecular diameters ($D_{X\text{-}ray}$, Å) for A) **2·Cs(18-c-6)₂** (20 Å), and B) [Cs(18-c-6)$_2$]$^+$ (12 Å), and C) **2**$^-$ (14 Å) units obtained from the molecular structure of **2·Cs(18-c-6)₂**. These estimations were calculated using a geometrically centered spherical probe of the given radii (Å) within space-filling models, utilizing van der Waals radii for all atoms as reported by S. Álvarez.[19]

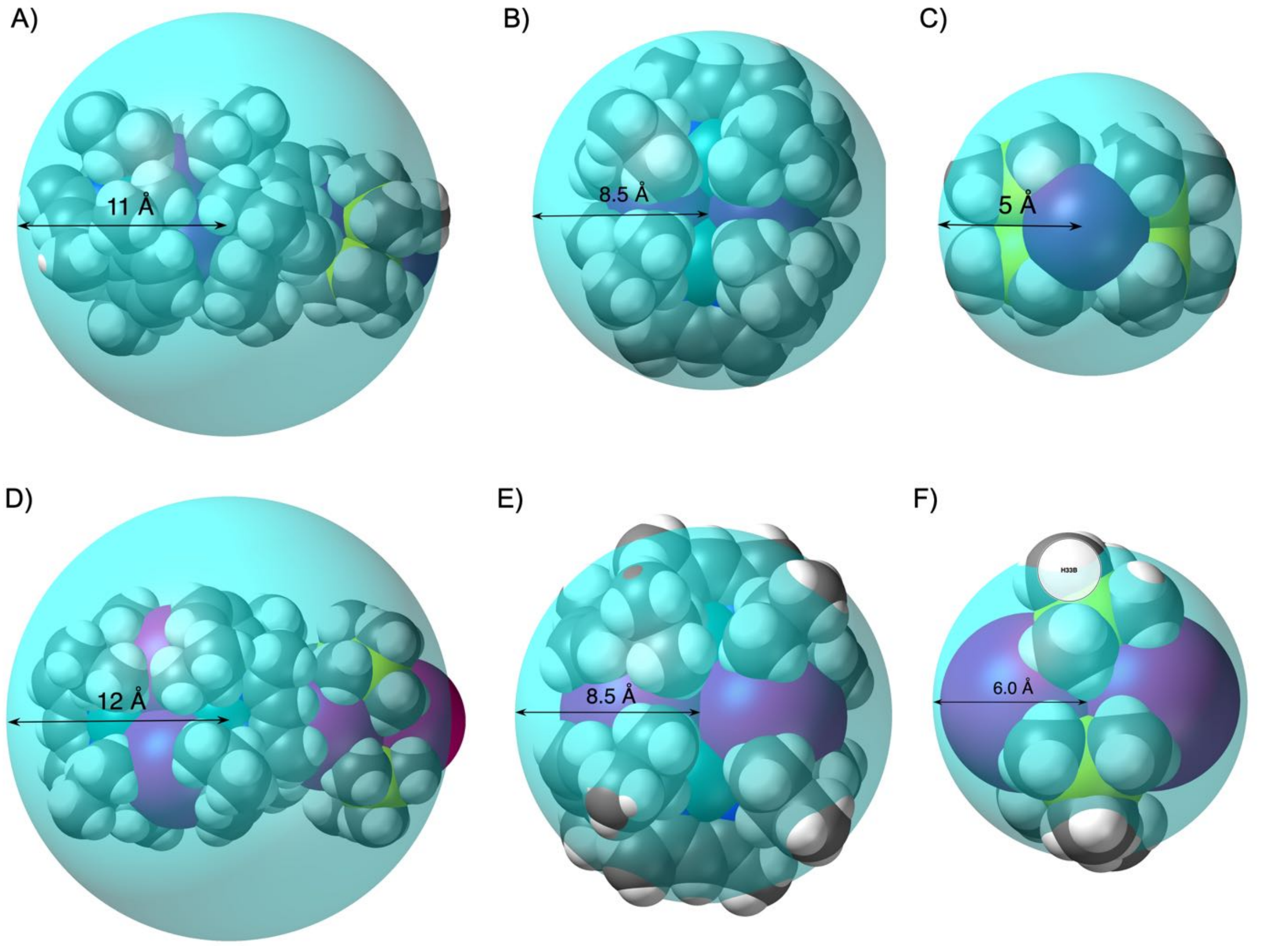

Figure S41. Estimated molecular diameters ($D_{X\text{-}ray}$, Å) for A) [**(2·K)$_2$(KHMDS)$_2$**] (22 Å), B) (**2·K**)$_2$ (17 Å), C) (KHMDS)$_2$ (10 Å), D) [(**2·Cs**)$_2$**(CsHMDS)$_2$**] (24 Å), E) (**2·Cs**)$_2$ (17 Å) and F) (CsHMDS)$_2$ (12 Å) obtained from their molecular structures. These estimations were calculated using a geometrically centered spherical probe of the given radii (Å) within space-filling models, utilizing van der Waals radii for all atoms as reported by S. Álvarez.[19]

S4.1.1 ^{1}H DOSY NMR spectroscopic study of 2·Li(crypt-222)

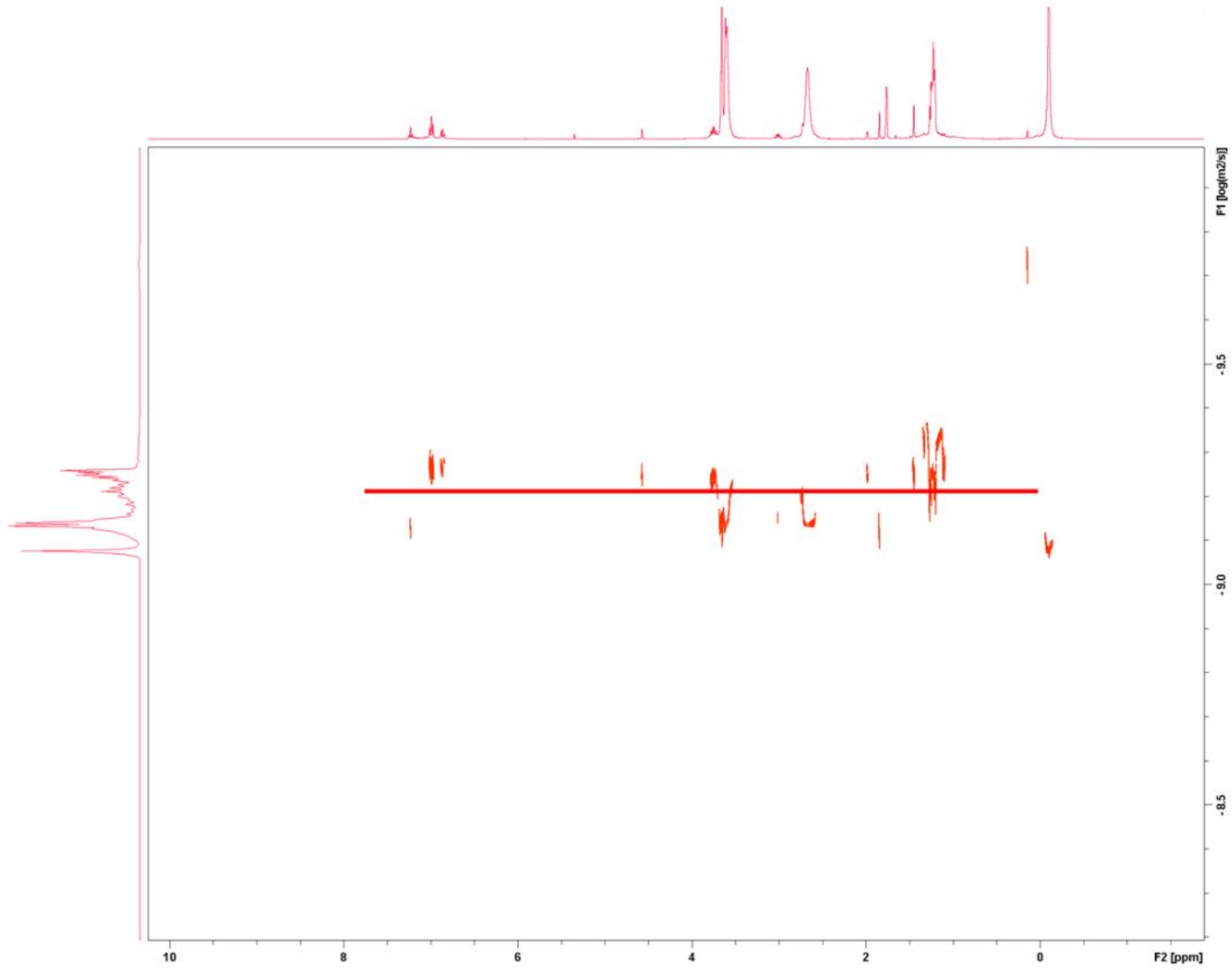

Figure S42. ^{1}H DOSY NMR 400 MHz of **2·Li(crypt-222)** in THF-d_8 at 298 K.

Table S2. $D_{x,norm}$ ($m^2 \cdot s^{-1}$) and MW values for species of **2·Li(crypt-222)** in THF-d_8 at 298 K.

Species	$D_{x,norm}$ (m^2 s^{-1})	*MW found (g/mol)*	*MW calc (g/mol)*	*MW error*	D_{hyd} *(Å)*	$D_{X\text{-}ray}$ *(Å)*
diffusing specie	6.82×10^{-10}	770[a]<br>668[b]	-	-	16	-
2···Li(crypt)<br>*contacted species*	-	-	884	13%[a]<br>30%[b]	-	17
2^{-}<br>*separated anion*	-	-	486	-	-	-
$Li(crypt)^{+}$<br>*separated cation*	-	-	384	-	-	-

[a] *Values from ECC-CS.*[11-12] [b] *Values from OEC (own external calibration method).*

Comments on the ^{1}H DOSY NMR spectrum of 2·Li(crypt-222) in THF-d_8 solution: Only one diffusing species is observed. The molecular weight determined using the calibration curves reported by Stalke,[11-12] resulted in a value of 770 g/mol (ECC + highly compact spheres). This molecular weight is corroborated by our regression method (OEC, own external calibration method), which yielded an MW of 668 g/mol. The predicted MW values from DOSY are in close proximity to the MW of the monomeric contacted species, with a slight error of 13% and 30% respectively. Both methodologies converge on the suggestion that the cationic [Li(crypt)]$^+$ and anionic **2**$^-$ units diffusing as a cohesive unit or a contacted pair, termed [**2**···Li(crypt)], in the THF-d_8 solution. It's important to note that the diffusion measurements might not rule out the possible inclusion of additional THF-d_8 solvent molecules jointly within this association, possibly forming complexes of the type [**2**···Li(crypt)(S)$_x$] (where solvent THF-d_8). Moreover, the hydrodynamic diameter (D_{hyd} ~ 15 Å), predicted from the diffusion coefficient, aligns with the estimated value from the molecular structure $D_{X\text{-}ray}$ ~ 17 Å (see Figure S38) taking into account the electrostatic interplays between the Ga and Cs atoms. These Ga···Li interactions of ~ 5.77 Å, while possibly evanescent in the solid state, seem to wield a greater influence in solution. They potentially act as stabilizing forces, shepherding the **2**···Li(crypt) into its predominant contacted pair configuration. The DOSY results also further supports the presence of contacted species in the THF-d_8 solution. In addition, the insights from DFT computational studies (detailed in Section S6) echo these observations. They underline the plausibility of these feeble electrostatic interactions within **2**···Li(crypt) and intimate the formation of weak contacted species, mirroring configurations captured in X-ray diffraction and in solution by DOSY NMR experiments.

S4.1.2 ^{1}H DOSY NMR spectroscopic study of 2·Na(crypt-222)

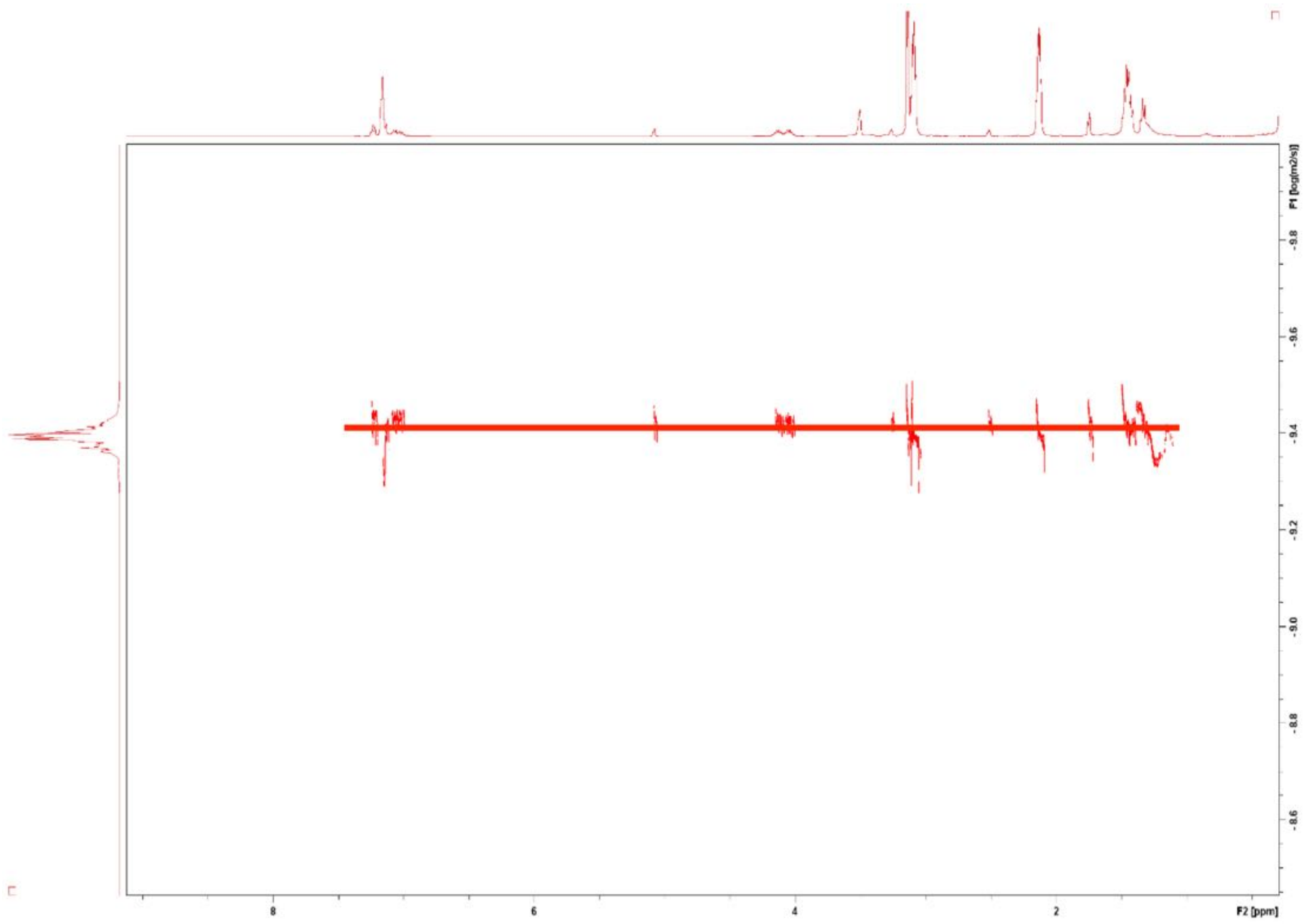

Figure S43. ^{1}H DOSY NMR 500 MHz of **2·Na(crypt-222)** in C_6D_6:THF-d_8 at 298 K.

Table S3. $D_{x,norm}$ ($m^2 \cdot s^{-1}$) and MW values for species of **2·Na(crypt-222)** in C_6D_6:THF-d_8 at 298 K.

Species	$D_{x,norm}$ ($m^2\, s^{-1}$)	*MW found (g/mol)*	*MW calc (g/mol)*	*MW error*	D_{hyd} *(Å)*	$D_{X\text{-}ray}$ *(Å)*
diffusing specie	3.74×10^{-10}	903^{a} 1057^{b}	-	-	18	-
2···Na(crypt) *contacted species*	-	-	886	2%^{a}, 23%^{b}	-	-
2^{-} *separated anion*	-	-	486	-	-	-
$Na(crypt)^{+}$ *separated cation*	-	-	399	-	-	-

^{a} Values from ECC-ED.[11-12] ^{b} Values from OEC (own external calibration method).

Comments on the ^{1}H DOSY NMR spectrum of [2][Na(crypt) in C_6D_6:THF-d_8 solution: A solitary diffusing species is identified in the C_6D_6/THF-d_8 solution from the DOSY data. Utilizing the calibration curves established by Stalke,[11-12] the molecular weight for this species is determined to be 903 g/mol (under the ECC model of expanded disks). Our in-house regression approach (OEC, own external calibration method) yielded a slightly higher molecular weight of 1057 g/mol. The predicted MW values from DOSY are in close proximity to the MW of the monomeric contacted species, with a slight error of 2% and 23% respectively. Both methodologies indicate that in solution, there's a strong likelihood of interaction between the cationic $[Na(crypt)]^+$ and anionic **2**$^-$ components, manifesting as a contacted pair, **2**⋯Na(crypt). Considering the observed diffusion rates, the incorporation of additional solvent molecules, specifically C_6D_6 and THF-d_8, into this association is conceivable, leading to potential species like [**2**⋯Na(crypt)$(C_6D_6)_n$(THF-$d_8)_m$]. Moreover, the hydrodynamic diameter (D_{hyd} ~ 18 Å), predicted from the diffusion coefficient, aligns with the other [**2**⋯M(crypt)] congeners $D_{X\text{-}ray}$ ~ 17-18 Å (see Figure S38) taking into account the electrostatic interplays between the Ga and the different alkali metals. These Ga⋯Na interactions seem to be prominent in solution. They potentially act as stabilizing forces, shepherding the **2**⋯Na(crypt) into its predominant contacted pair configuration. The DOSY results also further supports the presence of contacted species in the C_6D_6/THF-d_8 solution. In addition, the insights from DFT computational studies (detailed in Section S6) echo these observations. They underline the plausibility of these feeble electrostatic interactions within **2**⋯Na(crypt) and intimate the formation of weak contacted species, mirroring configurations captured by DOSY NMR experiments.

S4.1.3 ^{1}H DOSY NMR spectroscopic study of 2·K(crypt-222)

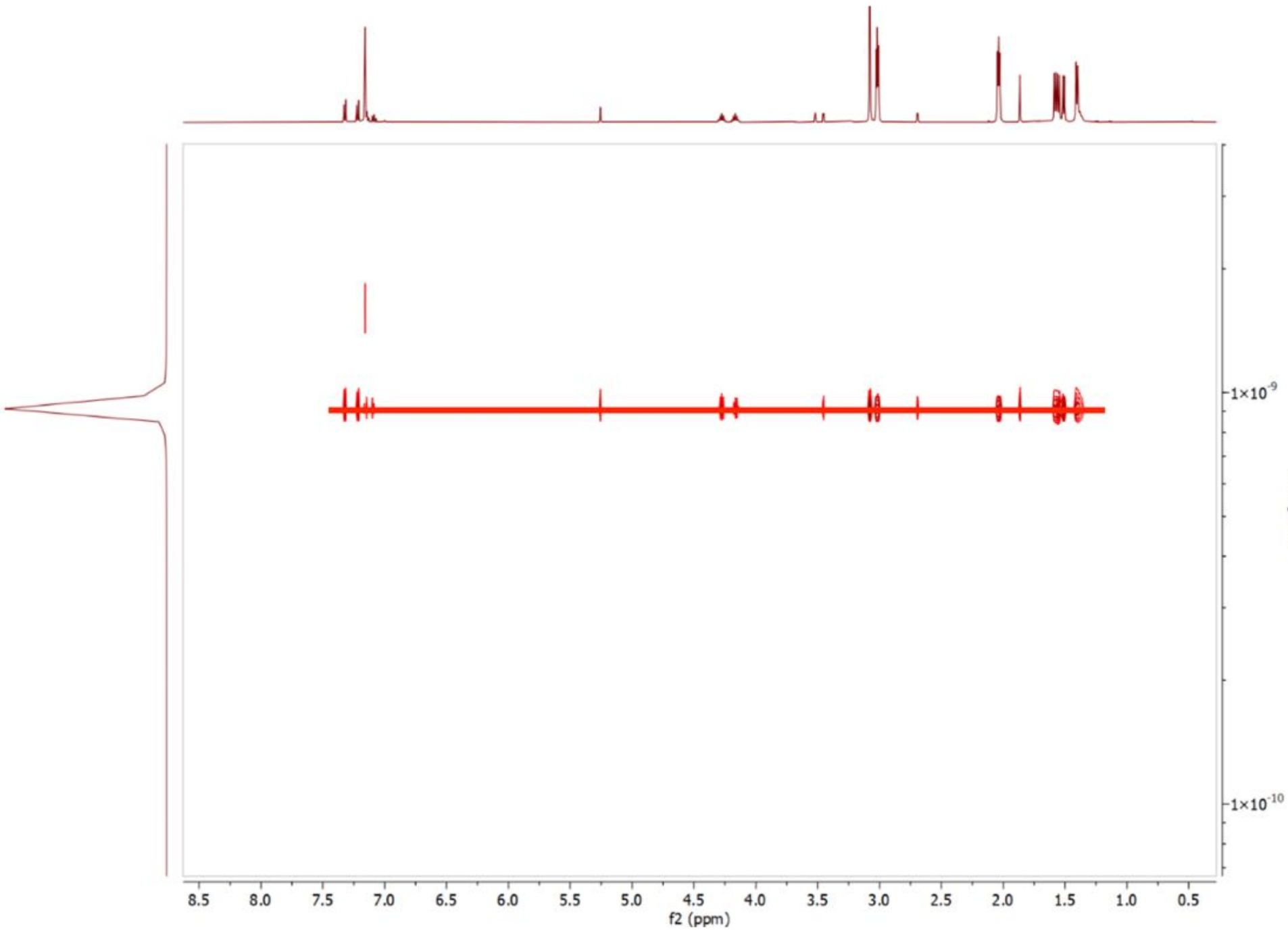

Figure S44. ^{1}H DOSY NMR 500 MHz of **2·K(crypt-222)** in C_6D_6:THF-d_8 at 298 K.

Table S4. $D_{x,norm}$ ($m^2 \cdot s^{-1}$) and MW values for species of **2·K(crypt-222)** in C_6D_6:THF-d_8 at 298 K.

Species	$D_{x,norm}$ ($m^2\,s^{-1}$)	*MW found (g/mol)*	*MW calc (g/mol)*	*MW error*	D_{hyd} *(Å)*	$D_{X\text{-}ray}$ *(Å)*
diffusing specie	5.14×10^{-10}	1012[a]<br>1110[b]	-	-	18	-
2···K(crypt)<br>*contacted species*	-	-	902	11%[a]<br>19%[b]	-	18
2^{-}<br>*separated anion*	-	-	486	-	-	-
$K(crypt)^{+}$<br>*separated cation*	-	-	416	-	-	-

[a] *Values from ECC-DSE.*[11-12] [b] *Values from OEC (own external calibration method).*

Comments on the ^{1}H DOSY NMR spectrum of 2·K(crypt-222) in C_6D_6:THF-d$_8$ solution: A single diffusing species is identified in the C_6D_6/THF-d_8 solution from the DOSY data. Utilizing the calibration curves established by Stalke,[11-12] the molecular weight for this species is determined to be 1012 g/mol (under the ECC model of diffused spheres and ellipsoids). Our in-house regression approach (OEC, own external calibration method) yielded a slightly higher molecular weight of 1110 g/mol. The predicted MW values from DOSY are in close proximity to the MW of the monomeric contacted species, with a slight error of 11% and 19% respectively. Both methodologies indicate that in solution, there's a strong likelihood of interaction between the cationic $[K(crypt)]^+$ and anionic $\mathbf{2}^-$ components, manifesting as a contacted pair, **2···K(crypt)**. Considering the observed diffusion rates, the incorporation of additional solvent molecules, specifically C_6D_6 and THF-d_8, into this association is conceivable, leading to potential species like [**2···K(crypt)(C_6D_6)$_n$(THF-d_8)$_m$**]. Moreover, the hydrodynamic diameter (D_{hyd} ~ 18 Å), predicted from the diffusion coefficient, aligns with the estimated value from the molecular structure $D_{X\text{-}ray}$ ~ 18 Å (see Figure S38) taking into account the electrostatic interplays between the Ga and K atoms. These Ga···K interactions of ~ 5.15 Å, while possibly evanescent in the solid state, seem to wield a greater influence in solution. They potentially act as stabilizing forces, shepherding the **2**···K(crypt) into its predominant contacted pair configuration. The DOSY results also further supports the presence of contacted species in the C_6D_6/THF-d_8 solution. In addition, the insights from DFT computational studies (detailed in Section S6) echo these observations. They underline the plausibility of these feeble electrostatic interactions within **2···K(crypt)** and intimate the formation of weak contacted species, mirroring configurations captured in X-ray diffraction and in solution by DOSY NMR experiments.

S4.1.4 ^{1}H DOSY NMR spectroscopic study of 2·Cs(crypt-222)

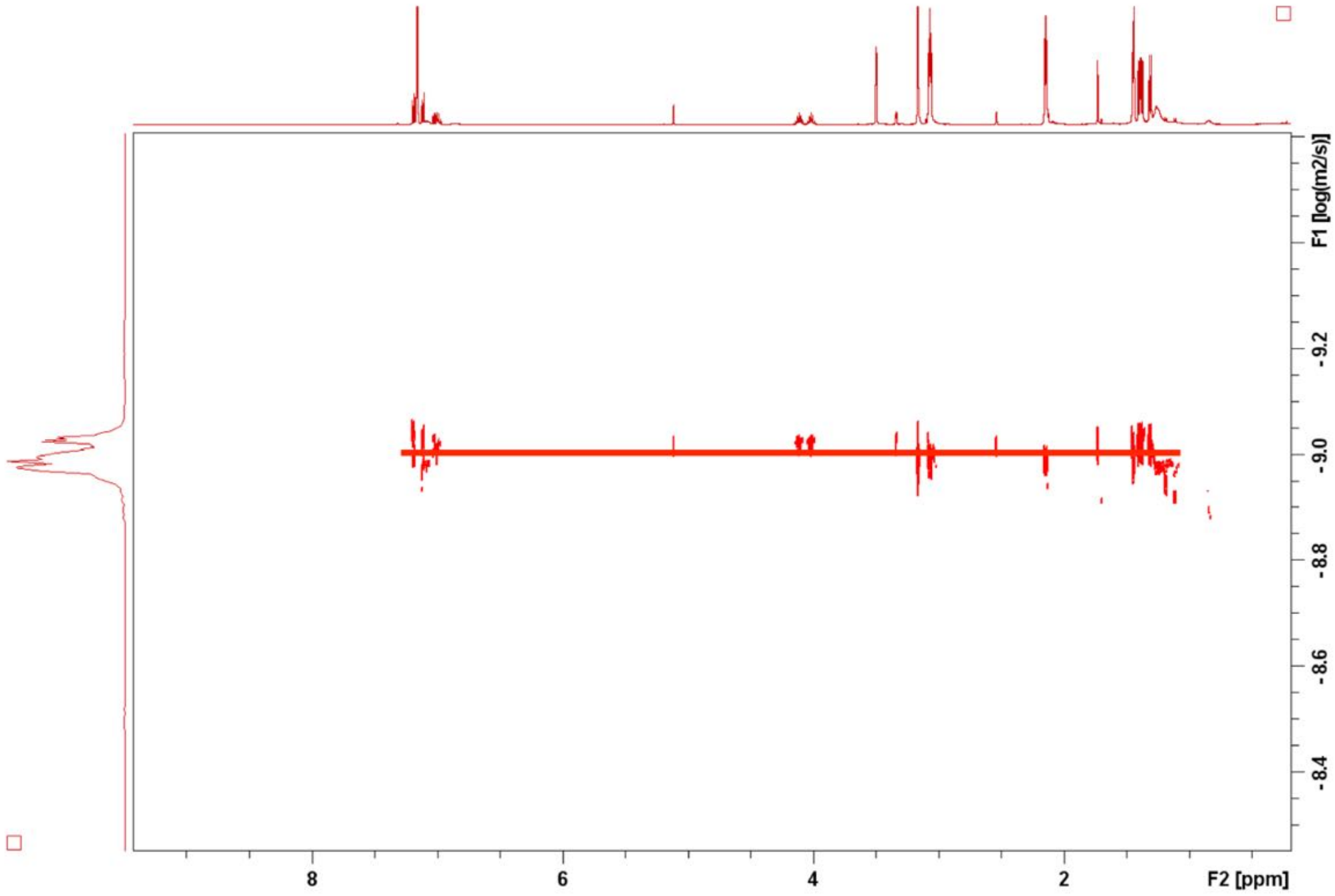

Figure S45. ^{1}H DOSY NMR 400 MHz of **2·Cs(crypt-222)** in C_6D_6:THF-d_8 at 298 K.

Table S5. $D_{x,norm}$ ($m^2·s^{-1}$) and MW values for species of **2·Cs(crypt-222)** in C_6D_6:THF-d_8 at 298 K.

Species	$D_{x,norm}$ ($m^2 s^{-1}$)	*MW found (g/mol)*	*MW calc (g/mol)*	*MW error*	D_{hyd} *(Å)*	$D_{X\text{-}ray}$ *(Å)*
diffusing specie	5.19×10^{-10}	994[a] 1135[b]	-	-	18	-
2···Cs(crypt) *contacted species*	-	-	995	< 1%[a], 11%[b]	-	19
2^{-} *separated species*	-	-	486	-	-	-
$Cs(crypt)^{+}$ *separated species*	-	-	509	-	-	-

[a] *Values from ECC-DSE.*[11-12] [b] *Values from OEC (own external calibration method).*

Comments on the ^{1}H DOSY NMR spectrum of 2·Cs(crypt-222) in C_6D_6:THF-d_8 solution: A single diffusing species is identified in the C_6D_6/THF-d_8 solution from the DOSY data. Utilizing the calibration curves established by Stalke,[11-12] the molecular weight for this species is determined to be 994 g/mol (under the ECC model of diffused spheres and ellipsoids). Our in-house regression approach (OEC, own external calibration method) yielded a slightly higher molecular weight of 1135 g/mol. The predicted MW values from DOSY are in close proximity to the MW of the monomeric contacted species, with a slight error of < 1% and 12% respectively. Both methodologies indicate that in solution, there's a strong likelihood of interaction between the cationic $[Cs(crypt)]^+$ and anionic **2**$^-$ components, manifesting as a contacted pair, **2···Cs(crypt)**. Considering the observed diffusion rates, the incorporation of additional solvent molecules, specifically C_6D_6 and THF-d_8, into this association is conceivable, leading to potential species like [**2···Cs(crypt)(C_6D_6)$_n$(THF-d_8)$_m$**]. Moreover, the hydrodynamic diameter (D_{hyd} ~ 19 Å), predicted from the diffusion coefficient, aligns with the estimated value from the molecular structure $D_{X\text{-}ray}$ ~ 18 Å (see Figure S38) taking into account the electrostatic interplays between the Ga and Cs atoms. These Ga···Cs interactions of ~ 5.13 Å, while possibly evanescent in the solid state, seem to wield a greater influence in solution. They potentially act as stabilizing forces, shepherding the **2···Cs(crypt)** into its predominant contacted pair configuration. The DOSY results also further supports the presence of contacted species in the C_6D_6/THF-d_8 solution. In addition, the insights from DFT computational studies (detailed in Section S6) echo these observations. They underline the plausibility of these feeble electrostatic interactions within **2**···Cs(crypt) and intimate the formation of weak contacted species, mirroring configurations captured in X-ray diffraction and in solution by DOSY NMR experiments.

S4.1.5 1H DOSY NMR spectroscopic study of 2·Na(18-c-6)

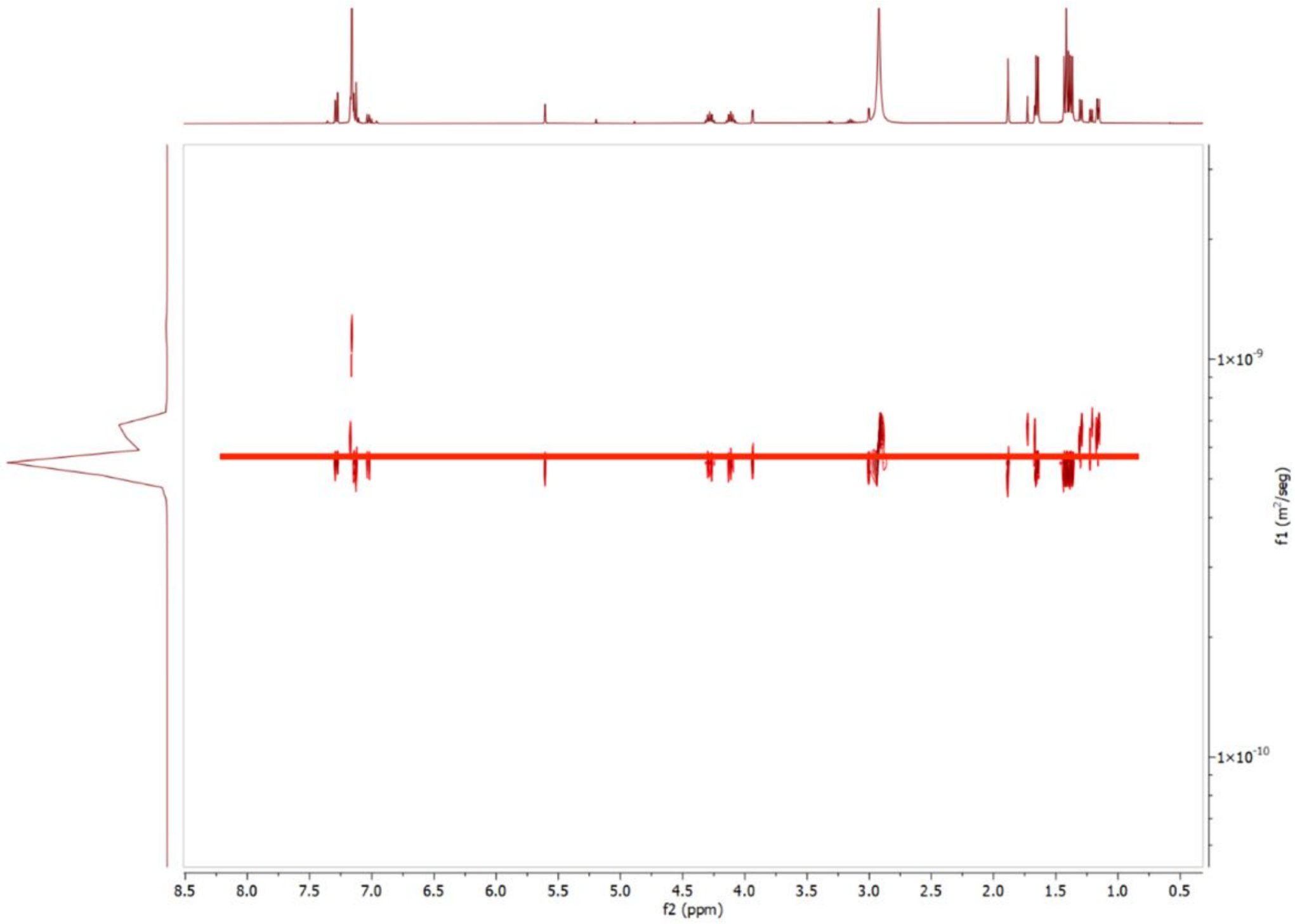

Figure S46. ^{1}H DOSY NMR 400 MHz of **2·Na(18-c-6)** in C_6D_6 at 298 K.

Table S6. $D_{x,norm}$ ($m^2·s^{-1}$) and MW values for species of **2·Na(18-c-6)** in C_6D_6 at 298 K.

Species	$D_{x,norm}$ ($m^2 s^{-1}$)	*MW found (g/mol)*	*MW calc (g/mol)*	*MW error*	*D_{hyd} (Å)*	*$D_{X\text{-}ray}$ (Å)*
diffusing specie	5.95×10^{-10}	665[a] 648[b]	-	-	14	-
2·Na(18-c-6) *contacted species*	-	-	774	16%[a] 19%[b]	-	14
2^{-} *separated species*	-	-	486	-	-	-
$Na(18\text{-}c\text{-}6)^+$ *separated species*	-	-	287	-	-	-

[a] *Values from ECC-DSE.*[11-12] [b] *Values from OEC (own external calibration method).*

Comments on the ^{1}H DOSY NMR spectrum of 2·Na(18-c-6) in C_6D_6 solution: A single diffusing species is identified in the C_6D_6 solution from the DOSY data. Utilizing the calibration curves established by Stalke,[11-12] the molecular weight for this species is determined to be 665 g/mol (under the ECC model of diffused spheres and ellipsoids). Our in-house regression approach (OEC, own external calibration method) yielded a slightly higher molecular weight of 648 g/mol. The predicted MW values from DOSY are in close proximity to the MW of the monomeric contacted species, with a slight error of < 16% and 19% respectively. Both methodologies indicate that in solution, there's a strong likelihood of interaction between the cationic $[Na(18\text{-}c\text{-}6)]^+$ and anionic **2**$^-$ components, manifesting as a contacted pair, **2·Na(18-c-6)**. Considering the observed diffusion rates, the incorporation of additional solvent molecules, specifically C_6D_6, into this association is conceivable, leading to potential species such as [**2·Na(18-c-6)(C_6D_6)$_n$**]. Moreover, the hydrodynamic diameter (D_{hyd} ~ 14 Å), predicted from the diffusion coefficient, aligns with the estimated value from the molecular structure $D_{X\text{-}ray}$ ~ 14 Å (see Figure S39) taking into account the electrostatic interplays between the Ga and Na atoms. These Ga–Na interactions, measuring approximately 3.33 Å in the solid state, appear to exert a more significant influence in solution. They potentially act as stabilizing forces, shepherding the **2·Na(18-c-6)** into its predominant contacted pair configuration. The DOSY results also further supports the presence of contacted species in the C_6D_6 solution. In addition, the insights from DFT computational studies (in Section S6) echo these observations. They underline the plausibility of these feeble electrostatic interactions within **2·Na(18-c-6)** and intimate the formation of weak contacted species, mirroring configurations captured in X-ray diffraction and in solution by DOSY NMR experiments.

S4.1.6 ^{1}H DOSY NMR spectroscopic study of 2·K(18-c-6)

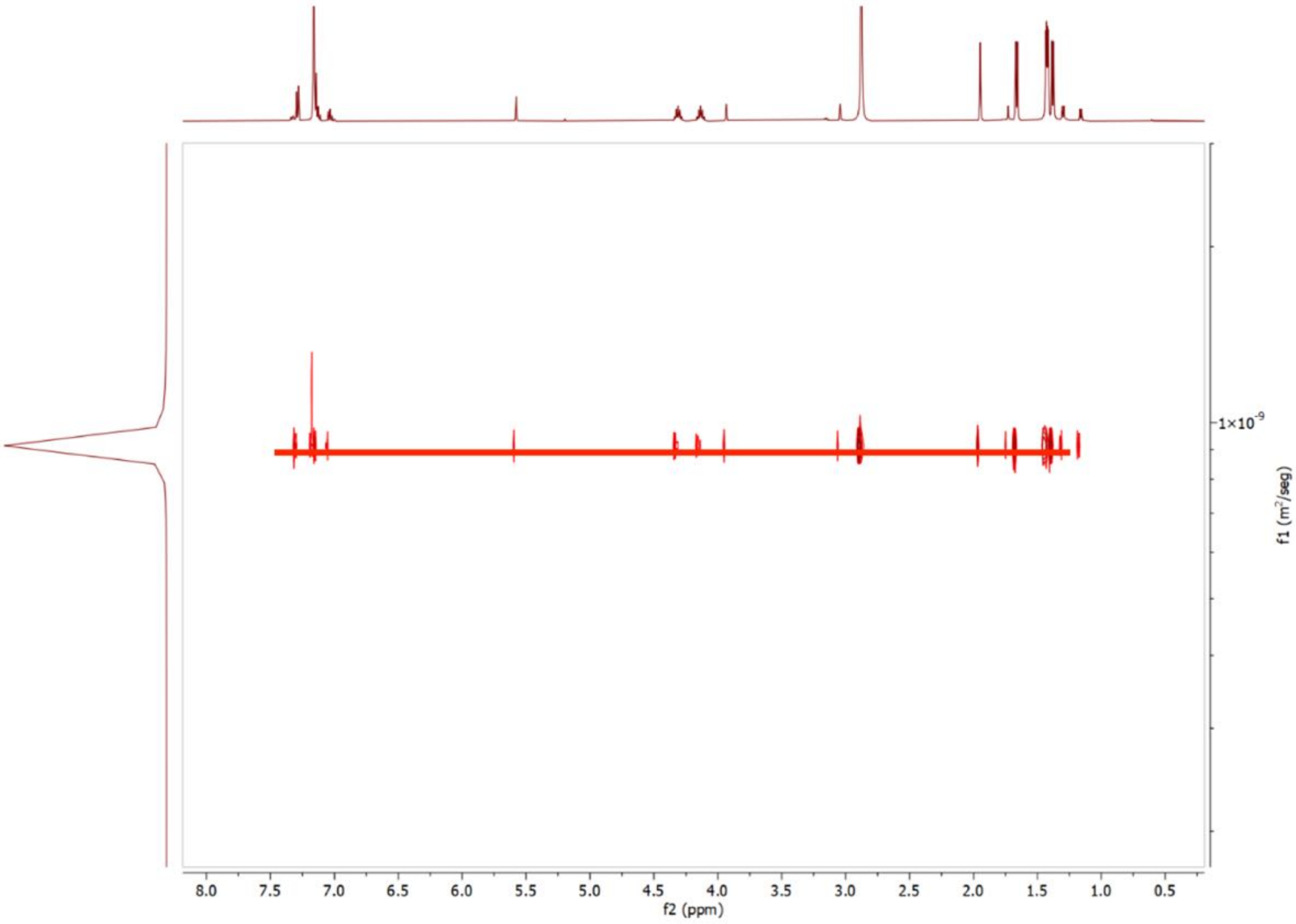

Figure S47. ^{1}H DOSY NMR 400 MHz of **2·K(18-c-6)** in C_6D_6 at 298 K.

Table S7. $D_{x,norm}$ ($m^2·s^{-1}$) and MW values for species of **2·K(18-c-6)** in C_6D_6 at 298 K.

Species	$D_{x,norm}$ (m^2 s^{-1})	*MW found (g/mol)*	*MW calc (g/mol)*	*MW error*	D_{hyd} *(Å)*	$D_{X\text{-}ray}$ *(Å)*
diffusing specie	5.09×10^{-10}	855[a] 1132[b]	-	**-**	14	-
2·K(18-c-6) *contacted species*	-	-	789	8%[a] 30%[b]	-	14
2^{-} *separated species*	-	-	486		-	-
$K(18\text{-}c\text{-}6)^{+}$ *separated species*	-	-	303	**-**	-	-

[a] *Values from ECC-DSE.*[11-12] [b] *Values from OEC (own external calibration method).*

Comments on the ^{1}H DOSY NMR spectrum of 2·K(18-c-6) in C_6D_6 solution: A single diffusing species is identified in the C_6D_6 solution from the DOSY data. Utilizing the calibration curves established by Stalke,[11-12] the molecular weight for this species is determined to be 855 g/mol (under the ECC model of diffused spheres and ellipsoids). Our in-house regression approach (OEC, own external calibration method) yielded a slightly higher molecular weight of 1132 g/mol. The predicted MW values from DOSY are in close proximity to the MW of the monomeric contacted species, with a slight error of < 8% and 30% respectively. Both methodologies indicate that in solution, there's a strong likelihood of interaction between the cationic $[K(18\text{-c-}6)]^+$ and anionic **2**$^-$ components, manifesting as a contacted pair, **2·K(18-c-6)**. Considering the observed diffusion rates, the incorporation of additional solvent molecules, specifically C_6D_6, into this association is conceivable, leading to potential species like [**2·K(18-c-6)(C_6D_6)$_n$**]. Moreover, the hydrodynamic diameter (D_{hyd} ~ 14 Å), predicted from the diffusion coefficient, aligns with the estimated value from the molecular structure $D_{X\text{-}ray}$ ~ 14 Å (see **Figure S39**) taking into account the electrostatic interplays between the Ga and K atoms. These Ga–K interactions, approximately 3.49 Å, in the solid state and appear to have a greater impact in solution. They potentially act as stabilizing forces, shepherding the **2·K(18-c-6)** into its predominant contacted pair configuration. The DOSY results also further supports the presence of contacted species in the C_6D_6 solution. In addition, the insights from DFT computational studies (detailed in Section S6) echo these observations. They underline the plausibility of these feeble electrostatic interactions within **2·K(18-c-6)** and intimate the formation of weak contacted species, mirroring configurations captured in X-ray diffraction and in solution by DOSY NMR experiments.

S4.1.7 ^{1}H DOSY NMR spectroscopic study of 2·Cs(18-c-6)

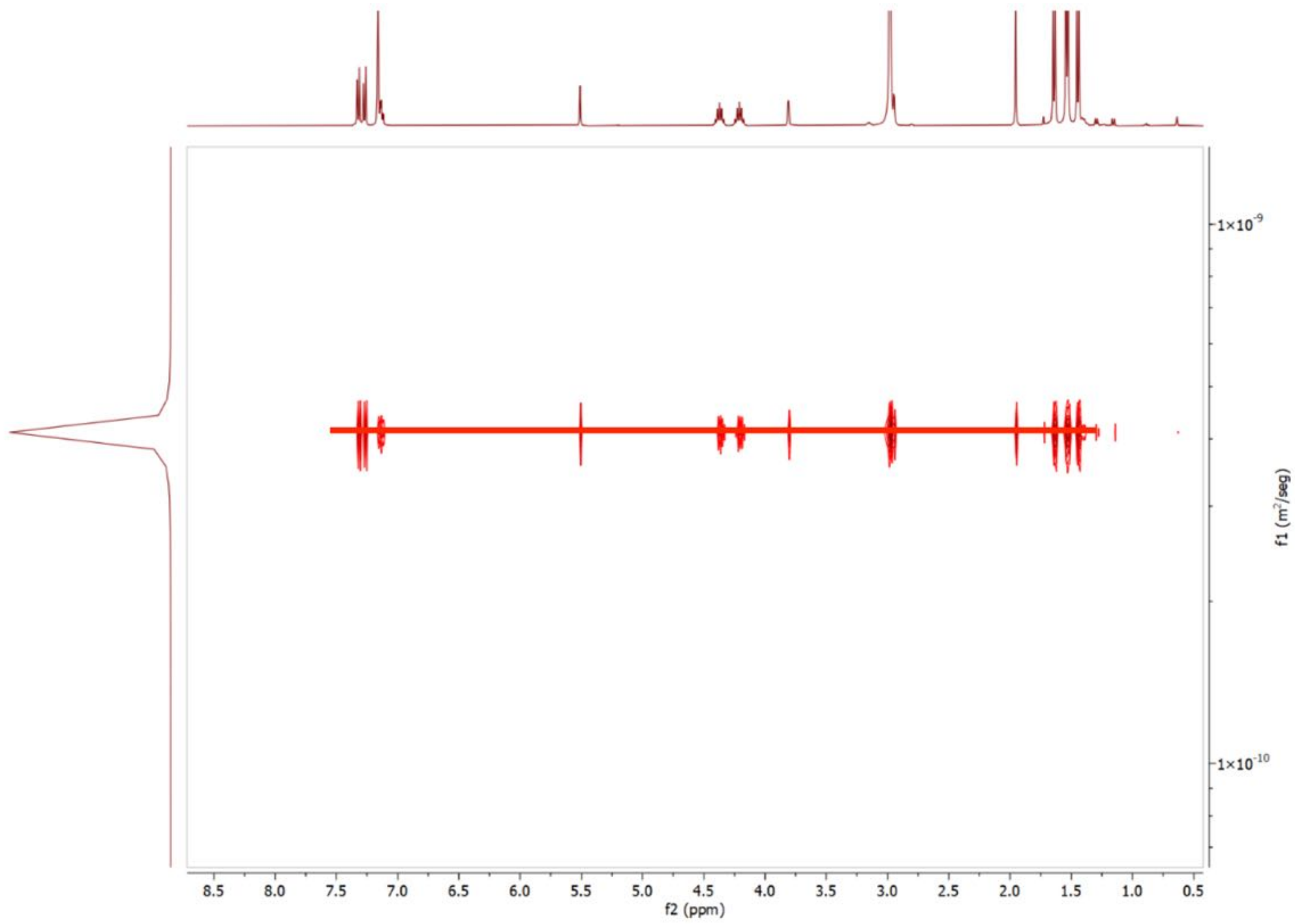

Figure S48. ^{1}H DOSY NMR 400 MHz of **2·Cs(18-c-6)** in C_6D_6 at 298 K.

Table S8. $D_{x,norm}$ ($m^2 \cdot s^{-1}$) and MW values for species of **2·Cs(18-c-6)** in C_6D_6 at 298 K.

Species	$D_{x,norm}$ (m^2 s^{-1})	*MW found (g/mol)*	*MW calc (g/mol)*	*MW error*	*D_{hyd} (Å)*	*D_{X-ray} (Å)*
diffusing specie	4.49×10^{-10}	807[a] 1061[b]	-	-	15	-
2·Cs(18-c-6) *contacted species*	-	-	883	10%[a] 17%[b]	-	15
2^- *separated species*	-	-	486		-	-
$Cs(18\text{-}c\text{-}6)^+$ *separated species*	-	-	397		-	-

[a] Values from ECC-ED.[11-12] [b] Values from OEC (own external calibration method).

Comments on the ^{1}H DOSY NMR spectrum of 2·Cs(18-c-6) in C_6D_6 solution: A single diffusing species is identified in the C_6D_6 solution from the DOSY data. Utilizing the calibration curves established by Stalke,[11-12] the molecular weight for this species is determined to be 807 g/mol (under the ECC model of expanded disks). Our in-house regression approach (OEC, own external calibration method) yielded a slightly higher molecular weight of 1061 g/mol. The predicted MW values from DOSY are in close proximity to the MW of the monomeric contacted species, with a slight error of < 10% and 17% respectively. Both methodologies indicate that in solution, there's a strong likelihood of interaction between the cationic [Cs(18-c-6)]$^{+}$ and anionic **2**$^{-}$ components, manifesting as a contacted pair, **2·Cs(18-c-6)**. Considering the observed diffusion rates, the incorporation of additional solvent molecules, specifically C_6D_6, into this association is conceivable, leading to potential species like [**2·Cs(18-c-6)(C_6D_6)$_n$**]. Moreover, the hydrodynamic diameter (D_{hyd} ~ 15 Å), predicted from the diffusion coefficient, aligns with the estimated value from the molecular structure $D_{X\text{-}ray}$ ~ 15 Å (see **Figure S39**) taking into account the electrostatic interplays between the Ga and Cs atoms. These Ga–Cs interactions of approximately 3.71 Å in the solid state and appear to have a more significant effect in solution. They potentially act as stabilizing forces, shepherding the **2·Cs(18-c-6)** into its predominant contacted pair configuration. The DOSY results also further supports the presence of contacted species in the C_6D_6. In addition, the insights from DFT computational studies (in Section S6) echo these observations. They underline the plausibility of these feeble electrostatic interactions within **2·Cs(crypt-222)** and intimate the formation of weak contacted species, mirroring configurations captured in X-ray diffraction and in solution by DOSY NMR experiments.

S4.1.8 ^{1}H DOSY NMR spectroscopic study of **2·Cs(18-c-6)$_2$**

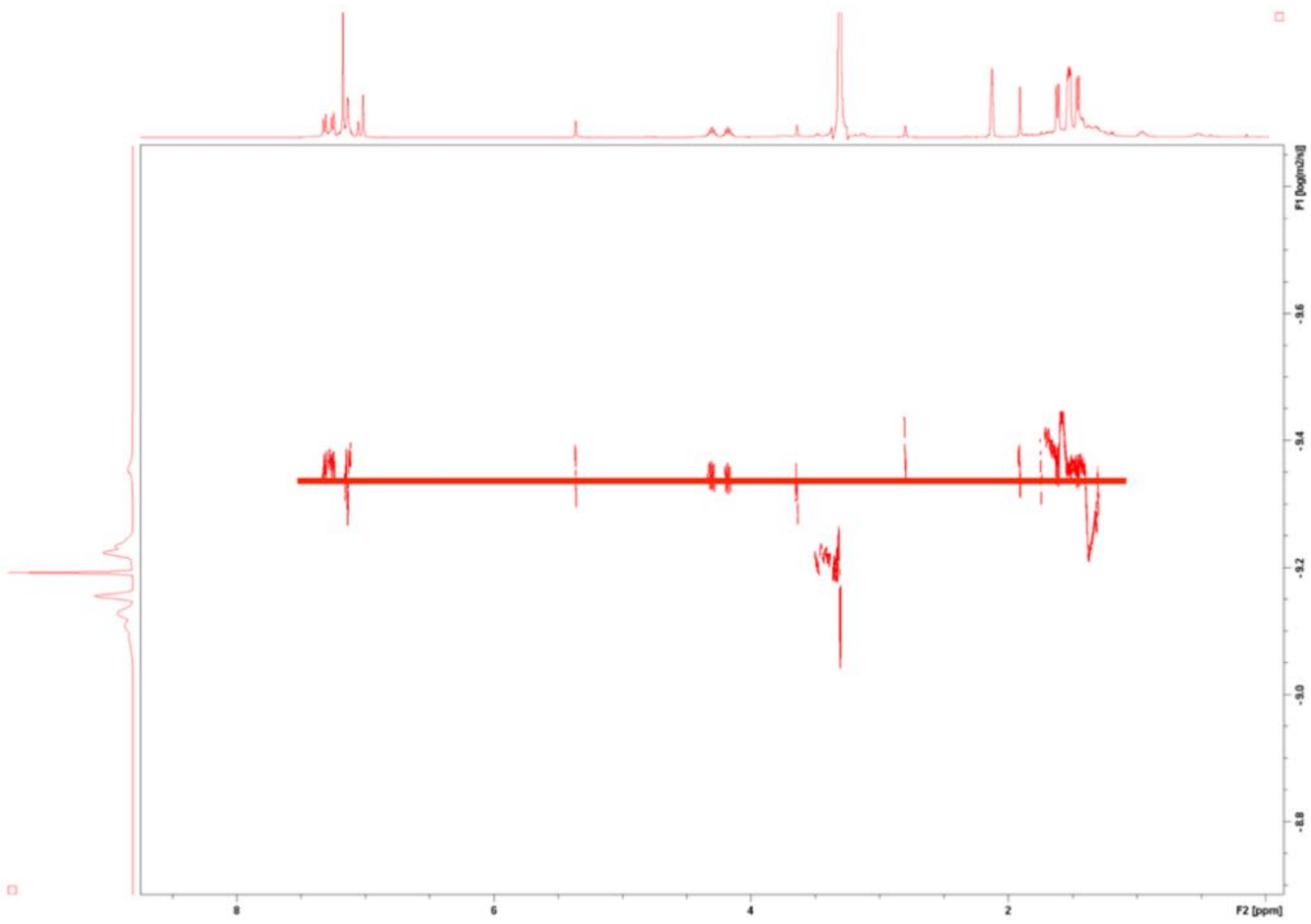

Figure S49. ^{1}H DOSY NMR 400 MHz of **2·Cs(18-c-6)$_2$** in Tol-d$_8$ at 298 K.

Table S9. $D_{x,norm}$ ($m^2·s^{-1}$) and MW values for species of **2·Cs(18-c-6)$_2$** in Tol-d$_8$ at 298 K.

Species	$D_{x,norm}$ (m^2 s^{-1})	*MW found (g/mol)*	*MW calc (g/mol)*	*MW error*	D_{hyd} *(Å)*	$D_{X\text{-}ray}$ *(Å)*
diffusing species 1	4.90×10^{-10}	658[a] 921[b]	-	-	15	-
diffusing species 2	7.18×10^{-10}	571[c] 557[b]			11	-
2···Cs(18-c-6)$_2$ *contacted species*	-	-	1148		-	20
2$^{–}$ *separated species*	-	-	486	26%[a] 47%[b]	-	14
Cs(18-c-6)$_2$$^{+}$ *separated species*	-	-	662	16%[c] 19%[b]	-	12

[a] Values from ECC-DSE.[11-12] [b] Values from OEC (own external calibration method). [c] Values from ECC-CS.[11-12]

Comments on the ^{1}H DOSY NMR spectrum of 2·Cs(18-c-6)$_2$ in Tol-d_8 solution: In the Tol-d_8 solution, two species are observed to be diffusing. For the **2**$^-$ signals, the determined molecular weight, using calibration curves reported by Stalke,[11-12] is 658 g/mol (under the ECC model of diffused spheres and ellipsoids). Our regression method (OEC, own external calibration method) yields a comparable MW of 921 g/mol. For the 18-c-6 signals, the determined molecular weight, applying Stalke's calibration curves,[11-12] is 571 g/mol (ECC + highly compact spheres), and 557 g/mol using our regression method (OEC). The predicted MW values from DOSY are smaller than that for the aggregated ion pair [**2**···Cs(18-c-6)$_2$]. This data suggests an aggregation process in the Tol-d_8 solution favouring separated species **2**$^-$ and [Cs(18-c-6)$_2$]$^+$. Further support for this observation comes from the predicted hydrodynamic diameters, D_{hyd} ~ 14 Å for the anionic **[2]**$^-$ and D_{hyd} ~ 12 Å for the cationic [Cs(18-c-6)$_2$]$^+$, obtained from the diffusion coefficients. Both D_{hyd} values are close to the isolated diameters $D_{X\text{-}ray}$ (~ 14 for **2**$^-$ and ~12 Å for [Cs(18-crown-6)$_2$]$^+$) and smaller that the contacted ion pair $D_{X\text{-}ray}$ (~ 20 Å) estimated from the molecular structure (see **Figure S39** and **Figure S41**). These hydrodynamic diameter analyses also point towards the existence of separated-ion-pairs in Tol-d_8 solution and is further backed up by the DFT analyses (in Section S6).

S4.1.9 ^{1}H DOSY NMR spectroscopic study of [(2·K)$_2$(KHMDS)$_2$]$_\infty$

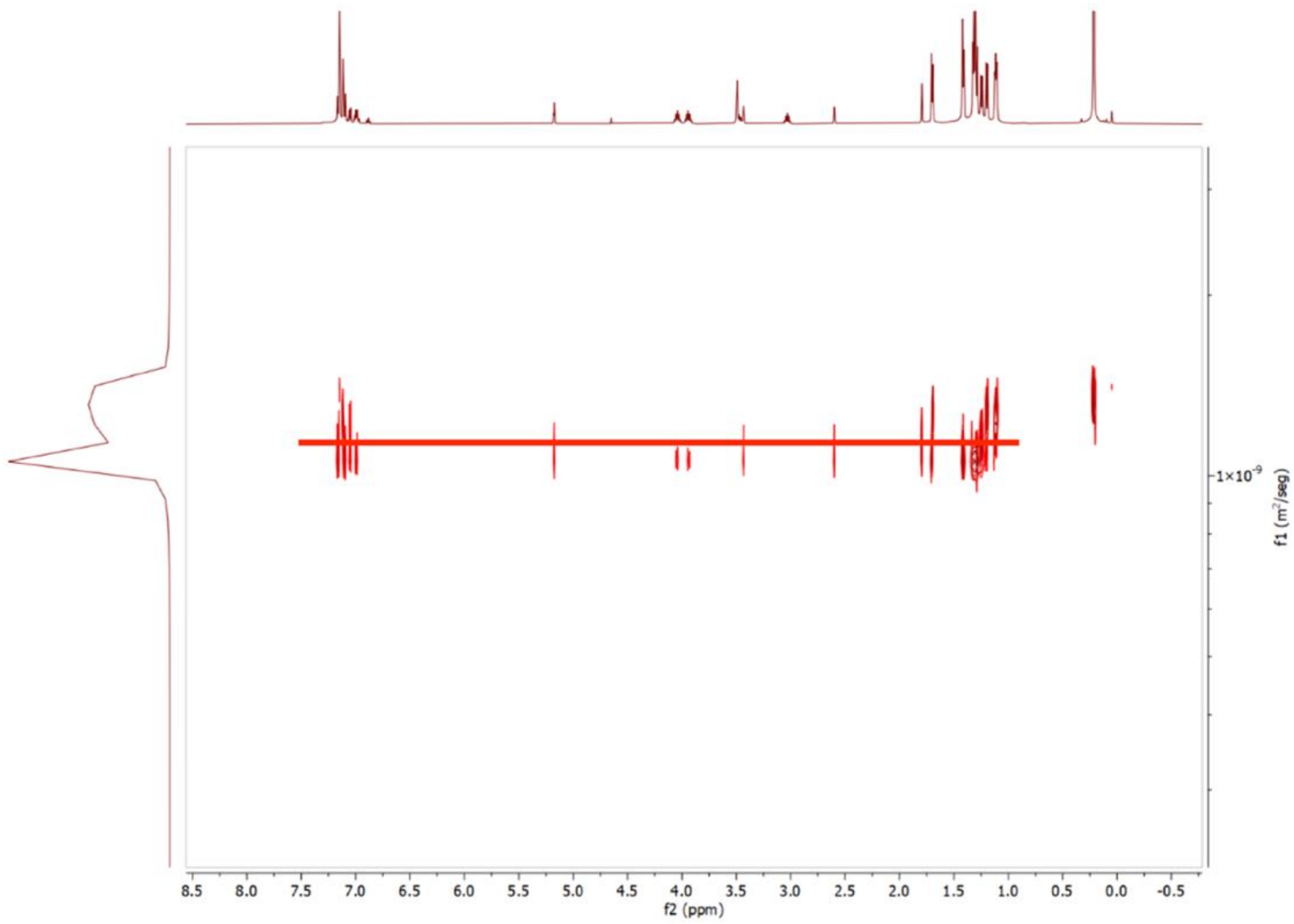

Figure S50. ^{1}H DOSY NMR 500 MHz of [**(2·K)$_2$(KHMDS)$_2$**]$_\infty$ in C_6D_6:THF-d_8 at 298 K.

Table S10. $D_{x,norm}$ ($m^2 \cdot s^{-1}$) and MW values for species of [**(2·K)$_2$(KHMDS)$_2$**]$_\infty$ (C_6D_6:THF-d_8, 298 K).

Species	$D_{x,norm}$ ($m^2\ s^{-1}$)	*MW found (g/mol)*	*MW calc (g/mol)*	*MW error*	*D_{hyd} (Å)*	*$D_{X\text{-}ray}$ (Å)*
diffusing specie 1	5.92×10^{-10}	981[a]<br>845[b]	-	-	17	-
diffusing specie 2	7.72×10^{-10}	572[a]<br>490[b]	-	-	10	-
[(2·K)$_2$(KHMDS)$_2$]	-	-	1445	-	-	22
(2·K)$_2$	-	-	1051	7%[a], 24%[b]	-	17
(KHMDS)$_2$			399	-		10
(KHMDS)$_2$(THF-d_8)		--	471	18%[a], 4%[b]	-	-
(KHMDS)$_2$(THF-d_8)$_2$		--	551	4%[a], 11%[b]	-	-

[a] *Values from ECC-CS.*[11-12] [b] *Values from OEC (own external calibration method).*

Comments on the ^{1}H DOSY NMR spectrum of [(2·K)$_2$(KHMDS)$_2$]$_\infty$ in C_6D_6:THF-d_8 solution: The DOSY measurements in C_6D_6/THF-d_8 solution unveil two diffusing species: the dimeric assembly, (**2·K**)$_2$ and its counterpart, (KHMDS)$_2$. Analyzing the (**2·K**)$_2$ assembly, the molecular weight derived from the calibration curves of Stalke,[11-12] identifies it as being 981 g/mol (under the ECC model of compacted spheres). Confirming this, our regression methodology (OEC, own external calibration method) yields a molecular weight of 845 g/mol. The variance seen between these values and the calculated molecular weight of (**2**···K)$_2$ falls within a range of 7-24%. Turning to the (KHMDS)$_2$ assembly, its molecular weight is determined as 572 g/mol using Stalke's calibration curves and 490 g/mol via our OEC method. These results are close to the expected molecular weight of a THF-solvated dimer (KHMDS)$_2$(THF-d_8)$_2$, deviating by only 4-11%. The DOSY data imparts a clear picture: in solution, the aggregate unit (**2·K**)$_2$(**KHMDS**)$_2$ break apart into individual dimeric units (**2·K**)$_2$ and (KHMDS)$_2$. Likewise, the dimeric (**2·K**)$_2$ entity maintains its integrity, showcasing no evident dissolution into monomeric units. Further support for this observation comes from the predicted hydrodynamic diameters, D_{hyd} ~ 17 Å for the dimeric (**2·K**)$_2$ unit and D_{hyd} ~ 10 Å for (KHMDS)$_2$, obtained from the diffusion coefficients. Both D_{hyd} values are identical to the isolated diameters $D_{X\text{-}ray}$ (~ 17 for (**2·K**)$_2$ and ~ 10 Å for (KHMDS)$_2$) estimated from the molecular structure (see **Figure S41**). These hydrodynamic diameter analyses also point towards the existence of dimeric (**2·K**)$_2$ retaining their structures in solution and is further backed up by DFT analyses.

S4.1.10 ^{1}H DOSY NMR spectroscopic study of $[(\mathbf{2}\cdot\mathbf{Cs})_2(\mathbf{CsHMDS})_2]_\infty$

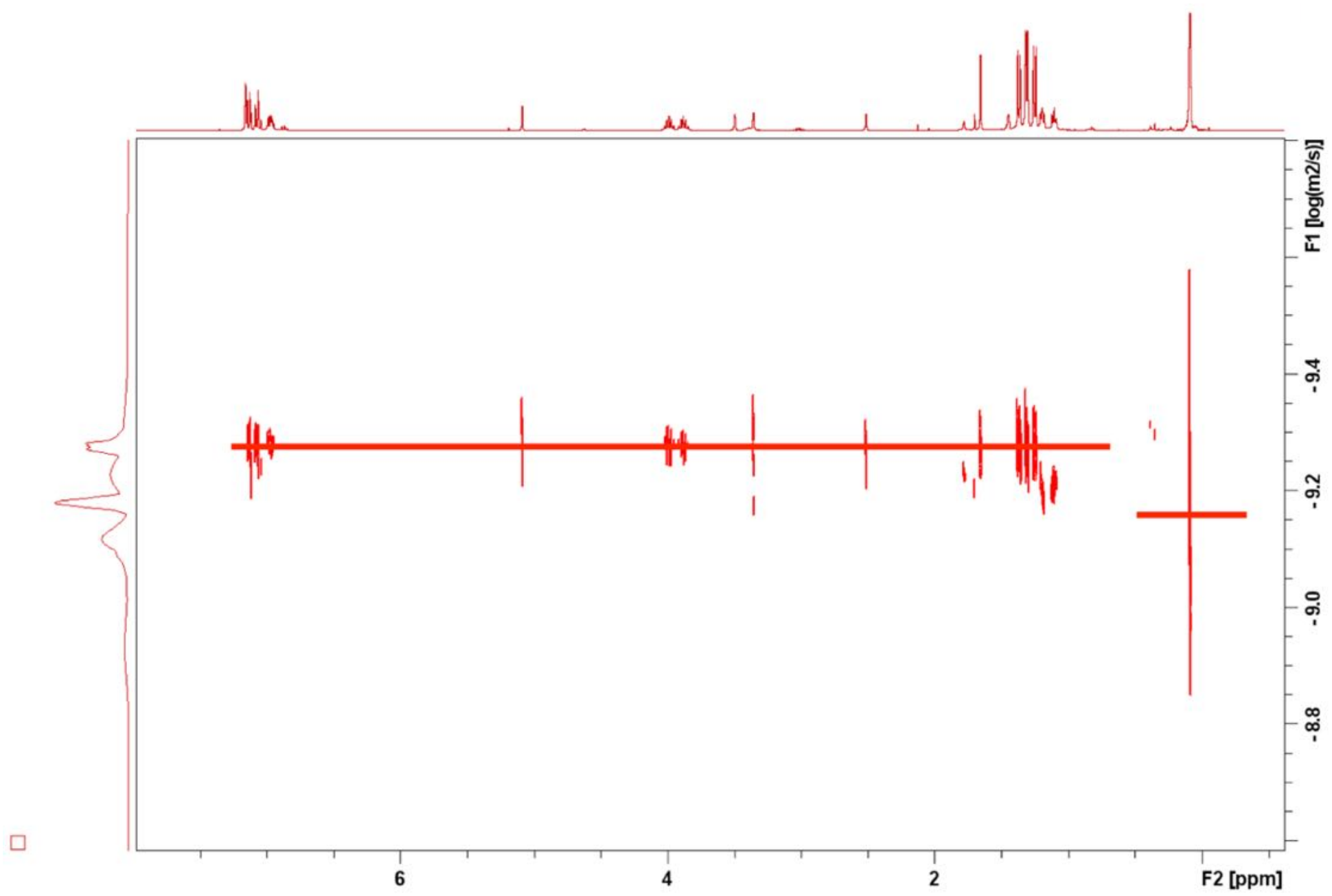

Figure S51. ^{1}H DOSY NMR 400 MHz of $[(\mathbf{2}\cdot\mathbf{Cs})_2(\mathbf{CsHMDS})_2]_\infty$ in C_6D_6:THF-d_8 at 298 K.

Table S11. $D_{x,norm}$ ($m^2\cdot s^{-1}$) and MW values for $[(\mathbf{2}\cdot\mathbf{Cs})_2(\mathbf{CsHMDS})_2]_\infty$ in C_6D_6:THF-d_8 at 298 K.

Species	$D_{x,norm}$ ($m^2 s^{-1}$)	*MW found (g/mol)*	*MW calc (g/mol)*	*MW error*	D_{hyd} *(Å)*	$D_{X\text{-}ray}$ *(Å)*
diffusing specie 1	5.81×10^{-10}	1067[a] 956[b]	-	-	17	-
diffusing specie 2	7.20×10^{-10}	690[a] 624[b]	-	-	12	-
$[(2\cdot Cs)_2(CsHMDS)_2]$	-	-	1825	-	-	24
$[(2\cdot Cs)_2]$	-	-	1239	16%[a], 26%[b]	-	17
$(CsHMDS)_2$			587	-	-	12
$(CsHMDS)_2$(THF-d_8)	-	-	659	5%[a], 5%[b]	-	-
$(CsHMDS)_2$(THF-d_8)$_2$	-	-	731	6%[a], 15%[b]	-	-

[a] *Values from ECC-CS.*[11-12] [b] *Values from OEC (own external calibration method).*

Comments on the ^{1}H DOSY NMR spectrum of [(2·Cs)$_2$(CsHMDS)$_2$]$_\infty$ in C_6D_6:THF-d_8 solution: The DOSY measurements in C_6D_6/THF-d_8 solution unveil two diffusing species: the dimeric assembly, (**2·Cs**)$_2$ and its counterpart, (CsHMDS)$_2$. Analyzing the (**2·Cs**)$_2$ assembly, the molecular weight derived from the calibration curves of Stalke,[11-12] identifies it as being 1067 g/mol (under the ECC model of compacted spheres). Confirming this, our regression methodology (OEC, own external calibration method) yields a molecular weight of 956 g/mol. The variance seen between these values and the calculated molecular weight of (**2·Cs**)$_2$ falls within a range of 16-26%. Turning to the (CsHMDS)$_2$ assembly, its molecular weight is determined as 690 g/mol using Stalke's calibration curves and 624 g/mol via our OEC method. These results are close to the expected molecular weight of a THF-solvated dimer (CsHMDS)$_2$(THF-d_8)$_2$, deviating by only 5-6%. The DOSY data imparts a clear picture: in solution, the aggregate unit (**2·Cs**)$_2$(**CsHMDS**)$_2$ break apart into individual dimeric units (**2·Cs**)$_2$ and (CsHMDS)$_2$. Likewise, the dimeric (**2**···Cs)$_2$ entity maintains its integrity, showcasing no evident dissolution into monomeric units. Further support for this observation comes from the predicted hydrodynamic diameters, D_{hyd} ~ 17 Å for the dimeric (**2·Cs**)$_2$ unit and D_{hyd} ~ 12 Å for (CsHMDS)$_2$, obtained from the diffusion coefficients. Both D_{hyd} values are close to the isolated diameters $D_{X\text{-}ray}$ (~ 17 for (**2·Cs**)$_2$ and ~ 12 Å for (CsHMDS)$_2$) estimated from the molecular structure (see **Figure S41**). These hydrodynamic diameter analyses also point towards the existence of dimeric (**2·Cs**)$_2$ retaining their structures in solution and is further backed up by DFT analyses.

S4.1.11 ^{1}H DOSY NMR spectroscopic study of 3 with added THF

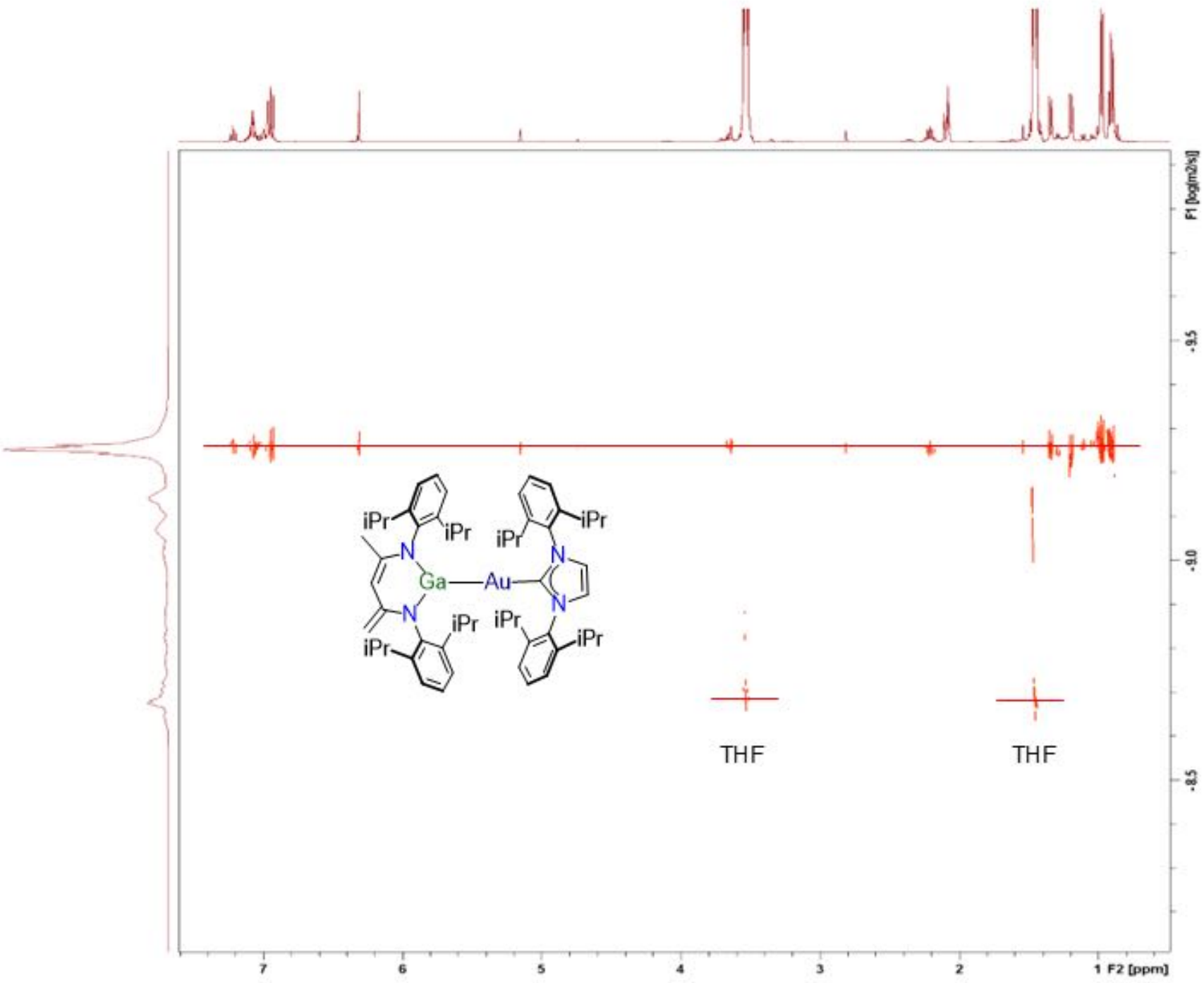

Figure S52. ^{1}H DOSY NMR 400 MHz of **3** in Tol-d_8 with added THF (15:1) at 298 K.

Comments on the ^{1}H DOSY NMR spectrum of 3 in Tol-d_8 solution with added THF (15:1): The resonances assigned to THF display diffusion behavior distinct from that of **3** and do not co-diffuse with **3** on the NMR timescale. This is inconsistent with a persistent THF adduct on the NMR timescale and supports only a weak, labile interaction in solution.

S5 Crystal Structure Determinations

Single-crystal X-ray diffraction data for all analysed samples were collected utilizing a Bruker D8 Quest Eco diffractometer, equipped with a Photon II 7 detector, and employing Mo radiation (λ = 0.71073 Å). The collections were performed under a nitrogen gas stream, facilitated by an Oxford Cryostream 800 unit,[21] at specified temperatures. The raw frame data underwent reduction through the application of APEX3,[22] and structures were elucidated via SHELXT[23] and refined employing full-matrix least squares refinement on F^2 data through SHELXL,[24] interfaced by OLEX2.[25] Unless noted otherwise, all non-hydrogen atoms underwent anisotropic refinement, with hydrogen atoms geometrically placed, and constrained to ride on their parent atoms. Any encountered disorder was addressed using a split site model, with restrained geometries and displacement parameters. Calculations of distances and angles incorporated the full covariance matrix. Concise summaries of selected crystallographic data are integrated within the text, while comprehensive details are accessible in the supplementary deposited CIF file (CCDC 2391227-2391237, 2502503-2502504 and 2546879-2546882), available at no charge from the Cambridge Crystallographic Data Centre via http://www.ccdc.cam.ac.uk/data_request/cif.

S5.1 Crystal data and structure refinement for 2·Li(crypt-222) (CCDC 2391227)

Empirical formula	$C_{47}H_{76}GaLiN_4O_6$	
Formula weight	869.77	
Temperature	150(1) K	
Wavelength	0.71073 Å	
Crystal system	Monoclinic	
Space group	P21/n	
Unit cell dimensions	a = 10.6062(2) Å	α = 90°
	b = 38.3535(7) Å	β = 106.9790(10)°
	c = 12.4523(2) Å	γ = 90°
Volume	4844.61(15) $Å^3$	
Z	4	
Density (calculated)	1.192 Mg/m^3	
Absorption coefficient	0.616 mm^{-1}	
F(000)	1872	
Crystal size	0.26 x 0.24 x 0.22 mm^3	
Theta range for data collection	2.225 to 32.583°.	
Index ranges	-16<=h<=16, -58<=k<=58, -18<=l<=18	
Reflections collected	233580	
Independent reflections	17625 [R(int) = 0.0316]	
Completeness to theta = 25.242°	99.8 %	
Absorption correction	Semi-empirical from equivalents	
Max. and min. transmission	0.1758 and 0.1374	
Refinement method	Full-matrix least-squares on F^2	
Data / restraints / parameters	17625 / 83 / 569	
Goodness-of-fit on F^2	1.091	
Final R indices [I>2sigma(I)]	R1 = 0.0404, wR2 = 0.0973	
R indices (all data)	R1 = 0.0487, wR2 = 0.1008	
Absolute structure parameter	n/a	
Extinction coefficient	0.688 and -0.490 e.$Å^{-3}$	
Largest diff. peak and hole	$C_{47}H_{76}GaLiN_4O_6$	

Additional details for 2·Li(crypt-222)*: This compound crystallized in the monoclinic space group P21/n. During the modelling of this compound, no restraints or constraints were applied, as no disorder within the crystal structure was observed. The hydrogen atoms were incorporated using a riding model, aligning them with their carrying atoms for the purposes of the refinement.*

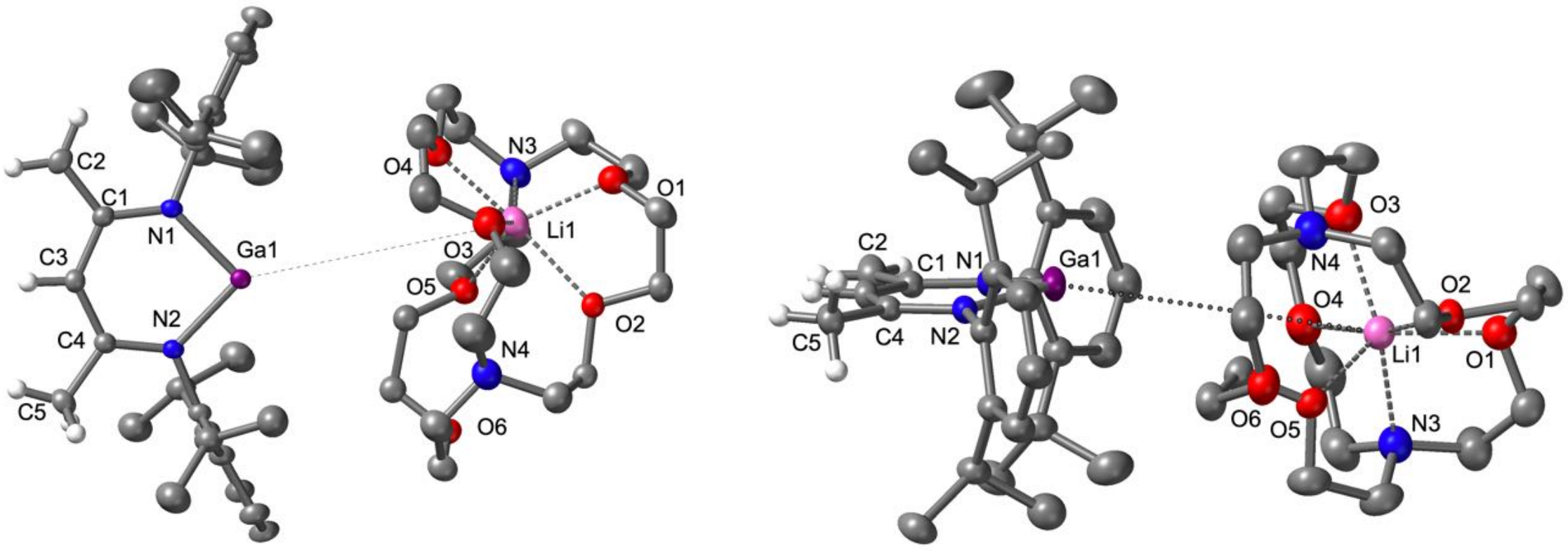

Figure S53. Front (left) and side (right) views of the molecular structure of **2·Li(crypt-222)** with displacement ellipsoids at 35% probability and disclosing the contents of one of the two molecules within the asymmetric unit. For clarity, all H atoms, except for those on the {C1,C5}-backbone, have been omitted. The figure elucidates selected bond lengths (Å) and angles (°) as follows: Ga(1)-N(2) 1.9862(9), Ga(1)-N(1) 1.9847(9), Ga(1)···Li(1) 5.769(2), N(2)-C(4) 1.3769(13), N(1)-C(1) 1.3800(14), C(4)-C(3) 1.4020(15), C(4)-C(5) 1.4555(15), C(3)-C(1) 1.4200(15), C(1)-C(2) 1.4232(16), N(1)-Ga(1)-N(2)91.47(4).

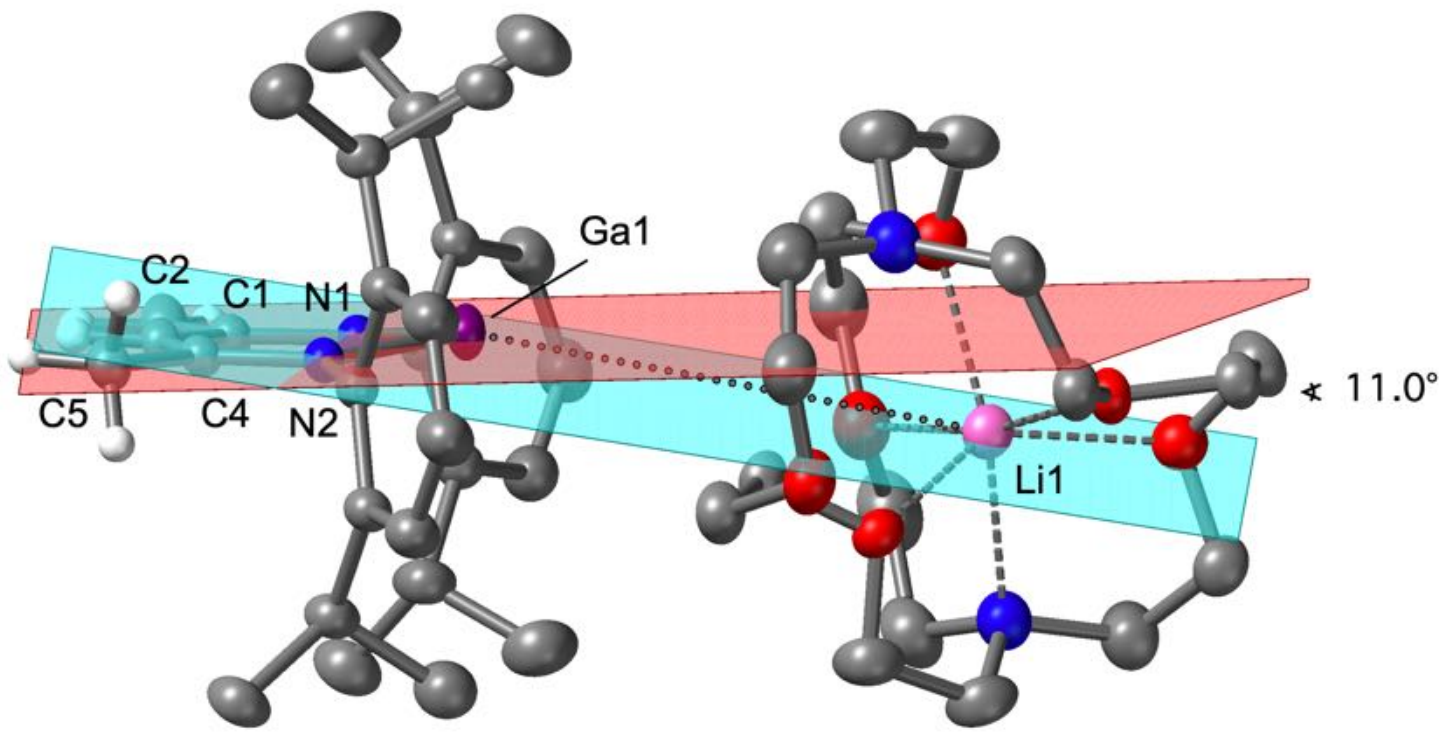

Figure S54. Representation of the 11.0° angle formed between the planes defined by atoms {Ga1,N1,N3,C1,C3,C4} and {N1,N2,Li1} in the structure of **2·Li(crypt-222)**.

S5.2 Crystal data and structure refinement for 2·K(crypt-222) (CCDC 2391228)

Empirical formula	$C_{47}H_{76}GaKN_4O_6$	
Formula weight	901.93	
Temperature	150(1) K	
Wavelength	0.71073 Å	
Crystal system	Triclinic	
Space group	P1	
Unit cell dimensions	a = 11.072(2) Å	α = 81.617(6)°.
	b = 11.857(2) Å	β = 86.333(8)°.
	c = 20.749(4) Å	γ = 70.086(4)°
Volume	2533.5(8) $Å^3$	
Z	2	
Density (calculated)	1.182 Mg/m^3	
Absorption coefficient	0.672 mm^{-1}	
F(000)	968	
Crystal size	0.18 x 0.16 x 0.1 mm^3	
Theta range for data collection	0.992 to 26.412°.	
Index ranges	-13<=h<=13, -14<=k<=14, -25<=l<=25	
Reflections collected	127108	
Independent reflections	20606 [R(int) = 0.0498]	
Completeness to theta = 25.242°	99.8 %	
Absorption correction	Semi-empirical from equivalents	
Max. and min. transmission	0.1340 and 0.1081	
Refinement method	Full-matrix least-squares on F^2	
Data / restraints / parameters	20606 / 3 / 1082	
Goodness-of-fit on F^2	1.047	
Final R indices [I>2sigma(I)]	R1 = 0.0462, wR2 = 0.1177	
R indices (all data)	R1 = 0.0548, wR2 = 0.1225	
Absolute structure parameter	0.050(10)	
Extinction coefficient	n/a	
Largest diff. peak and hole	0.701 and -0.336 e.$Å^{-3}$	

Additional details for 2·K(crypt-222): *This compound was refined as a 2-component inversion twin. This compound was crystallized and measured multiple times using Mo-Kα radiation (0.71073 Å) at different temperatures of 100, 150, and 200 K. All the crystals displayed modulated diffraction peaks. As the model does not accommodate this type of disorder, it is hypothesized to be the source of the observed long-range disorder of the non-modulated model (i.e., four molecules within the asymmetric unit). Furthermore, the non-modulated model is optimally represented as being twinned over a two-fold axis in a supercell, containing four molecules in the asymmetric unit, within the triclinic space group P-1. Exhaustive exploration of alternative space groups and twin laws yielded no better solutions.*

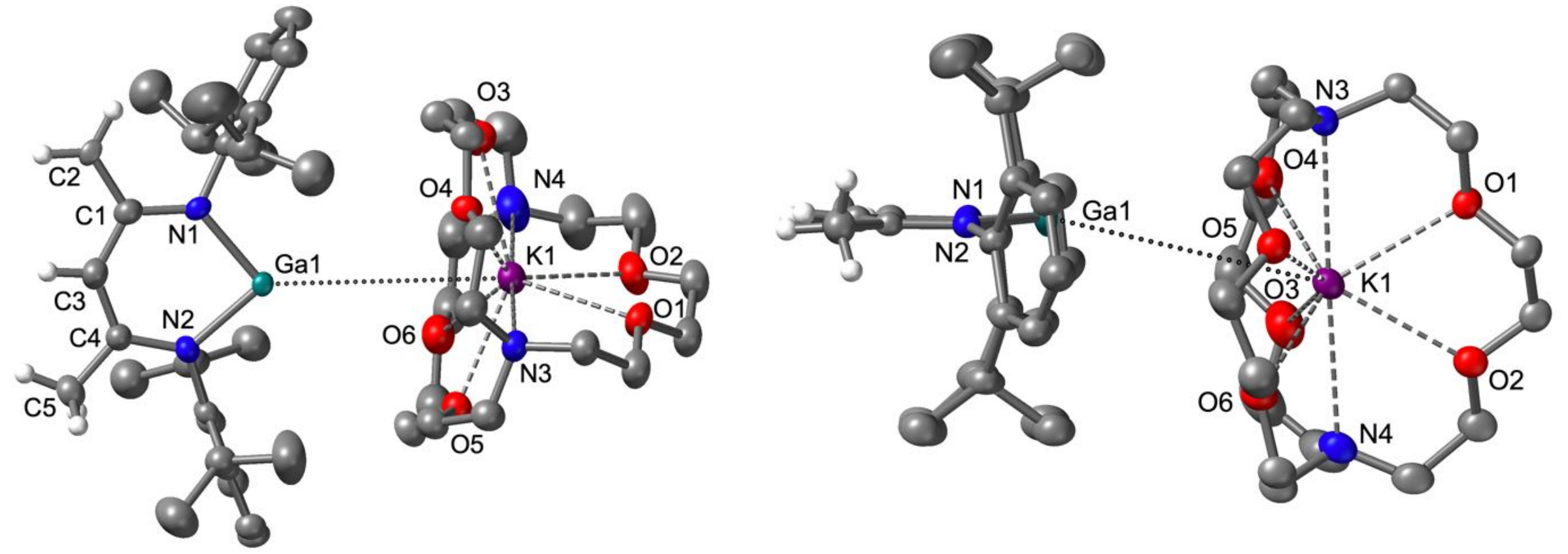

Figure S55. Front (left) and side (right) views of the molecular structure of **2·K(crypt-222)** are presented with displacement ellipsoids depicted at the 35% probability level, illustrating the contents of one of the molecules within the asymmetric unit. For clarity, all H atoms have been omitted, except for those located on the {C1, C5}-backbone. Selected bond lengths (Å) and angles (°) are specified as follows: Ga(1)-N(2) 1.992(4), Ga(1)-N(1) 1.987(4), Ga(1)···K(1) 5.1539(15), N(1)-C(1) 1.385(7), N(1)-C(6) 1.411(7), C(2)-C(1) 1.421(7), C(1)-C(3) 1.398(7),C(3)-C(4) 1.389(7), N(2)-Ga(1)-K(1) 133.52(12), N(1)-Ga(1)-N(2) 91.43(17), N(1)Ga(1)-K(1) 129.64(12).

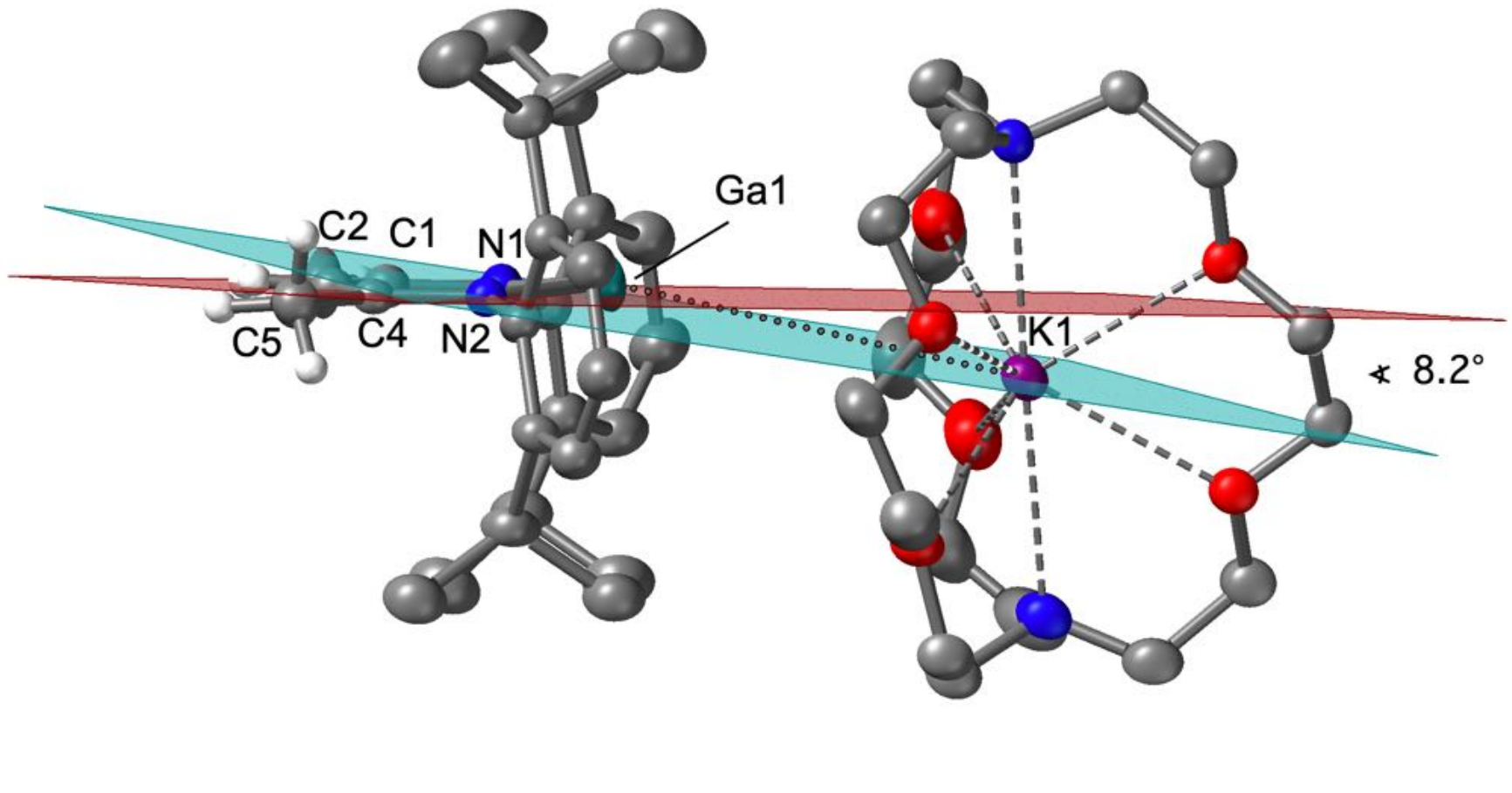

Figure S56. Representation of the 8.2° angle formed between the planes defined by atoms {Ga1,N1,N3,C1,C3,C4} and {N1,N2,K1} in the structure of **2·K(crypt-222)**.

S5.3 Crystal data and structure refinement for 2·Cs(crypt-222) (CCDC 2391229)

Empirical formula	$C_{47}H_{76}CsGaN_4O_6$	
Formula weight	995.74	
Temperature	150(1) K	
Wavelength	0.71073 Å	
Crystal system	Triclinic	
Space group	P*1*	
Unit cell dimensions	a = 11.1342(11) Å	α = 81.739(2)°
	b = 11.8773(11)) Å	β = 86.195(3)°
	c = 20.7480(19) Å	γ = 69.820(2)°
Volume	2548.2(4) Å^3	
Z	2	
Density (calculated)	1.298 Mg/m^3	
Absorption coefficient	1.290 mm^{-1}	
F(000)	1040	
Crystal size	0.18 x 0.16 x 0.08 mm^3	
Theta range for data collection	0.992 to 29.642°.	
Index ranges	-15<=h<=15, -16<=k<=16, -28<=l<=28	
Reflections collected	175282	
Independent reflections	28667 [R(int) = 0.0465]	
Completeness to theta = 25.242°	99.9 %	
Absorption correction	Semi-empirical from equivalents	
Max. and min. transmission	0.1402 and 0.1063	
Refinement method	Full-matrix least-squares on F^2	
Data / restraints / parameters	28667 / 3 / 1082	
Goodness-of-fit on F^2	1.139	
Final R indices [I>2sigma(I)]	R1 = 0.0513, wR2 = 0.1189	
R indices (all data)	R1 = 0.0601, wR2 = 0.1225	
Absolute structure parameter	0.051(13)	
Extinction coefficient	n/a	
Largest diff. peak and hole	3.356 and -1.426 e.Å^{-3}	

Additional details for 2·Cs(crypt-222): *This compound was crystallized, and its data were collected multiple times using Mo-Kα radiation (0.71073 Å) at various temperatures including 298, 150, and 100 K. Each crystallization presented modulated diffraction peaks. Given the model's inability to account for such disorder, this phenomenon is perceived to be the origin of the long-range disorder observed in the non-modulated model, evident by the presence of two molecules within the asymmetric unit and the unaccounted residual electron density surrounding the Cs atoms. Moreover, the non-modulated model is optimally represented as twinned, oriented over a two-fold axis in a supercell, and containing two molecules in the*

asymmetric unit within the triclinic space group P1. Extensive exploration into alternative space groups and twin laws yielded no viable alternatives, affirming the selected model and its inherent complexities.

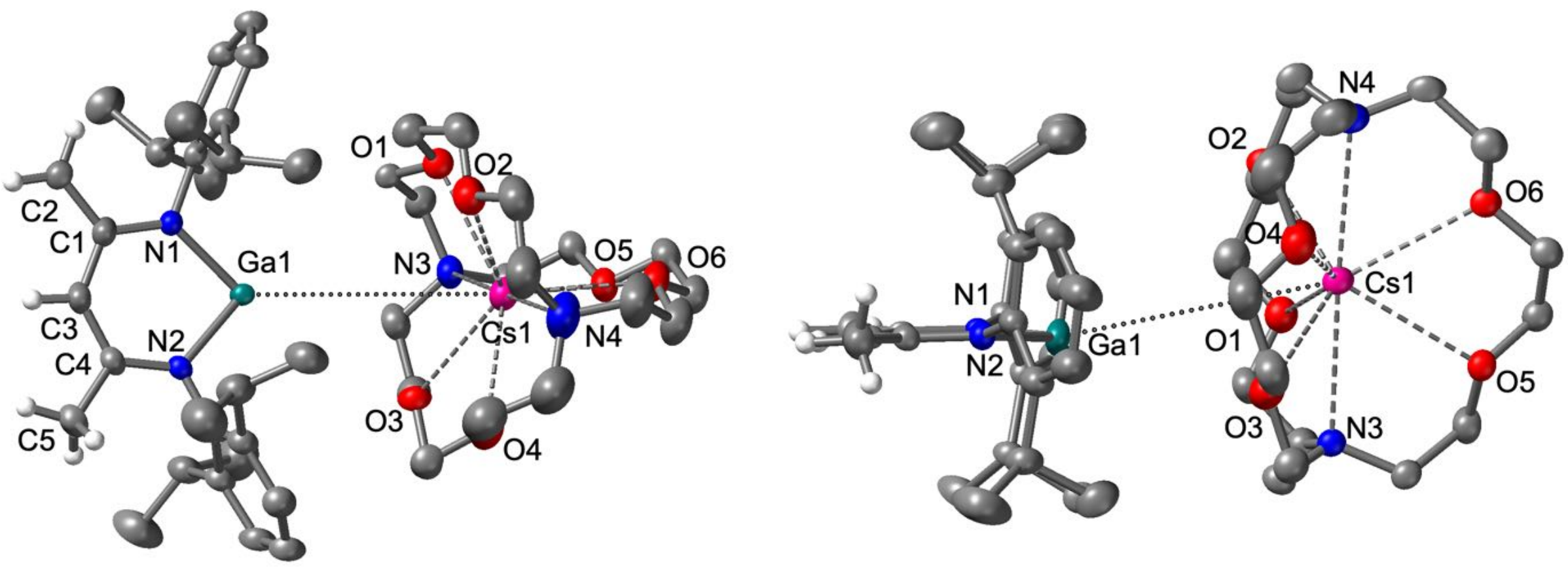

Figure S57. Front (left) and side (right) views of the molecular structure of **2·Cs(crypt-222)**, showing displacement ellipsoids at 35% probability. The figure illustrates the configuration of one of the two molecules present within the asymmetric unit. For clarity, all H atoms are omitted except those on the {C1,C5}-backbone. The selected bond lengths (Å) and angles (°) are as follows: Ga(1)···Cs(1) 5.128(1); Ga(1)-N(1) 2.000(5); Ga(1)-N(2) 1.995(5); N(1)-C(1) 1.377(8); N(2)-C(4) 1.368(8); C(1)-C(3) 1.405(10); C(1)-C(2) 1.427(9); C(3)-C(4) 1.391(10); C(4)-C(5) 1.460(9); and N(2)-Ga(1)-N(1) 91.3(2).

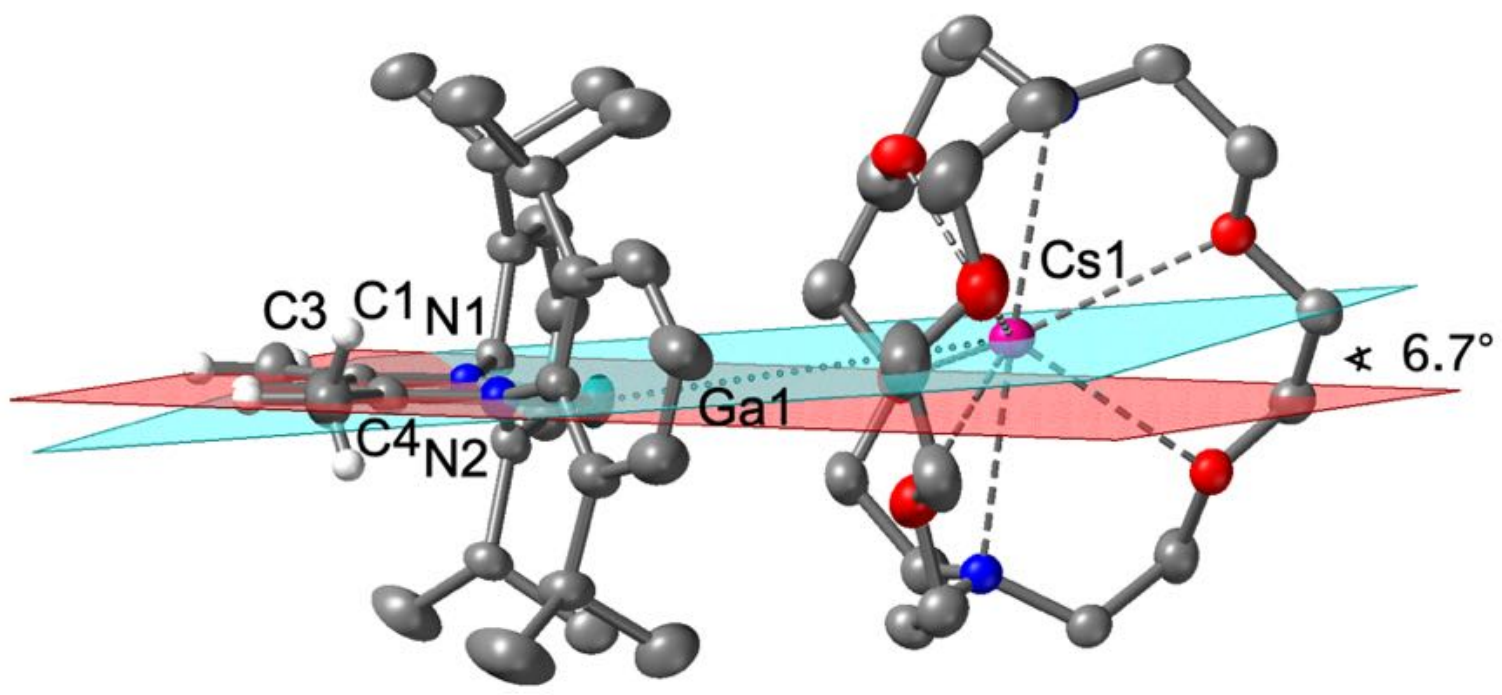

Figure S58. Representation of the 6.7° angle formed between the planes defined by atoms {Ga1,N1,N3,C1,C3,C4} and {N1,N2,Cs1} in the structure of **2·Cs(crypt-222)**.

S5.4 Crystal data and structure refinement for 2·Na(18-c-6) (CCDC 2391231)

Empirical formula	$C_{41}H_{64}GaN_2NaO_6$	
Formula weight	773.65	
Temperature	260(1) K	
Wavelength	0.71073 Å	
Crystal system	Orthorhombic	
Space group	Pbca	
Unit cell dimensions	a = 18.205(2) Å	α = 90°
	b = 18.267(3) Å	β = 90°
	c = 26.284(4) Å	γ = 90°
Volume	8741(2) $Å^3$	
Z	8	
Density (calculated)	1.176 Mg/m^3	
Absorption coefficient	0.683 mm^{-1}	
F(000)	3312	
Crystal size	0.3 x 0.2 x 0.18 mm^3	
Theta range for data collection	2.230 to 26.404°.	
Index ranges	-22<=h<=22, -22<=k<=22, -32<=l<=32	
Reflections collected	331438	
Independent reflections	8947 [R(int) = 0.0525]	
Completeness to theta = 25.242°	99.8 %	
Absorption correction	Semi-empirical from equivalents	
Max. and min. transmission	0.1660 and 0.1337	
Refinement method	Full-matrix least-squares on F^2	
Data / restraints / parameters	8947 / 1129 / 632	
Goodness-of-fit on F^2	1.075	
Final R indices [I>2sigma(I)]	R1 = 0.0520, wR2 = 0.1329	
R indices (all data)	R1 = 0.0713, wR2 = 0.1492	
Extinction coefficient	n/a	
Largest diff. peak and hole	0.455 and -0.414 e.$Å^{-3}$	

Additional details for 2·Na(18-c-6): *This compound crystallized in the orthorhombic space group Pbca. The crystallization and data collection for this compound were performed several times using Mo-Kα radiation (0.71073 Å) at varying temperatures including 100 K, 150 K, and 200 K. A phase change was consistently observed in all the crystals below 280 K, thus optimal data collection was determined to be at 260 K. The 18-c-6 ligand in the structure presented disorder over two main domains. To address this, appropriate restraints and constraints were applied to model the disorder. Additionally, hydrogen atoms were incorporated into the model using a riding model, considering them as part of the carrying atom for purposes of the refinement.*

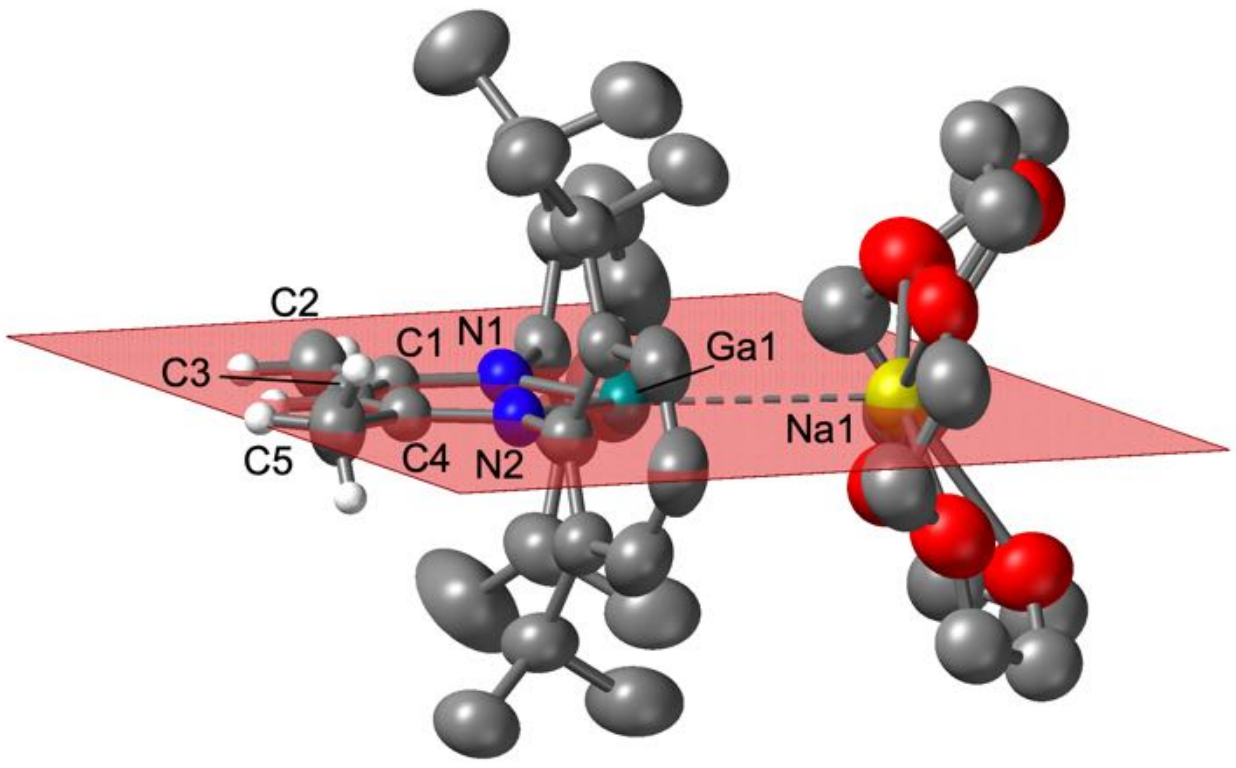

Figure S59. Molecular structure of **2·Na(18-c-6)**, featuring displacement ellipsoids at 35%. For clarity, all H atoms, except for those on the {C1,C5}-backbone, have been omitted. This view represents the coplanar configuration between the planes defined by atoms {Ga1,N1,N3,C1,C3,C4} and {N1,N2,Na1} in the structure. The figure elucidates selected bond lengths (Å) and angles (°) as follows: Ga(1)-Na(1) 3.3371(15), Ga(1)-N(1) 1.965(2), Ga(1)-N(2) 1.979(2), (1)-C(1) 1.382(3), N(1)-C(6) 1.424(3), C(4)-C(3) 1.354(4), C(4)-C(5) 1.510(4), C(1)-C(3) 1.447(4), N(1)-Ga(1)-N(2) 92.16(9).

S5.5 Crystal data and structure refinement for 2·K(18-c-6) (CCDC 2391232)

Empirical formula	$C_{41}H_{64}GaKN_2O_6$	
Formula weight	789.76	
Temperature	150(1) K	
Wavelength	0.71073 Å	
Crystal system	Orthorhombic	
Space group	Pbca	
Unit cell dimensions	a = 19.0416(3) Å	α = 90°
	b = 17.7891(3) Å	β = 90°
	c = 25.6657(4) Å	γ = 90°
Volume	8693.8(2) $Å^3$	
Z	8	
Density (calculated)	1.207 Mg/m^3	
Absorption coefficient	0.773 mm^{-1}	
F(000)	3376	
Crystal size	0.18 x 0.16 x 0.14 mm^3	
Theta range for data collection	1.587 to 27.904°.	
Index ranges	-25<=h<=25, -23<=k<=23, -33<=l<=33	
Reflections collected	444828	
Independent reflections	10377 [R(int) = 0.0846]	
Completeness to theta = 25.242°	99.8 %	
Absorption correction	Semi-empirical from equivalents	
Max. and min. transmission	0.1375 and 0.1082	
Refinement method	Full-matrix least-squares on F^2	
Data / restraints / parameters	10377 / 0 / 469	
Goodness-of-fit on F^2	1.021	
Final R indices [I>2sigma(I)]	R1 = 0.0378, wR2 = 0.0827	
R indices (all data)	R1 = 0.0558, wR2 = 0.0913	
Extinction coefficient	n/a	
Largest diff. peak and hole	0.561 and -0.399 e.$Å^{-3}$	

Additional details for 2·K(18-c-6): *This compound crystallized in the orthorhombic space group Pbca. During the modelling of this compound, no restraints or constraints were applied, as no disorder within the crystal structure was observed. The hydrogen atoms were incorporated using a riding model, aligning them with their carrying atoms for the purposes of the refinement.*

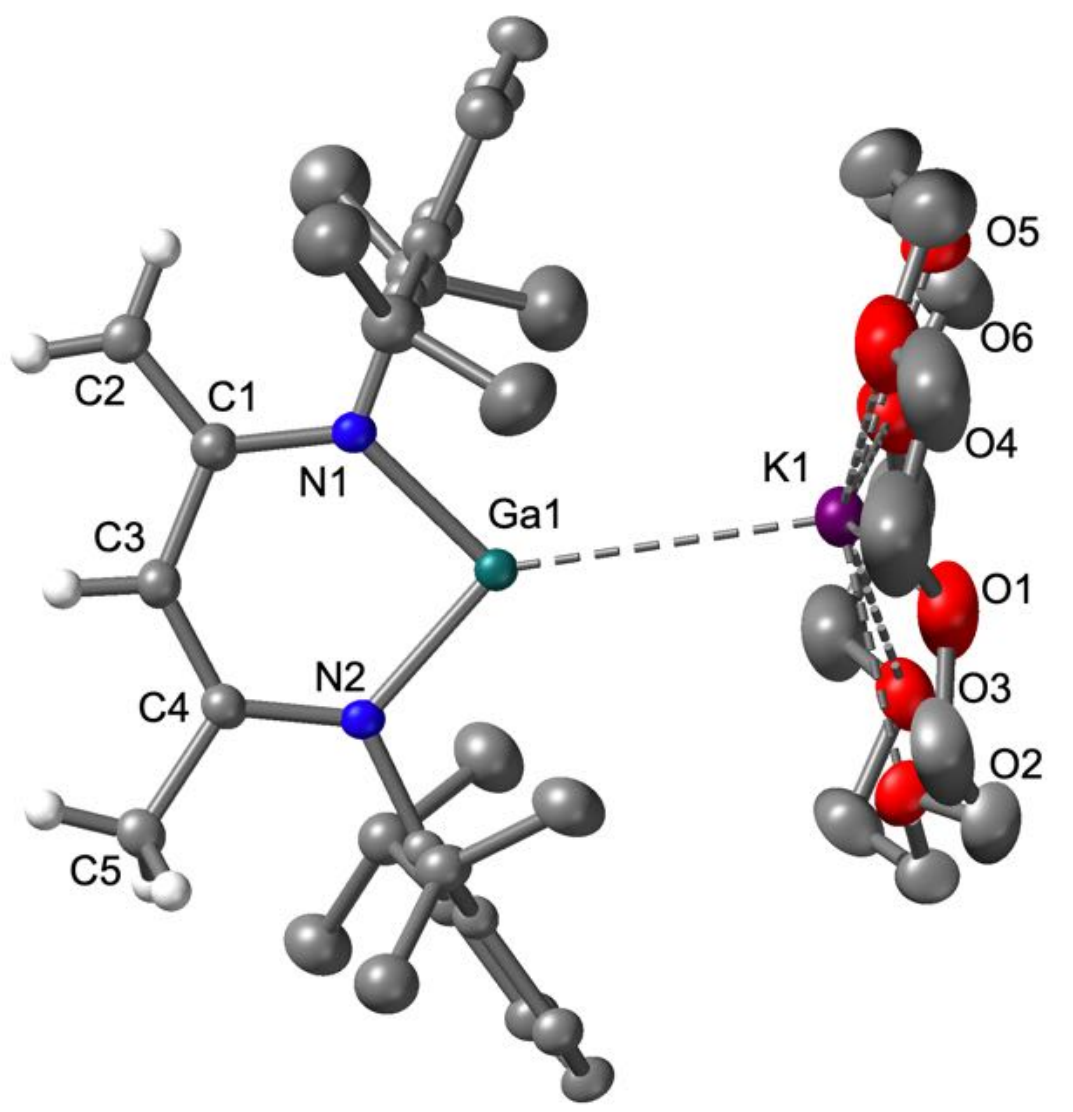

Figure S60. Molecular structure of **2·K**(18-c-6), featuring displacement ellipsoids at 35%. For clarity, all H atoms, except for those on the {C1,C5}-backbone, have been omitted. The figure elucidates selected bond lengths (Å) and angles (°) as follows: Ga(1)-K(1) 3.4899(5), Ga(1)-N(2) 1.9762(14), Ga(1)-N(1) 1.9536(14), K(1)-O(1) 2.8722(18), K(1)-O(2) 2.8226(18), K(1)-O(3) 2.9123(17), K(1)-O(4) 2.7562(16), K(1)-O(5) 2.8600(17), K(1)-O(6) 2.8030(18), N(2)-C(4) 1.375(2), N(1)-C(1) 1.386(2), N(2)-Ga(1)-K(1) 139.85(4).

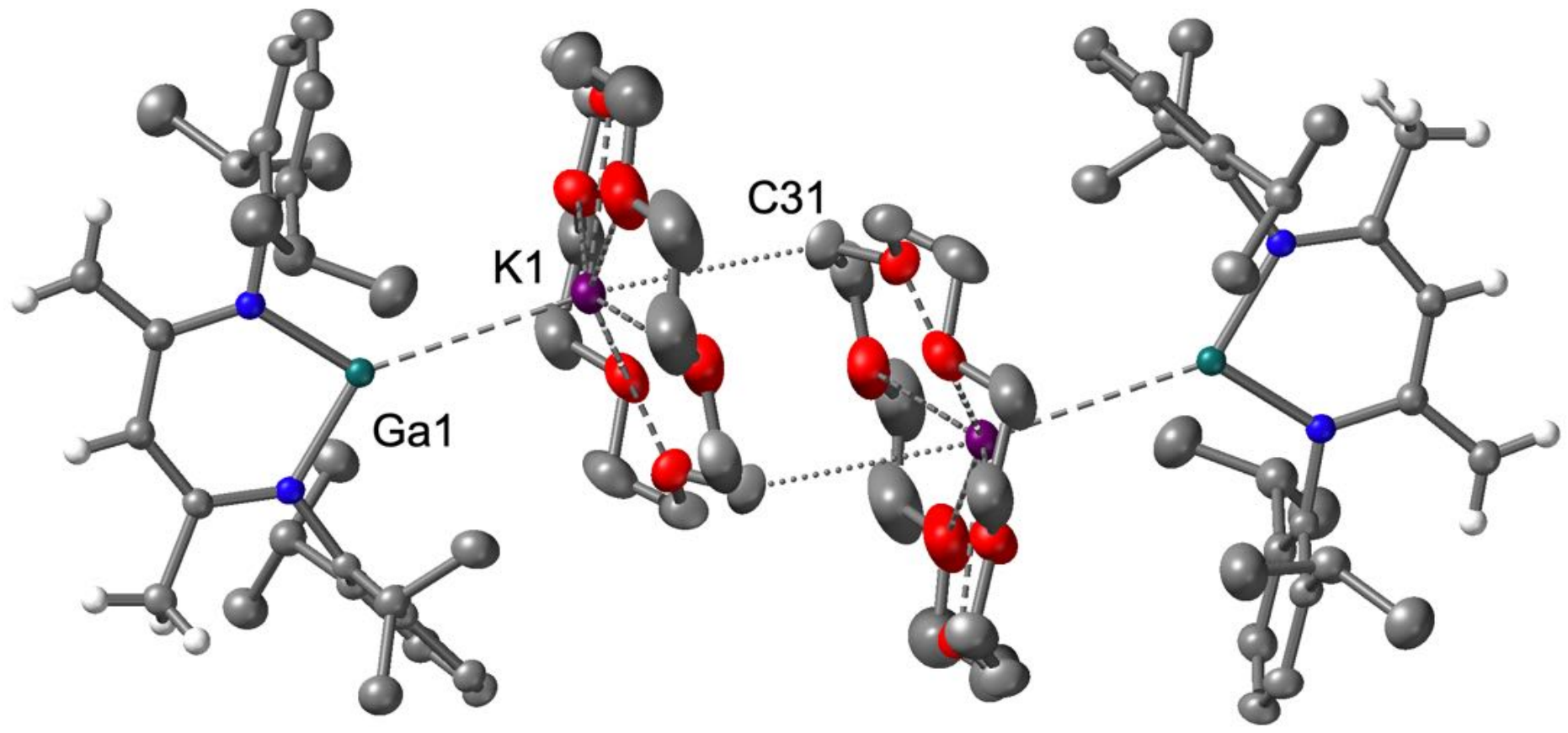

Figure S61. Representation of dimeric array found in **2·K(18-c-6)** through intermolecular K1···C31 contacts of 3.748(3) Å.

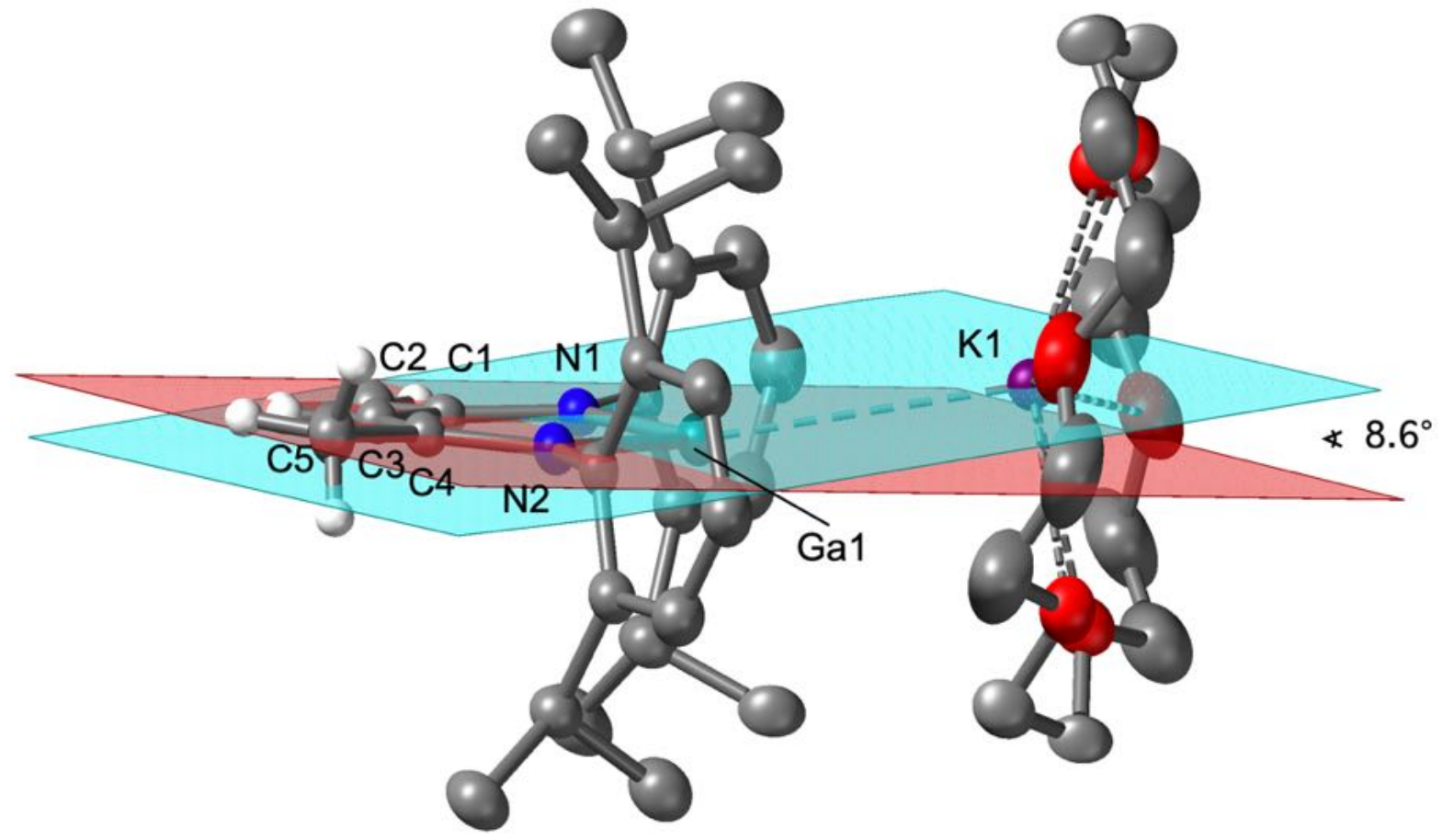

Figure S62. Representation of the 8.6° angle formed between the planes defined by atoms {Ga1,N1,N3,C1,C3,C4} and {N1,N2,K1} in the structure of **2·K(18-c-6).**

Crystal data and structure refinement for [2·K(18-c-6)]·benzene (CCDC 2391233)

Empirical formula	$C_{43}H_{69}GaKN_2O_6$	
Formula weight	818.82	
Temperature	150(1) K	
Wavelength	0.71073 Å	
Crystal system	Hexagonal	
Space group	P63/m	
Unit cell dimensions	a = 19.4344(2) Å	α = 90°
	b = 19.4344(2) Å	β = 90°
	c = 21.0533(4) Å	γ = 120°
Volume	6886.41(19) Å^3	
Z	6	
Density (calculated)	1.185 Mg/m^3	
Absorption coefficient	0.734 mm^{-1}	
F(000)	2634	
Crystal size	0.28 x 0.06 x 0.04 mm^3	
Theta range for data collection	2.282 to 25.693°.	
Index ranges	-23<=h<=23, -23<=k<=23, -25<=l<=25	
Reflections collected	140832	
Independent reflections	4495 [R(int) = 0.0819]	
Completeness to theta = 25.242°	99.9%	
Absorption correction	Semi-empirical from equivalents	
Max. and min. transmission	0.1341 and 0.1018	
Refinement method	Full-matrix least-squares on F^2	
Data / restraints / parameters	4495 / 2 / 252	
Goodness-of-fit on F^2	1.043	
Final R indices [I>2sigma(I)]	R1 = 0.0693, wR2 = 0.1823	
R indices (all data)	R1 = 0.0843, wR2 = 0.1949	
Extinction coefficient	n/a	
Largest diff. peak and hole	1.012 and -0.468 e.Å^{-3}	

Additional details for [2·K(18-c-6)]·benzene: *This compound crystallized in the hexagonal space group P63/m. During the modelling of this compound, no restraints or constraints were applied, as no disorder within the crystal structure was observed. The hydrogen atoms were incorporated using a riding model, aligning them with their carrying atoms for the purposes of the refinement.*

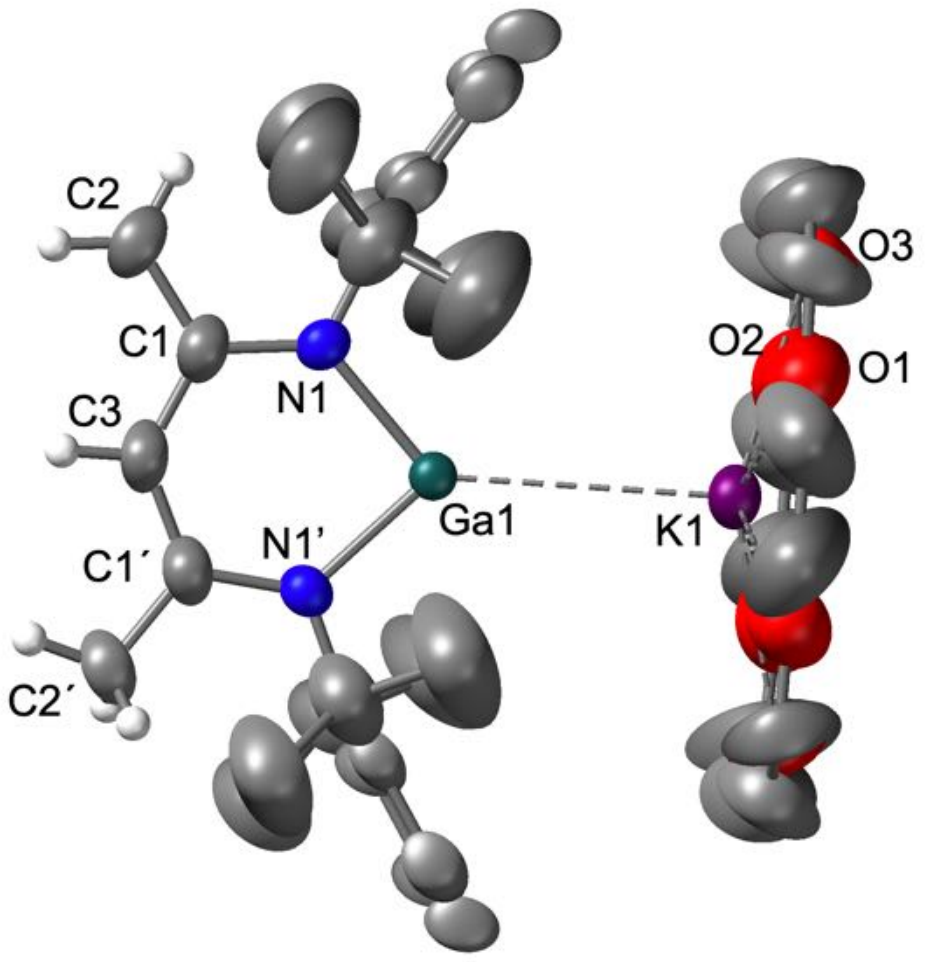

Figure S63. Molecular structure of [**2·K(18-c-6)**]**·benzene**, featuring displacement ellipsoids at 35%. For clarity, all H atoms, except for those on the {C1,C5}-backbone, and the benzene solvent molecule have been omitted. The figure elucidates selected bond lengths (Å) and angles (°) as follows: Ga(1)-K(1') 3.4872(13), Ga(1)-N(1'') 1.957(3), Ga(1)-N(1) 1.957(3), C(1)-C(2) 1.400(5), N(1'')-Ga(1)-N(1) 92.32(19). Symmetry operator for atoms labelled with (') is x,y,-z+1/2.

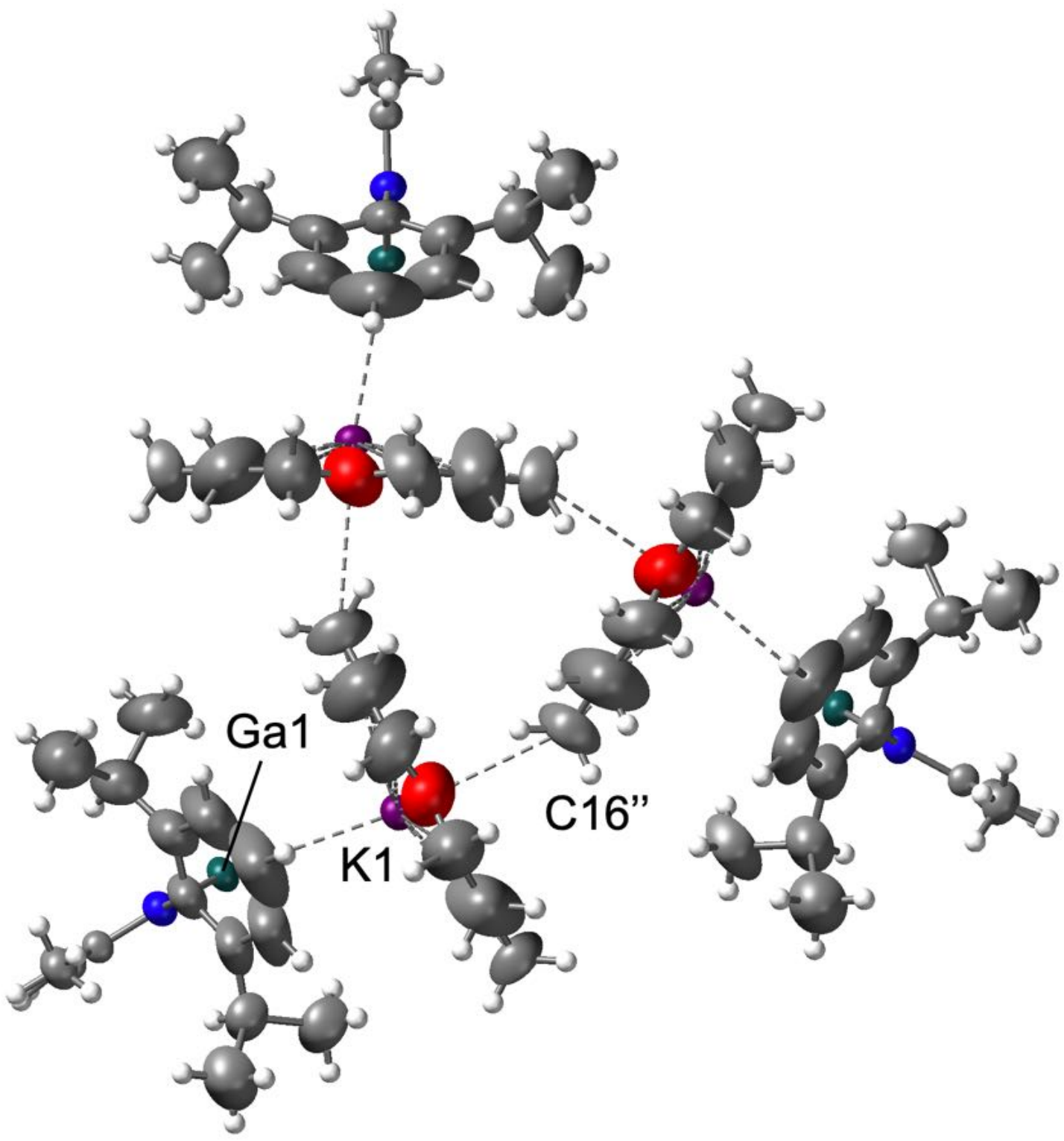

Figure S64. Representation of dimeric array found in [**2·K(18-c-6)**]·**benzene** through intermolecular K1···C16'' contacts of 3.69(1) Å. Symmetry operator for atoms labelled with ('') is -1+y-x,-x,z.

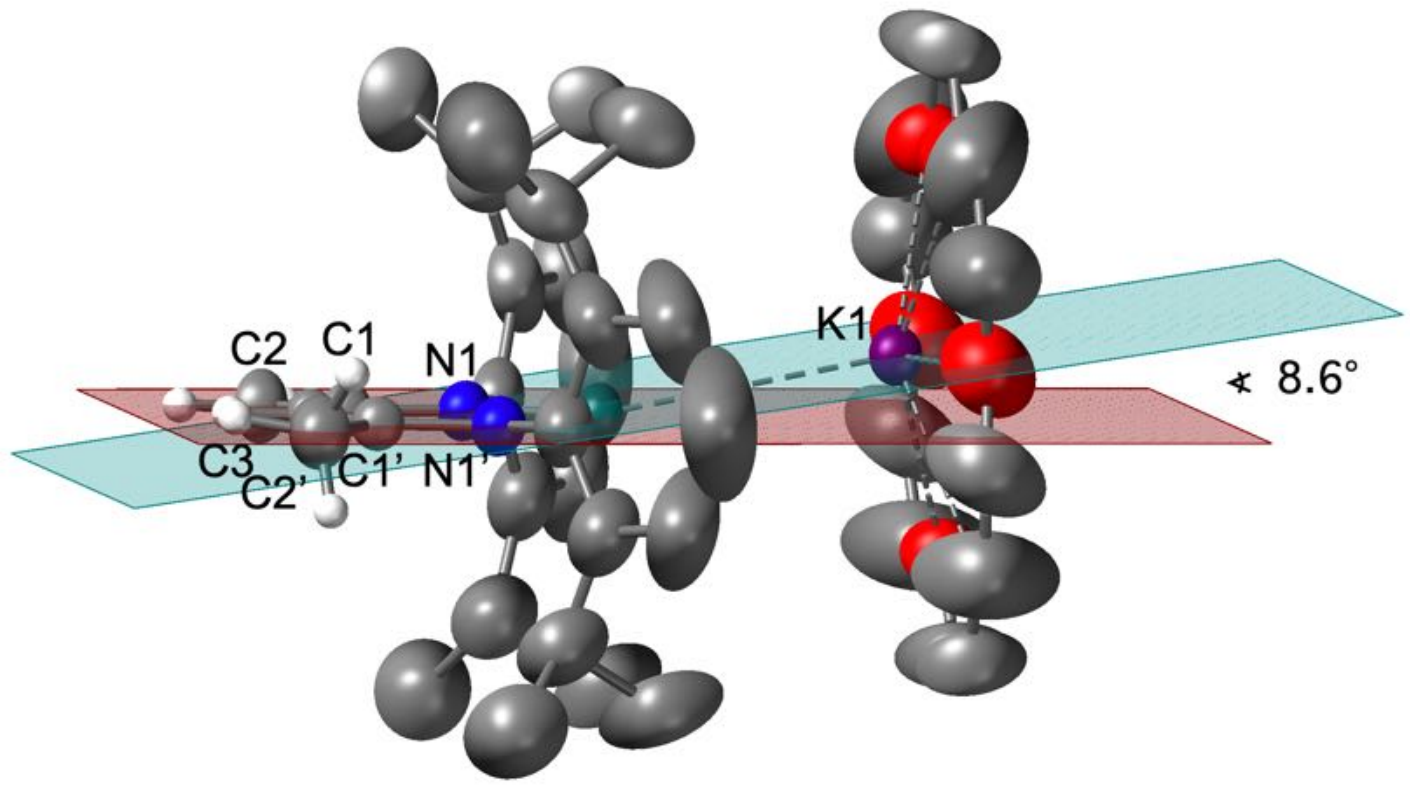

Figure S65. Representation of the 8.6° angle formed between the planes defined by atoms {Ga1,N1,N1’,C1,C3,C1} and {N1,N1’,K1} in the structure of [**2·K(18-c-6)**]·benzene**.**

S5.6 Crystal data and structure refinement for 2·K(18-c-6)(THF) (CCDC 2391234)

Empirical formula	$C_{45}H_{77}GaKN_2O_6$	
Formula weight	858.90	
Temperature	277 (1) K	
Wavelength	0.71073 Å	
Crystal system	Orthorhombic	
Space group	Ama2	
Unit cell dimensions	a = 20.103(10) Å	α = 90°
	b = 18.207(11) Å	β = 90°
	c = 13.649(8) Å	γ = 90°
Volume	4996(5) $Å^3$	
Z	4	
Density (calculated)	1.142 Mg/m^3	
Absorption coefficient	0.678 mm^{-1}	
F(000)	1852	
Crystal size	0.12 x 0.1 x 0.08 mm^3	
Theta range for data collection	2.456 to 25.035°.	
Index ranges	--23<=h<=23, -21<=k<=21, -16<=l<=16	
Reflections collected	19258	
Independent reflections	4545 [R(int) = 0.0739]	
Completeness to theta = 25.242°	99.6 %	
Absorption correction	Semi-empirical from equivalents	
Max. and min. transmission	0.1640 and 0.1239	
Refinement method	Full-matrix least-squares on F^2	
Data / restraints / parameters	4545 / 628 / 366	
Goodness-of-fit on F^2	1.007	
Final R indices [I>2sigma(I)]	R1 = 0.0534, wR2 = 0.1202	
R indices (all data)	R1 = 0.1375, wR2 = 0.1561	
Absolute structure parameter	0.022(12)	
Largest diff. peak and hole	0.265 and -0.189 e.Å-3	

Additional details for 2·K(18-c-6)(THF): *This compound crystallized in the orthorhombic space group Ama2, with an absolute structure parameter close to zero. Crystallization and data collection were attempted multiple times at different temperatures, including 100 K, 150 K, 260 K, and 277 K. The sample is suspected to undergo a phase transition around these temperatures. The most reliable data set was obtained at 277 K. The structure at this temperature shows a high degree of disorder, particularly affecting the 18-c-6 ether ligand and the organic fragment bound to gallium. To manage this disorder, appropriate restraints and constraints were applied during refinement. Although the data quality is limited at this temperature and the structure exhibits considerable disorder, this model provides a valid basis for establishing atomic connectivity and confirming the chemical identity of the compound.*

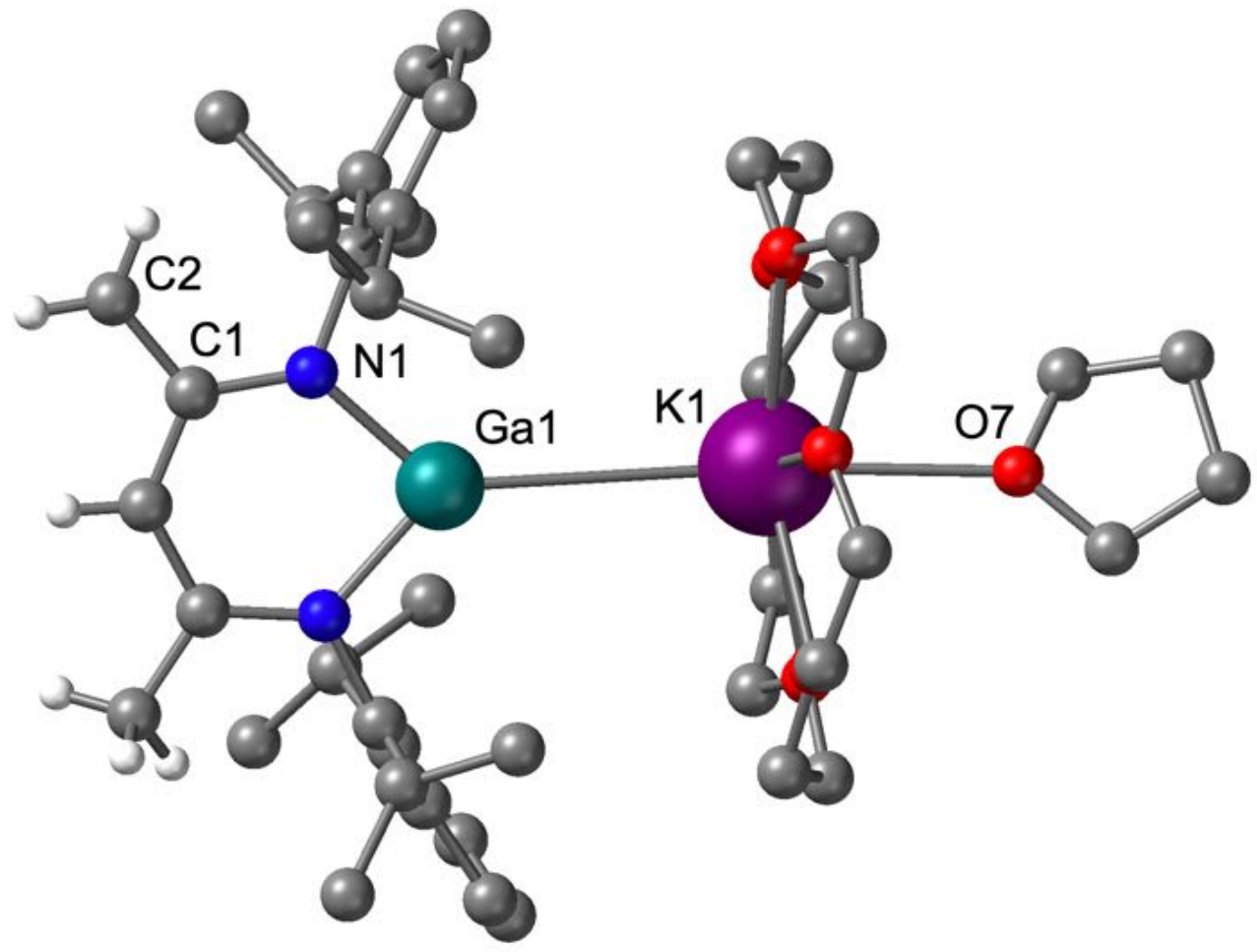

Figure S66. Ball-and-stick model of the molecular structure of **2·K(18-c-6)(THF),** illustrating atomic connectivity. Due to a high degree of disorder affecting the entire molecule, meaningful metrics, such as precise bond lengths and angles, cannot be reliably discussed. This representation is intended to confirm the overall chemical identity.

S5.7 Crystal data and structure refinement for 2·Cs(18-c-6) (CCDC 2391235)

Empirical formula	$C_{41}H_{64}CsGaN_2O_6$	
Formula weight	883.57	
Temperature	150(1) K	
Wavelength	0.71073 Å	
Crystal system	Triclinic	
Space group	P-1	
Unit cell dimensions	a = 18.008(5) Å	α = 90.004(18)°
	b = 19.430(5) Å	β = 89.965(16)°
	c = 25.598(4) Å	γ = 89.91(2)°
Volume	8957(4) Å^3	
Z	8	
Density (calculated)	1.310 Mg/m^3	
Absorption coefficient	1,458 mm^{-1}	
F(000)	3664	
Crystal size	0.28 x 0.26 x 0.14 mm^3	
Theta range for data collection	0.795 to 25.726°.	
Index ranges	21<=h<=21, -23<=k<=23, -31<=l<=31	
Reflections collected	428380	
Independent reflections	34042 [R(int) = 0.0854]	
Completeness to theta = 25.242°	99.9 %	
Absorption correction	Semi-empirical from equivalents	
Max. and min. transmission	0.1341 and 0.0904	
Refinement method	Full-matrix least-squares on F^2	
Data / restraints / parameters	34042 / 4204 / 1838	
Goodness-of-fit on F^2	1.130	
Final R indices [I>2sigma(I)]	R1 = 0.0938, wR2 = 0.1682	
R indices (all data)	R1 = 0.1327, wR2 = 0.1874	
Extinction coefficient	n/a	
Largest diff. peak and hole	2.442 and -2.041 e.Å^{-3}	

Additional details for 2·Cs(18-c-6): *This compound was refined as a 2-component inversion twin. This compound was crystallized and measured multiple times using Mo-Kα radiation (0.71073 Å) at different temperatures of 100, 150, and 200 K. All the crystals displayed modulated diffraction peaks. As the model does not accommodate this type of disorder, it is hypothesized to be the source of the observed long-range disorder of the non-modulated model (i.e., four molecules within the asymmetric unit). Furthermore, the non-modulated model is optimally represented as being twinned over a two-fold axis in a supercell, containing four molecules in the asymmetric unit, within the triclinic space group P-1. Exhaustive exploration of alternative space groups and twin laws yielded no better solutions.*

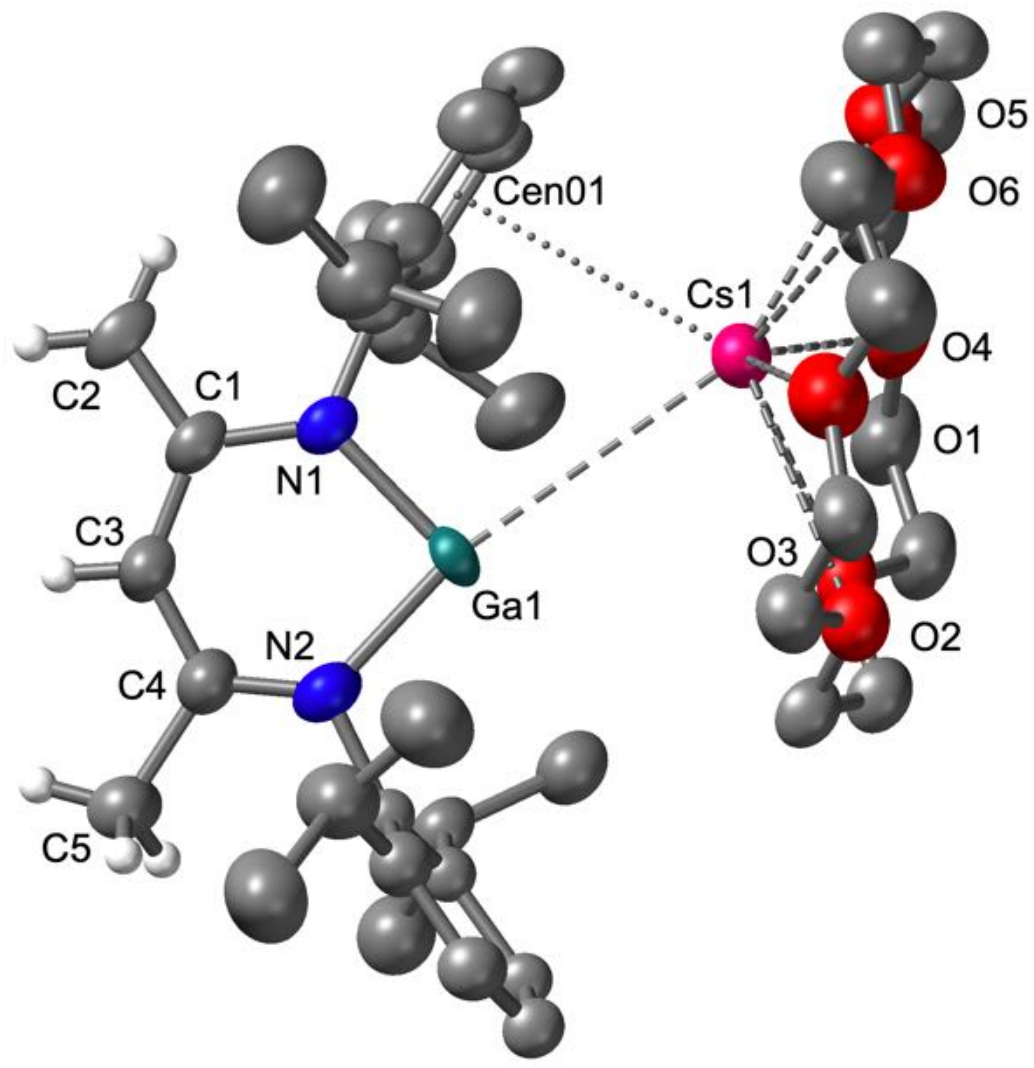

Figure S67. Molecular structure of **2·Cs(18-c-6)**, featuring one molecule from the asymmetric unit with displacement ellipsoids at 35%. For clarity, all H atoms, except for those on the {C1,C5}-backbone, have been omitted. The figure elucidates selected bond lengths (Å) and angles (°) as follows: Ga(1)…Cs(1) 3.7076(17), Cs(1)···Cent1 3.556(7), Cs(1)-O(1) 3.012(9), Cs(1)-O(2) 3.299(10), Cs(1)-O(3) 3.100(10), Cs(1)-O(4) 3.107(10), Cs(1)-O(5) 3.086(9), Cs(1)-O(6) 3.130(11), Ga(1)-N(2) 1.979(11), Ga(1)-N(1) 1.971(11), N(2)-C(4) 1.313(17), N(1)-C(1) 1.412(18), N(1)-Ga(1)-N(2) 91.7(5).

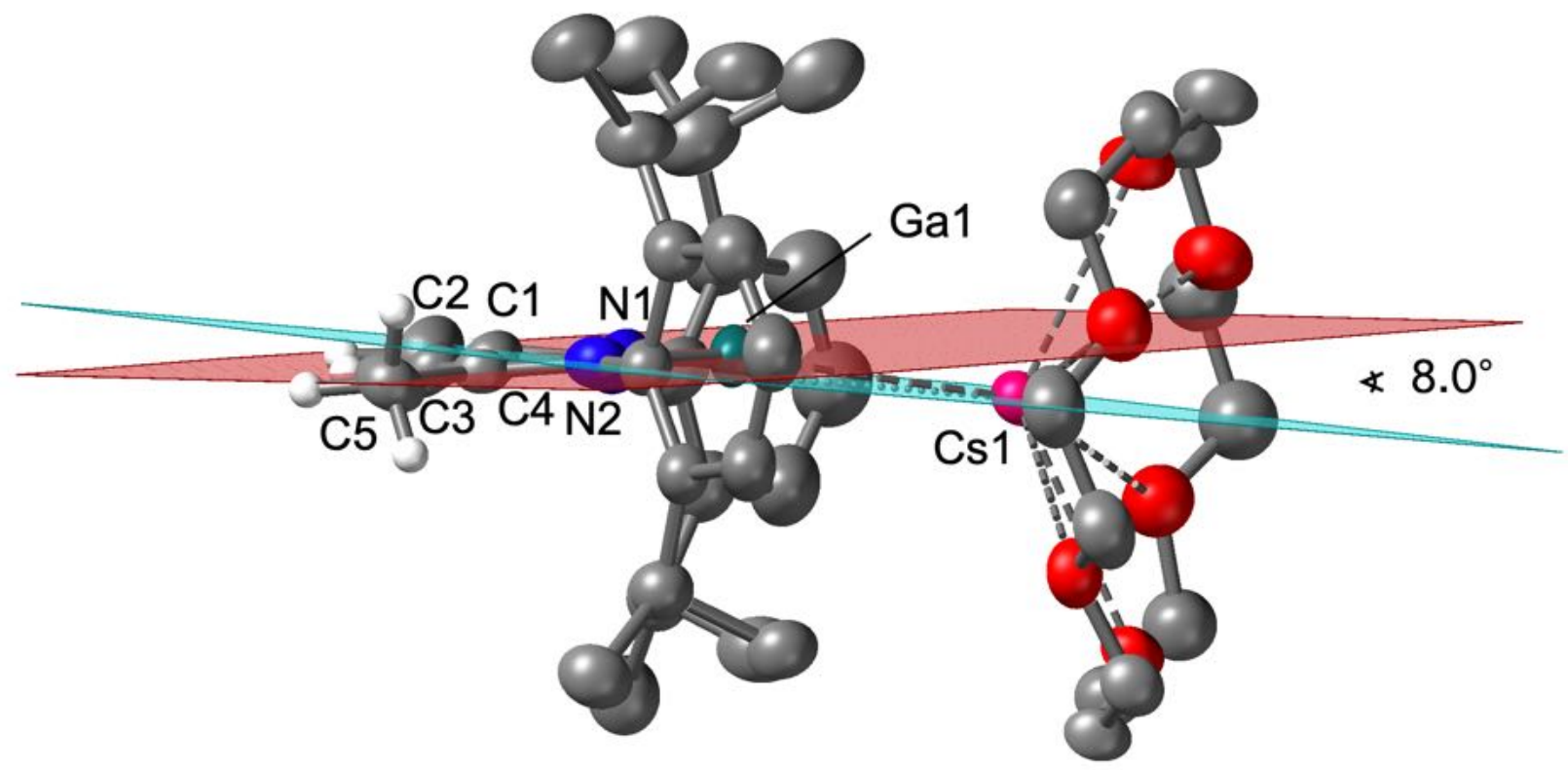

Figure S68. Representation of the 8.0° angle formed between the planes defined by atoms {Ga1,N1,N3,C1,C3,C4} and {N1,N2,Cs1} in the structure of **2·Cs(18-c-6)**.

S5.8 Crystal data and structure refinement for 2·Cs(18-c-6)$_2$ (CCDC 2391230)

Empirical formula	$C_{65}H_{100}CsGaN_2O_{12}$	
Formula weight	1304.09	
Temperature	150(1) K	
Wavelength	0.71073 Å	
Crystal system	Monoclinic	
Space group	C/c	
Unit cell dimensions	a = 12.9635(2) Å	α = 90°
	b = 26.7272(4) Å	β = 104.4420(10)°
	c = 20.5498(3) Å	γ = 90°
Volume	6895.06(18) Å^3	
Z	4	
Density (calculated)	1.256 Mg/m^3	
Absorption coefficient	0.976 mm^{-1}	
F(000)	2744	
Crystal size	0.32 x 0.3 x 0.2 mm^3	
Theta range for data collection	2.047 to 26.397°.	
Index ranges	-16<=h<=15, -33<=k<=33, -25<=l<=25	
Reflections collected	47728	
Independent reflections	12653 [R(int) = 0.0983]	
Completeness to theta = 25.242°	99.9 %	
Absorption correction	Semi-empirical from equivalents	
Max. and min. transmission	0.1352 and 0.0983	
Refinement method	Full-matrix least-squares on F^2	
Data / restraints / parameters	12653 / 2642 / 1054	
Goodness-of-fit on F^2	1.086	
Final R indices [I>2sigma(I)]	R1 = 0.0629, wR2 = 0.1581	
R indices (all data)	R1 = 0.0662, wR2 = 0.1609	
Absolute structure parameter	0.28(3)	
Extinction coefficient	n/a	
Largest diff. peak and hole	1.508 and -1.283 e.Å^{-3}	

Additional details for 2·Cs(18-c-6)$_2$: *This compound crystallized in the monoclinic C/c space group, incorporating two molecules of benzene within the asymmetric unit. Both 18-c-6 ligands exhibited disorder, modelled over two principal domains, with the application of suitable restraints and constraints to maintain the model's integrity. For refinement purposes, the hydrogen atoms were included using a riding model, aligning them appropriately with their respective carrying atoms.*

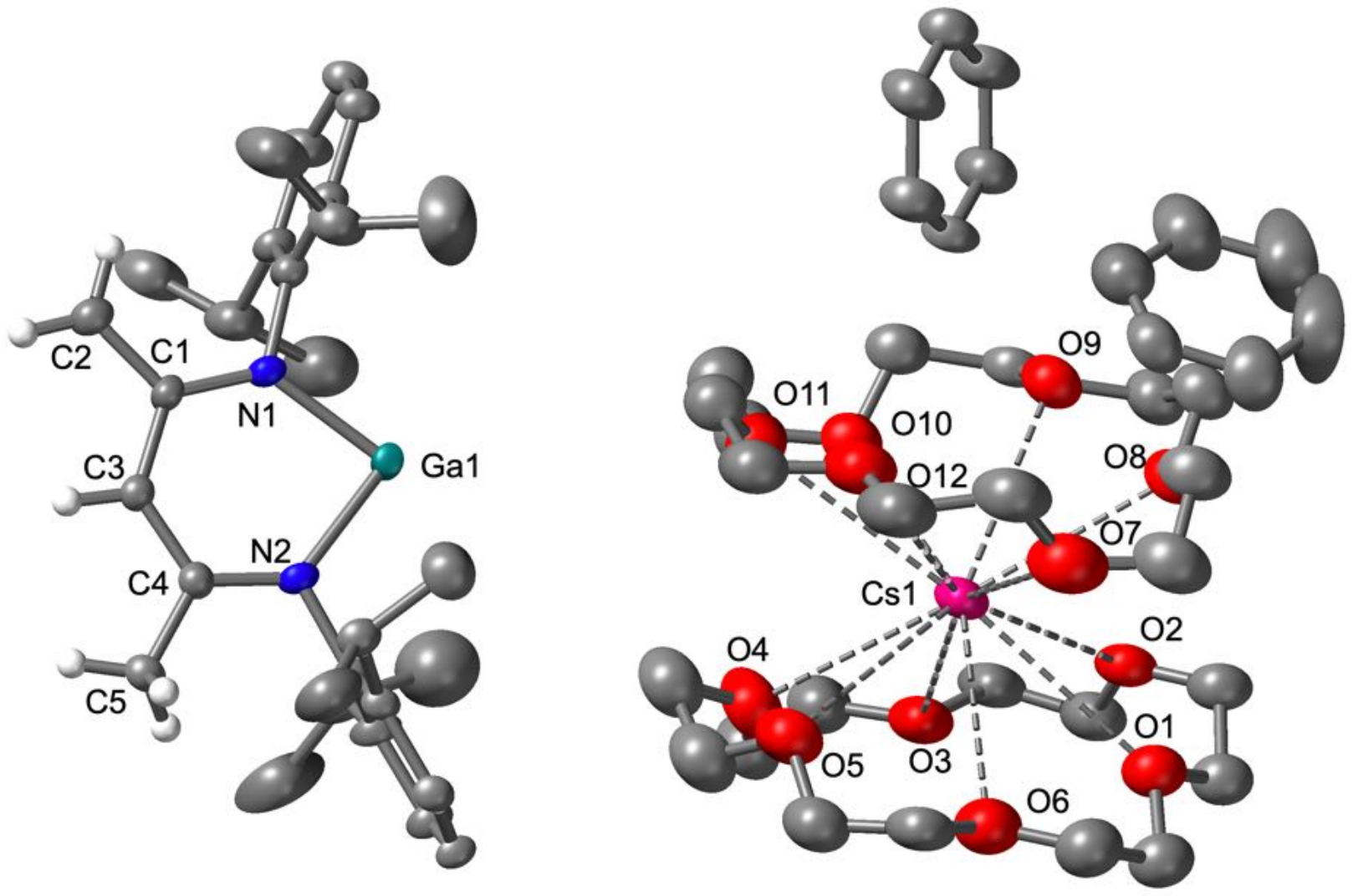

Figure S69. Front (left) and side (right) views of the molecular structure of **2·Cs(18-c-6)$_2$**, displaying displacement ellipsoids at a 35% probability level and revealing the contents of one of the molecules within the asymmetric unit. The minor disordered components of the 18-c-6 groups, along with all H atoms, have been omitted for clarity, except those located on the {C1, C5}-backbone. Selected bond lengths (Å) and angles (°) are as follows: Cs(1)-O(8) 3.298(8), Cs(1)-O(3) 3.358(8), Cs(1)-O(2) 3.296(8), Cs(1)-O(5) 3.334(8), Cs(1)-O(4) 3.283(9), Cs(1)-O(7) 3.300(9), Ga(1)-N(1) 1.990(7), Ga(1)-N(2) 2.002(7), N(1)-C(1) 1.369(10), N(2)-C(4) 1.364(10), C(2)-C(1) 1.425(11), C(1)-C(3) 1.424(12), C(4)-C(5) 1.446(11), C(4)-C(3) 1.402(12), N(1)-Ga(1)-N(2) 90.4(3).

S5.9 Crystal data and structure refinement for $[(2\cdot K)_2(KHMDS)_2]_\infty$ (CCDC 2391236)

Empirical formula	$C_{35}H_{58}GaK_2N_3Si_2$	
Formula weight	724.94	
Temperature	150(2) K	
Wavelength	0.71073 Å	
Crystal system	Monoclinic	
Space group	*C*2/c	
Unit cell dimensions	a = 18.2200(7) Å	α = 90°
	b = 21.2293(9) Å	β = 96.002(2)°
	c = 21.1386(9) Å	γ = 90°
Volume	8131.5(6) $Å^3$	
Z	8	
Density (calculated)	1.184 Mg/m^3	
Absorption coefficient	0.966 mm^{-1}	
F(000)	3088	
Crystal size	0.24 x 0.22 x 0.20 mm^3	
Theta range for data collection	2.248 to 31.592°.	
Index ranges	-25<=h<=26, -31<=k<=31, -31<=l<=29	
Reflections collected	114561	
Independent reflections	13612 [R(int) = 0.0535]	
Completeness to theta = 25.242°	99.9 %	
Absorption correction	Semi-empirical from equivalents	
Max. and min. transmission	0.1199 and 0.0866	
Refinement method	Full-matrix least-squares on F^2	
Data / restraints / parameters	13612 / 0 / 416	
Goodness-of-fit on F^2	1.093	
Final R indices [I>2sigma(I)]	R1 = 0.0395, wR2 = 0.0785	
R indices (all data)	R1 = 0.0674, wR2 = 0.0878	
Extinction coefficient	n/a	
Largest diff. peak and hole	0.485 and -0.501 e.Å-3	

Additional details for $[(2\cdot K)_2(KHMDS)_2]_\infty$: *This compound crystallized in the monoclinic space group C2/c. Data collection was acquired at 150 K. The structure obtained at this temperature displayed good data quality and structural integrity, allowing for reliable metric analysis and discussion of bond lengths and angles.*

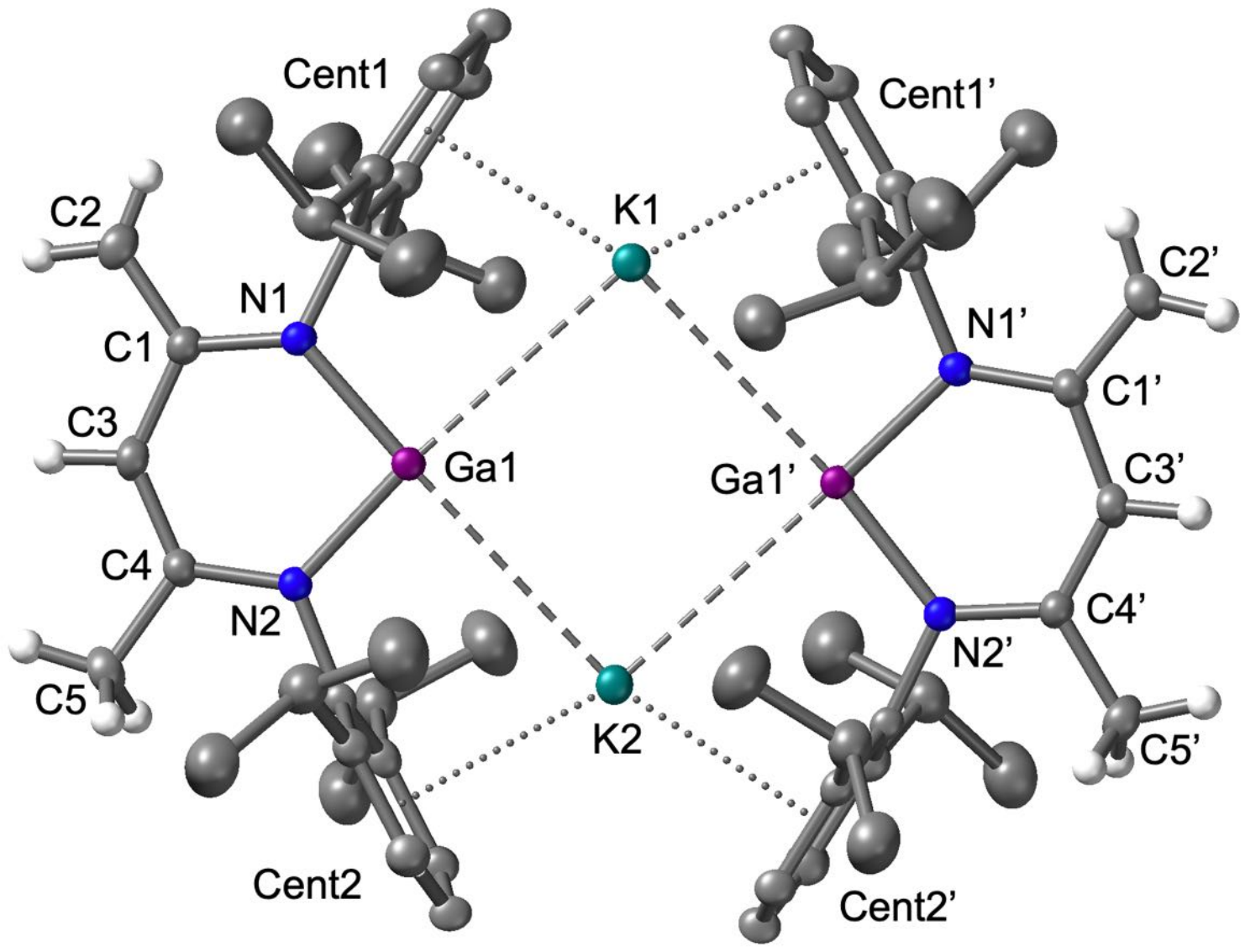

Figure S70. Molecular structure of [**(2·K)$_2$(KHMDS)$_2$**] with displacement ellipsoids at 50% probability. The figure illustrates the configuration of the dimeric (**2·K**)$_2$ array present within the asymmetric unit. For clarity, all H atoms are omitted except those on the {C1,C5}-backbone. The selected bond lengths (Å) and angles (°) are as follows: Ga(1)-K(1) 3.4679(4), Ga(1)-K(2) 3.4779(4), Ga(1)-N(1) 1.9380(12), Ga(1)-N(2) 1.9446(12), K(1)···Cent1 2.921(1), K(1)···Cent1' 2.921(1), K(2)···Cent2 2.894(1), K(2)···Cent2' 2.894(1), N(1)-C(1) 1.3886(19), N(2)-C(4) 1.3803(19), C(2)-C(1) 1.394(2), C(1)-C(3) 1.435(2), C(3)-C(4) 1.385(2), C(4)-C(5) 1.477(2), K(1)-Ga(1)-K(2) 88.949(10), N(1)-Ga(1)-K(1) 89.94(4), N(1)-Ga(1)-K(2) 172.46(4), N(1)-Ga(1)-N(2) 93.57(5), N(2)-Ga(1)-K(1) 171.87(4), N(2)-Ga(1)-K(2) 86.61(4). The symmetry transformation used to generate equivalent atoms labelled with (') is -x,y,1/2-z.

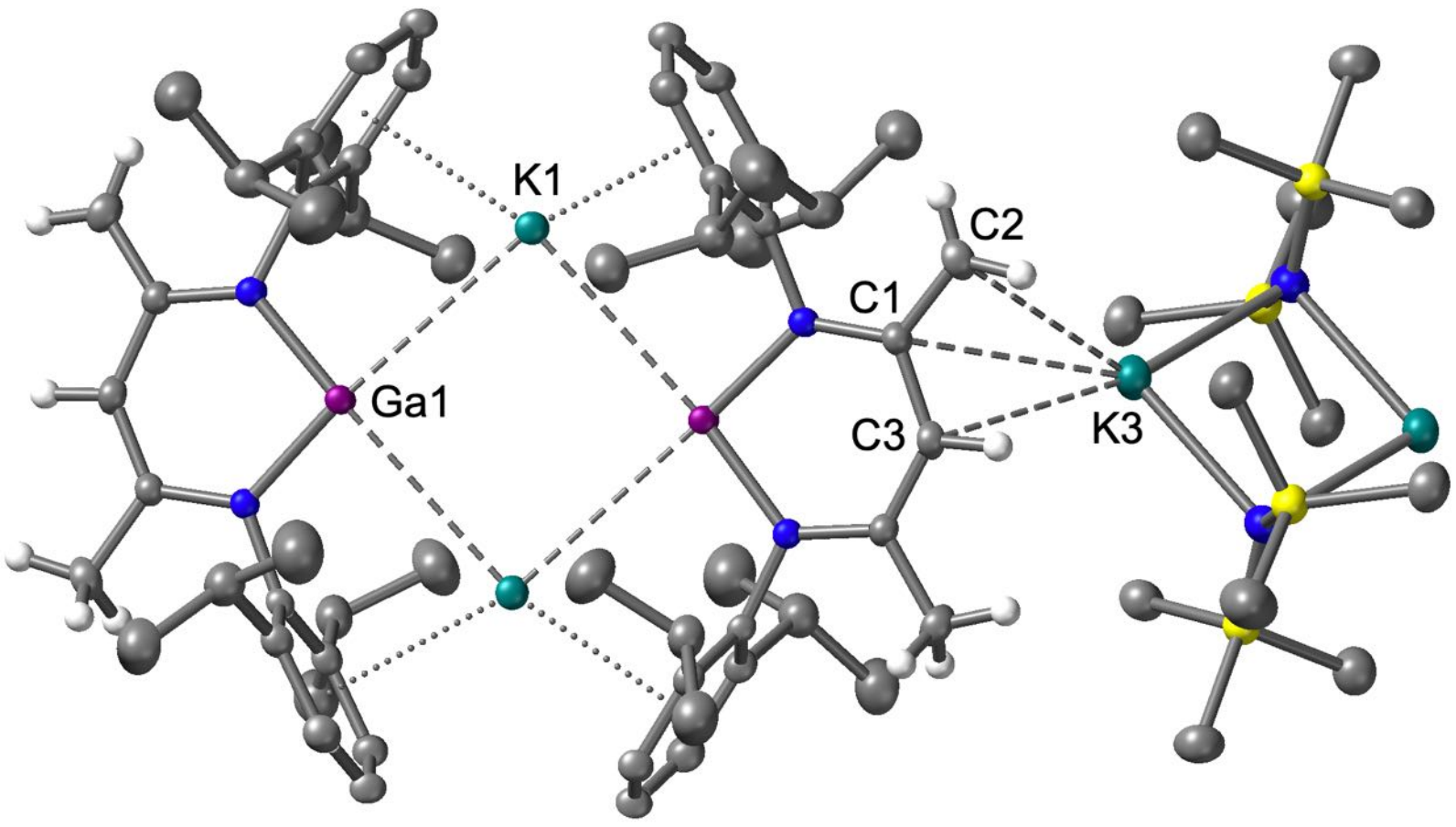

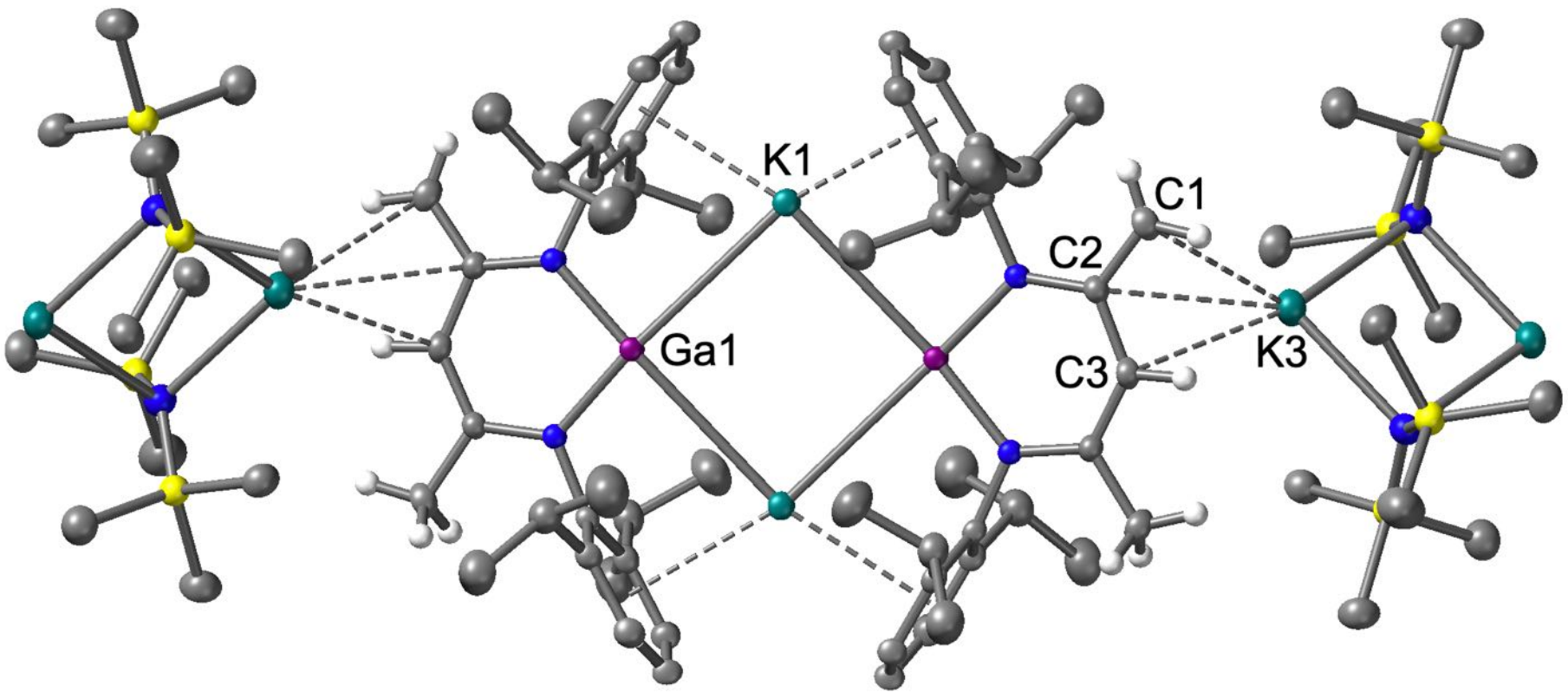

Figure S71. Molecular structure of [**(2·K)$_2$(KHMDS)$_2$**] with displacement ellipsoids at 50% probability. The top figure illustrates the configuration of the dimeric (**2·K**)$_2$ array interacting with (KHMDS)$_2$ dimers within the asymmetric unit. The bottom figure illustrates the chain growth of the dimeric (2·K)2 array via K···olefin(C1,C2,C3) interactions with neighboring (KHMDS)2 units. For clarity, all H atoms are omitted except those on the {C1,C5}-backbone. The selected bond lengths (Å) and angles (°) are as follows: K(3)···C(1) 3.340(2), K(3)···C(2) 3.489(2) , K(3)···C(3) 3.241(2).

S5.10 Crystal data and structure refinement for [(2·Cs)$_2$(CsHMDS)$_2$]$_\infty$ (CCDC 2391237)

Empirical formula	$C_{35}H_{58}Cs_2GaN_3Si_2$	
Formula weight	912.56	
Temperature	260(1) K	
Wavelength	0.71073 Å	
Crystal system	Triclinic	
Space group	*P*-1	
Unit cell dimensions	a = 10.2851(12) Å	α = 96.100(5)°
	b = 14.5783(16) Å	β = 104.251(5)°
	c = 14.8997(16) Å	γ = 91.477(6)°
Volume	2149.9(4) Å^3	
Z	2	
Density (calculated)	1.410 Mg/m^3	
Absorption coefficient	1.389 mm^{-1}	
F(000)	916	
Crystal size	0.18 x 0.16 x 0.14 mm^3	
Theta range for data collection	2.545 to 29.420	
Index ranges	-14<=h<=14, -20<=k<=20, -20<=l<=20	
Reflections collected	112384	
Independent reflections	12099 [R(int) = 0.0347]	
Completeness to theta = 25.242°	99.9 %	
Absorption correction	Semi-empirical from equivalents	
Max. and min. transmission	0.1402 and 0.1052	
Refinement method	Full-matrix least-squares on F^2	
Data / restraints / parameters	12099 / 0 / 411	
Goodness-of-fit on F^2	1.037	
Final R indices [I>2sigma(I)]	R1 = 0.0335, wR2 = 0.0734	
R indices (all data)	R1 = 0.0487, wR2 = 0.0813	
Extinction coefficient	n/a	
Largest diff. peak and hole	1.674 and -1.352 e.Å^{-3}	

Additional details for [(2·Cs)$_2$(CsHMDS)$_2$]$_\infty$: This compound crystallized in the triclinic space group *P-1*. Data collection was attempted on several crystals across a range of temperatures, including 100 K, 150 K, 200 K, 240 K, and 260 K. A phase transition was observed consistently in crystals measured below 240 K, so the optimal data set was collected at 260 K. The structure obtained at this temperature displayed good data quality and structural integrity, allowing for reliable metric analysis and discussion of bond lengths and angles.

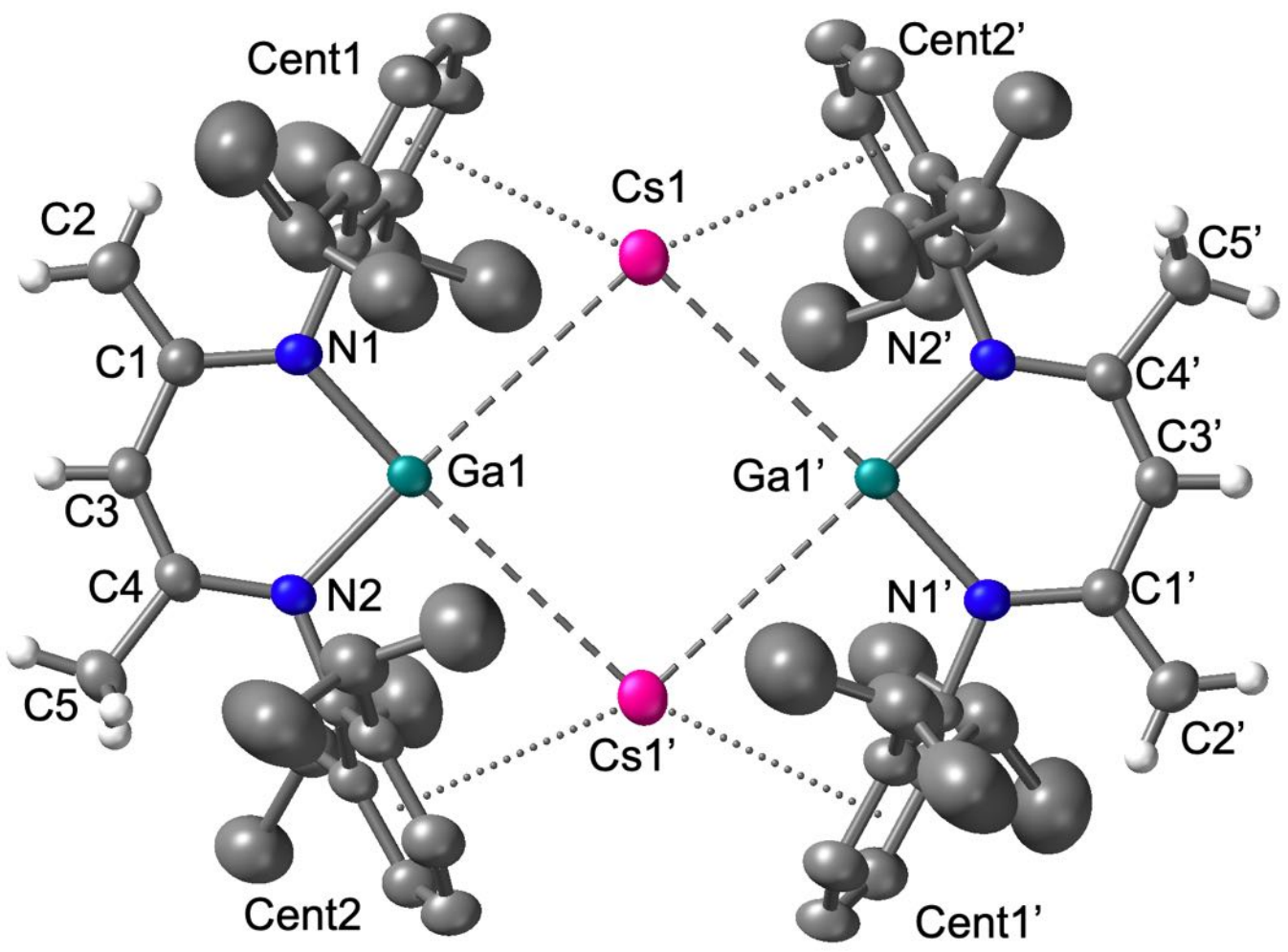

Figure S72. Molecular structure of [(**2·Cs**)$_2$**(CsHMDS)$_2$**] with displacement ellipsoids at 50% probability. The figure illustrates the configuration of the dimeric (**2·Cs**)$_2$ array present within the asymmetric unit. For clarity, all H atoms are omitted except those on the {C1,C5}-backbone. The selected bond lengths (Å) and angles (°) are as follows: Ga(1)-Cs(1) 3.8481(6), Ga(1)-Cs(1') 3.7860(6), Ga(1)-N(2) 1.9595(18), Ga(1)-N(1) 1.9394(18), Cs(1)···Cent1 3.254(1), Cs(1)···Cent2' 3.284(1), Cs(1')···Cent2 3.284(1), Cs(1')···Cent1' 3.254(1), N(2)-C(4) 1.376(3), N(1)-C(1) 1.389(3), C(2)-C(1) 1.377(3), C(1)-C(3) 1.439(3), C(3)-C(4) 1.373(3), C(4)-C(5) 1.491(3), Cs(1')-Ga(1)-Cs(1) 86.957(8), N(2)-Ga(1)-Cs(1) 164.63(6), N(2)-Ga(1)-Cs(1') 91.04(5), N(1)-Ga(1)-Cs(1) 90.06(5), N(1)-Ga(1)-Cs(1') 172.88(6). The symmetry transformation used to generate equivalent atoms labelled with (') is -x+1,-y+2,-z+1.

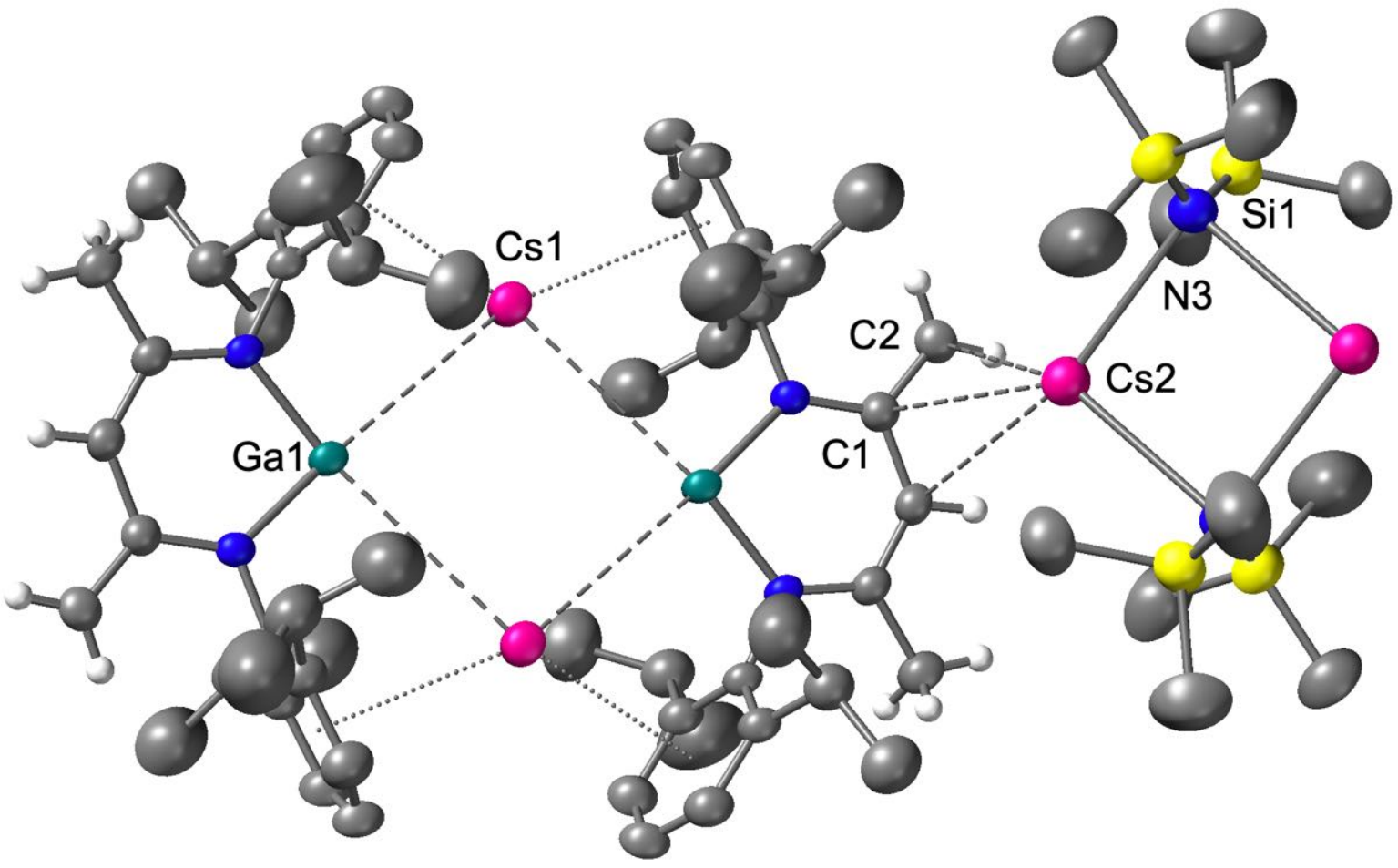

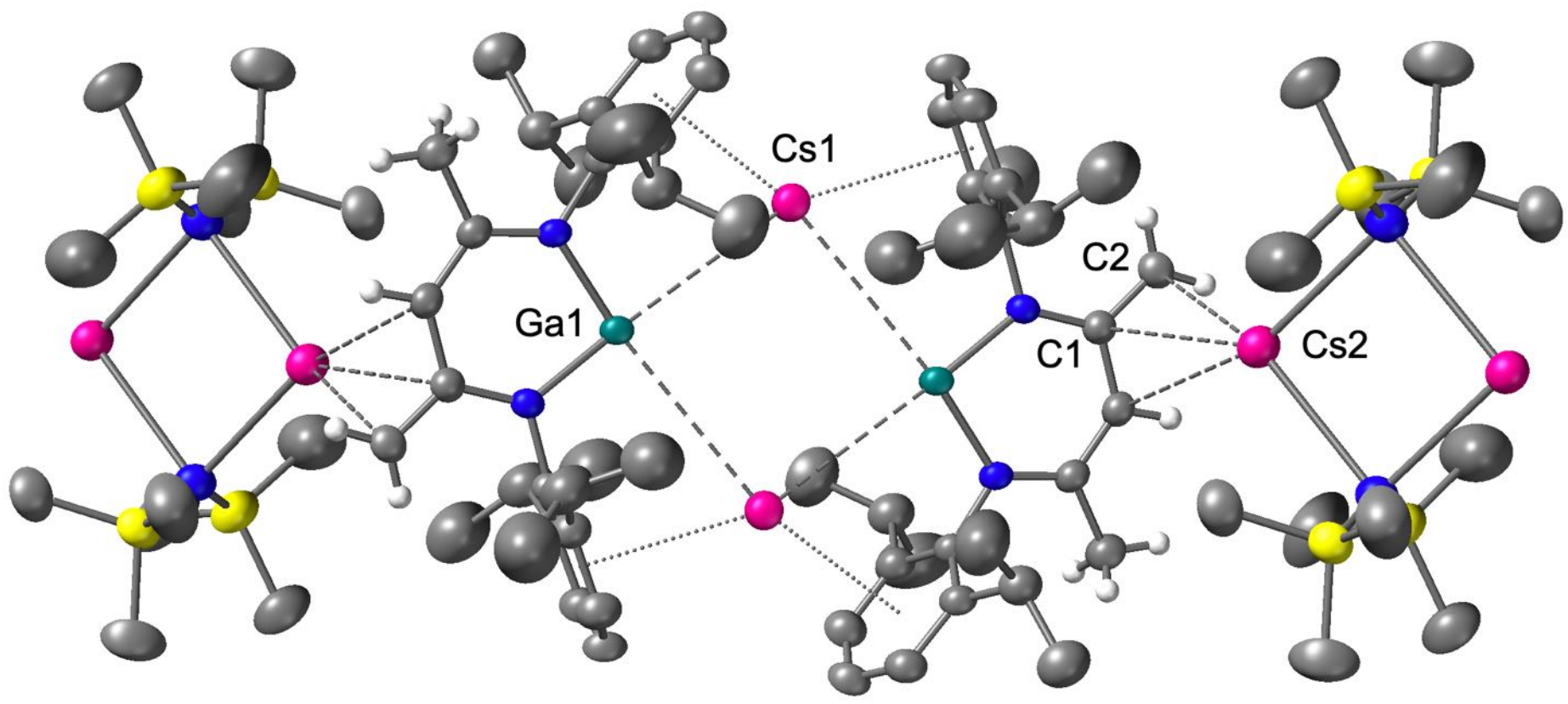

Figure S73. Molecular structure of [(**2·Cs)$_2$(CsHMDS)$_2$**] with displacement ellipsoids at 50% probability. The top figure illustrates the configuration of the dimeric (**2·Cs**)$_2$ array interacting with (CsHMDS)$_2$ dimers within the asymmetric unit. The bottom figure shows the growth of the dimeric (2·Cs)2 array through Cs···olefin(C1,C2,C3) interactions with adjacent (CsHMDS)2 units. For clarity, all H atoms are omitted except those on the {C1,C5}-backbone. The selected bond lengths (Å) and angles (°) are as follows: Cs(2)···C(1) 3.726(2), Cs(2)···C(2) 3.536(3), Cs(2)···C(3) 3.835(2).

S5.11 Crystal data and structure refinement for 3·(toluene)$_{0.5}$ (CCDC 2546880)

Empirical formula	$C_{59.5}H_{80}AuGaN_4$	
Formula weight	1117.96	
Temperature	150.00(10) K	
Wavelength	1.54184 Å	
Crystal system	Monoclinic	
Space group	*P 1 21/c 1*	
Unit cell dimensions	a = 13.3021(3) Å	α = 90°
	b = 21.0944(4) Å	β = 94.039(2)°
	c = 20.9403(4) Å	γ = 90°
Volume	5861.3(2) Å^3	
Z	4	
Density (calculated)	1.267 Mg/m^3	
Absorption coefficient	5.472 mm^{-1}	
F(000)	2300	
Crystal size	0.1 x 0.1 x 0.01 mm^3	
Theta range for data collection	3.331 to 70.073°	
Index ranges	-16<=h<=16, -25<=k<=24, -23<=l<=25	
Reflections collected	47571	
Independent reflections	11125 [R(int) = 0.0942]	
Completeness to theta = 25.242°	100.0 %	
Absorption correction	Semi-empirical from equivalents	
Max. and min. transmission	1.00000 and 0.58184	
Refinement method	Full-matrix least-squares on F^2	
Data / restraints / parameters	11125 / 136 / 640	
Goodness-of-fit on F^2	1.037	
Final R indices [I>2sigma(I)]	R1 = 0.0540, wR2 = 0.1354	
R indices (all data)	R1 = 0.0743, wR2 = 0.1486	
Extinction coefficient	n/a	
Largest diff. peak and hole	2.612 and -1.117 e.Å^{-3}	

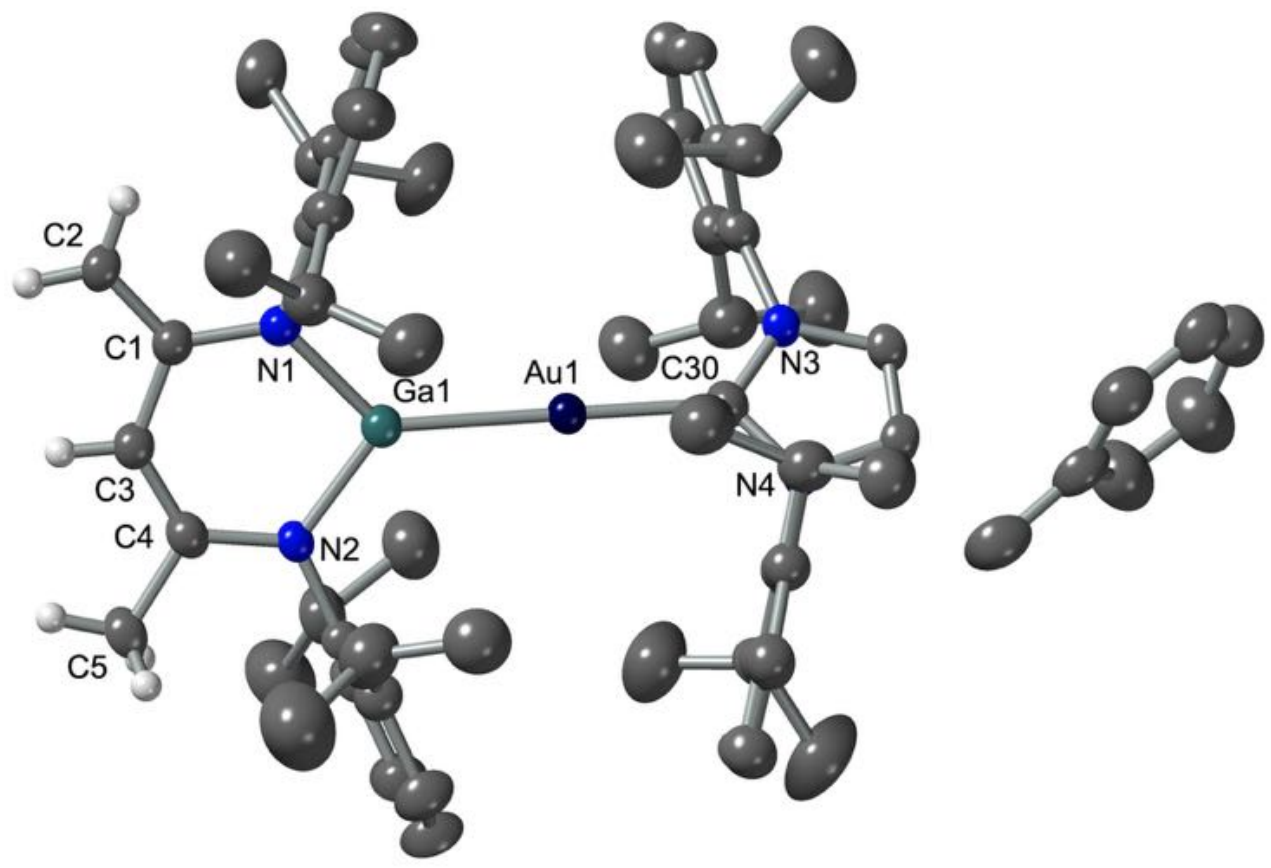

Figure S74. Molecular structure of **3·(toluene)$_{0.5}$** showing the contents of the asymmetric unit with displacement ellipsoids at 50% probability. Within the asymmetric unit half molecule of disordered toluene solvent was also found. For clarity, all H atoms are omitted except those on the {C1,C5}-backbone. The selected bond lengths (Å) and angles (°) are as follows: Ga(1)-Au(1) 2.3874(8), Ga(1)-N(1) 1.878(5), Ga(1)-N(2) 1.879(5), Au(1)-C(30) 2.068(6), N(1)-C(1) 1.399(8), N(1)-C(6) 1.413(8), N(2)-C(4) 1.393(8), C(1)-C(2) 1.364(9), C(1)-C(3) 1.457(9), C(3)-C(4) 1.363(9), C(4)-C(5) 1.503(9), Ga(1)-Au(1)-C(30) 178.27(16), N(1)-Ga(1)-Au(1) 130.91(16), N(1)-Ga(1)-N(2) 100.2(2), N(2)-Ga(1)-Au(1) 128.89(15), N(4)-C(30)-N(3) 104.6(5).

S5.12 Crystal data and structure refinement for 3·benzene (CCDC 2546879)

Empirical formula	$C_{62}H_{82}AuGaN_4$	
Formula weight	1150.00	
Temperature	150.00(10) K	
Wavelength	1.54184 Å	
Crystal system	Orthorhombic	
Space group	*P212121*	
Unit cell dimensions	a = 14.03021(5) Å	α = 90°
	b = 17.71221(6) Å	β = 90°
	c = 23.00208(8) Å	γ = 90°
Volume	5716.15(3) $Å^3$	
Z	4	
Density (calculated)	1.336 Mg/m^3	
Absorption coefficient	5.627 mm^{-1}	
F(000)	2368	
Crystal size	0.1 x 0.1 x 0.01 mm^3	
Theta range for data collection	3.149 to 79.393°	
Index ranges	-17<=h<=17, -22<=k<=22, -29<=l<=29	
Reflections collected	251195	
Independent reflections	12385 [R(int) = 0.0328]	
Completeness to theta = 25.242°	100.0 %	
Absorption correction	Semi-empirical from equivalents	
Max. and min. transmission	1.00000 and 0.62822	
Refinement method	Full-matrix least-squares on F^2	
Data / restraints / parameters	12385 / 346 / 694	
Goodness-of-fit on F^2	1.115	
Final R indices [I>2sigma(I)]	R1 = 0.0142, wR2 = 0.0355	
R indices (all data)	R1 = 0.0142, wR2 = 0.0355	
Extinction coefficient	n/a	
Largest diff. peak and hole	0.750 and -0.302 e.$Å^{-3}$	

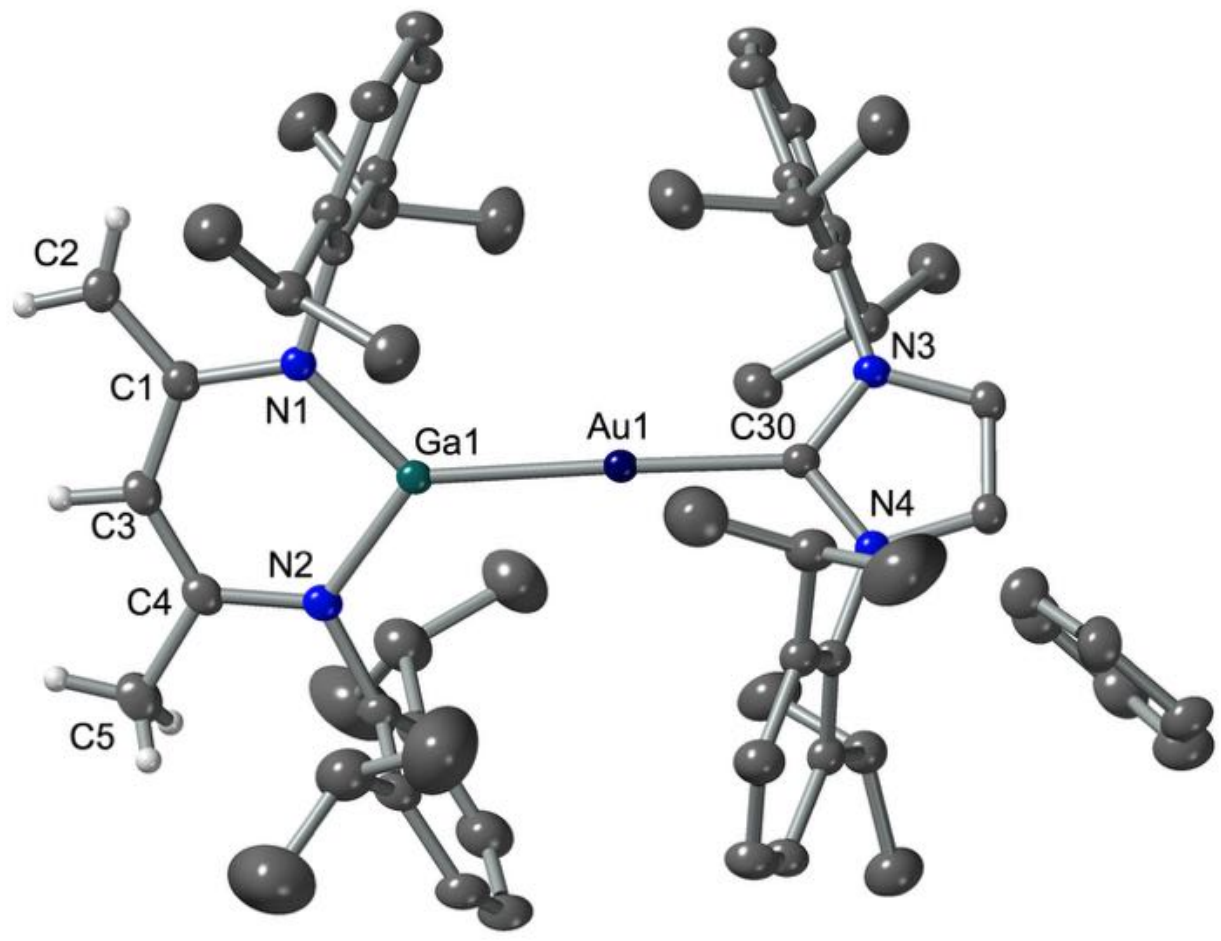

Figure S75. Molecular structure of **3·benzene** showing the contents of the asymmetric unit with displacement ellipsoids at 50% probability. Within the asymmetric unit one disordered molecule of benzene solvent was also found, only the major disordered component is shown. For clarity, all H atoms are omitted except those on the {C1,C5}-backbone. The selected bond lengths (Å) and angles (°) are as follows: Ga(1)-Au(1) 2.3855(3), Ga(1)-N(1) 1.887(2), Ga(1)-N(2) 1.880(2), Au(1)-C(30) 2.073(2), N(1)-C(1) 1.393(4), N(2)-C(4) 1.398(3), C(1)-C(2) 1.469(4), C(1)-C(3) 1.376(4), C(3)-C(4) 1.427(4), C(4)-C(5) 1.394(4), Ga(1)-Au(1)-C(30) 176.44(7), Au(1)-Ga(1)-N(2) 131.12(7), N(1)-Ga(1)-N(2) 99.94(10), N(1)-Ga(1)-Au(1) 128.80(7), N(3)-C(30)-N(4) 104.8(2).

S5.13 Crystal data and structure refinement for 4 (CCDC 2502503)

Empirical formula	$C_{63}H_{87}AuGaN_5$	
Formula weight	1181.06	
Temperature	150.00(10) K	
Wavelength	1.54184 Å	
Crystal system	Monoclinic	
Space group	*P 1 21/c 1*	
Unit cell dimensions	a = 19.77337(5) Å	α = 90°
	b = 14.33568(4) Å	β = 106.2663(3)°
	c = 21.51151(7) Å	γ = 90°
Volume	5853.66(3) $Å^3$	
Z	4	
Density (calculated)	1.340 Mg/m^3	
Absorption coefficient	5.513 mm^{-1}	
F(000)	2440	
Crystal size	0.27 x 0.208 x 0.067 mm^3	
Theta range for data collection	2.328 to 79.590°	
Index ranges	-25<=h<=22, -18<=k<=18, -27<=l<=27	
Reflections collected	249769	
Independent reflections	12655 [R(int) = 0.0724]	
Completeness to theta = 25.242°	100.0 %	
Absorption correction	Semi-empirical from equivalents	
Max. and min. transmission	1.00000 and 0.51057	
Refinement method	Full-matrix least-squares on F^2	
Data / restraints / parameters	12655 / 413 / 722	
Goodness-of-fit on F^2	1.094	
Final R indices [I>2sigma(I)]	R1 = 0.0299, wR2 = 0.0813	
R indices (all data)	R1 = 0.0301, wR2 = 0.0814	
Extinction coefficient	n/a	
Largest diff. peak and hole	1.531 and -2.021 e.$Å^{-3}$	

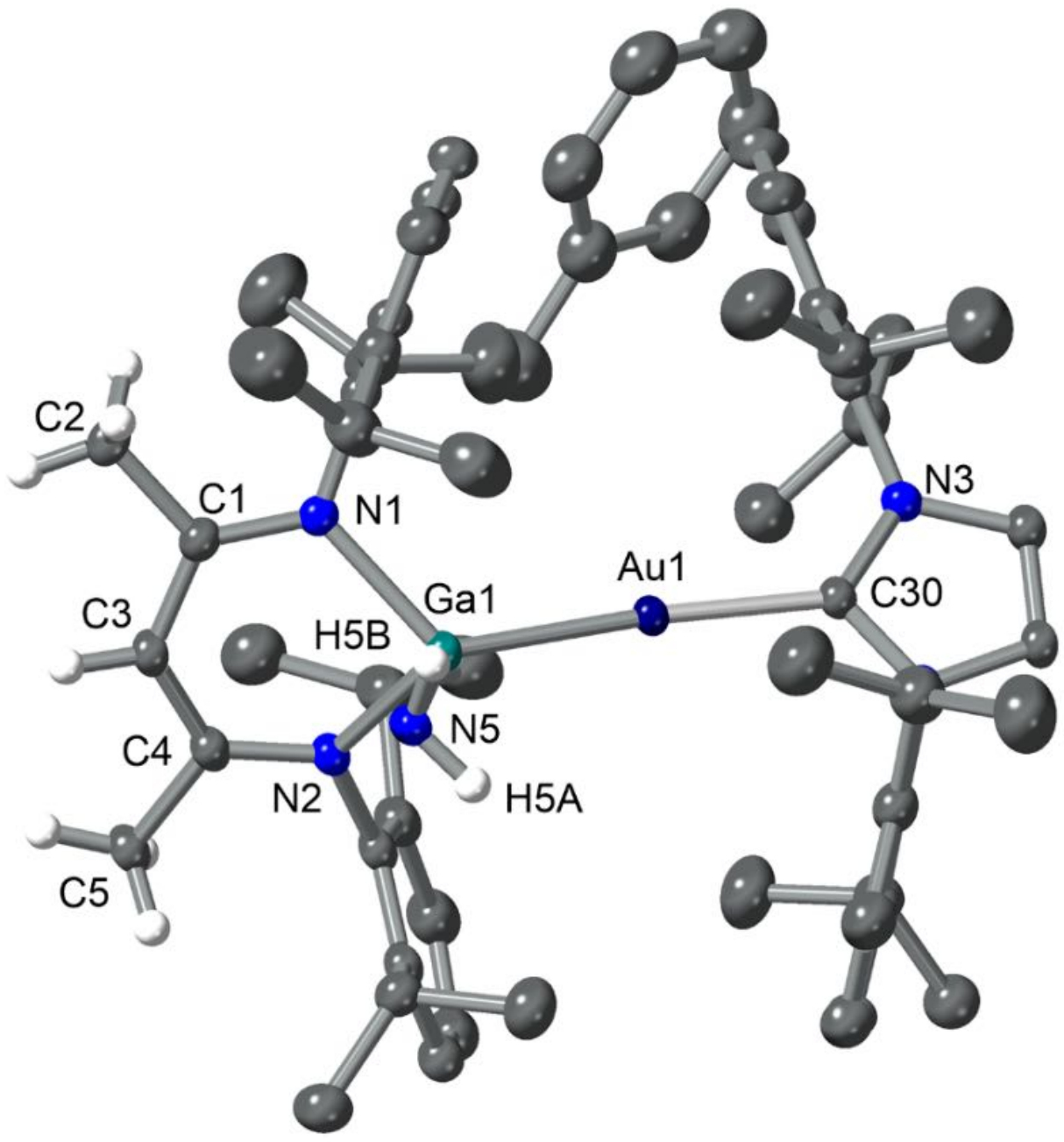

Figure S76. Molecular structure of **4** showing the contents of the asymmetric unit with displacement ellipsoids at 50% probability. Within the asymmetric unit one molecule of toluene solvent was also found. For clarity, all H atoms are omitted except those on the {C1,C5}-backbone. The selected bond lengths (Å) and angles (°) are as follows: Ga(1)-Au(1) 2.4052(3), Ga(1)-N(5) 1.920(2), N(5)-H5A 0.937(10), N(5)-H5B 0.942(10), Ga(1)-N(1) 2.018(2), Ga(1)-N(2) 2.0169(19), Au(1)-C(30) 2.094(2), N(1)-C(1) 1.322(3), N(2)-C(4) 1.329(3), C(2)-C(1) 1.512(3), C(1)-C(3) 1.411(3), C(3)-C(4) 1.398(3), C(4)-C(5) 1.518(3), Ga(1)-Au(1)-C(30) 173.01(6), N(1)-Ga(1)-N(2) 91.97(8), N(3)-C(30)-N(4) 104.7(2).

S5.14 Crystal data and structure refinement for 5 (CCDC 2502504)

Empirical formula	$C_{63}H_{86}AuGaN_4O$	
Formula weight	1182.04	
Temperature	150.00(10) K	
Wavelength	1.54184 Å	
Crystal system	Monoclinic	
Space group	*P 1 21/c 1*	
Unit cell dimensions	a = 19.72422(6) Å	a= 90°
	b = 14.37523(4) Å	b= 105.9054(3)°
	c = 21.35854(6) Å	g = 90°
Volume	5824.15(3) $Å^3$	
Z	4	
Density (calculated)	1.348 Mg/m^3	
Absorption coefficient	5.550 mm^{-1}	
F(000)	2440	
Crystal size	0.27 x 0.208 x 0.067 mm^3	
Theta range for data collection	2.329 to 77.253°.	
Index ranges	-24<=h<=23, -18<=k<=17, -26<=l<=27	
Reflections collected	125850	
Independent reflections	12260 [R(int) = 0.0316]	
Completeness to theta = 67.684°	100.0 %	
Absorption correction	Semi-empirical from equivalents	
Max. and min. transmission	1.00000 and 0.76666	
Refinement method	Full-matrix least-squares on F^2	
Data / restraints / parameters	12260 / 405 / 718	
Goodness-of-fit on F2	1.124	
Final R indices [I>2sigma(I)]	R1 = 0.0199, wR2 = 0.0474	
R indices (all data)	R1 = 0.0201, wR2 = 0.0475	
Extinction coefficient	n/a	
Largest diff. peak and hole	0.394 and -0.607 e.$Å^{-3}$	

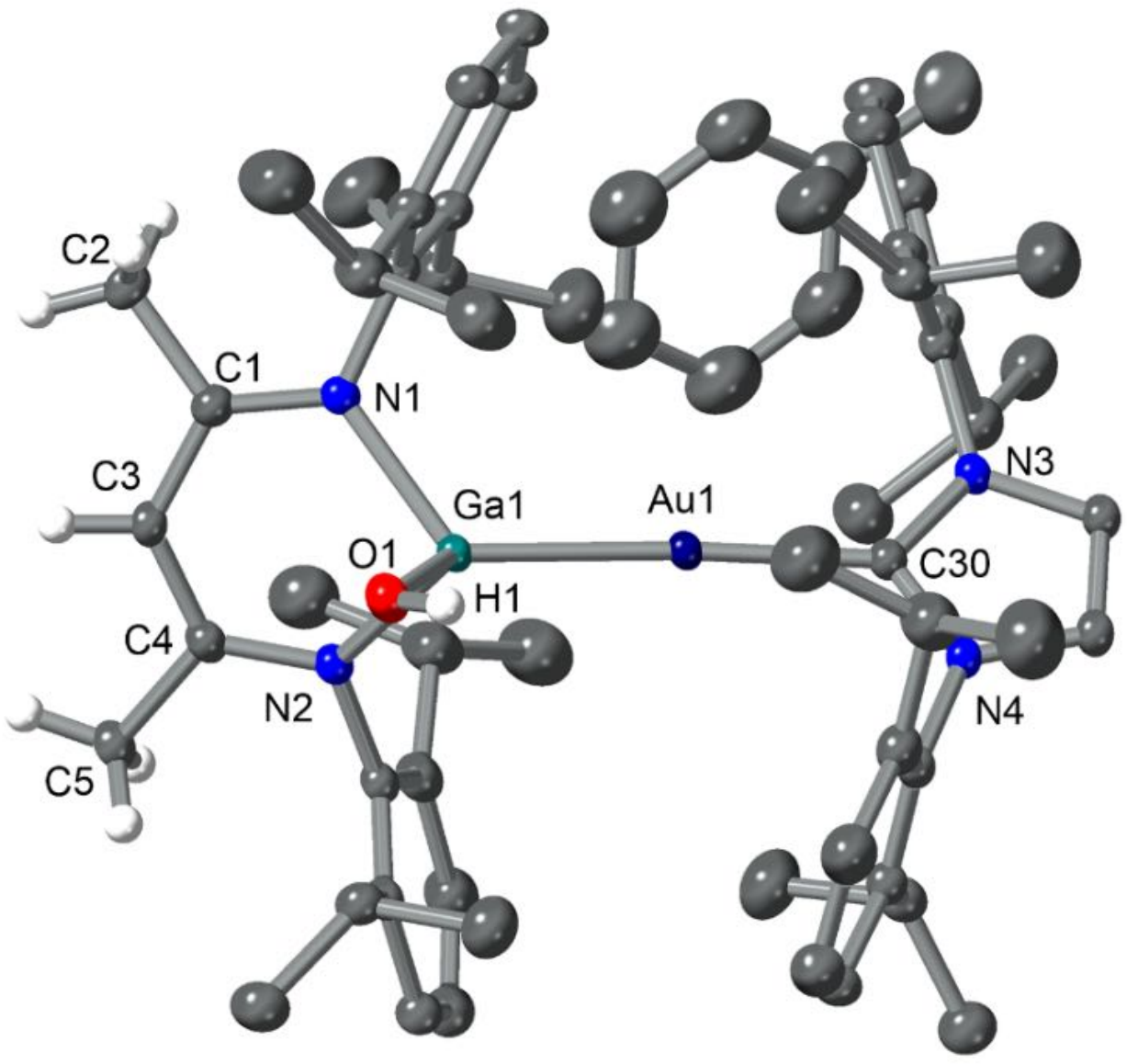

Figure S77. Molecular structure of **5** showing the contents of the asymmetric unit with displacement ellipsoids at 50% probability. Within the asymmetric unit one molecule of toluene solvent was also found. For clarity, all H atoms are omitted except those on the {C1,C5}-backbone. The selected bond lengths (Å) and angles (°) are as follows: Ga(1)-Au(1) 2.4045(2), Ga(1)-O(1) 1.8768(13), O(1)-H(1) 0.827(10), Ga(1)-N(1) 2.0093(15), Ga(1)-N(2) 2.0078(14), Au(1)-C(30) 2.0880(18), N(1)-C(1) 1.323(2), N(2)-C(4) 1.331(2), C(2)-C(1) 1.511(3), C(1)-C(3) 1.409(3), C(3)-C(4) 1.397(3), C(4)-C(5) 1.515(2), Ga(1)-Au(1)-C(30) 174.53(5), N(1)-Ga(1)-N(2) 91.80(6), N(3)-C(30)-N(4) 104.29(15).

S5.15 Crystal data and structure refinement for 6 (CCDC 2546881)

Empirical formula	$C_{57}H_{81}AuGaN_5$	
Formula weight	1102.95	
Temperature	150.00(10) K	
Wavelength	1.54184 Å	
Crystal system	Monoclinic	
Space group	*P 1 21/n 1*	
Unit cell dimensions	a = 10.83520(10) Å	α = 90°
	b = 22.13220(10) Å	β = 93.2260(10)°
	c = 23.05590(10) Å	γ = 90°
Volume	5520.20(6) Å^3	
Z	4	
Density (calculated)	1.327 Mg/m^3	
Absorption coefficient	5.806 mm^{-1}	
F(000)	2272	
Crystal size	0.14 x 0.1 x 0.1 mm^3	
Theta range for data collection	2.770 to 79.814°	
Index ranges	-13<=h<=13, -28<=k<=27, -29<=l<=25	
Reflections collected	147217	
Independent reflections	11918 [R(int) = 0.0553]	
Completeness to theta = 25.242°	100.0 %	
Absorption correction	Semi-empirical from equivalents	
Max. and min. transmission	1.00000 and 0.43647	
Refinement method	Full-matrix least-squares on F^2	
Data / restraints / parameters	11918 / 0 / 601	
Goodness-of-fit on F^2	1.033	
Final R indices [I>2sigma(I)]	R1 = 0.0270, wR2 = 0.0713	
R indices (all data)	R1 = 0.0281, wR2 = 0.0721	
Extinction coefficient	n/a	
Largest diff. peak and hole	1.459 and -1.193 e.Å^{-3}	

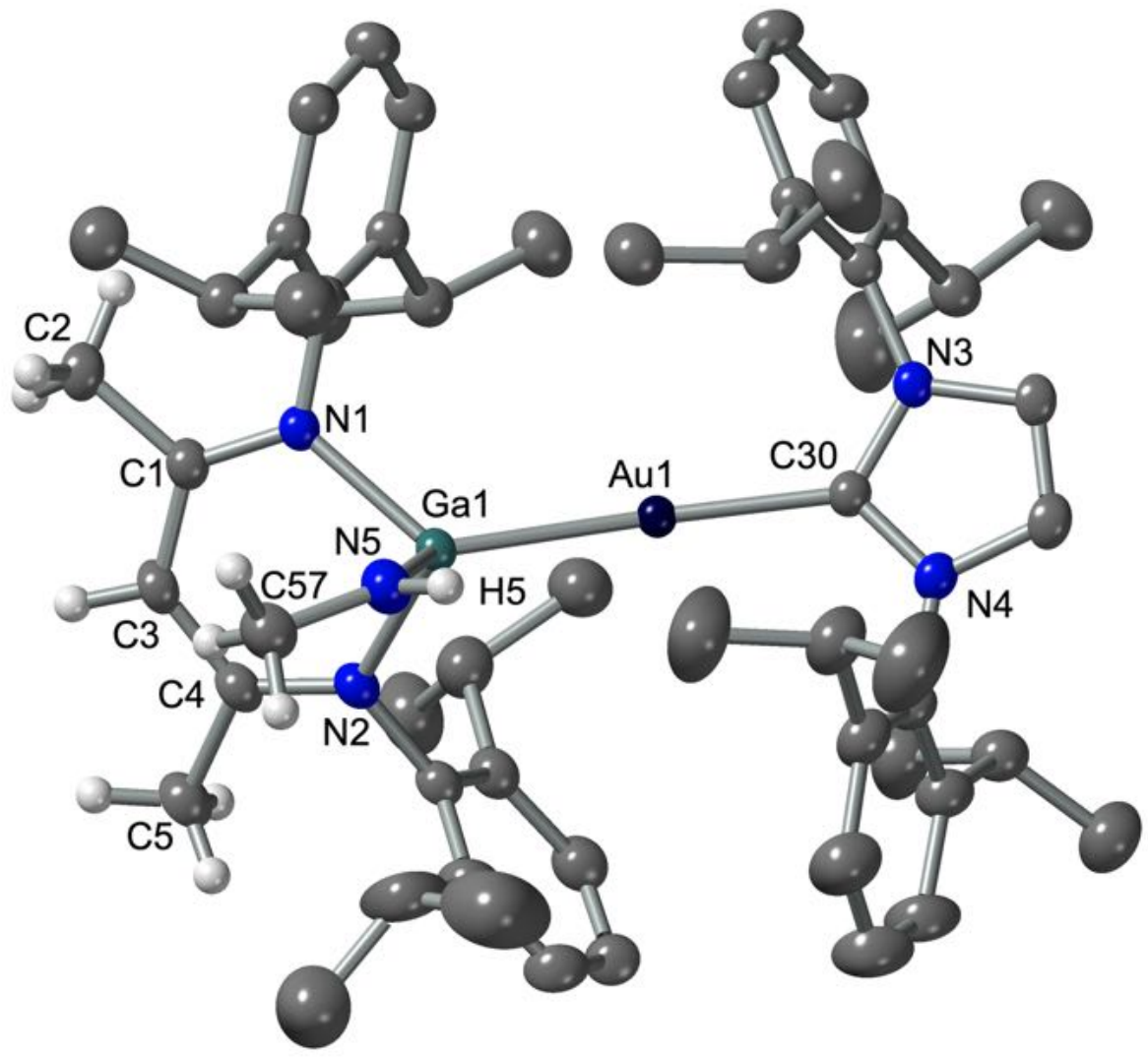

Figure S78. Molecular structure of **6** showing the contents of the asymmetric unit with displacement ellipsoids at 50% probability. For clarity, all H atoms are omitted except those on the {C1,C5}-backbone. The selected bond lengths (Å) and angles (°) are as follows: Ga(1)-Au(1) 2.4203(3), Au(1)-C(30) 2.098(2), Ga(1)-N(2) 2.0300(18), Ga(1)-N(1) 2.0407(18), Ga(1)-N(5) 1.883(2), N(1)-C(1) 1.325(3), N(2)-C(4) 1.327(3), N(5)-C(57) 1.443(3), N(5)-H(5) 0.91(4), C(1)-C(2) 1.514(3), C(1)-C(3) 1.407(3), C(3)-C(4) 1.397(3), C(4)-C(5) 1.518(3), C(30)-Au(1)-Ga(1) 169.09(6), N(2)-Ga(1)-Au(1) 116.72(5), N(2)-Ga(1)-N(1) 90.81(8), N(1)-Ga(1)-Au(1) 125.42(5), N(5)-Ga(1)-Au(1) 115.25(7), N(5)-Ga(1)-N(2) 104.14(9), N(5)-Ga(1)-N(1) 100.20(9).

S5.16 Crystal data and structure refinement for 7 (CCDC 2546882)

Empirical formula	$C_{64}H_{88}AuGaN_4O$	
Formula weight	1196.07	
Temperature	150.01(12) K	
Wavelength	1.54184 Å	
Crystal system	Monoclinic	
Space group	*P 1 21/n 1*	
Unit cell dimensions	a = 13.70950(10) Å	α = 90°
	b = 24.2747(2) Å	β = 97.0760(10)°
	c = 18.22290(10) Å	γ = 90°
Volume	6018.28(8) $Å^3$	
Z	4	
Density (calculated)	1.320 Mg/m^3	
Absorption coefficient	5.377 mm^{-1}	
F(000)	2472	
Crystal size	0.1 x 0.1 x 0.05 mm^3	
Theta range for data collection	3.047 to 70.069°	
Index ranges	-16<=h<=16, -29<=k<=29, -22<=l<=22	
Reflections collected	246249	
Independent reflections	11424 [R(int) = 0.1570]	
Completeness to theta = 25.242°	100.0 %	
Absorption correction	Semi-empirical from equivalents	
Max. and min. transmission	1.00000 and 0.65904	
Refinement method	Full-matrix least-squares on F^2	
Data / restraints / parameters	11424 / 2576 / 1097	
Goodness-of-fit on F^2	1.009	
Final R indices [I>2sigma(I)]	R1 = 0.0364, wR2 = 0.0959	
R indices (all data)	R1 = 0.0385, wR2 = 0.0977	
Extinction coefficient	n/a	
Largest diff. peak and hole	1.896 and -1.271 e.$Å^{-3}$	

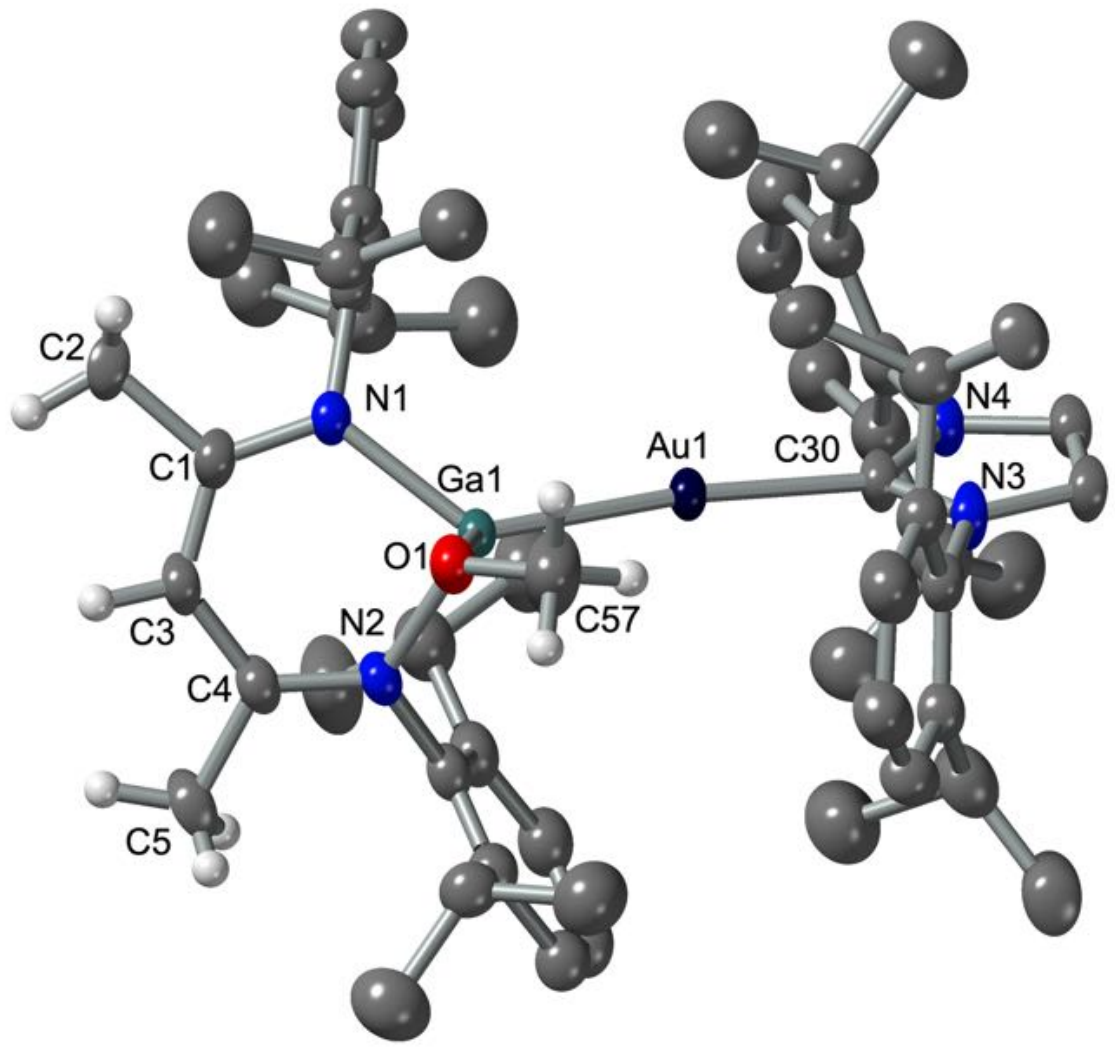

Figure S79. Molecular structure of **7** showing the contents of the asymmetric unit with displacement ellipsoids at 50% probability. Within the asymmetric unit one molecule of disordered toluene solvent was also found. For clarity, disordered Dipp and IPr groups, and all H atoms are omitted except those on the {C1,C5}-backbone. The selected bond lengths (Å) and angles (°) are as follows: Au(1)-Ga(1) 2.4165(4), Au(1)-C(30) 2.103(5), Ga(1)-O(1) 1.875(2), Ga(1)-N(1) 2.015(3), Ga(1)-N(2) 2.018(3), O(1)-C(57) 1.378(5), N(1)-C(1) 1.326(4), N(2)-C(4) 1.318(4), C(1)-C(2) 1.514(5), C(1)-C(3) 1.392(5), C(3)-C(4) 1.396(5), C(4)-C(5) 1.518(5), Ga(1)-Au(1)-C(30) 170.9(2), N(1)-Ga(1)-Au(1) 125.79(7), N(1)-Ga(1)-N(2) 91.28(11), N(2)-Ga(1)-Au(1) 120.36(7), N(3)-C(30)-Au(1) 124.6(6), N(4)-C(30)-Au(1) 131.8(6), N(4)-C(30)-N(3) 103.0(5).

Note: H atoms on the NH_2 and OH groups on the crystal structures of compounds **4**, **5** and **6** were found in the difference Fourier map and freely refined to maintain sensible geometries, with N5–H5A and N5–H5B bond distances of 0.937(10) and 0.942(10) Å in **4**, respectively; O1–H1 of 0.827(10) Å in **5**, and N5-H5 for 0.91(4) Å in **6. Figure S80** shows the residual electron density peaks attributed to both H atoms on the NH_2 group in **4**, the H atom on the OH group in **5**, and the H atom on the MeNH group in **6.** NH_2, OH and MeNH groups are also observed by ^{1}H NMR spectroscopy (Tol-d_8), a broad singlet at δ –0.05 ppm (2H) for the NH2 group in **4**, and a single resonance for the OH group at δ –0.26 ppm (1H) in **5**, and a broad quartet at δ –0.18 (1H) for the NH site of the MeNH in **6**.

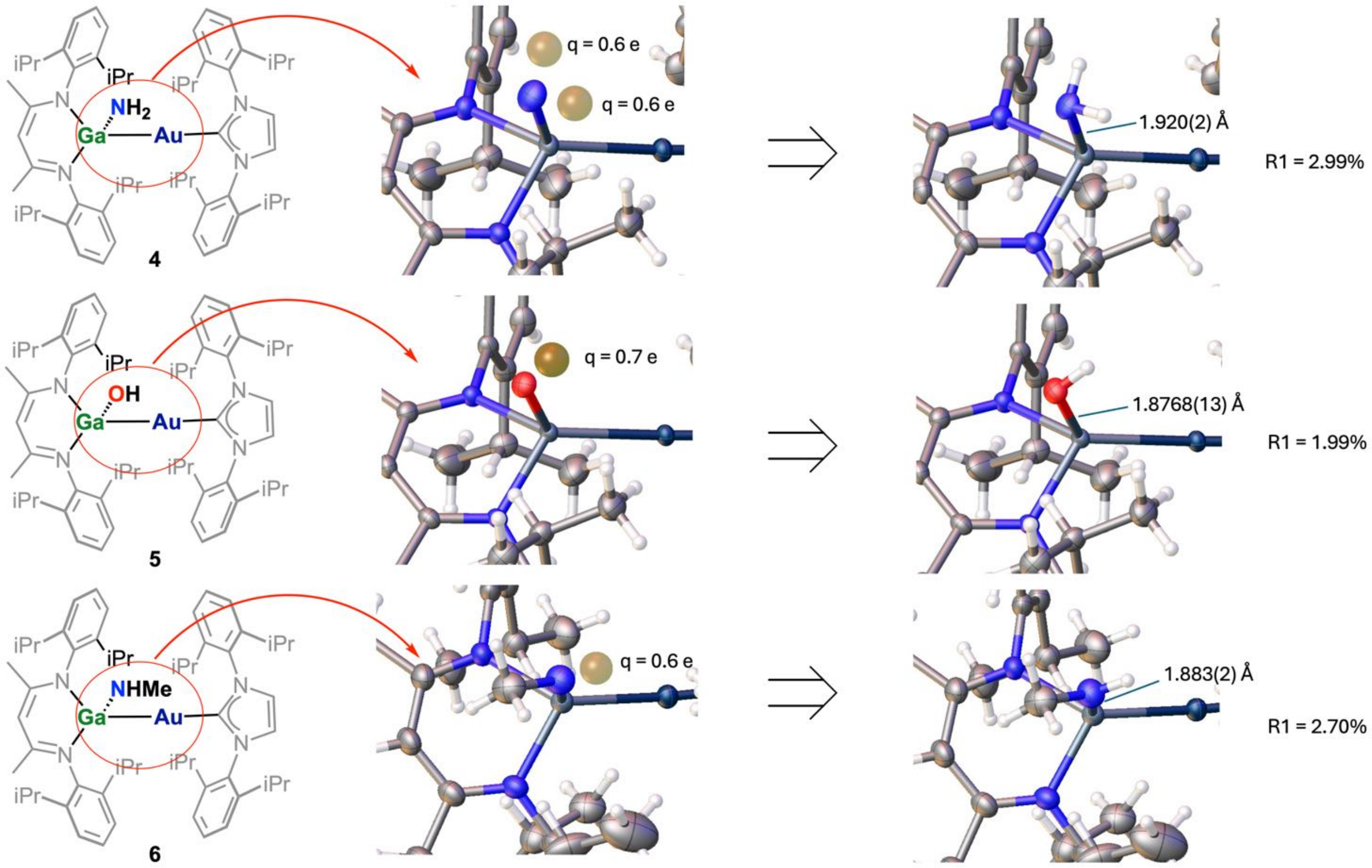

Figure S80. Crystallographic study of **4**, **5** and **6** and NH_2, OH and NH (MeNH) assignments from the residual electron density analysis from their single-crystal structures. Hydrogen atoms on the NH_2, OH and NH (MeNH) groups of **4**, **5** and **6**, respectively, were located in the difference Fourier map and freely refined.

S6 Computational Methods

All electronic structure calculations were carried out using the Gaussian 16 (G16 revision C.01), Amsterdam Density Functional 2022.102 (ADF), ORCA (Version 6.1.0) and CP2K (Version 7.1) program packages.[26-28] Unconstrained optimizations of ground-state geometries and subsequent analytical frequency calculations were carried out using G16 at the DFT level using the PBE GGA exchange correlation functional[29] in conjunction with Ahlrich's def2-TZVP basis set on Au (including corresponding ECP), Al, Ga, Na, K and Cs atoms and def2-SV(P) on C, N, O and H atoms.[30-31] Effects due to dispersion were accounted though Grimme's D3 dispersion correction including Becke-Johnson damping.[32] Exceptions include the contacted ion pair species, which are discussed in section S6.1.

An ultrafine integration grid, corresponding to a pruned grid of 99 radial shells and 590 angular points per shell, was used for all calculations. All stationary points were confirmed to be minima by the absence of any imaginary modes or the presence of exactly one imaginary mode for transition states in their vibrational spectra. Transition states were connected to their nearest minima by geometry optimizations along the displacement vector of the reaction coordinate in forward/reverse direction. Frequency calculations also provided thermal and entropic corrections to the total energy in gas phase at T = 298.15 K and p = 1 atm within the rigid-rotor/harmonic oscillator (RRHO) approximation. Single-point calculations for accurate energies and electronic structure analysis were performed using the PBE0 hybrid exchange correlation functional,[32] corrected for dispersion effects using Grimme's D3BJ correction in conjunction with Ahlrich's basis set of either def2-QZVP (NBO etc), or def2-TZVP (mechanism) quality on all atoms. Solvent effects on relative energies and barriers within the mechanisms were accounted for by the SMD model with solvent parameters corresponding to those of benzene.[32] Energies were adjusted for a standard state of 1 mol/L.

Energy decomposition analysis (EDA) calculations were carried out using ADF at the PBE0/TZP[33] level of theory, employing Grimme's D4 dispersion correction[34] and an all-electron basis set. Alternatively, ORCA was used in combination with PBE0-D3(BJ)/def2-TZVP. The NBO program (version 6.0) was used to perform Natural Bond Orbital analyses on the optimized structures.[35] The topology of the electron density was analyzed using QTAIM (quantum theory of atoms in molecules),[36] as implemented in the AIMALL package.[37]

Geometry optimizations of selected geometries in the solid state were carried out employing the Gaussian Plane Wave (GPW) formalism as implemented in the QUICKSTEP module of CP2K.[38] Molecularly optimized basis sets of double-ζ quality plus polarization in their short-range variant (DZVPMOLOPT-SR-GTH) were used on all atomic species.[39] The interaction between the core electrons and the valence shell was described by Goedecker-Teter-Hutter (GTH) pseudo potentials.[40-42] The PBE GGA exchange correlation functional was used in combination with Grimme's D3-correction for dispersion interactions. The auxiliary plane wave basis set was truncated

at a cutoff of 500 Ry. The maximum force convergence criterion was set to 10^{-4} $E_h\,a_0^{-1}$, whilst default values were used for the remaining criteria. The convergence criterion for the self-consistent field (SCF) accuracy was set to 10^{-7} E_h and 10^{-8} E_h for geometry optimizations and vibrational analysis, respectively. Non-covalent interaction plots were obtained using the NCIPLOT program,[43-44] and rendered using the VMD program.[45]

S6.1 Optimized geometries

Initial geometry optimizations of two reference structures, [**2**]·**K(crypt-222)** and (**2·K**)$_2$, were performed to assess the performance of the chosen DFT functional in reproducing key structural parameters with respect to their X-ray crystallographic counterparts. Particularly for **2·K(crypt-222)** inclusion of Grimme's D3 dispersion correction leads to a more contracted geometry, with the Ga···K contact substantially shortened (4.45 Å *cf.* expt. 5.15 Å). This indicates that the dispersion is likely considerably overestimated in these types of complexes. A much more balanced description is achieved by omitting the D3 correction. This is also seen in the structure of **(2·K)$_2$**, although the differences are more subtle. Similar protocols omitting D3-corrections for related systems have been reported in the literature.[46] Dispersion corrections were however included in all geometry optimizations involving the gold-coordinated complexes **3-5** and their mechanistic studies.

Table S12. Comparison of selected benchmark experimental and calculated bond parameters (in Å) in **2·K(crypt-222)** and **(2·K)$_2$**.

**Structure**	**Parameter**	**X-ray**	**DFT**			
			PBE-D3(BJ)	PBE	SMD-PBE-D3(BJ)	PBE (periodic)
**2·K(crypt-222)**	Ga···K	5.154	4.450	5.219	5.001	5.119
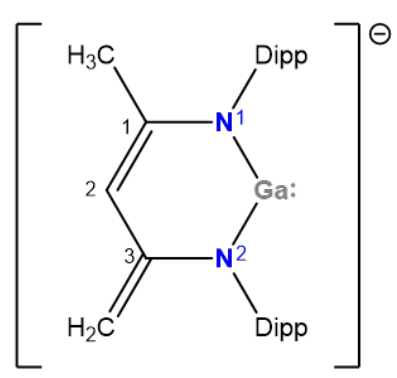	Ga–N1	1.992	2.002	2.039	2.003	2.084
	Ga–N2	1.986	2.039	2.001	2.005	2.047
	N1–C1	1.383	1.390	1.391	1.380	1.376
	N2–C3	1.386	1.405	1.404	1.396	1.386
	C1–C2	1.388	1.377	1.379	1.383	1.375
	C2–C3	1.398	1.458	1.461	1.460	1.452
	C3–CH_2	1.420	1.383	1.384	1.388	1.382
(**2·K**)$_2$	Ga1···Ga2	4.956	4.934	5.093	4.825	
	K1···K2	4.866	4.681	4.891	4.783	
	Ga1···K1	3.468	3.400	3.535	3.408	
	Ga1···K2	3.478	3.401	3.257	3.386	
	Ga2···K1	3.468	3.399	3.535	3.408	
	Ga2···K2	3.478	3.402	3.527	3.390	
	K1···Dipp1 Centroid	2.921	3.076	3.001	3.042	
	K1···Dipp2 Centroid	2.921	3.099	3.001	2.928	
	K2···Dipp3 Centroid	2.894	2.965	3.047	3.042	
	K2···Dipp4 Centroid	2.894	2.963	3.047	2.928	
	Unit 1 (Ga–N1)	1.945	1.985	1.993	1.985	
	Unit 1 (Ga–N2)	1.938	1.952	1.960	1.962	
	Unit 1 (N1–C1)	1.380	1.392	1.393	1.384	
	Unit 1 (N2–C3)	1.389	1.404	1.406	1.398	
	Unit 1 (C1–C2)	1.385	1.377	1.378	1.381	
	Unit 1 (C2–C3)	1.435	1.463	1.464	1.463	
	Unit 1 (C3–CH_2)	1.394	1.378	1.380	1.383	
	Unit 2 (Ga–N1)	1.945	1.985	1.993	1.985	
	Unit 2 (Ga–N2)	1.938	1.952	1.960	1.962	
	Unit 2 (N1–C1)	1.380	1.392	1.393	1.384	
	Unit 2 (N2–C3)	1.389	1.404	1.406	1.398	
	Unit 2 (C1–C2)	1.385	1.377	1.378	1.381	
	Unit 2 (C2–C3)	1.435	1.463	1.464	1.463	
	Unit 2 (C3–CH_2)	1.394	1.378	1.380	1.383	

Table S13. Comparison of selected experimental and calculated bond parameters (in Å) in **2**⁻, and **2·M(crypt-222)** (M = Li, Na, K, Cs).

Structure	Parameter	Experimental	DFT
**2**⁻	Ga–N1	1.986	2.069
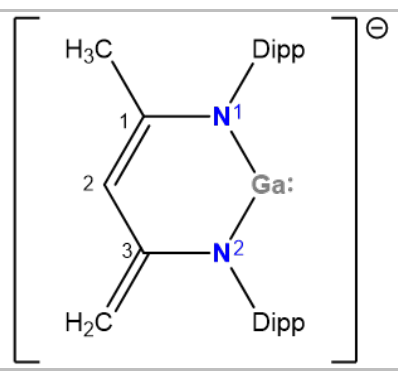	Ga–N2	1.985	2.024
	N1–C1	1.377	1.385
	N2–C3	1.380	1.399
	C1–C2	1.402	1.382
	C2–C3	1.420	1.462
	C3–CH_2	1.423	1.387
**2·Li(crypt-222)**	Ga···Li	5.769	5.747
	Ga–N1	1.986	2.025
	Ga–N2	1.985	1.996
	N1–C1	1.377	1.390
	N2–C3	1.380	1.406
	C1–C2	1.402	1.379
	C2–C3	1.420	1.462
	C3–CH_2	1.423	1.384
**2·Na(crypt-222)**	Ga···Na	NA	5.110
	Ga–N1	NA	2.032
	Ga–N2	NA	1.994
	N1–C1	NA	1.391
	N2–C3	NA	1.404
	C1–C2	NA	1.379
	C2–C3	NA	1.462
	C3–CH_2	NA	1.383
**2·K(crypt-222)**	Ga···K	5.154	5.219
	Ga–N1	1.992	2.039
	Ga–N2	1.986	2.001
	N1–C1	1.383	1.391
	N2–C3	1.386	1.404
	C1–C2	1.388	1.379
	C2–C3	1.398	1.461
	C3–CH_2	1.420	1.384
**2·Cs(crypt-222)**	Ga···Cs	5.129	5.194
	Ga–N1	1.995	2.040
	Ga–N2	2.000	2.001
	N1–C1	1.368	1.392
	N2–C3	1.378	1.404
	C1–C2	1.392	1.379
	C2–C3	1.406	1.461
	C3–CH_2	1.426	1.384

Table S14. Comparison of selected experimental and calculated bond parameters (Å) in **2·M(18-c-6)** (M = Na, K, Cs), **2·K(18-c-6)(THF)** and **2·Cs(18-c-6)**. [b]Periodic DFT-optimized structure.

Structure	Parameter	Experimental	DFT
**2·Na(18-c-6)**	Ga···Na	3.337	3.203
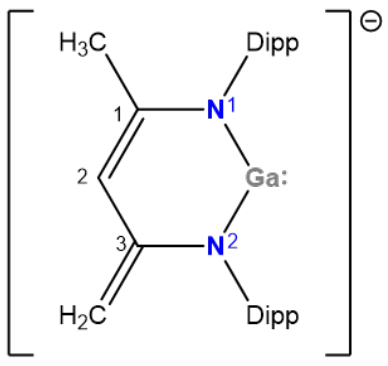	Ga–N1	1.979	2.011
	Ga–N2	1.965	1.976
	N1–C1	1.374	1.394
	N2–C3	1.382	1.408
	C1–C2	1.354	1.377
	C2–C3	1.447	1.460
	C3–CH_2	1.363	1.383
**2·K(18-c-6)**	Ga···K	3.490	3.517
	Ga–N1	1.976	2.014
	Ga–N2	1.953	1.978
	N1–C1	1.375	1.392
	N2–C3	1.386	1.406
	C1–C2	1.368	1.378
	C2–C3	1.451	1.462
	C3–CH_2	1.375	1.383
**2·Cs(18-c-6)**	Ga···Cs	3.708	3.776
	Ga–N1	1.979	1.997
	Ga–N2	1.971	1.992
	N1–C1	1.313	1.389
	N2–C3	1.412	1.404
	C1–C2	1.363	1.381
	C2–C3	1.432	1.462
	C3–CH_2	1.408	1.383
**2·K(18-c-6)(THF)**	Ga···K	3.705	3.626 (3.629)[b]
	K···O(THF)	2.839	3.283 (2.950)[b]
	Ga–N1	1.984	2.015 (2.055)[b]
	Ga–N2	1.984	1.976 (2.024)[b]
	N1–C1	1.375	1.392 (1.377)[b]
	N2–C3	1.375	1.407 (1.396)[b]
	C1–C2	1.399	1.378 (1.375)[b]
	C2–C3	1.399	1.462 (1.457)[b]
	C3–CH_2	1.428	1.383 (1.380)[b]
**2·Cs(18-c-6)$_2$**	Ga···Cs	8.013	6.808 (7.891)[b]
	Ga–N1	2.003	2.035 (2.083)[b]
	Ga–N2	1.990	1.997 (2.043)[b]
	N1–C1	1.364	1.389 (1.377)[b]
	N2–C3	1.369	1.401 (1.395)[b]
	C1–C2	1.402	1.380 (1.376)[b]
	C2–C3	1.425	1.464 (1.455)[b]
	C3–CH_2	1.424	1.383 (1.380)[b]

Table S15. Comparison of selected experimental and calculated bond parameters (Å, °) in **(2·M)$_2$** (M = K, Cs).

Structure	Parameter	Experimental	DFT
**(2·K)$_2$**	Ga1···Ga2	4.956	5.093
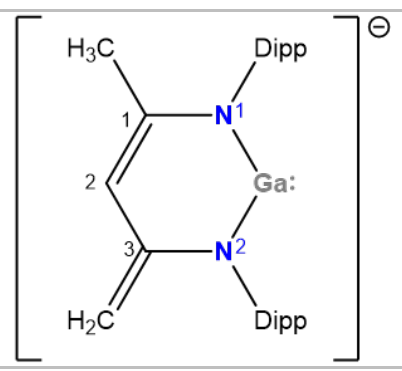	K1···K2	4.866	4.891
	Ga1···K1	3.468	3.535
	Ga1···K2	3.478	3.257
	Ga2···K1	3.468	3.535
	Ga2···K2	3.478	3.527
	Plane Angle	1.84	16.94
	K1···Dipp1 Centroid	2.921	3.001
	K1···Dipp2 Centroid	2.921	3.001
	K2···Dipp3 Centroid	2.894	3.047
	K2···Dipp4 Centroid	2.894	3.047
	Unit 1 (Ga–N1)	1.945	1.993
	Unit 1 (Ga–N2)	1.938	1.960
	Unit 1 (N1–C1)	1.380	1.393
	Unit 1 (N2–C3)	1.389	1.406
	Unit 1 (C1–C2)	1.385	1.378
	Unit 1 (C2–C3)	1.435	1.464
	Unit 1 (C3–CH$_2$)	1.394	1.380
	Unit 2 (Ga–N1)	1.945	1.993
	Unit 2 (Ga–N2)	1.938	1.960
	Unit 2 (N1–C1)	1.380	1.393
	Unit 2 (N2–C3)	1.389	1.406
	Unit 2 (C1–C2)	1.385	1.378
	Unit 2 (C2–C3)	1.435	1.464
	Unit 2 (C3–CH$_2$)	1.394	1.380
**(2·Cs)$_2$**	Ga1···Ga2	5.540	5.713
	Cs1···Cs2	5.253	5.125
	Ga1···Cs1	3.848	3.835
	Ga1···Cs2	3.786	3.840
	Ga2···Cs1	3.786	3.840
	Ga2···s2	3.848	3.835
	Plane Angle	0.00	0.01
	Cs1···Dipp1 Centroid	3.254	3.332
	Cs1···Dipp2 Centroid	3.284	3.380
	Cs2···Dipp3 Centroid	3.284	3.380
	Cs2···Dipp4 Centroid	3.254	3.332
	Unit 1 (Ga–N1)	1.960	1.998
	Unit 1 (Ga–N2)	1.939	1.967
	Unit 1 (N1–C1)	1.376	1.394
	Unit 1 (N2–C3)	1.389	1.407
	Unit 1 (C1–C2)	1.373	1.378
	Unit 1 (C2–C3)	1.439	1.462
	Unit 1 (C3–CH$_2$)	1.377	1.380
	Unit 2 (Ga–N1)	1.960	1.998
	Unit 2 (Ga–N2)	1.939	1.967
	Unit 2 (N1–C1)	1.376	1.394
	Unit 2 (N2–C3)	1.389	1.407
	Unit 2 (C1–C2)	1.373	1.378
	Unit 2 (C2–C3)	1.439	1.462
	Unit 2 (C3–CH$_2$)	1.377	1.380

Table S16. Comparison of selected experimental and calculated bond parameters (Å) in **3**, **4** and **5**.

Structure	Parameter	Experimental	DFT
**3**	Ga1–Au1	2.386	2.419
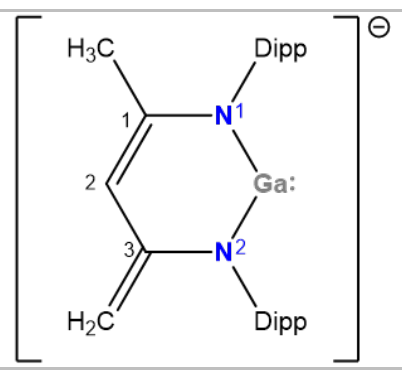	Au1–C_{NHC}	2.073	2.082
	Ga–N1	1.887	1.905
	Ga–N2	1.880	1.884
	N1–C1	1.393	1.398
	N2–C3	1.398	1.410
	C1–C2	1.376	1.375
	C2–C3	1.427	1.461
	C3–CH_2	1.395	1.376
**4**	Ga1–Au1	2.406	2.441
	Au1–C_{NHC}	2.077	2.107
	Ga1–NH_2	1.920	1.909
	Ga–N1	2.004	2.044
	Ga–N2	2.011	2.062
	N1–C1	1.335	1.341
	N2–C3	1.320	1.338
	C1–C2	1.399	1.417
	C2–C3	1.411	1.416
	C3–CH_3	1.509	1.515
**5**	Ga1–Au1	2.404	2.439
	Au1–C_{NHC}	2.078	2.103
	Ga1–OH	1.876	1.872
	Ga–N1	2.007	2.023
	Ga–N2	2.004	2.026
	N1–C1	1.327	1.340
	N2–C3	1.333	1.342
	C1–C2	1.410	1.414
	C2–C3	1.398	1.413
	C3–CH_3	1.517	1.515

Electronic structure analysis

S6.1.1 Canonical Kohn-Sham Molecule orbital analysis

Table S17. Relevant orbital energies and energetic gaps (eV) for all non-dimeric complexes. [b]Single-point energies were carried out on the solid-state optimized geometries.

Structure	HOMO→LUMO	HOMO–1→LUMO+*n*	LUMO+*n*	LUMO	HOMO	HOMO–1
**2**$^{-}$	3.72	4.31 (*n* = 0)	2.28	2.28	-1.44	-2.03
**2·Li(crypt-222)**	2.88	4.41 (*n* = 4)	0.01	-0.68	-3.56	-4.40
**2·Na(crypt-222)**	2.92	4.37 (*n* = 4)	0.02	-0.63	-3.55	-4.35
**2·K(crypt-222)**	2.83	4.34 (*n* = 4)	0.04	-0.67	-3.50	-4.30
**2·Cs(crypt-222)**	2.74	4.17 (*n* = 5)	-0.14	-0.75	-3.49	-4.31
**2·Na(18-c-6)**	3.34	4.64 (*n* = 1)	-0.32	-0.47	-3.81	-4.96
**2·K(18-c-6)**	3.28	4.63 (*n* = 3)	-0.12	-0.46	-3.74	-4.74
**2·Cs(18-c-6)**	3.13	4.32 (*n* = 3)	-0.28	-0.60	-3.73	-4.60
**2·K(18-c-6)(THF)**[b]	2.71	4.52 (*n* = 3)	-0.01	-0.71	-3.42	-4.60
**2·Cs(18-c-6)$_2$**[b]	2.01	4.45 (n = 9)	0.37	-0.98	-2.99	-4.08

Table S18. Relevant orbital energies and energetic gaps for the dimeric complexes.

Structure	HOMO→LUMO	LUMO+3	LUMO	HOMO	HOMO–2	HOMO–3
**(2·K)$_2$**	3.30	-0.80	-1.13	-4.43	-5.27	-5.79
**(2·Cs)$_2$**	3.24	-0.73	-1.13	-4.37	-5.32	-5.50

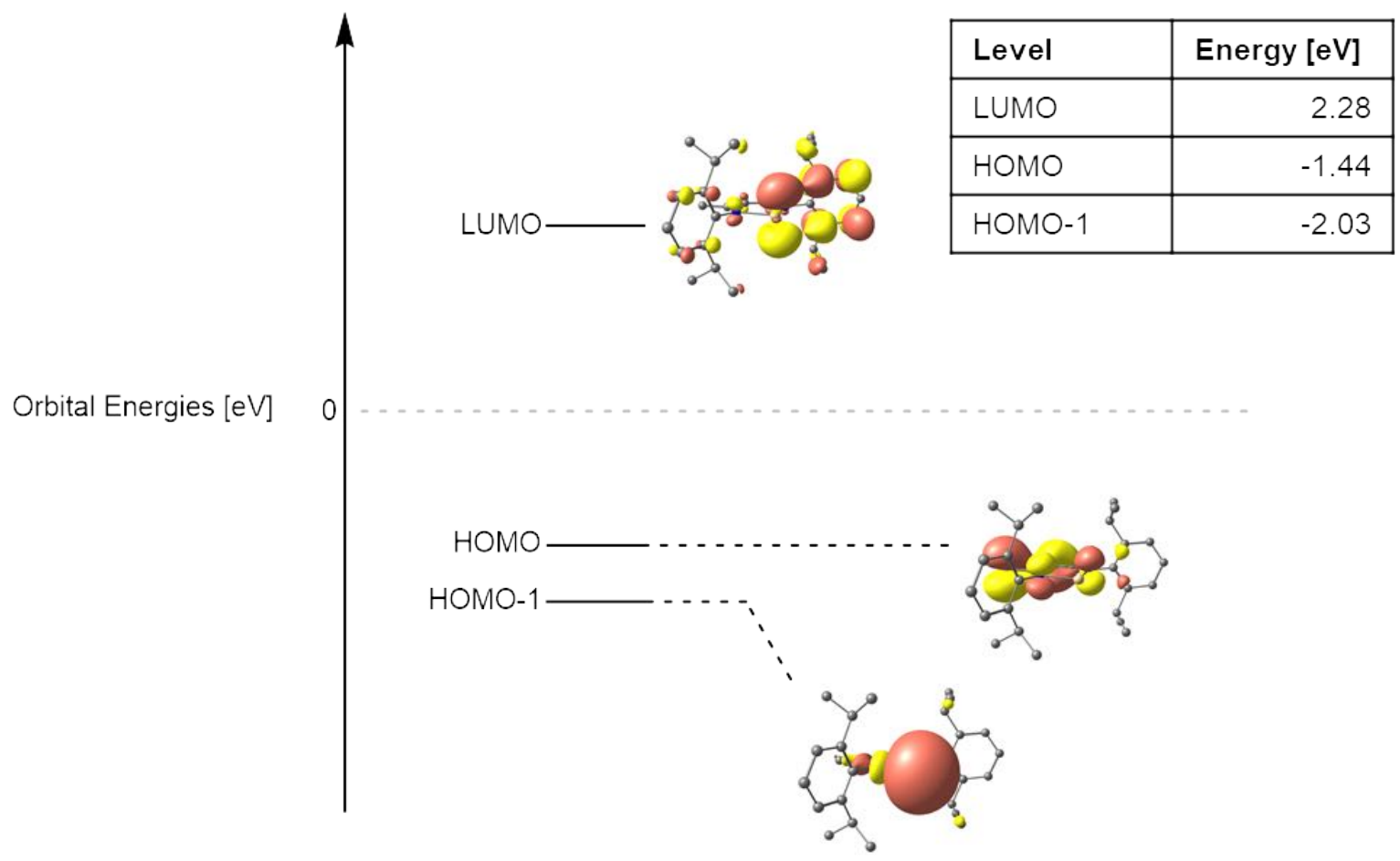

Figure S81. Canonical Kohn-Sham frontier MO diagram and orbitals for **2**$^{-}$ (isovalue = 0.3 a.u.).

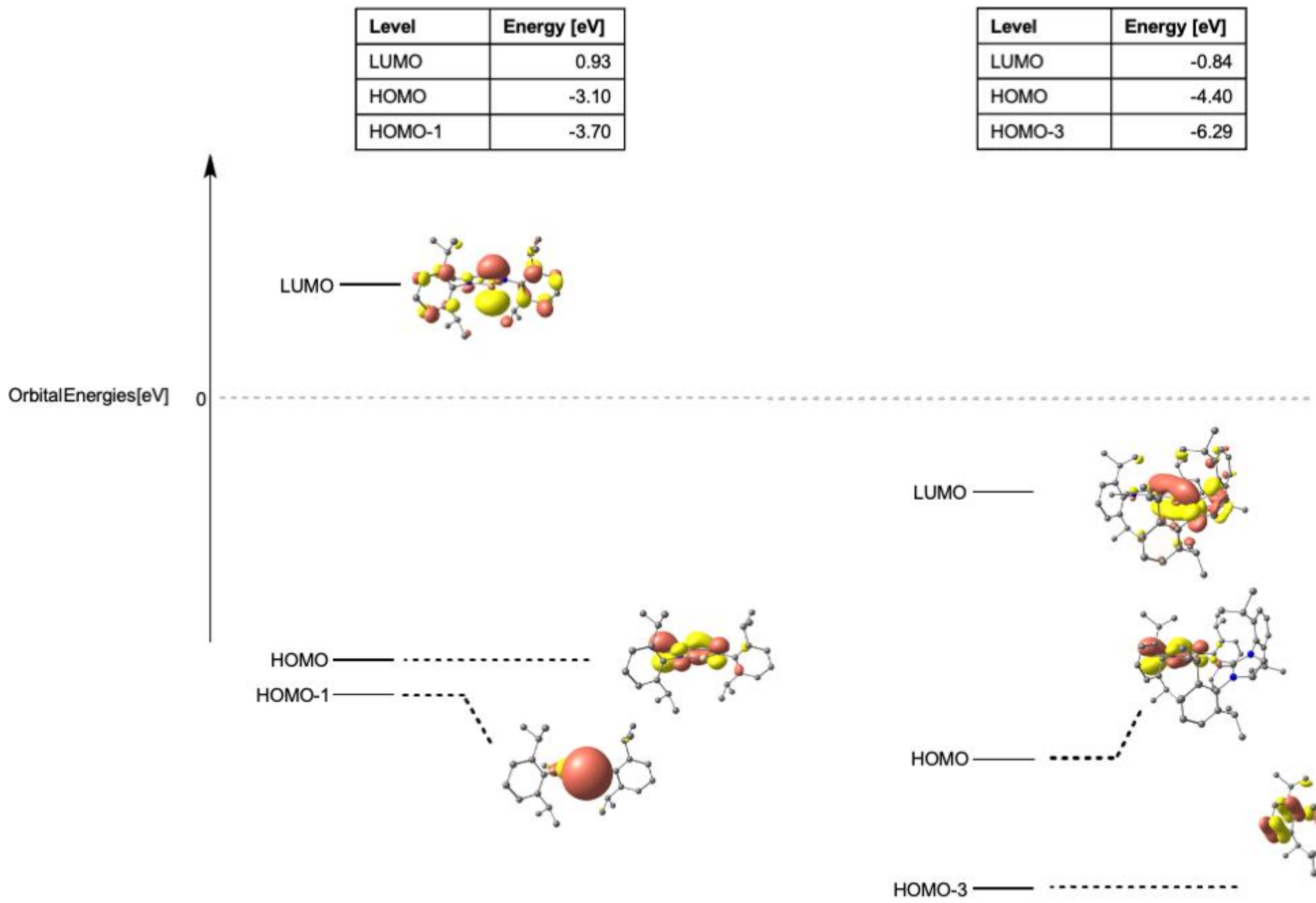

Figure S82. Canonical Kohn-Sham frontier MO diagram and orbitals for **2**$^{-}$ (left) and **3** (right) (isovalue = 0.03 au), obtained at the (SMD=benzene)-PBE0-D3(BJ)/def2-TZVP level of theory.

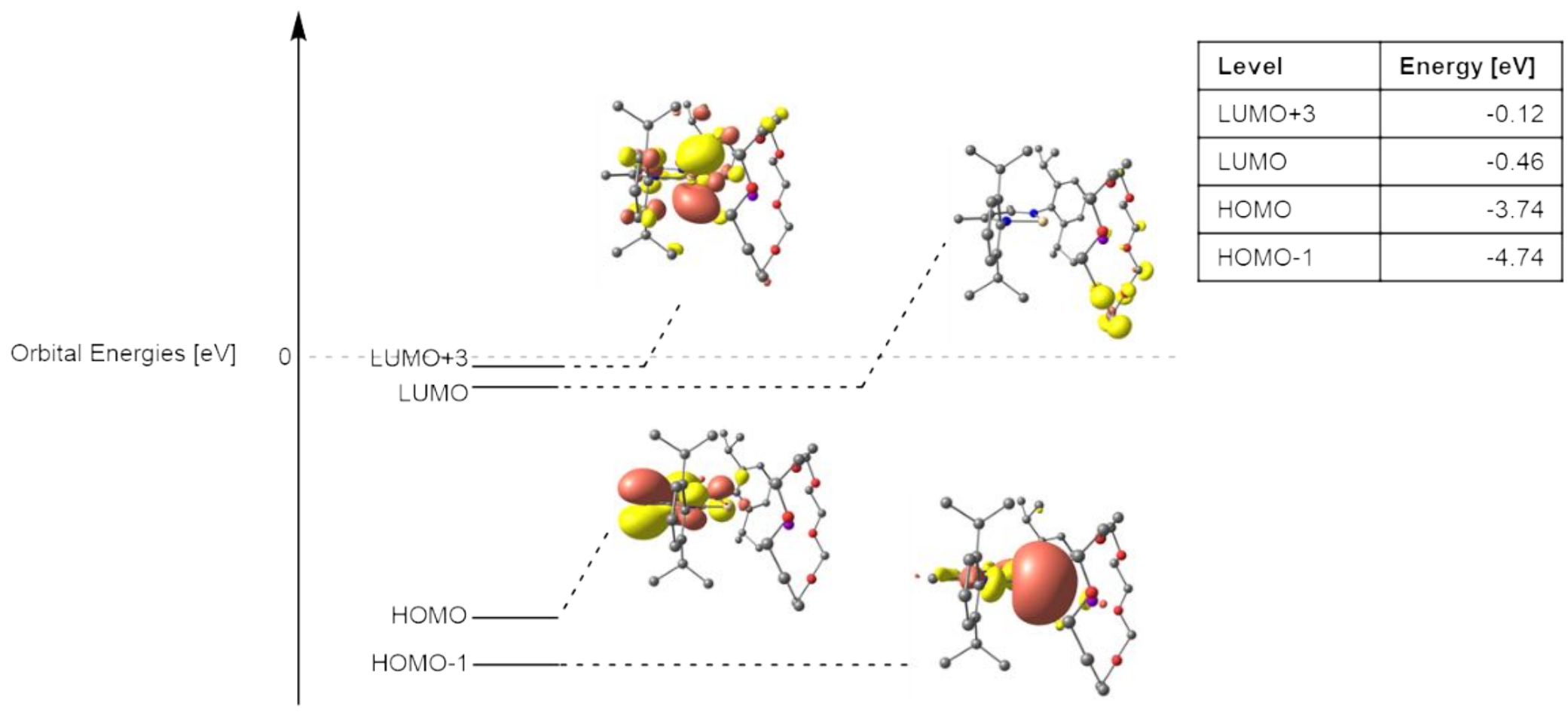

Figure S83. Canonical Kohn-Sham frontier MO diagram and orbitals for **2·K(18-c-6)** (isovalue = 0.3 au).

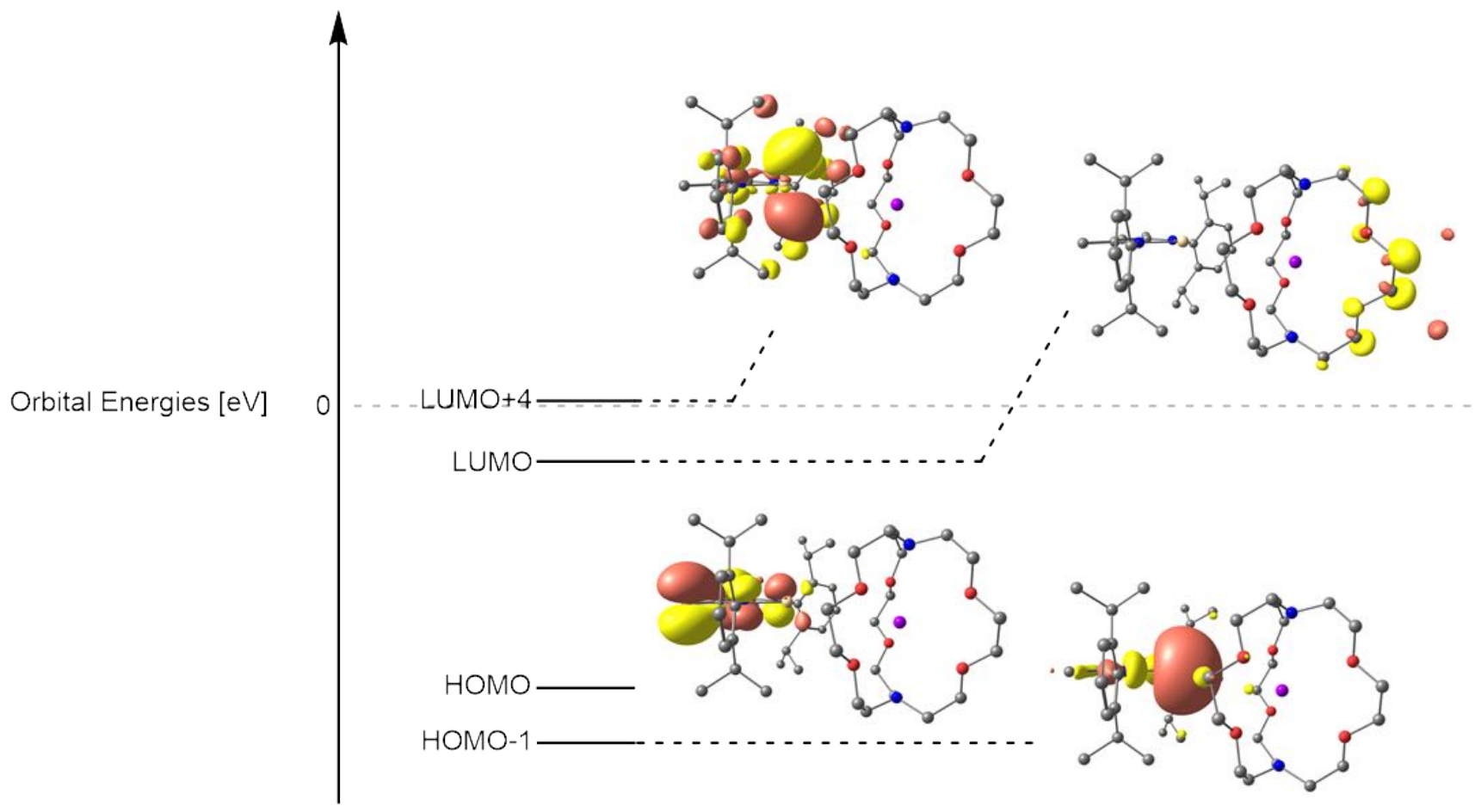

Level	Energy [eV]
LUMO+4	0.04
LUMO	-0.67
HOMO	-3.50
HOMO-1	-4.30

Figure S84. Canonical Kohn-Sham frontier MO diagram and orbitals for **2·K(crypt-222)** (isovalue = 0.3 au).

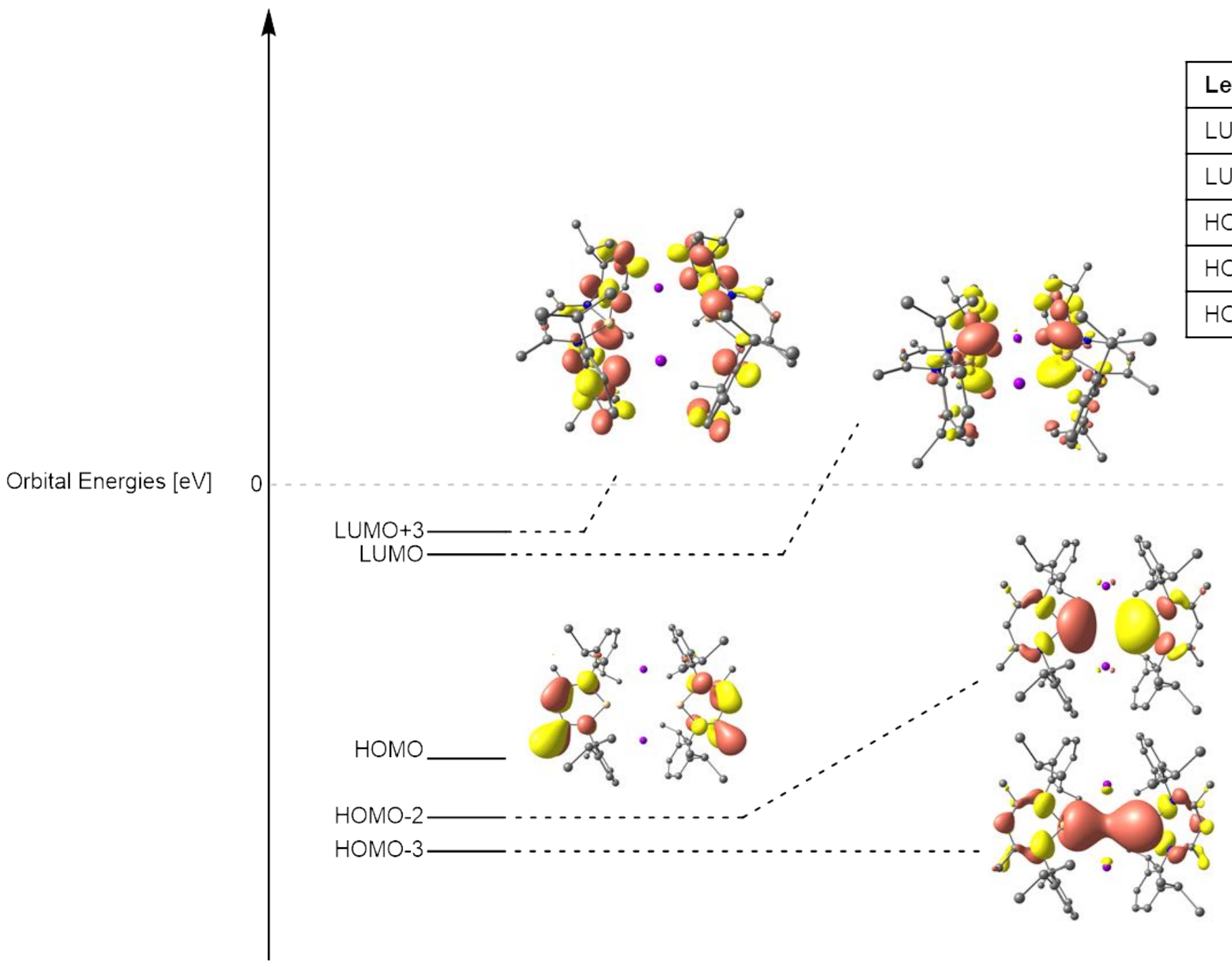

Level	Energy [eV]
LUMO+3	-0.80
LUMO	-1.13
HOMO	-4.43
HOMO-2	-5.27
HOMO-3	-5.79

Figure S85. Canonical Kohn-Sham frontier MO diagram and orbitals for **$(2{\cdot}K)_2$** (isovalue = 0.3 au).

S6.1.2 Natural bond orbital (NBO) analysis

First, we analyze the NBO results of the free gallyl anion **2**⁻ (**Table S19**). There is an NPA charge of +0.72 on the Ga center, and the negative charge is predominantly located on the ligand backbone, with charges of -0.90e and -0.94e on N1 and N2, respectively. Both Ga–N bonds are highly polarized towards the nitrogen centers (4% Ga and 96% N), reflected in the low Wiberg bond indices for the Ga–N bonds (0.21 and 0.25). Second order perturbation analysis revealed donations of similar magnitudes from the lone pair of each nitrogen center into the orthogonal p orbital of Ga (5.71 and 7.52 kcal mol^{-1} for N1 and N2, respectively). There is strong donation from the lone pair of N1 into the π* orbital of C1–C2 (57.57 kcal mol^{-1}) and from the lone pair of N2 into the π* orbital of C3–CH_2 (58.75 kcal mol^{-1}). There are also donations from the π orbital of C1–C2 into the π* orbital of C3–CH_2 (20.89 kcal mol^{-1}), and from the π orbital of C3–CH_2 into the π* orbital of C1–C2 (12.26 kcal mol^{-1}).

The extensive delocalization of the negative charge across the N2C5 backbone, as reflected in these interactions, can be better visualized through the resonance structures depicted in **Figure S86**. These structures highlight the charge delocalization and tautomeric equilibrium within the β-diketiminate framework, distinguishing this class of gallyls systems.

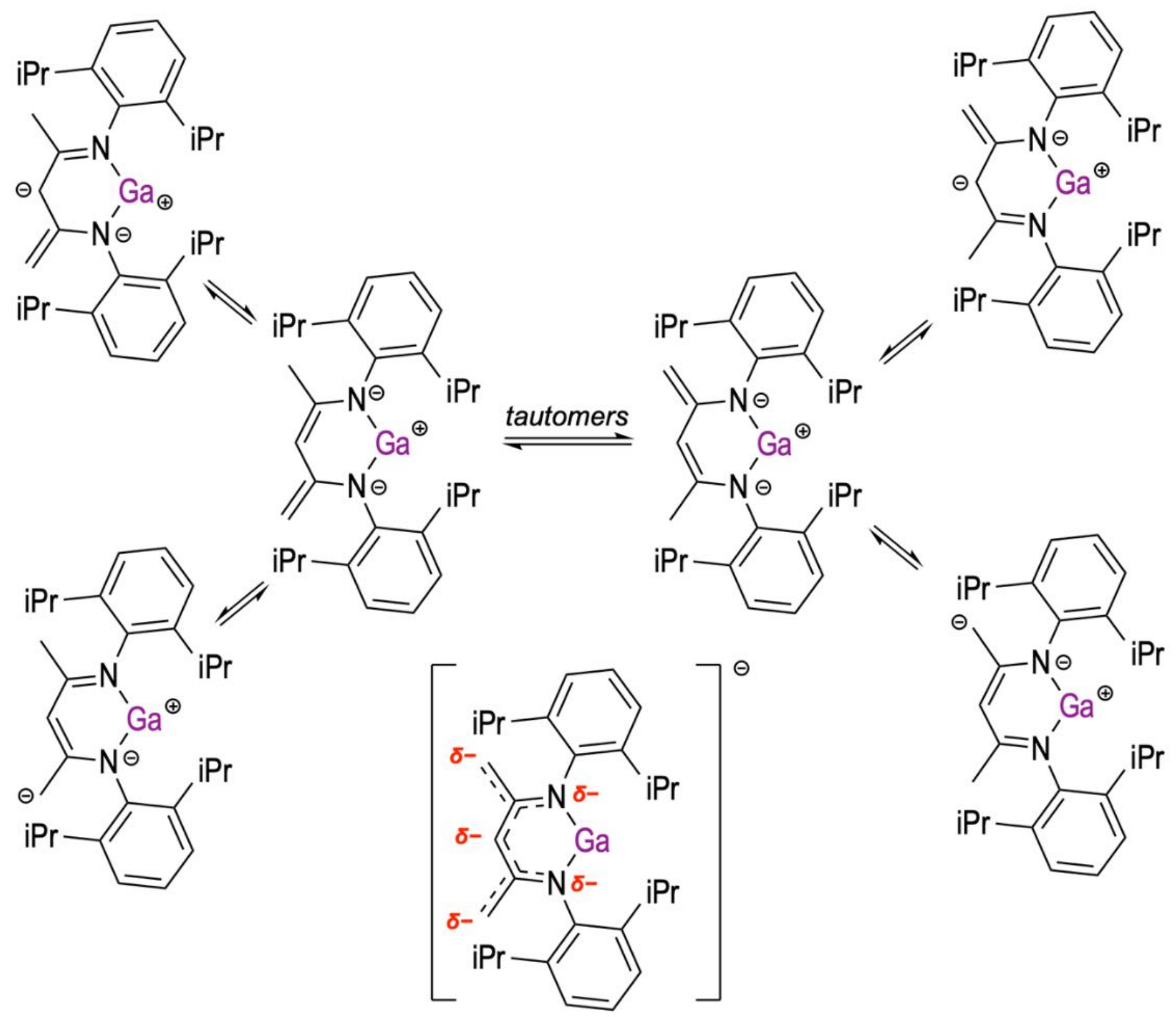

Figure S86. Representation of the resonance structures of the β-diketiminate backbone in the gallyl anion **2**⁻. The figure highlights the delocalization of the negative charge across the N2C5 backbone and the tautomeric equilibrium, as derived from NBO analysis.

For **2·Li(crypt-222)**, the NPA charge of +0.77e on the Ga center is only marginally more positive than in the free anion. The negative charge is predominantly located on the ligand backbone, with charges of -0.98e and -1.02 on N1 and N2, respectively. The small increase in negative charge on the nitrogen centers, and marginal decrease of negative charge on C2 and the methylene carbon is likely due to the electrostatic effect of the $[Li(crypt)]^+$ cation, which causes migration of the negative charge towards the nitrogen centers. The lone pair on Ga has a slightly lower occupancy of 1.89e, compared to 1.95e in the free anion, and there is slightly more polarization of the Ga–N bonds (2% Ga and 98% N), reflected in the lower Wiberg bond indices (0.15 and 0.18). Second order perturbation analysis revealed that there is strong donation from the lone pair of N1 into the π* orbital of C1–C2 (51.99 kcal mol^{-1}) but no donation from the lone pair of N2 into the π* orbital of C3–CH_2, unlike the free anion. However, there is a significant donation from the σ orbital of Ga–N2 into the π* orbital of C3–CH_2 (50.08 kcal mol^{-1}), reflected in the lower occupancy of the Ga–N2 NBO (1.76e), compared to the free anion (1.91e). The only noteworthy donation into the p orbital of Ga comes from the lone pair of N2 (10.51 kcal mol^{-1}). The donation from the lone pair of Ga into the s orbital of Li (0.11 kcal mol^{-1}) is negligible.

The notable difference between **2·Na(18-c-6)** and **2·Li(crypt-222)**, is the degree of interaction between the lone pair of the Ga center and the s orbital of Na. The occupancy of the lone pair on the Ga center is 1.86e, lower than in **2·Li(crypt-222)**. The occupancy of the Na 3s orbital is 0.19e, higher than the occupancy of the Li 2s orbital in **2·Li(crypt-222)** (0.14e). This is reflected in the second order perturbation analysis, which revealed a donation from the lone pair of Ga into the 3s orbital of Na (20.76 kcal mol^{-1}). This stronger interaction between the Ga and Na centers is in line with the contacted nature of this structure, in comparison to the separated nature of **2·Li(crypt-222)**.

2·K(18-c-6), is a useful comparison to **2·Na(18-c-6)**, as we can analyze the effect of changing the alkali metal on the electronic structure. The degree of donation into the 4s orbital of potassium from the Ga lone pair (15.98 kcal mol^{-1}) is less than that of **2·Na(18-c-6)** by ~5 kcal mol^{-1}. This is reflected in the slightly lower population of the potassium 4s orbital (0.17e), and due to the more electronegative Na center.

The coordination of a THF molecule in gas phase optimized **2·K(18-c-6)(THF)** leads to a difference in the interaction between the two metal centers, in comparison to the donor-free structure. There is a stronger donation from the lone pair of Ga into the 4s orbital of potassium (28.11 kcal mol^{-1}), accompanied by a small donation from the oxygen lone pair of THF into the 4s orbital of potassium (3.99 kcal mol^{-1}). This predictably leads to a less positive charge on potassium (0.78e), a higher occupancy of the 4s orbital (0.20e), as well as a slightly lower occupancy of the Ga lone pair (1.84e) in comparison to the donor-free structure (1.86e). The solid state optimized **2·K(18-c-6)(THF)** gives similar results, despite the difference in the K···O(THF) distance (3.283 / 2.950 Å for gas / solid-state optimized structures). The donation from the Ga lone pair to the K 4s orbital has a magnitude of 27.04 kcal mol^{-1} (~1 kcal mol^{-1} decrease relative to gas-phase optimized geometry), and the donation from the THF oxygen to the K 4s orbital has a magnitude of 4.03 kcal mol^{-1} (unchanged relative to gas-phase optimized geometry). Therefore, we conclude that the structural changes in the gas and solid state-optimized geometries only induce marginal differences in their NBO results.

For **2·K(crypt-222)**, the only notable difference to related structure of **2·Li(crypt-222)**, is the degree of donation from the lone pair of Ga into the s orbital of the alkali metal. In the case of potassium, there is a donation of 6.27 kcal mol^{-1}, in comparison to 0.11 kcal mol^{-1} for lithium. For **2·Cs(crypt-222)**, there is a donation from the lone pair of Ga into the 6s orbital of Cs (4.38 kcal mol^{-1}). We can attribute this to the presence of a Coulomb contact, one that is weaker than **2·K(crypt-222)** (6.27 kcal mol^{-1}) and stronger than **2·Li(crypt-222)** (0.11 kcal mol^{-1}), placing it in an intermediary position between the two.

When comparing **2·Cs(18-c-6)** with **2·K(18-c-6)**, there are two notable differences. The first is the degree of donation from the lone pair of gallium into the s orbital of the alkali metal. In the case of **2·Cs(18-c-6)**, there is a donation of 14.45 kcal mol^{-1}, compared to a donation of 15.98 kcal mol^{-1} in the case of **2·K(18-c-6)**. The second notable difference is the population of the Ga–N2 σ bond, which has a population of 1.91e in **2·K(18-c-6)** and 1.76e in **2·Cs(18-c-6)**. The lower population in the case of cesium can be attributed to the strong donation of 50.63 kcal mol^{-1} from the Ga–N2 σ bond into the C3–CH2 π* bond, also observed in the case of **2·Li(crypt-222)**. This strong donation seemingly replaces the donation from the lone pair of N2 into the C3–CH_2 π* bond, which is present in **2·K(18-c-6)**, but not in **2·Cs(18-c-6)**.

When comparing the two dimeric structures, **(2·K)$_2$** and **(2·Cs)$_2$**, the major difference is the degree of donation from the gallium centers into the alkali metal. In the case of **(2·K)$_2$** there are donations from the lone pairs of the Ga centers, that are more than twice that of the donations in **(2·Cs)$_2$** (18.54/18.51 vs 8.36/8.14 kcal mol^{-1} for K and Cs, respectively). This is reflected in the charges on the alkali metal centers (0.78e and 0.85e for K and Cs, respectively), the occupancies of the Ga lone pairs (1.78e and 1.84e for **(2·K)$_2$** and **(2·Cs)$_2$**, respectively) and the alkali metal s orbitals (0.20e and 0.13e for **(2·K)$_2$** and **(2·Cs)$_2$**, respectively).

Table S19. NBO analysis for **2$^-$** and **2·M(crypt-222)** (M = Li, Na, K, Cs).

Structure	NBO	NPA Charge	Occup.	Pol.	Hybrid.	WBI
**2$^-$**	LP (Ga)	0.72 (Ga)	1.95	-	$sp^{0.08}$ (Ga)	-
	σ (Ga–N1)	0.72 (Ga)<br>-0.90 (N1)	1.89	4% (Ga)<br>96% (N1)	$sp^{18.82}$ (Ga)<br>$sp^{3.00}$ (N1)	0.21
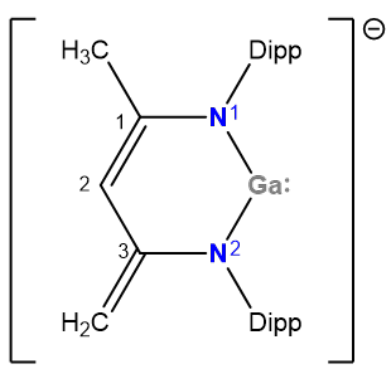	σ (Ga–N2)	0.72 (Ga)<br>-0.94 (N2)	1.91	4% (Ga)<br>96% (N2)	$sp^{16.32}$ (Ga)<br>$sp^{2.74}$ (N2)	0.25
	σ (C1–C2)	0.20 (C1)<br>-0.37 (C2)	1.98	51% (C1)<br>49% (C2)	$sp^{1.62}$ (C1)<br>$sp^{1.64}$ (C2)	1.61
	π (C1–C2)	0.20 (C1)<br>-0.37 (C2)	1.87	43% (C1)<br>57% (C2)	$sp^{1.00}$ (C1)<br>$sp^{1.00}$ (C2)	1.61
	σ (C3–CH_2)	0.17 (C3)<br>-0.64 (CH_2)	1.98	52% (C3)<br>48% (CH_2)	$sp^{1.60}$ (C3)<br>$sp^{1.60}$ (CH_2)	1.62
	π (C3–CH_2)	0.17 (C3)<br>-0.64 (CH_2)	1.94	39% (C3)<br>61% (CH_2)	$sp^{1.00}$ (C3)<br>$sp^{1.00}$ (CH_2)	1.62
**2·Li(crypt-222)**	LP (Ga)	0.77 (Ga)	1.89	-	$sp^{0.05}$ (Ga)	0.00
	LV (Li)	0.82 (Li)	0.14	-	s	
	σ (Ga–N1)	0.77 (Ga)<br>-0.98 (N1)	1.89	2% (Ga)<br>98% (N1)	$sp^{8.39}$ (Ga)<br>$sp^{3.83}$ (N1)	0.15
	σ (Ga–N2)	0.77 (Ga)<br>-1.02 (N2)	1.76	2% (Ga)<br>98% (N2)	$sp^{99.99}$ (Ga)<br>$sp^{1.00}$ (N2)	0.18
	σ (C1–C2)	0.20 (C1)<br>-0.35 (C2)	1.98	51% (C1)<br>49% (C2)	$sp^{1.62}$ (C1)<br>$sp^{1.63}$ (C2)	1.63
	π (C1–C2)	0.20 (C1)<br>-0.35 (C2)	1.87	44% (C1)<br>56% (C2)	$sp^{99.99}$ (C1)<br>$sp^{1.00}$ (C2)	1.63
	σ (C3–CH_2)	0.16 (C3)<br>-0.62 (CH_2)	1.98	52% (C3)<br>48% (CH_2)	$sp^{1.63}$ (C3)<br>$sp^{1.60}$ (CH_2)	1.65
	π (C3–CH_2)	0.16 (C3)<br>-0.62 (CH_2)	1.93	40% (C3)<br>60% (CH_2)	$sp^{99.99}$ (C3)<br>$sp^{99.99}$ (CH_2)	1.65
**2·Na(crypt-222)**	LP (Ga)	0.77 (Ga)	1.88	-	$sp^{0.06}$ (Ga)	0.04
	LV (Na)	0.82 (Na)	0.16	-	s	
	σ (Ga–N1)	0.77 (Ga)<br>-0.97 (N1)	1.89	2% (Ga)<br>98% (N1)	$sp^{15.77}$ (Ga)<br>$sp^{3.60}$ (N1)	0.16
	σ (Ga–N2)	0.77 (Ga)<br>-1.01 (N2)	1.90	2% (Ga)<br>98% (N2)	$sp^{13.91}$ (Ga)<br>$sp^{3.31}$ (N2)	0.19
	σ (C1–C2)	0.20 (C1)<br>-0.35 (C2)	1.98	51% (C1)<br>49% (C2)	$sp^{1.62}$ (C1)<br>$sp^{1.63}$ (C2)	1.63
	π (C1–C2)	0.20 (C1)<br>-0.35 (C2)	1.87	44% (C1)<br>56% (C2)	$sp^{99.99}$ (C1)<br>$sp^{1.00}$ (C2)	1.63
	σ (C3–CH_2)	0.16 (C3)<br>-0.62 (CH_2)	1.98	52% (C3)<br>48% (CH_2)	$sp^{1.56}$ (C3)<br>$sp^{1.60}$ (CH_2)	1.65

	π (C3–CH_2)	0.16 (C3)<br>-0.62 (CH_2)	1.93	41% (C3)<br>59% (CH_2)	$sp^{1.00}$ (C3)<br>$sp^{99.99}$ (CH_2)	1.65
**2·K(crypt-222)**	LP (Ga)	0.75 (Ga)	1.91	-	$sp^{0.06}$ (Ga)	0.04
	LV (K)	0.83 (K)	0.15	-	s	
	σ (Ga–N1)	0.75 (Ga)<br>-0.96 (N1)	1.89	2% (Ga)<br>98% (N1)	$sp^{15.28}$ (Ga)<br>$sp^{3.56}$ (N1)	0.16
	σ (Ga–N2)	0.75 (Ga)<br>-1.00 (N2)	1.90	2% (Ga)<br>98% (N2)	$sp^{13.87}$ (Ga)<br>$sp^{3.27}$ (N2)	0.19
	σ (C1–C2)	0.20 (C1)<br>-0.35 (C2)	1.98	49% (C1)<br>51% (C2)	$sp^{1.63}$ (C1)<br>$sp^{1.62}$ (C2)	1.63
	π (C1–C2)	0.20 (C1)<br>-0.35 (C2)	1.87	56% (C1)<br>44% (C2)	$sp^{1.00}$ (C1)<br>$sp^{99.99}$ (C2)	1.63
	σ (C3–CH_2)	0.16 (C3)<br>-0.62 (CH_2)	1.98	48% (C3)<br>52% (CH_2)	$sp^{1.60}$ (C3)<br>$sp^{1.56}$ (CH_2)	1.64
	π (C3–CH_2)	0.16 (C3)<br>-0.62 (CH_2)	1.93	60% (C3)<br>40% (CH_2)	$sp^{1.00}$ (C3)<br>$sp^{1.00}$ (CH_2)	1.64
**2·Cs(crypt-222)**	LP (Ga)	0.75 (Ga)	1.91	-	$sp^{0.06}$ (Ga)	0.04
	LV (Cs)	0.82 (Cs)	0.15	-	s	
	σ (Ga–N1)	0.75 (Ga)<br>-0.97 (N1)	1.89	2% (Ga)<br>98% (N1)	$sp^{14.84}$ (Ga)<br>$sp^{3.60}$ (N1)	0.16
	σ (Ga–N2)	0.75 (Ga)<br>-1.01 (N2)	1.90	2% (Ga)<br>98% (N2)	$sp^{13.76}$ (Ga)<br>$sp^{3.31}$ (N2)	0.19
	σ (C1–C2)	0.20 (C1)<br>-0.35 (C2)	1.98	51% (C1)<br>49% (C2)	$sp^{1.62}$ (C1)<br>$sp^{1.63}$ (C2)	1.63
	π (C1–C2)	0.20 (C1)<br>-0.35 (C2)	1.87	44% (C1)<br>56% (C2)	$sp^{99.99}$ (C1)<br>$sp^{1.00}$ (C2)	1.63
	σ (C3–CH_2)	0.16 (C3)<br>-0.62 (CH_2)	1.98	52% (C3)<br>48% (CH_2)	$sp^{1.56}$ (C3)<br>$sp^{1.60}$ (CH_2)	1.64
	π (C3–CH_2)	0.16 (C3)<br>-0.62 (CH_2)	1.93	40% (C3)<br>60% (CH_2)	$sp^{99.99}$(C3)<br>$sp^{1.00}$ (CH_2)	1.64

Table S20. NBO analysis for **2·M(18-c-6)** (M = Na, K, Cs) and **2·K(18-c-6)(THF)**. [b]Solid state optimized geometry was used for NBO analysis.

**Structure**	**NBO**	**NPA Charge**	**Occup.**	**Pol.**	**Hybrid.**	**WBI**
**2·Na(18-c-6)**	LP (Ga)	0.75 (Ga)	1.86	-	$sp^{0.09}$ (Ga)	0.14
	LV (Na)	0.80 (Na)	0.19	-	s	
	σ (Ga–N1)	0.75 (Ga)<br>-0.97 (N1)	1.90	3% (Ga)<br>97% (N1)	$sp^{13.62}$ (Ga)<br>$sp^{3.23}$ (N1)	0.19
	σ (Ga–N2)	0.75 (Ga)<br>-1.01 (N2)	1.91	3% (Ga)<br>97% (N2)	$sp^{99.99}$ (Ga)<br>$sp^{1.00}$ (N2)	0.22
	σ (C1–C2)	0.20 (C1)<br>-0.35 (C2)	1.98	51% (C1)<br>49% (C2)	$sp^{1.61}$ (C1)<br>$sp^{1.63}$ (C2)	1.64
	π (C1–C2)	0.20 (C1)<br>-0.35 (C2)	1.87	44% (C1)<br>56% (C2)	$sp^{99.99}$ (C1)<br>$sp^{1.00}$ (C2)	1.64
	σ (C3–CH_2)	0.15 (C3)<br>-0.61 (CH_2)	1.98	52% (C3)<br>48% (CH_2)	$sp^{1.57}$ (C3)<br>$sp^{1.60}$ (CH_2)	1.65
	π (C3–CH_2)	0.15 (C3)<br>-0.61 (CH_2)	1.93	41% (C3)<br>59% (CH_2)	$sp^{1.00}$ (C3)<br>$sp^{1.00}$ (CH_2)	1.65
**2·K(18-c-6)**	LP (Ga)	0.77 (Ga)	1.86	-	$sp^{0.08}$ (Ga)	0.13
	LV (K)	0.81 (K)	0.17	-	s	
	σ (Ga–N1)	0.77 (Ga)<br>-0.97 (N1)	1.89	3% (Ga)<br>97% (N1)	$sp^{14.93}$ (Ga)<br>$sp^{3.35}$ (N1)	0.18
	σ (Ga–N2)	0.77 (Ga)<br>-1.01 (N2)	1.91	3% (Ga)<br>97% (N2)	$sp^{13.52}$ (Ga)<br>$sp^{3.16}$ (N2)	0.21
	σ (C1–C2)	0.20 (C1)<br>-0.35 (C2)	1.98	51% (C1)<br>49% (C2)	$sp^{1.61}$ (C1)<br>$sp^{1.63}$ (C2)	1.63
	π (C1–C2)	0.20 (C1)<br>-0.35 (C2)	1.87	44% (C1)<br>56% (C2)	$sp^{99.99}$ (C1)<br>$sp^{1.00}$ (C2)	1.63
	σ (C3–CH_2)	0.16 (C3)<br>-0.61 (CH_2)	1.98	52% (C3)<br>48% (CH_2)	$sp^{1.57}$ (C3)<br>$sp^{1.60}$ (CH_2)	1.65
	π (C3–CH_2)	0.16 (C3)<br>-0.61 (CH_2)	1.93	41% (C3)<br>59% (CH_2)	$sp^{99.99}$ (C3)<br>$Sp^{99.99}$ (CH_2)	1.65

**2·Cs(18-c-6)**	LP (Ga)	0.78 (Ga)	1.87	-	$sp^{0.06}$ (Ga)	0.08
	LV (Cs)	0.83 (Cs)	0.15	-	s	
	σ (Ga–N1)	0.78 (Ga)<br>-0.99 (N1)	1.90	3% (Ga)<br>97% (N1)	$sp^{8.71}$ (Ga)<br>$sp^{3.56}$ (N1)	0.17
	σ (Ga–N2)	0.78 (Ga)<br>-1.01 (N2)	1.76	2% (Ga)<br>98% (N2)	$sp^{99.99}$ (Ga)<br>$Sp^{1.00}$ (N2)	0.18
	σ (C1–C2)	0.21 (C1)<br>-0.36 (C2)	1.98	51% (C1)<br>49% (C2)	$sp^{1.62}$ (C1)<br>$sp^{1.64}$ (C2)	1.62
	π (C1–C2)	0.21 (C1)<br>-0.36 (C2)	1.87	43% (C1)<br>57% (C2)	$sp^{1.00}$ (C1)<br>$sp^{1.00}$ (C2)	1.62
	σ (C3–CH_2)	0.16 (C3)<br>-0.62 (CH_2)	1.98	52% (C3)<br>48% (CH_2)	$sp^{1.62}$ (C3)<br>$sp^{1.60}$ (CH_2)	1.64
	π (C3–CH_2)	0.16 (C3)<br>-0.62 (CH_2)	1.94	40% (C3)<br>60% (CH_2)	$sp^{1.00}$ (C3)<br>$sp^{1.00}$ (CH_2)	1.64
**2·K(18-c-6)(THF)**	LP (Ga)	0.79 (Ga)	1.84	-	$sp^{0.08}$ (Ga)	0.14
	LV (K)	0.78 (K)	0.20	-	s	0.02
	LP (THF O)	-0.56 (O)	1.92	-	$sp^{99.99}$	
	σ (Ga–N1)	0.79 (Ga)<br>-0.98 (N1)	1.89	2% (Ga)<br>98% (N1)	$sp^{15.01}$ (Ga)<br>$sp^{3.41}$ (N1)	0.17
	σ (Ga–N2)	0.79 (Ga)<br>-1.02 (N2)	1.91	3% (Ga)<br>97% (N2)	$sp^{13.06}$ (Ga)<br>$sp^{3.24}$ (N2)	0.20
	σ (C1–C2)	0.20 (C1)<br>-0.35 (C2)	1.98	51% (C1)<br>49% (C2)	$sp^{1.61}$ (C1)<br>$sp^{1.63}$ (C2)	1.63
	π (C1–C2)	0.20 (C1)<br>-0.35 (C2)	1.87	44% (C1)<br>56% (C2)	$sp^{1.00}$ (C1)<br>$sp^{1.00}$ (C2)	1.63
	σ (C3–CH_2)	0.16 (C3)<br>-0.61 (CH_2)	1.98	52% (C3)<br>48% (CH_2)	$sp^{1.56}$ (C3)<br>$sp^{1.60}$ (CH_2)	1.65
	π (C3–CH_2)	0.16 (C3)<br>-0.61 (CH_2)	1.93	41% (C3)<br>59% (CH_2)	$sp^{1.00}$ (C3)<br>$sp^{1.00}$ (CH_2)	1.65
**2·K(18-c-6)(THF)**[b]	LP (Ga)	0.80 (Ga)	1.86	-	$sp^{0.06}$ (Ga)	0.15
	LV (K)	0.77 (K)	0.20	-	s	0.02
	LP (THF O)	-0.57 (O)	1.93	-	$sp^{99.99}$	
	σ (Ga–N1)	0.80 (Ga)<br>-0.96 (N1)	1.88	2% (Ga)<br>98% (N1)	$sp^{16.07}$ (Ga)<br>$sp^{3.65}$ (N1)	0.16
	σ (Ga–N2)	0.80 (Ga)<br>-0.99 (N2)	1.90	3% (Ga)<br>97% (N2)	$sp^{14.46}$ (Ga)<br>$sp^{3.28}$ (N2)	0.20
	σ (C1–C2)	0.20 (C1)<br>-0.36 (C2)	1.98	51% (C1)<br>49% (C2)	$sp^{1.62}$ (C1)<br>$sp^{1.65}$ (C2)	1.62
	π (C1–C2)	0.20 (C1)<br>-0.36 (C2)	1.87	43% (C1)<br>57% (C2)	$sp^{1.00}$ (C1)<br>$sp^{1.00}$ (C2)	1.62
	σ (C3–CH_2)	0.16 (C3)<br>-0.62 (CH_2)	1.98	52% (C3)<br>48% (CH_2)	$sp^{1.58}$ (C3)<br>$sp^{1.62}$ (CH_2)	1.64
	π (C3–CH_2)	0.16 (C3)<br>-0.62 (CH_2)	1.94	40% (C3)<br>60% (CH_2)	$sp^{99.99}$ (C3)<br>$sp^{99.99}$ (CH_2)	1.64

Table S21. NBO analysis for (**2·**M)$_2$ (M = K, Cs).

Structure	NBO	NPA Charge	Occup.	Pol.	Hybrid.	WBI
**(2·K)$_2$**	LP (Ga1)	0.82 (Ga)	1.78	-	$sp^{0.07}$ (Ga)	0.13<br>0.10<br>0.11
	LV (K1)	0.78 (K)	0.21	-	s	
	LP (Ga2)	0.82 (Ga)	1.78	-	$sp^{0.07}$ (Ga)	
	LV (K2)	0.78 (K)	0.20	-	s	
	Unit 1 σ (Ga–N1)	0.82 (Ga)<br>-0.98 (N1)	1.89	3% (Ga)<br>97% (N1)	$sp^{15.07}$ (Ga)<br>$sp^{3.36}$ (N1)	0.18
	Unit 1 σ (Ga–N2)	0.82 (Ga)<br>-1.03 (N2)	1.90	3% (Ga)<br>97% (N2)	$sp^{13.15}$ (Ga)<br>$sp^{3.15}$ (N2)	0.21
	Unit 1 σ (C1–C2)	0.20 (C1)<br>-0.35 (C2)	1.98	51% (C1)<br>49% (C2)	$sp^{1.61}$ (C1)<br>$sp^{1.63}$ (C2)	1.64
	Unit 1 π (C1–C2)	0.20 (C1)<br>-0.35 (C2)	1.87	44% (C1)<br>56% (C2)	$sp^{1.00}$ (C1)<br>$sp^{1.00}$ (C2)	1.64
	Unit 1 σ (C3–CH_2)	0.15 (C3)<br>-0.59 (CH_2)	1.98	52% (C3)<br>48% (CH_2)	$sp^{1.56}$ (C3)<br>$sp^{1.60}$ (CH_2)	1.66
	Unit 1 π (C3–CH_2)	0.15 (C3)<br>-0.59 (CH_2)	1.93	42% (C3)<br>58% (CH_2)	$sp^{1.00}$ (C3)<br>$sp^{1.00}$ (CH_2)	1.66
	Unit 2 σ (Ga–N1)	0.82 (Ga)<br>-0.98 (N1)	1.89	3% (Ga)<br>97% (N1)	$sp^{15.07}$ (Ga)<br>$sp^{3.36}$ (N1)	0.18
	Unit 2 σ (Ga–N2)	0.82 (Ga)<br>-1.03 (N2)	1.90	3% (Ga)<br>97% (N2)	$sp^{13.15}$ (Ga)<br>$sp^{3.15}$ (N2)	0.21
	Unit 2 σ (C1–C2)	0.20 (C1)<br>-0.35 (C2)	1.98	51% (C1)<br>49% (C2)	$sp^{1.61}$ (C1)<br>$sp^{1.63}$ (C2)	1.64
	Unit 2 π (C1–C2)	0.20 (C1)<br>-0.35 (C2)	1.87	44% (C1)<br>56% (C2)	$sp^{1.00}$ (C1)<br>$sp^{1.00}$ (C2)	1.64
	Unit 2 σ (C3–CH_2)	0.15 (C3)<br>-0.59 (CH_2)	1.98	52% (C3)<br>48% (CH_2)	$sp^{1.56}$ (C3)<br>$sp^{1.60}$ (CH_2)	1.66
	Unit 2 π (C3–CH_2)	0.15 (C3)<br>-0.59 (CH_2)	1.93	42% (C3)<br>58% (CH_2)	$sp^{1.00}$ (C3)<br>$sp^{1.00}$ (CH_2)	1.66
**(2·Cs)$_2$**	LP (Ga1)	0.79 (Ga)	1.84	-	$sp^{0.08}$ (Ga)	0.04<br>0.07<br>0.07
	LV (Cs1)	0.85 (Cs)	0.13	-	s	
	LP (Ga2)	0.79 (Ga)	1.84	-	$sp^{0.08}$ (Ga)	
	LV (Cs2)	0.85 (Cs)	0.13	-	s	
	Unit 1 σ (Ga–N1)	0.79 (Ga)<br>-0.99 (N1)	1.89	3% (Ga)<br>97% (N1)	$sp^{13.88}$ (Ga)<br>$sp^{3.33}$ (N1)	0.19
	Unit 1 σ (Ga–N2)	0.79 (Ga)<br>-1.03 (N2)	1.91	3% (Ga)<br>97% (N2)	$sp^{12.49}$ (Ga)<br>$sp^{3.17}$ (N2)	0.22
	Unit 1 σ (C1–C2)	0.20 (C1)<br>-0.35 (C2)	1.98	51% (C1)<br>49% (C2)	$sp^{1.61}$ (C1)<br>$sp^{1.63}$ (C2)	1.64
	Unit 1 π (C1–C2)	0.20 (C1)<br>-0.35 (C2)	1.87	44% (C1)<br>56% (C2)	$sp^{1.00}$ (C1)<br>$sp^{1.00}$ (C2)	1.64
	Unit 1 σ (C3–CH_2)	0.15 (C3)<br>-0.60 (CH_2)	1.98	52% (C3)<br>48% (CH_2)	$sp^{1.56}$ (C3)<br>$sp^{1.60}$ (CH_2)	1.66
	Unit 1 π (C3–CH_2)	0.15 (C3)<br>-0.60 (CH_2)	1.93	41% (C3)<br>59% (CH_2)	$sp^{1.00}$ (C3)<br>$sp^{1.00}$ (CH_2)	1.66
	Unit 2 σ (Ga–N1)	0.79 (Ga)<br>-0.99 (N1)	1.89	3% (Ga)<br>97% (N1)	$sp^{12.49}$ (Ga)<br>$sp^{3.17}$ (N1)	0.19
	Unit 2 σ (Ga–N2)	0.79 (Ga)<br>-1.03 (N2)	1.91	3% (Ga)<br>97% (N2)	$sp^{13.15}$ (Ga)<br>$sp^{3.15}$ (N2)	0.22
	Unit 2 σ (C1–C2)	0.20 (C1)<br>-0.35 (C2)	1.98	51% (C1)<br>49% (C2)	$sp^{1.61}$ (C1)<br>$sp^{1.63}$ (C2)	1.64
	Unit 2 π (C1–C2)	0.20 (C1)<br>-0.35 (C2)	1.87	44% (C1)<br>56% (C2)	$sp^{1.00}$ (C1)<br>$sp^{1.00}$ (C2)	1.64
	Unit 2 σ (C3–CH_2)	0.15 (C3)<br>-0.60 (CH_2)	1.98	52% (C3)<br>48% (CH_2)	$sp^{1.56}$ (C3)<br>$sp^{1.60}$ (CH_2)	1.66
	Unit 2 π (C3–CH_2)	0.15 (C3)<br>-0.60 (CH_2)	1.93	41% (C3)<br>59% (CH_2)	$sp^{1.00}$ (C3)<br>$sp^{1.00}$ (CH_2)	1.66

Table S22. Second order perturbation theory analysis for **2**$^-$ and **2·**M(crypt-222) (M = Li, Na, K, Cs).

**Structure**	**Donor NBO Occup.**	**Acceptor NBO Occup.**	**Energy / kcal mol^{-1}**
**2**$^-$	LP (N1)	p$_\perp$ (Ga)	5.71
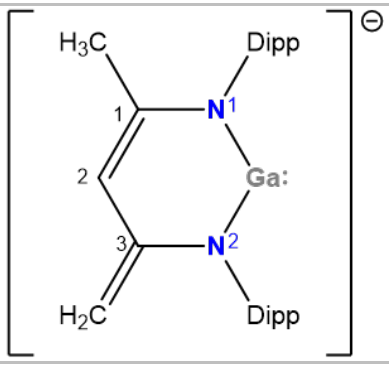	LP (N1)	π* (C1–C2)	57.57
	LP (N2)	p$_\perp$ (Ga)	7.52
	LP (N2)	π* (C3–CH_2)	58.75
	π (C1–C2)	π* (C3–CH_2)	20.89
	π (C3–CH_2)	π* (C1–C2)	12.26
**2·Li(crypt-222)**	LP (Ga)	s (Li)	0.11
	LP (N1)	π* (C1–C2)	51.99
	LP (N2)	p$_\parallel$ (Ga)	10.51
	σ (Ga–N2)	π* (C3–CH_2)	50.08
	π (C1–C2)	π* (C3–CH_2)	20.16
	π (C3–CH_2)	π* (C1–C2)	12.64
**2·Na(crypt-222)**	LP (Ga)	s (Na)	4.80
	LP (N1)	π* (C1–C2)	51.51
	LP (N1)	LV (Ga)	4.32
	LP (N2)	p$_\parallel$ (Ga)	5.92
	LP (N2)	π* (C3–CH_2)	53.47
	π (C1–C2)	π* (C3–CH_2)	19.97
	π (C3–CH_2)	π* (C1–C2)	12.60
**2·K(crypt-222)**	LP (Ga)	s (K)	6.27
	LP (N1)	p$_\perp$ (Ga)	4.44
	LP (N1)	π* (C1–C2)	52.10
	LP (N2)	p$_\perp$ (Ga)	5.99
	LP (N2)	π* (C3–CH_2)	53.85
	π (C1–C2)	π* (C3–CH_2)	20.15
	π (C3–CH_2)	π* (C1–C2)	12.66
**2·Cs(crypt-222)**	LP (Ga)	s (Cs)	4.38
	LP (N1)	p$_\perp$ (Ga)	4.36
	LP (N1)	π* (C1–C2)	51.99
	LP (N2)	p$_\perp$ (Ga)	5.88
	LP (N2)	π* (C3–CH_2)	53.79
	π (C1–C2)	π* (C3–CH_2)	20.15
	π (C3–CH_2)	π* (C1–C2)	12.70

Table S23. Second order perturbation theory analysis for **2·M(18-c-6)** (M = Na, K, Cs) and **2·K(18-c-6)(THF)**.

Structure	Donor NBO Occup.	Acceptor NBO Occup.	Energy / kcal mol^{-1}
**2·Na(18-c-6)**	LP (Ga)	s (Na)	20.76
	LP (N1)	p⊥ (Ga)	5.53
	LP (N1)	π* (C1–C2)	50.09
	LP (N2)	p⊥ (Ga)	6.91
	π (C1–C2)	π* (C3–CH_2)	19.76
	π (C3–CH_2)	π* (C1–C2)	12.98
**2·K(18-c-6)**	LP (Ga)	s (K)	15.98
	LP (N1)	p⊥ (Ga)	5.23
	LP (N1)	π* (C1–C2)	51.05
	LP (N2)	p⊥ (Ga)	6.71
	LP (N2)	π* (C3–CH_2)	51.98
	π (C1–C2)	π* (C3–CH_2)	19.98
	π (C3–CH_2)	π* (C1–C2)	12.73
**2·Cs(18-c-6)**	LP (Ga)	s (Cs)	14.45
	LP (N1)	π* (C1–C2)	53.21
	LP (N2)	p⊥ (Ga)	11.67
	σ (Ga–N2)	π* (C3–CH_2)	50.63
	π (C1–C2)	π* (C3–CH_2)	20.96
	π (C3–CH_2)	π* (C1–C2)	12.22
**2·K(18-c-6)(THF)**	LP (Ga)	s (K)	28.11
	LP (O(THF))	s (K)	3.99
	LP (N1)	p⊥ (Ga)	5.18
	LP (N1)	π* (C1–C2)	51.78
	LP (N2)	p⊥ (Ga)	6.46
	LP (N2)	π* (C3–CH_2)	51.64
	π (C1–C2)	π* (C3–CH_2)	19.93
	π (C3–CH_2)	π* (C1–C2)	12.88
**2·K(18-c-6)(THF)**[b]	LP (Ga)	s (K)	27.04
	LP (O(THF))	s (K)	4.03
	LP (N1)	p⊥ (Ga)	4.62
	LP (N1)	π* (C1–C2)	55.56
	LP (N2)	p⊥ (Ga)	6.06
	LP (N2)	π* (C3–CH_2)	54.89
	π (C1–C2)	π* (C3–CH_2)	20.38
	π (C3–CH_2)	π* (C1–C2)	12.77

Table S24. Second order perturbation theory analysis for ($\mathbf{2}$·M)$_2$ (M = K, Cs).

Structure	Donor NBO Occup.	Acceptor NBO Occup.	Energy / kcal mol^{-1}
**(2·K)$_2$**	LP (Ga1)	s (K1)	18.54
	LP (Ga1)	s (K2)	18.51
	Unit 1 LP (N1)	$p_{\perp}$ (Ga1)	5.33
	Unit 1 LP (N1)	π* (C1–C2)	49.19
	Unit 1 LP (N2)	$p_{\perp}$ (Ga1)	6.59
	Unit 1 LP (N2)	π* (C3–CH_2)	50.26
	Unit 1 π (C1–C2)	π* (C3–CH_2)	19.65
	Unit 1 π (C3–CH_2)	π* (C1–C2)	12.76
	LP (Ga2)	s (K1)	18.54
	LP (Ga2)	s (K2)	18.51
	Unit 2 LP (N1)	$p_{\perp}$ (Ga1)	5.33
	Unit 2 LP (N1)	π* (C1–C2)	49.20
	Unit 2 LP (N2)	$p_{\perp}$ (Ga1)	6.59
	Unit 2 LP (N2)	π* (C3–CH_2)	50.26
	Unit 2 π (C1–C2)	π* (C3–CH_2)	19.65
	Unit 2 π (C3–CH_2)	π* (C1–C2)	12.76
**(2·Cs)$_2$**	LP (Ga1)	s (Cs1)	8.36
	LP (Ga1)	s (Cs2)	8.14
	Unit 1 LP (N1)	$p_{\perp}$ (Ga1)	5.70
	Unit 1 LP (N1)	π* (C1–C2)	50.19
	Unit 1 LP (N2)	$p_{\perp}$ (Ga1)	6.85
	Unit 1 LP (N2)	π* (C3–CH_2)	50.34
	Unit 1 π (C1–C2)	π* (C3–CH_2)	19.91
	Unit 1 π (C3–CH_2)	π* (C1–C2)	12.77
	LP (Ga2)	s (Cs1)	8.14
	LP (Ga2)	s (Cs2)	8.36
	Unit 2 LP (N1)	$p_{\perp}$ (Ga1)	5.70
	Unit 2 LP (N1)	π* (C1–C2)	50.19
	Unit 2 LP (N2)	$p_{\perp}$ (Ga1)	6.85
	Unit 2 LP (N2)	π* (C3–CH_2)	50.34
	Unit 2 π (C1–C2)	π* (C3–CH_2)	19.91
	Unit 2 π (C3–CH_2)	π* (C1–C2)	12.77

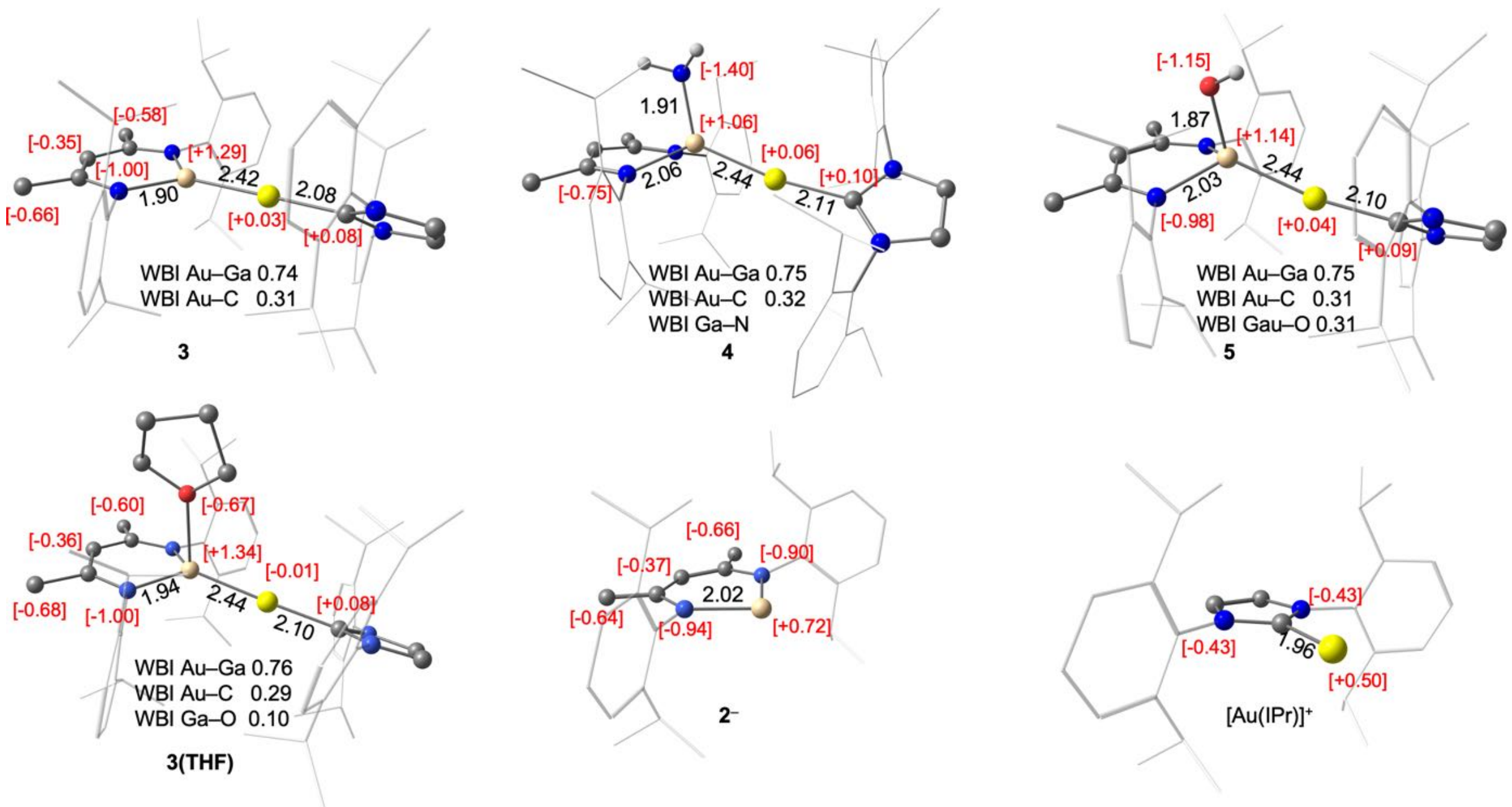

Figure S87. Calculated NPA charges (PBE0-D3(BJ)/def2-QZVP) on key atoms in complexes [Au(IPr)]$^+$, $\mathbf{2}^-$, **3**, **4** and **5** as well as in the THF-coordinated **3(THF)**. Wiberg Bond Indices and selected bond distances (in Å) are also given.

S6.1.3 QTAIM analysis

Table S25, Table S26, Table S27 and Table S28 contain selected properties of the bond critical points of all complexes. For reference, a low electron density $\rho(r)$, a positive Laplacian of the electron density $\nabla^2\rho(r)$, and kinetic and potential energy densities of either similar magnitudes or where there is a dominance of the kinetic energy density, all point towards closed shell ionic character of the bond. On the other hand, higher values of $\rho(r)$, negative values of $\nabla^2\rho(r)$ and a dominance of the potential energy density point towards covalent character. The ellipticity of a bond critical point, defined as $[(\lambda_1/\lambda_2) - 1]$, informs on the degree of single/multiple bond character. For example, an idealized single bond, λ_1/λ_2 is 1, leading to an ellipticity of 0. For an idealized double bond, λ_1/λ_2 is 2, leading to an ellipticity of 1. The ellipticity of a bond critical point can vary a significant amount from these ideal values, but nevertheless, can provide a useful metric for measure the degree of single/multiple bond character, in relative terms.

First, we analyze **2⁻**, the free anion. The Ga–N bonds exhibit low values of $\rho(r)$ (0.081 and 0.089 e/a_0^3), positive values of $\nabla^2\rho(r)$ (0.228 and 0.265 e/a_0^5) and slightly negative total energy densities (-0.026 and -0.030 E_h/a_0^3). These can be interpreted as N→Ga dative bonds, as the positions of the bond critical points lie slightly closer to the gallium center and the total energy densities are negative (due to a slightly larger magnitude of the potential energy density term). The N1–C1 and N2–C3 bonds possess larger values of $\rho(r)$ (0.312 and 0.304 e/a_0^3, respectively) and negative values of $\nabla^2\rho(r)$ (-0.983 and -0.939 e/a_0^5, respectively). Furthermore, the potential energy density dominates over the kinetic energy density, being over 3 times as large in magnitude. These bonds can be classified as possessing significant covalent character, and the same can be said of the bond critical points for C1–C2, C1–CH_3, C2–C3 and C3–CH_2. To provide insight into the degree of delocalization across the heterocyclic backbone, a discussion of the ellipticity values is in order. As a reference point, the C1–CH_3 bond critical point has an ellipticity of 0.049, close to the idealized value of zero for a single bond and thus can be taken as a measure of the ellipticity of a single bond in this context. The ellipticities of the Ga–N bonds and C2–C3 are raised from the reference value, while the ellipticities of the C1–C2 and C3–CH_2 bonds are far less than the idealized value of 1. Coupled with the observation that the C–C and C–N bonds lengths of the heterocycle are relatively close in length (see Table S12, Table S13, Table S14 and Table S15), we can conclude that there is at least partial delocalization present.

Due to the separated nature of **2·Li(crypt-222)**, there is predictably no bond critical point between the gallium and lithium centers. As a result, there is no appreciable change in any of the properties of the bond critical points of the gallyl anion. The same can be said of **2·K(crypt-222)**. However, in the case of **2·Cs(crypt-222)**, there is a bond critical point between Ga and Cs, but this is a negligible interaction due to the low $\rho(r)$ (0.001 e/a_0^3) and a total energy density of ~0 E_h/a_0^3.

For **2·Na(18-c-6)**, there is a bond critical point between the gallium and sodium centers. This interaction can be described as ionic, as evidenced by the low value of $\rho(r)$ (0.010 e/a_0^3), the positive value of $\nabla^2\rho$(r) (0.028 e/a_0^5) and equivalence of the kinetic and potential energy densities (-0.006 and 0.006 E_h/a_0^3, respectively). **2·K(18-c-6)** displays extremely similar properties of the bond critical points. One possibly noteworthy difference is the higher ellipticity value of the Ga···K BCP (0.027 vs 0.015, for K and Na, respectively). For **2·Cs(18-c-6)**, we can also ascribe ionic character to the Ga···Cs interaction.

When comparing the results for **2·K(18-c-6)(THF)** optimized in both the gas and solid state, there is only one notable difference. The magnitude of the properties of the K···O(THF) BCP for the solid-state optimized geometry are approximately double that of the gas state optimized geometry. This is unsurprising given the shorter K···O(THF) distance for the solid-state optimized structure (2.950 vs 3.283 Å for solid and gas phase, respectively). Furthermore, when comparing to **2·K(18-c-6)**, there are no notable differences of the BCP properties for the Ga···K interaction.

Moving on to $(\mathbf{2·K})_2$, bond critical points are observed between the two gallium centers, interestingly. However, this BCP has a low value of $\rho(r)$ (0.001 e/a_0^3), a marginally positive value of $\nabla^2\rho(r)$ (0.004 e/a_0^5) and the total energy density is 0.000 E_h/a_0^3, indicating a negligible interaction between the two gallium centers. The bond critical points for each of the Ga···K interactions all point towards an ionic situation, with low values of $\rho(r)$, slightly positive values of $\nabla^2\rho(r)$ and an equivalence of the kinetic and potential energy densities, which leads to total energy densities close to zero (0.001 E_h/a_0^3). For the Ga···Cs bond critical points in $(\mathbf{2·Cs})_2$ we can also assign significant ionic character. In contrast the to the potassium dimer, there is no bond critical point observed between the two Cs atoms in this dimer.

Table S25. Selected properties of the Bond Critical Points of free **2**$^{-}$ and **2·M(crypt-222)** (M = Li, Na, K, Cs). $\rho(r)$ is the electron density (e/a_0^3), $\nabla^2\rho(r)$ is the Laplacian of the electron density (e/a_0^5), λ_n are eigenvalues of the Hessian of $\rho(r)$ (e/a_0^5), ε is the bond ellipticity defined as (λ_1/λ_2)–1 and $V(r)$, $G(r)$ and $H(r)$ represent the potential, kinetic and total energy density, respectively (E_h/a_0^3).

Structure	BCP	$\rho(r)$	$\nabla^2\rho(r)$	λ_1	λ_2	λ_3	ε	$V(r)$	$G(r)$	$H(r)$
**2**$^{-}$	Ga–N1	0.081	0.228	-0.105	-0.094	0.427	0.119	-0.109	0.083	-0.026
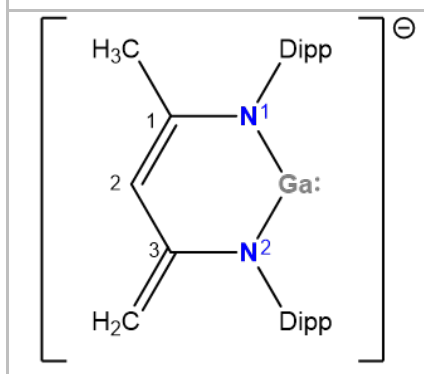	Ga–N2	0.089	0.265	-0.119	-0.104	0.488	0.141	-0.127	0.097	-0.030
	N1–C1	0.312	-0.983	-0.698	-0.613	0.327	0.140	-0.591	0.173	-0.418
	N2–C3	0.304	-0.939	-0.671	-0.604	0.336	0.111	-0.550	0.158	-0.392
	C1–C2	0.321	-0.936	-0.714	-0.531	0.309	0.343	-0.482	0.124	-0.358
	C1–CH_3	0.252	-0.643	-0.499	-0.476	0.333	0.049	-0.282	0.061	-0.221
	C2–C3	0.280	-0.779	-0.589	-0.518	0.328	0.136	-0.349	0.077	-0.272
	C3–CH_2	0.318	-0.914	-0.697	-0.527	0.310	0.321	-0.473	0.122	-0.351
**2·Li(crypt-222)**	Ga–N1	0.089	0.262	-0.119	-0.106	0.487	0.123	-0.127	0.096	-0.031
	Ga–N2	0.095	0.291	-0.129	-0.113	0.533	0.143	-0.140	0.107	-0.034
	N1–C1	0.309	-0.970	-0.687	-0.608	0.325	0.130	-0.583	0.170	-0.413
	N2–C3	0.299	-0.915	-0.656	-0.593	0.335	0.106	-0.536	0.154	-0.382
	C1–C2	0.323	-0.944	-0.719	-0.534	0.309	0.345	-0.487	0.126	-0.362
	C1–CH_3	0.253	-0.649	-0.502	-0.479	0.332	0.049	-0.285	0.061	-0.224
	C2–C3	0.280	-0.780	-0.588	-0.519	0.327	0.134	-0.350	0.077	-0.272
	C3–CH_2	0.320	-0.924	-0.703	-0.530	0.308	0.326	-0.479	0.124	-0.355
**2·Na(crypt-222)**	Ga–N1	0.088	0.257	-0.117	-0.104	0.478	0.122	-0.125	0.094	-0.030
	Ga–N2	0.095	0.292	-0.130	-0.114	0.535	0.143	-0.141	0.107	-0.034
	N1–C1	0.308	-0.966	-0.685	-0.606	0.326	0.129	-0.580	0.169	-0.410
	N2–C3	0.300	-0.923	-0.660	-0.597	0.334	0.106	-0.542	0.156	-0.386
	C1–C2	0.323	-0.943	-0.718	-0.534	0.309	0.345	-0.487	0.125	-0.361
	C1–CH_3	0.253	-0.647	-0.501	-0.478	0.332	0.049	-0.284	0.613	-0.223
	C2–C3	0.280	-0.780	-0.588	-0.519	0.327	0.133	-0.349	0.078	-0.272
	C3–CH_2	0.320	-0.927	-0.704	-0.531	0.308	0.326	-0.481	0.124	-0.356
**2·K(crypt-222)**	Ga–N1	0.087	0.251	-0.115	-0.102	0.468	0.123	-0.122	0.092	-0.029
	Ga–N2	0.094	0.286	-0.128	-0.112	0.525	0.143	-0.138	0.105	-0.033
	N1–C1	0.308	-0.963	-0.685	-0.605	0.326	0.131	-0.578	0.169	-0.409
	N2–C3	0.300	-0.923	-0.660	-0.597	0.334	0.106	-0.541	0.155	-0.386
	C1–C2	0.253	-0.646	-0.501	-0.477	0.332	0.049	-0.284	0.061	-0.223
	C1–CH_3	0.323	-0.944	-0.719	-0.534	0.309	0.345	-0.487	0.126	-0.362
	C2–C3	0.281	-0.783	-0.590	-0.520	0.327	0.133	-0.350	0.077	-0.273
	C3–CH_2	0.320	-0.925	-0.703	-0.530	0.308	0.325	-0.479	0.124	-0.355
**2·Cs(crypt-222)**	Ga–Cs	0.001	0.003	0.000	0.000	0.003	0.109	0.000	0.000	0.000
	Ga–N1	0.087	0.250	-0.114	-0.102	0.467	0.123	-0.121	0.092	-0.029
	Ga–N2	0.094	0.285	-0.127	-0.111	0.524	0.144	-0.138	0.105	-0.033
	N1–C1	0.308	-0.961	-0.684	-0.604	0.327	0.132	-0.576	0.168	-0.408
	N2–C3	0.300	-0.922	-0.660	-0.597	0.335	0.107	-0.541	0.155	-0.386
	C1–C2	0.323	-0.945	-0.719	-0.534	0.309	0.345	-0.488	0.126	-0.362
	C1–CH3	0.253	-0.646	-0.501	-0.477	0.332	0.049	-0.284	0.061	-0.223
	C2–C3	0.281	-0.784	-0.590	-0.521	0.327	0.133	-0.351	0.077	-0.273
	C3–CH2	0.320	-0.924	-0.703	-0.530	0.308	0.325	-0.479	0.124	-0.355

Table S26. Selected properties of the Bond Critical Points of **2·M(18-c-6)** (M = Na, K, Cs), **2·K(18-c-6)(THF)** and **2·Cs(18-c-6)$_2$**. $\rho(r)$ is the electron density (e/a_0^3), $\nabla^2\rho(r)$ is the Laplacian of the electron density (e/a_0^5), λ_n are eigenvalues of the Hessian of $\rho(r)$ (e/a_0^5), ε is the bond ellipticity defined as (λ_1/λ_2)–1 and $V(r)$, $G(r)$ and $H(r)$ represent the potential, kinetic and total energy density, respectively (E_h/a_0^3). [a]Periodic DFT-optimized geometry was used for analysis.

Structure	BCP	$\rho(r)$	$\nabla^2\rho(r)$	λ_1	λ_2	λ_3	ε	$V(r)$	$G(r)$	$H(r)$
**2·Na(18-c-6)**	Ga–Na	0.010	0.028	-0.007	-0.007	0.042	0.015	-0.006	0.006	0.001
	Ga–N1	0.092	0.274	-0.125	-0.111	0.509	0.126	-0.134	0.101	-0.033
	Ga–N2	0.099	0.308	-0.137	-0.120	0.566	0.145	-0.150	0.114	-0.037
	N1–C1	0.306	-0.953	-0.679	-0.601	0.327	0.129	-0.570	0.166	-0.404
	N2–C3	0.298	-0.907	-0.652	-0.590	0.335	0.105	-0.531	0.152	-0.379
	C1–C2	0.325	-0.952	-0.723	-0.537	0.308	0.346	-0.492	0.127	-0.365
	C1–CH_3	0.253	-0.648	-0.502	-0.478	0.332	0.049	-0.285	0.062	-0.224
	C2–C3	0.281	-0.786	-0.591	-0.522	0.327	0.132	-0.352	0.078	-0.274
	C3–CH_2	0.320	-0.928	-0.705	-0.531	0.308	0.328	-0.482	0.125	-0.357
**2·K(18-c-6)**	Ga–K	0.010	0.022	-0.006	-0.006	0.033	0.027	-0.004	0.005	0.001
	Ga–N1	0.091	0.271	-0.123	-0.110	0.504	0.123	-0.132	0.100	-0.032
	Ga–N2	0.098	0.307	-0.136	-0.119	0.562	0.144	-0.149	0.113	-0.036
	N1–C1	0.307	-0.961	-0.683	-0.605	0.326	0.129	-0.577	0.168	-0.409
	N2–C3	0.299	-0.917	-0.656	-0.594	0.333	0.105	-0.538	0.154	-0.384
	C1–C2	0.323	-0.945	-0.719	-0.534	0.309	0.346	-0.488	0.126	-0.362
	C1–CH_3	0.253	-0.649	-0.502	-0.479	0.332	0.049	-0.285	0.061	-0.224
	C2–C3	0.280	-0.781	-0.589	-0.519	0.327	0.133	-0.350	0.077	-0.272
	C3–CH_2	0.320	-0.928	-0.705	-0.531	0.308	0.327	-0.482	0.125	-0.357
**2·Cs(18-c-6)**	Ga–Cs	0.010	0.018	-0.005	-0.005	0.029	0.060	-0.004	0.004	0.000
	Ga–N1	0.095	0.287	-0.130	-0.115	0.532	0.124	-0.140	0.106	-0.034
	Ga–N2	0.095	0.296	-0.130	-0.114	0.540	0.137	-0.142	0.108	-0.034
	N1–C1	0.309	-0.972	-0.688	-0.609	0.324	0.131	-0.586	0.171	-0.414
	N2–C3	0.300	-0.924	-0.657	-0.596	0.329	0.101	-0.546	0.158	-0.389
	C1–C2	0.322	-0.936	-0.714	-0.531	0.309	0.344	-0.483	0.125	-0.359
	C1–CH_3	0.253	-0.651	-0.503	-0.480	0.332	0.048	-0.286	0.062	-0.224
	C2–C3	0.280	-0.780	-0.589	-0.518	0.327	0.137	-0.350	0.078	-0.272
	C3–CH_2	0.320	-0.926	-0.704	-0.530	0.308	0.328	-0.481	0.125	-0.356
**2·K(18-c-6)(THF)**	Ga···K	0.009	0.018	-0.005	-0.005	0.027	0.031	-0.003	0.004	0.001
	K···O(THF)	0.004	0.020	-0.003	-0.003	0.026	0.053	-0.002	0.004	0.001
	Ga–N1	0.091	0.271	-0.123	-0.110	0.504	0.124	-0.132	0.100	-0.032
	Ga–N2	0.099	0.309	-0.137	-0.120	0.566	0.145	-0.150	0.114	-0.036
	N1–C1	0.307	-0.963	-0.683	-0.604	0.325	0.131	-0.579	0.169	-0.410
	N2–C3	0.298	-0.911	-0.654	-0.592	0.335	0.105	-0.533	0.153	-0.381
	C1–C2	0.253	-0.650	-0.503	-0.479	0.332	0.049	-0.286	0.062	-0.224
	C1–CH_3	0.324	-0.946	-0.720	-0.535	0.308	0.346	-0.489	0.126	-0.363
	C2–C3	0.280	-0.781	-0.588	-0.519	0.327	0.133	-0.350	0.077	-0.272
	C3–CH_2	0.320	-0.928	-0.705	-0.531	0.308	0.327	-0.482	0.125	-0.357
**2·K(18-c-6)(THF)**[a]	Ga···K	0.008	0.018	-0.005	-0.005	0.027	0.019	-0.003	0.004	0.001
	K···O(THF)	0.009	0.045	-0.009	-0.008	0.062	0.103	-0.006	0.009	0.002
	Ga–N1	0.084	0.238	-0.110	-0.098	0.445	0.124	-0.115	0.087	-0.028
	Ga–N2	0.090	0.261	-0.120	-0.105	0.486	0.148	-0.128	0.097	-0.031
	N1–C1	0.316	-1.022	-0.710	-0.629	0.317	0.130	-0.633	0.189	-0.444
	N2–C3	0.305	-0.959	-0.674	-0.611	0.326	0.103	-0.569	0.165	-0.404
	C1–C2	0.326	-0.960	-0.726	-0.540	0.307	0.344	-0.495	0.128	-0.368
	C1–CH_3	0.255	-0.660	-0.508	-0.484	0.332	0.049	-0.290	0.062	-0.227
	C2–C3	0.283	-0.798	-0.598	-0.528	0.327	0.133	-0.356	0.078	-0.278
	C3–CH_2	0.322	-0.937	-0.709	-0.535	0.307	0.325	-0.486	0.126	-0.360
**2·Cs(18-c-6)$_2$**	Ga–N1	0.087	0.255	-0.116	-0.104	0.474	0.120	-0.123	0.094	-0.030
	Ga–N2	0.094	0.290	-0.129	-0.113	0.532	0.141	-0.140	0.106	-0.034
	N1–C1	0.309	-0.974	-0.688	-0.610	0.325	0.128	-0.585	0.171	-0.414
	N2–C3	0.302	-0.934	-0.665	-0.601	0.332	0.106	-0.549	0.158	-0.391
	C1–C2	0.322	-0.938	-0.716	-0.532	0.310	0.345	-0.484	0.125	-0.359
	C1–CH_3	0.253	-0.646	-0.501	-0.477	0.332	0.050	-0.284	0.061	-0.223
	C2–C3	0.279	-0.775	-0.585	-0.517	0.327	0.132	-0.347	0.076	-0.270
	C3–CH_2	0.320	-0.927	-0.704	-0.531	0.309	0.326	-0.480	0.124	-0.356

Table S27. Selected properties of the Bond Critical Points of **(2·M)₂** (M = K, Cs). $\rho(r)$ is the electron density (e/a_0^3), $\nabla^2\rho(r)$ is the Laplacian of the electron density (e/a_0^5), λ_n are eigenvalues of the Hessian of $\rho(r)$ (e/a_0^5), ε is the bond ellipticity defined as (λ_1/λ_2)–1 and $V(r)$, $G(r)$ and $H(r)$ represent the potential, kinetic and total energy density, respectively (E_h/a_0^3).

Structure	BCP	$\rho(r)$	$\nabla^2\rho(r)$	λ_1	λ_2	λ_3	ε	$V(r)$	$G(r)$	$H(r)$
**$(2\cdot K)_2$**	Ga1···Ga2	0.003	0.004	-0.001	-0.001	0.005	0.954	-0.001	0.001	0.000
	Ga1···K1	0.009	0.019	-0.005	-0.005	0.029	0.015	-0.004	0.004	0.001
	Ga1···K2	0.008	0.019	-0.005	-0.005	0.028	0.014	-0.004	0.004	0.001
	Ga2···K1	0.009	0.019	-0.005	-0.005	0.029	0.015	-0.004	0.004	0.001
	Ga2···K2	0.008	0.019	-0.005	-0.005	0.028	0.014	-0.004	0.004	0.001
	Unit 1 (Ga–N1)	0.096	0.292	-0.131	-0.117	0.540	0.121	-0.143	0.108	-0.035
	Unit 1 (Ga–N2)	0.102	0.327	-0.143	-0.126	0.596	0.142	-0.159	0.120	-0.039
	Unit 1 (N1–C1)	0.305	-0.955	-0.675	-0.601	0.321	0.124	-0.579	0.170	-0.409
	Unit 1 (N2–C3)	0.298	-0.917	-0.652	-0.592	0.328	0.101	-0.543	0.157	-0.386
	Unit 1 (C1–C2)	0.323	-0.943	-0.718	-0.533	0.308	0.348	-0.488	0.126	-0.362
	Unit 1 (C1–CH_3)	0.254	-0.654	-0.504	-0.481	0.332	0.049	-0.287	0.062	-0.225
	Unit 1 (C2–C3)	0.279	-0.775	-0.585	-0.517	0.327	0.132	-0.347	0.077	-0.270
	Unit 1 (C3–CH_2)	0.322	-0.939	-0.711	-0.535	0.307	0.330	-0.488	0.127	-0.361
	Unit 2 (Ga–N1)	0.096	0.292	-0.131	-0.117	0.540	0.121	-0.143	0.108	-0.035
	Unit 2 (Ga–N2)	0.102	0.327	-0.143	-0.126	0.596	0.142	-0.159	0.120	-0.039
	Unit 2 (N1–C1)	0.305	-0.955	-0.675	-0.601	0.321	0.124	-0.579	0.170	-0.409
	Unit 2 (N2–C3)	0.298	-0.917	-0.652	-0.593	0.328	0.101	-0.543	0.157	-0.386
	Unit 2 (C1–C2)	0.323	-0.943	-0.718	-0.533	0.308	0.348	-0.488	0.126	-0.362
	Unit 2 (C1–CH_3)	0.254	-0.654	-0.504	-0.481	0.332	0.049	-0.287	0.062	-0.225
	Unit 2 (C2–C3)	0.279	-0.775	-0.585	-0.517	0.327	0.132	-0.347	0.077	-0.270
	Unit 2 (C3–CH_2)	0.322	-0.939	-0.711	-0.535	0.307	0.330	-0.488	0.127	-0.361
**$(2\cdot Cs)_2$**	Ga1···Cs1	0.009	0.016	-0.005	-0.004	0.025	0.049	-0.003	0.004	0.000
	Ga1···Cs2	0.009	0.016	-0.004	-0.004	0.025	0.037	-0.003	0.004	0.000
	Ga2···Cs1	0.009	0.016	-0.004	-0.004	0.025	0.037	-0.003	0.004	0.000
	Ga2···Cs2	0.009	0.016	-0.005	-0.004	0.025	0.049	-0.003	0.004	0.000
	Unit 1 (Ga–N1)	0.095	0.288	-0.129	-0.115	0.532	0.122	-0.140	0.106	-0.034
	Unit 1 (Ga–N2)	0.101	0.320	-0.141	-0.123	0.584	0.142	-0.155	0.118	-0.038
	Unit 1 (N1–C1)	0.305	-0.951	-0.675	-0.599	0.323	0.127	-0.574	0.168	-0.406
	Unit 1 (N2–C3)	0.297	-0.911	-0.650	-0.591	0.330	0.101	-0.538	0.155	-0.383
	Unit 1 (C1–C2)	0.323	-0.944	-0.719	-0.533	0.308	0.348	-0.489	0.126	-0.362
	Unit 1 (C1–CH_3)	0.254	-0.653	-0.504	-0.481	0.332	0.048	-0.287	0.062	-0.225
	Unit 1 (C2–C3)	0.280	-0.778	-0.587	-0.518	0.327	0.133	-0.349	0.077	-0.272
	Unit 1 (C3–CH_2)	0.322	-0.935	-0.709	-0.533	0.307	0.329	-0.486	0.126	-0.360
	Unit 2 (Ga–N1)	0.095	0.288	-0.129	-0.115	0.532	0.122	-0.140	0.106	-0.034
	Unit 2 (Ga–N2)	0.101	0.320	-0.140	-0.123	0.584	0.142	-0.155	0.118	-0.038
	Unit 2 (N1–C1)	0.305	-0.951	-0.675	-0.599	0.323	0.127	-0.574	0.168	-0.406
	Unit 2 (N2–C3)	0.297	-0.911	-0.650	-0.591	0.330	0.101	-0.538	0.155	-0.383
	Unit 2 (C1–C2)	0.323	-0.944	-0.719	-0.533	0.308	0.348	-0.489	0.126	-0.362
	Unit 2 (C1–CH_3)	0.254	-0.653	-0.504	-0.481	0.332	0.048	-0.287	0.062	-0.225
	Unit 2 (C2–C3)	0.280	-0.778	-0.587	-0.518	0.327	0.133	-0.349	0.077	-0.272
	Unit 2 (C3–CH_2)	0.322	-0.935	-0.709	-0.533	0.307	0.329	-0.486	0.126	-0.360

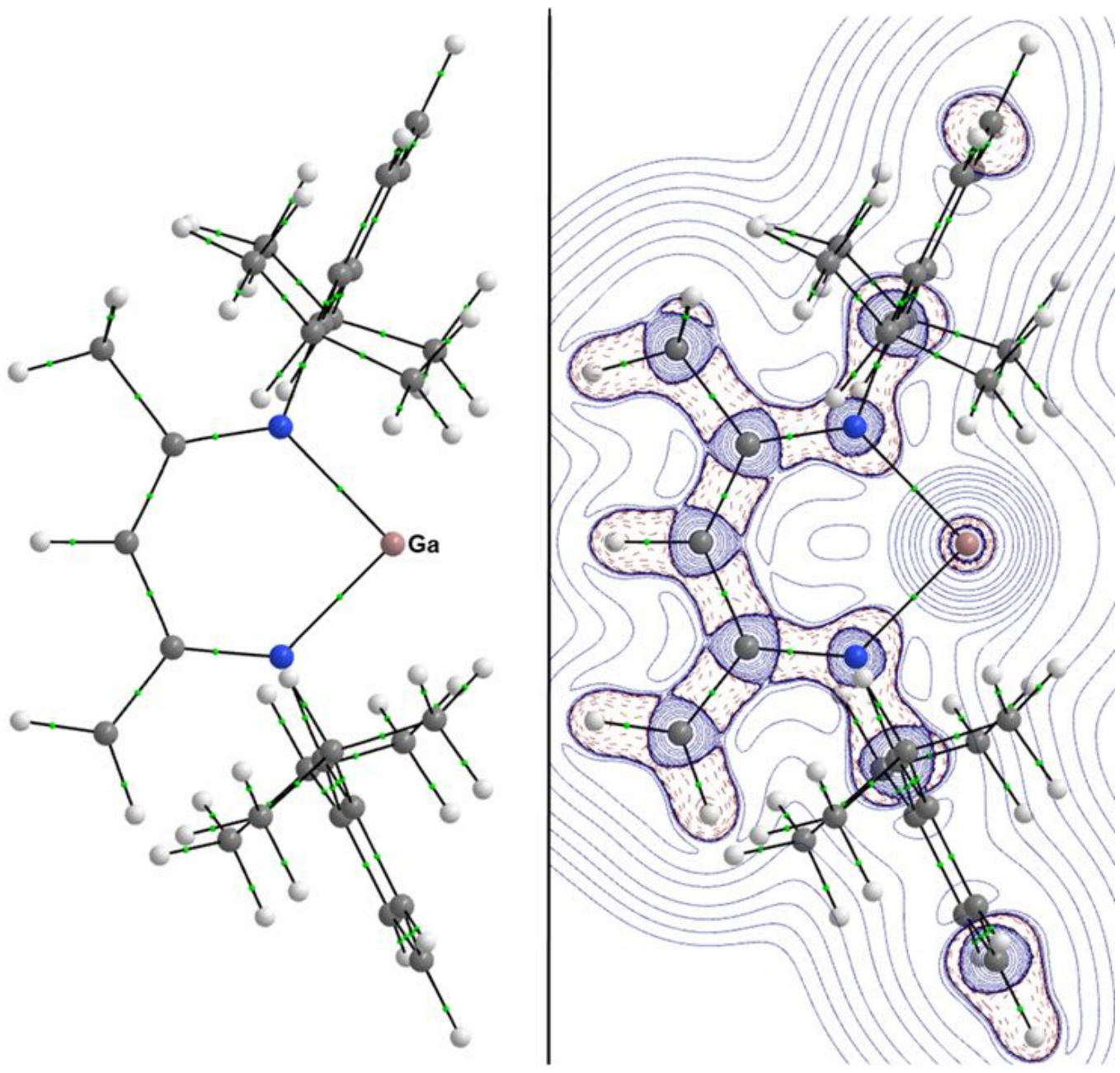

Figure S88. Molecular graph (left) and negative Laplacian $-\nabla^2\rho(r)$ of the electron density in the plane containing Ga, N1 and N2 (right) for **2**$^{-}$.

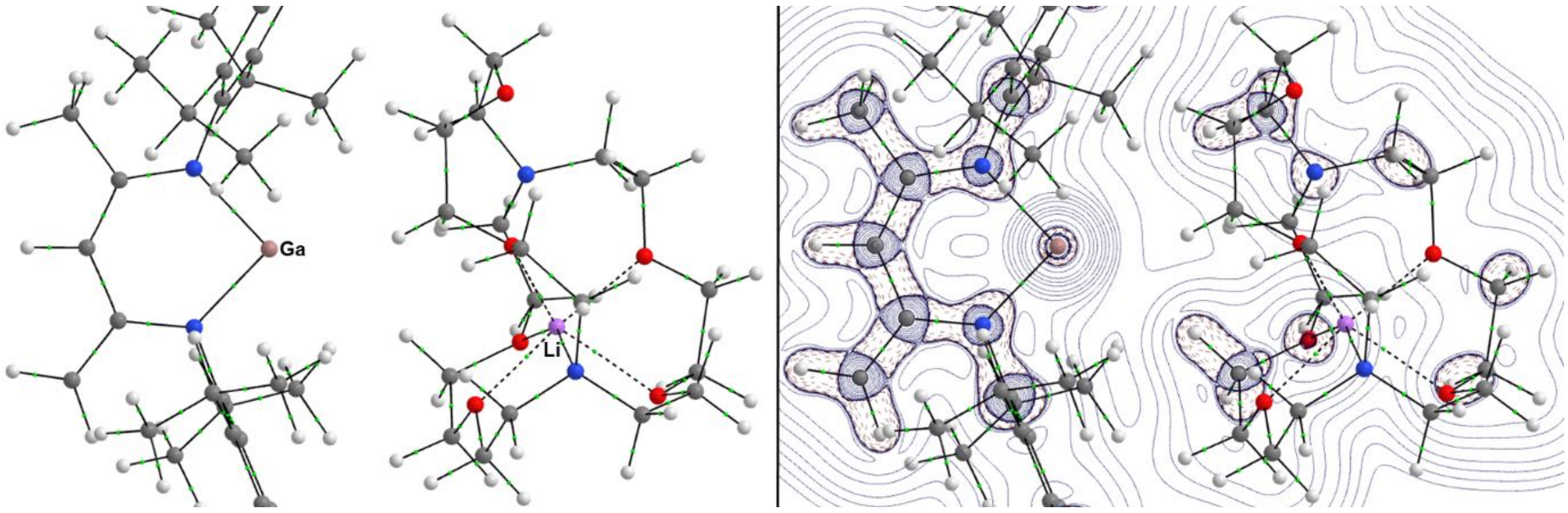

Figure S89. Molecular graph (left) and negative Laplacian $-\nabla^2\rho(r)$ of the electron density in the plane containing Ga, N1 and N2 (right) for **2·Li(crypt-222)**.

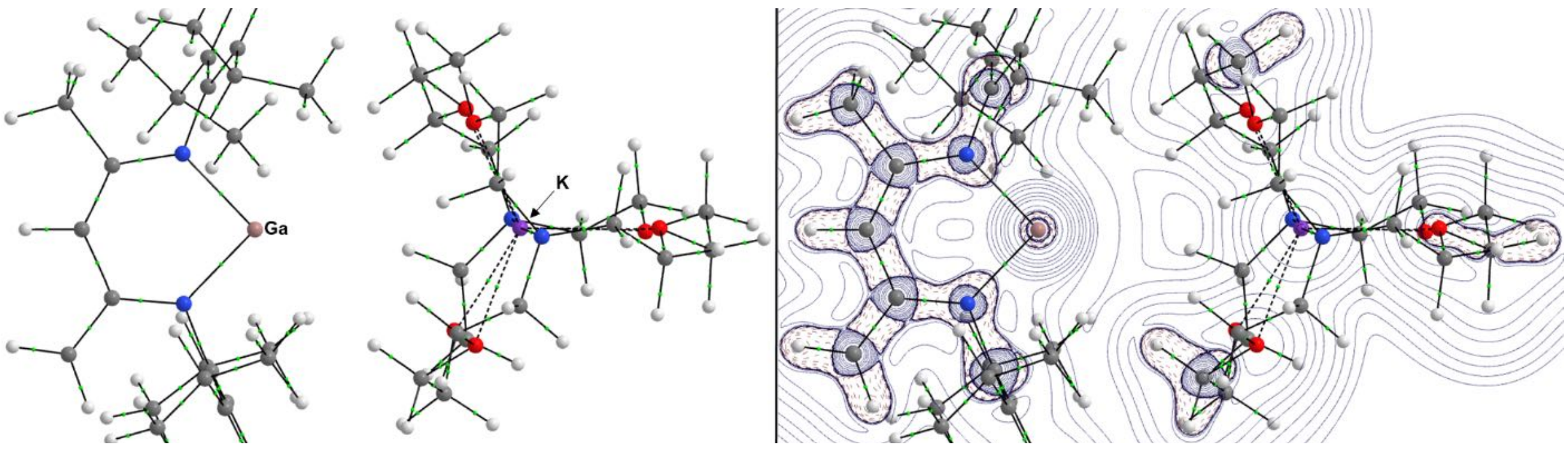

Figure S90. Molecular graph (left) and negative Laplacian $-\nabla^2\rho(r)$ of the electron density in the plane containing Ga, N1 and N2 (right) for **2·K(crypt-222)**.

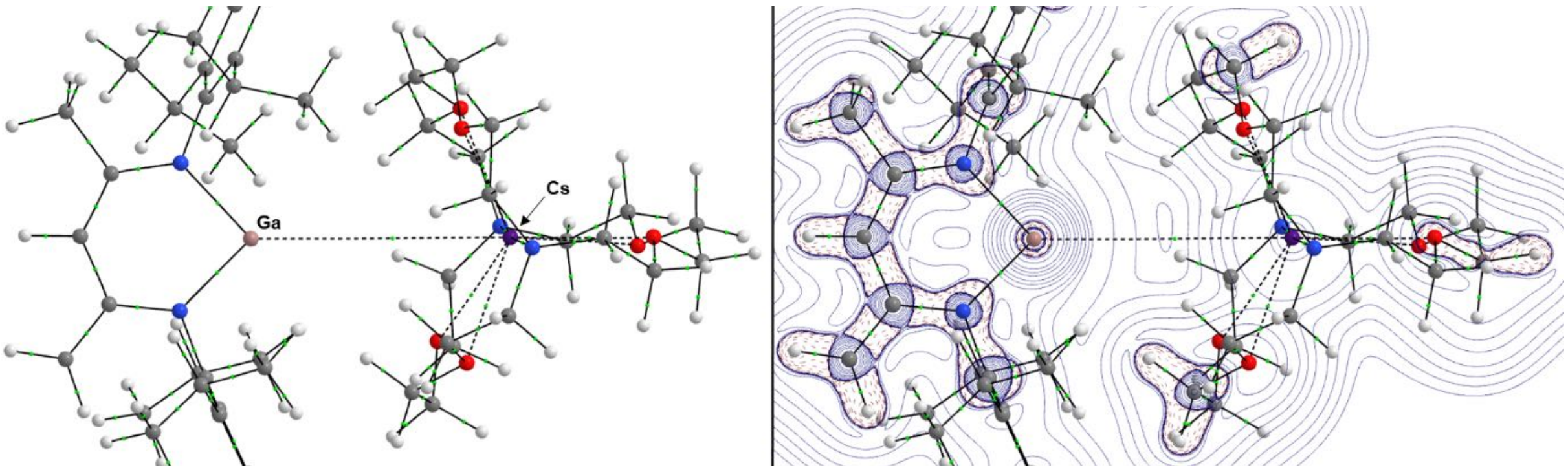

Figure S91. Molecular graph (left) and negative Laplacian $-\nabla^2\rho(r)$ of the electron density in the plane containing Ga1, N1 and N2 (right) for **2·Cs(crypt-222)**.

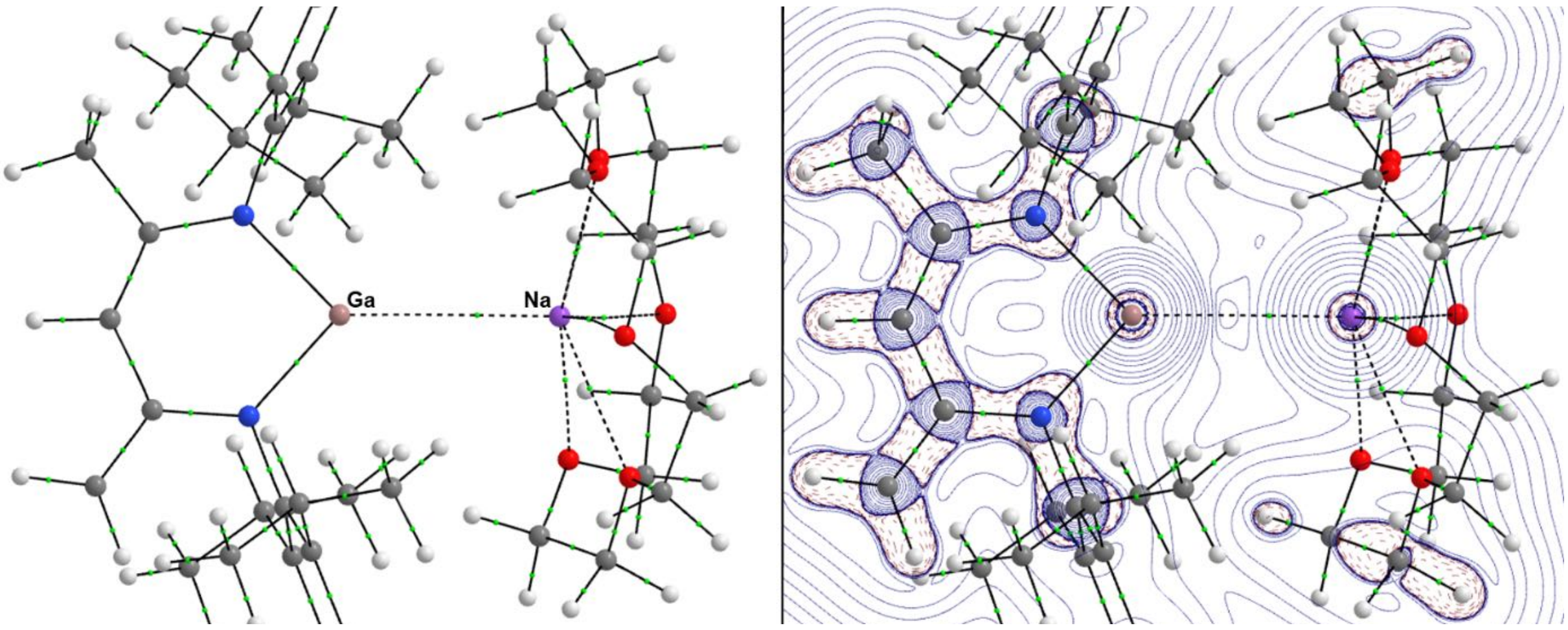

Figure S92. Molecular graph (left) and negative Laplacian $-\nabla^2\rho(r)$ of the electron density in the plane containing Ga, N1 and N2 (right) for **2·Na(18-c-6)**.

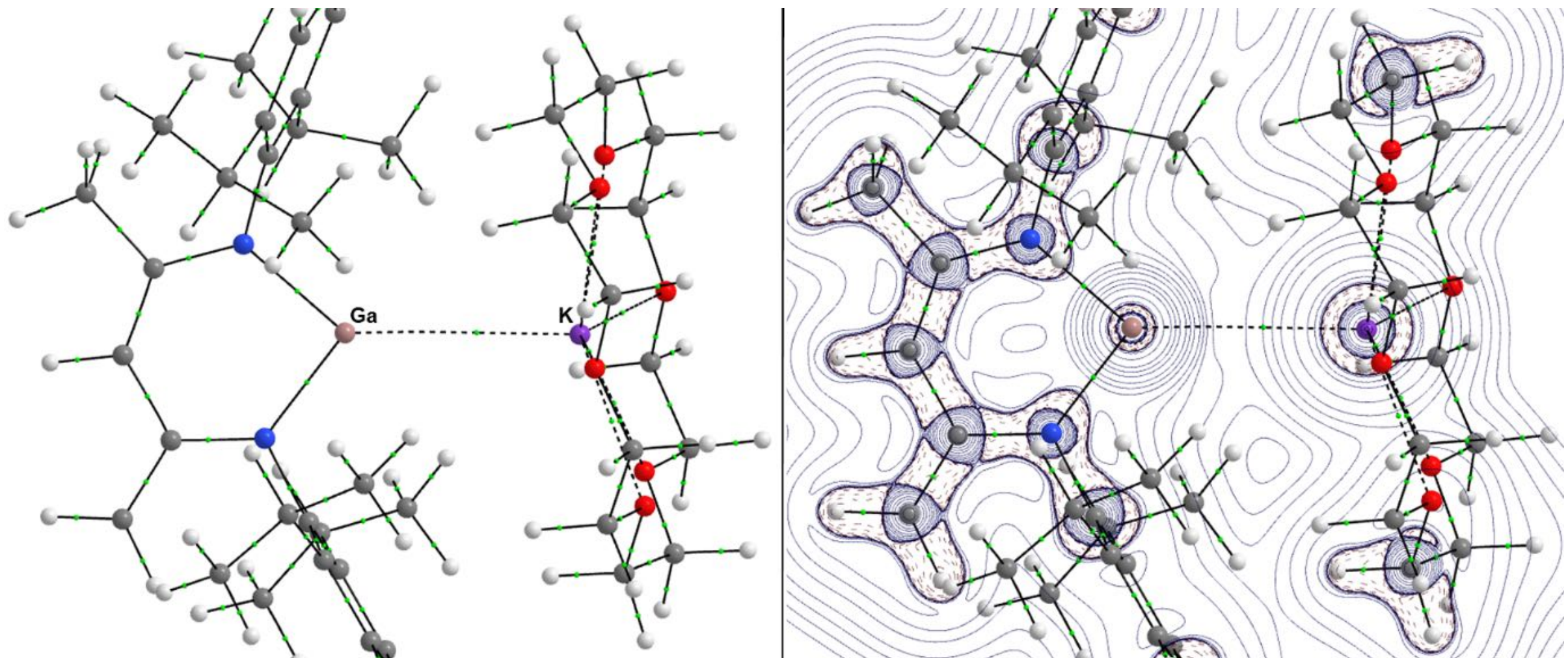

Figure S93. Molecular graph (left) and negative Laplacian $-\nabla^2\rho(r)$ of the electron density in the plane containing Ga, N1 and N2 (right) for **2·K(18-c-6)**.

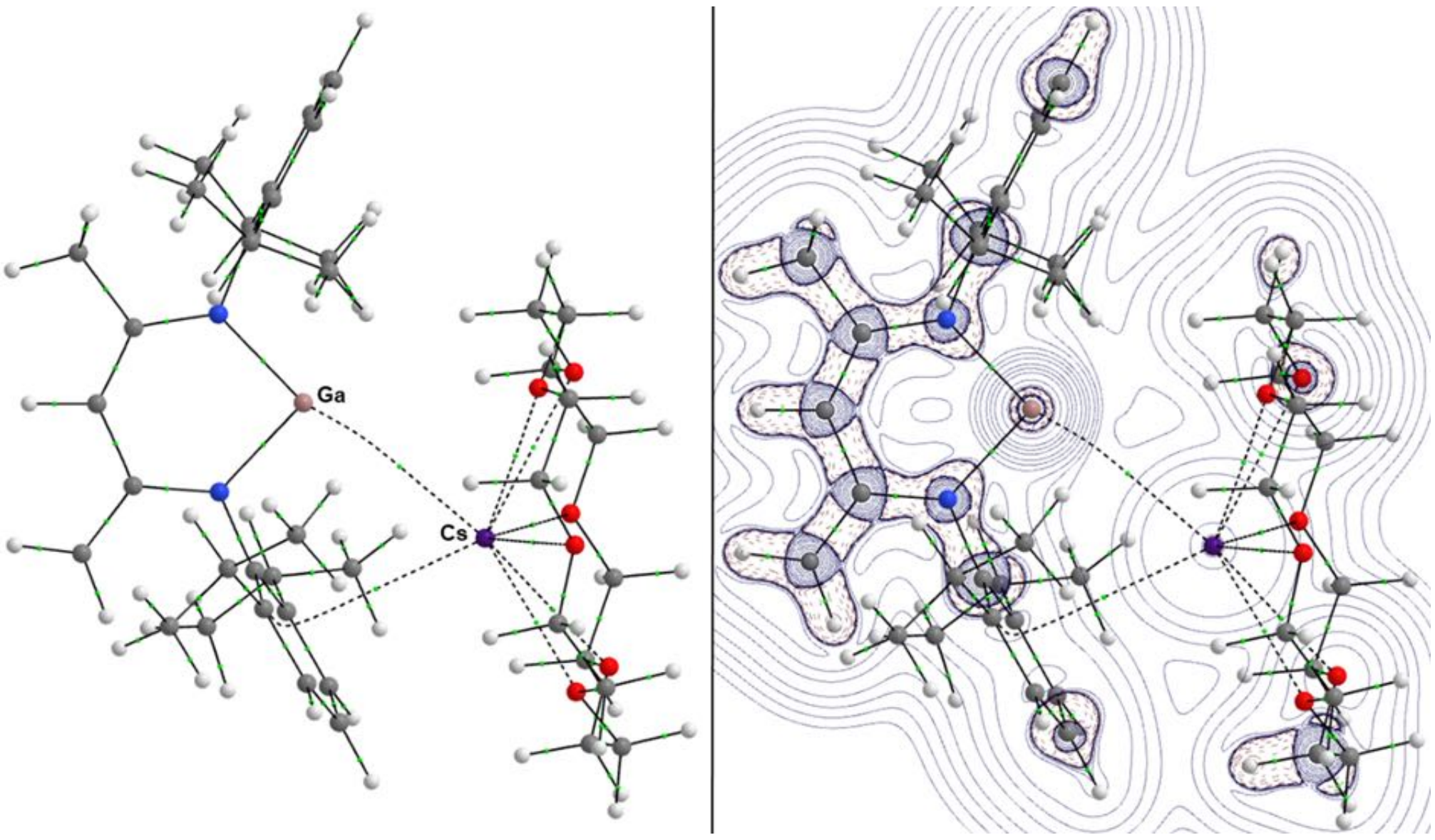

Figure S94. Molecular graph (left) and negative Laplacian $-\nabla^2\rho(r)$ of the electron density in the plane containing Ga, N1 and N2 (right) for **2·Cs(18-c-6)**.

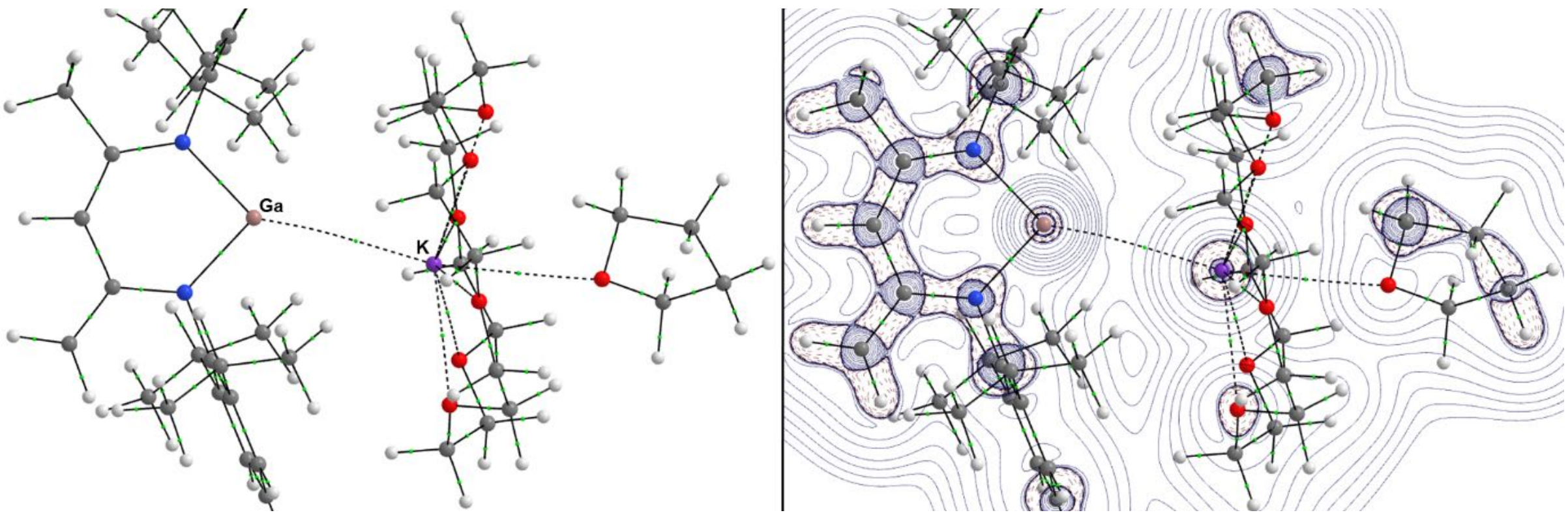

Figure S95. Molecular graph (left) and Laplacian $-\nabla^2\rho(r)$ of the electron density in the plane containing Ga, N1 and N2 (right) for **2·K(18-c-6)(THF)**.

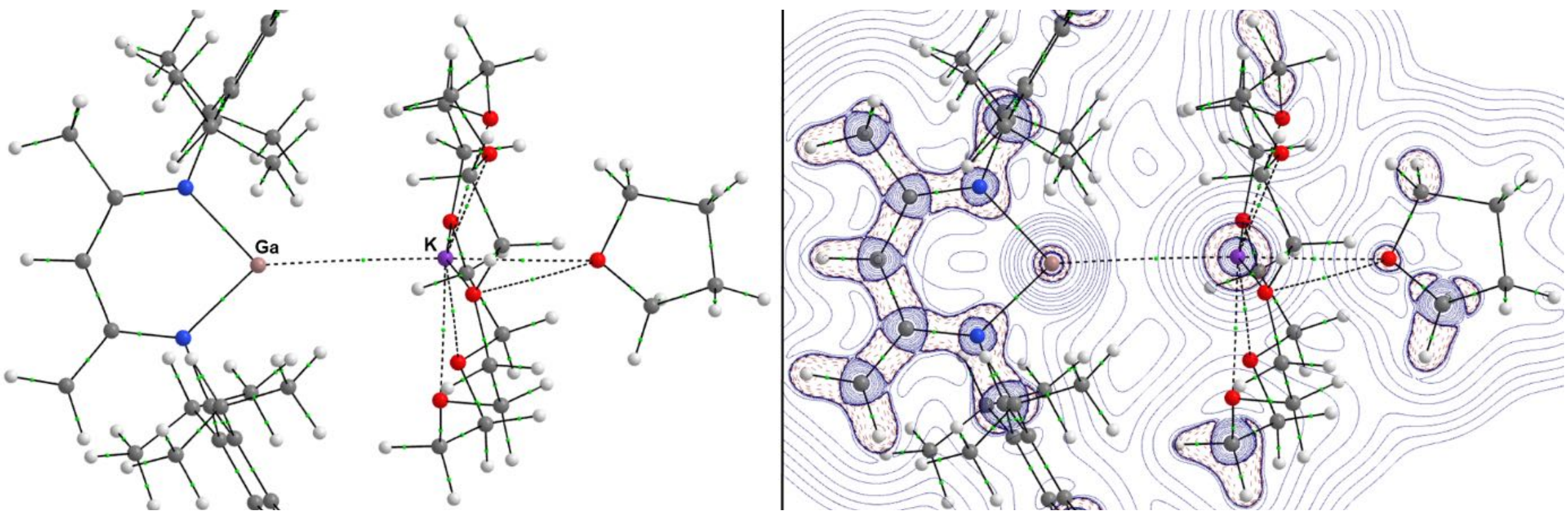

Figure S96. Molecular graph (left) and negative Laplacian $-\nabla^2\rho(r)$ of the electron density in the plane containing Ga, N1 and N2 (right) for solid state optimized **2·K(18-c-6)(THF)**.

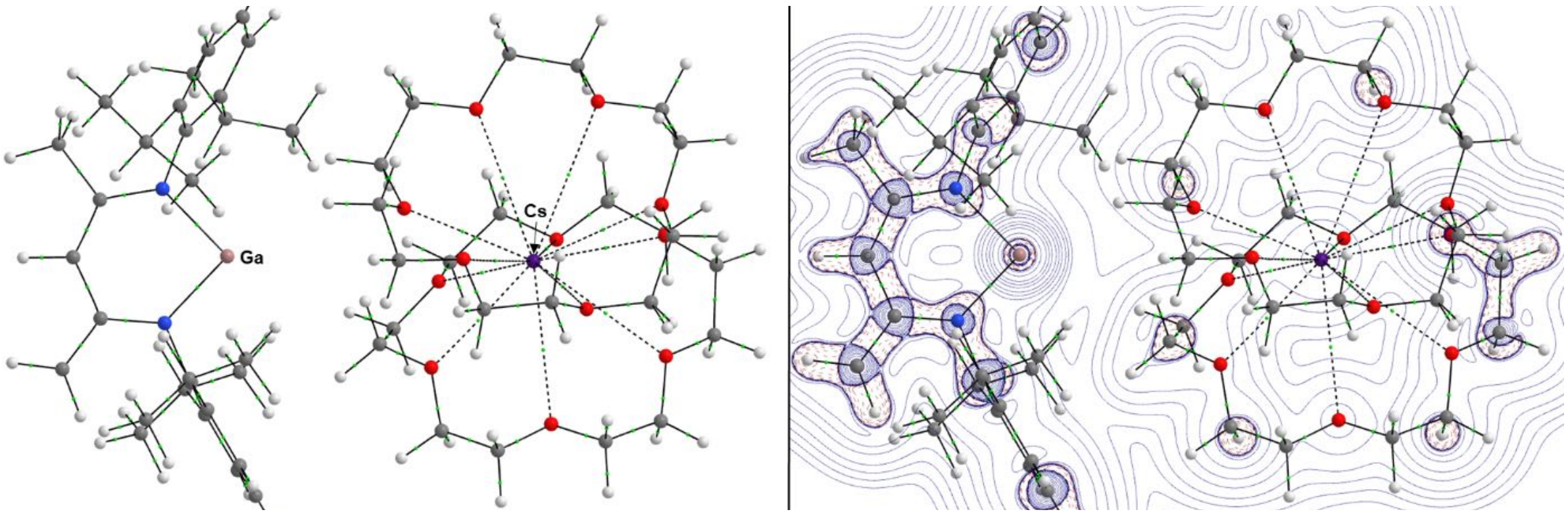

Figure S97. Molecular graph (left) and negative Laplacian $-\nabla^2\rho(r)$ of the electron density in the plane containing Ga1, N1 and N2 (right) for **2·Cs(18-c-6)$_2$**.

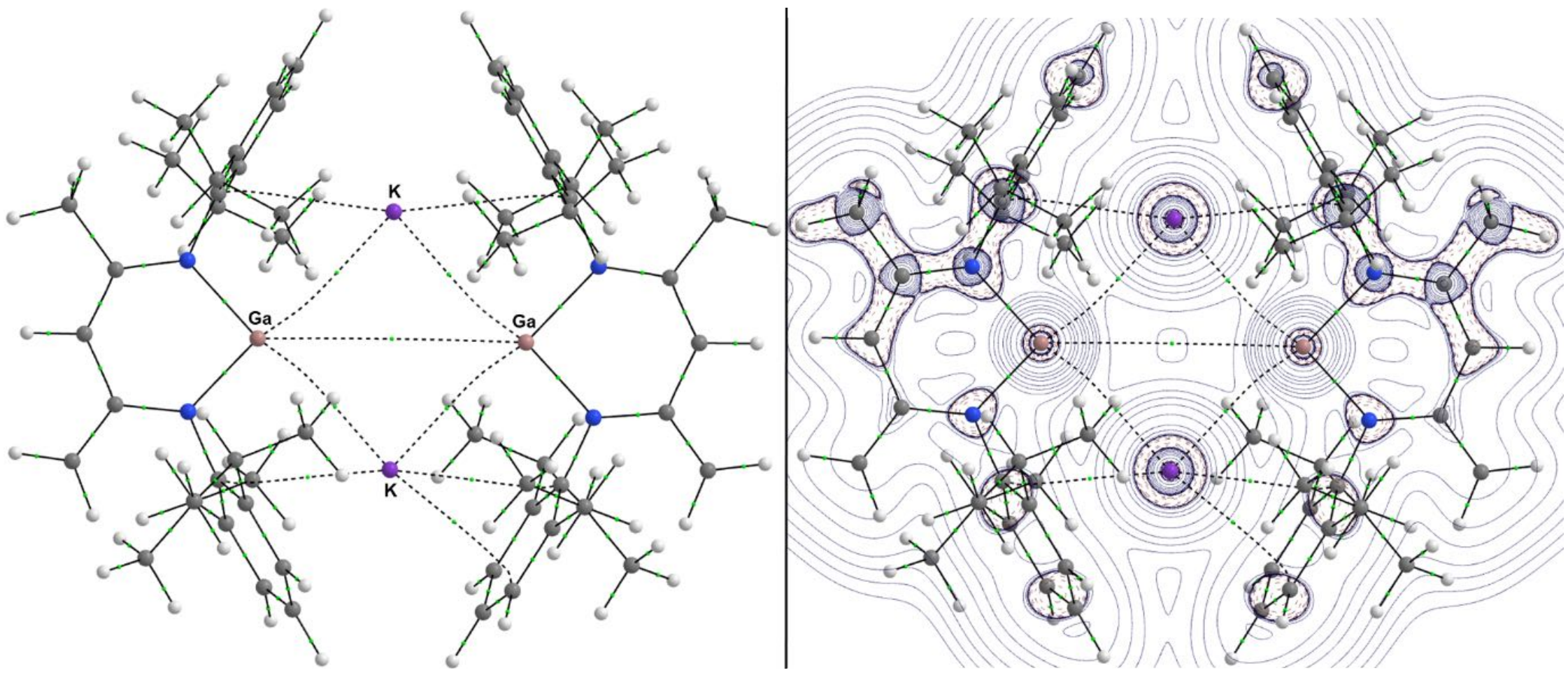

Figure S98. Molecular graph (left) and negative Laplacian $-\nabla^2\rho(r)$ of the electron density in the plane containing Ga1, K1 and Ga2 (right) for **(2·K)$_2$**.

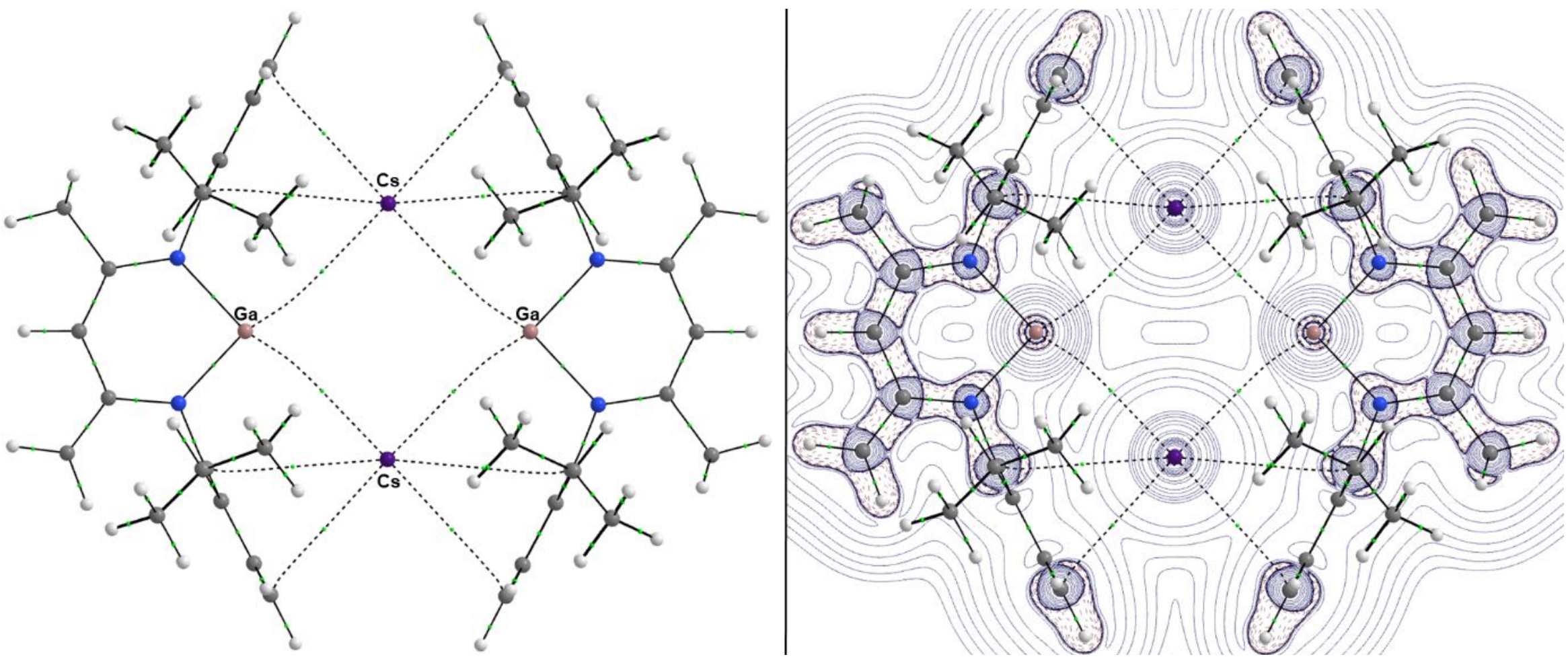

Figure S99. Molecular graph (left) and negative Laplacian $-\nabla^2\rho(r)$ of the electron density in the plane containing Ga1, K1 and Ga2 (right) for **(2·Cs)$_2$**.

Table S28. Selected properties of the Bond Critical Points of gallyl and aluminyl gold complexes. $\rho(r)$ is the electron density (e/a_0^3), $\nabla^2\rho(r)$ is the Laplacian of the electron density (e/a_0^5), λ_n are eigenvalues of the Hessian of $\rho(r)$ (e/a_0^5), ε is the bond ellipticity defined as (λ_1/λ_2)–1 and $V(r)$, $G(r)$ and $H(r)$ represent the potential, kinetic and total energy density, respectively (E_h/a_0^3).

Structure	BCP	$\rho(r)$	$\nabla^2\rho(r)$	λ_1	λ_2	λ_3	ε	$V(r)$	$G(r)$	$H(r)$
**3**	Ga–Au	0.086	0.033	-0.069	-0.069	0.170	0.001	-0.080	0.044	-0.036
	C–Au	0.127	0.222	-0.147	-0.140	0.509	0.0537	-0.179	0.117	-0.062
	Ga–N1	0.117	0.416	-0.175	-0.157	0.748	0.119	-0.198	0.151	-0.047
	Ga–N2	0.123	0.443	-0.185	-0.163	0.792	0.135	-0.213	0.162	-0.051
	N1–C1	0.296	-0.907	-0.616	-0.563	0.272	0.096	-0.592	0.183	-0.409
	N2–C3	0.288	-0.872	-0.594	-0.555	0.277	0.071	-0.555	0.168	-0.386
	C1–C2	0.313	-0.765	-0.640	-0.456	0.331	0.404	-0.425	0.117	-0.308
	C1–CH_3	0.250	-0.584	-0.464	-0.442	0.322	0.051	-0.260	0.057	-0.203
	C2–C3	0.272	-0.658	-0.530	-0.465	0.337	0.141	-0.304	0.070	-0.234
	C3–CH_2	0.312	-0.766	-0.638	-0.456	0.328	0.401	-0.427	0.118	-0.309
**4**	Ga–Au	0.084	0.027	-0.069	-0.068	0.164	0.003	-0.077	0.042	-0.035
	C–Au	0.120	0.228	-0.136	-0.129	0.493	0.056	-0.168	0.112	-0.055
	Ga–N	0.118	0.399	-0.172	-0.160	0.731	0.080	-0.195	0.147	-0.047
	Ga–N1	0.085	0.280	-0.117	-0.110	0.507	0.062	-0.119	0.094	-0.024
	Ga–N2	0.082	0.266	-0.111	-0.104	0.481	0.065	-0.111	0.089	-0.023
	N1–C1	0.335	-1.010	-0.731	-0.646	0.367	0.132	-0.834	0.290	-0.543
	N2–C3	0.335	-0.990	-0.732	-0.646	0.389	0.133	-0.848	0.300	-0.548
	C1–C2	0.293	-0.715	-0.587	-0.466	0.338	0.257	-0.363	0.092	-0.271
	C1–CH_3	0.250	-0.586	-0.464	-0.446	0.324	0.039	-0.260	0.057	-0.203
	C2–C3	0.294	-0.718	-0.588	-0.468	0.337	0.257	-0.364	0.092	-0.272
	C3–CH_3	0.251	-0.587	-0.464	-0.447	0.324	0.038	-0.260	0.057	-0.204
**5**	Ga–Au	0.084	0.029	-0.068	-0.068	0.164	0.007	-0.077	0.042	-0.035
	C–Au	0.121	0.228	-0.138	-0.130	0.496	0.056	-0.169	0.113	-0.056
	Ga–O	0.114	0.531	-0.178	-0.169	0.879	0.055	-0.203	0.168	-0.035
	Ga–N1	0.089	0.300	-0.125	-0.119	0.543	0.055	-0.129	0.101	-0.027
	Ga–N2	0.088	0.296	-0.123	-0.116	0.536	0.059	-0.127	0.100	-0.026
	N1–C1	0.334	-0.987	-0.729	-0.647	0.389	0.126	-0.844	0.299	-0.546
	N2–C3	0.333	-1.000	-0.726	-0.645	0.371	0.125	-0.831	0.291	-0.541
	C1–C2	0.295	-0.726	-0.592	-0.472	0.338	0.255	-0.367	0.093	-0.274
	C1–CH_3	0.251	-0.590	-0.466	-0.449	0.325	0.038	-0.261	0.057	-0.204
	C2–C3	0.295	-0.725	-0.593	-0.471	0.338	0.260	-0.368	0.093	-0.275
	C3–CH_3	0.251	-0.588	-0.465	-0.447	0.324	0.039	-0.261	0.057	-0.204

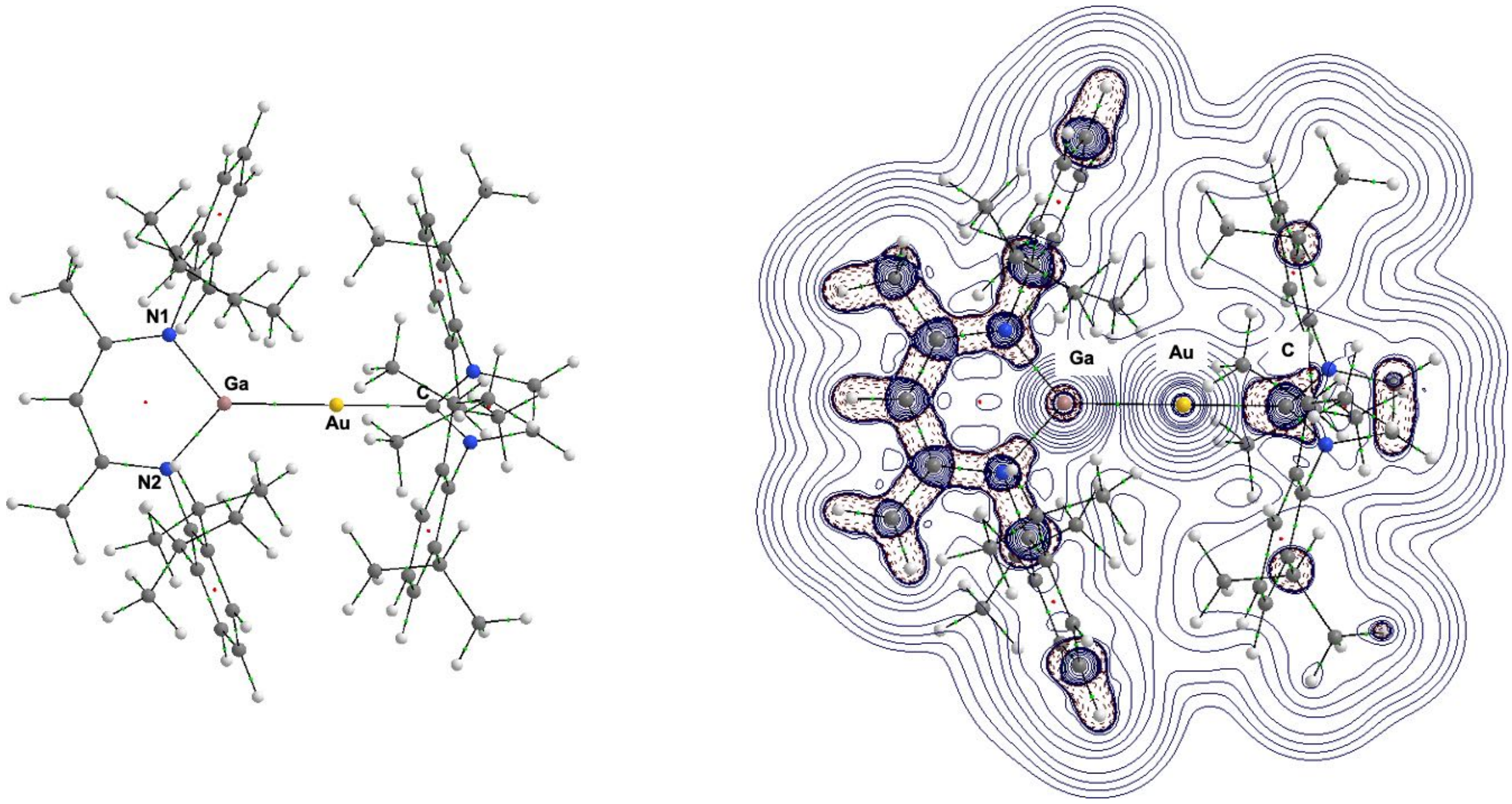

Figure S100. Molecular graph (left) and negative Laplacian $-\nabla^2\rho(r)$ of the electron density in the plane containing N1, Ga and Au (right) for **3**.

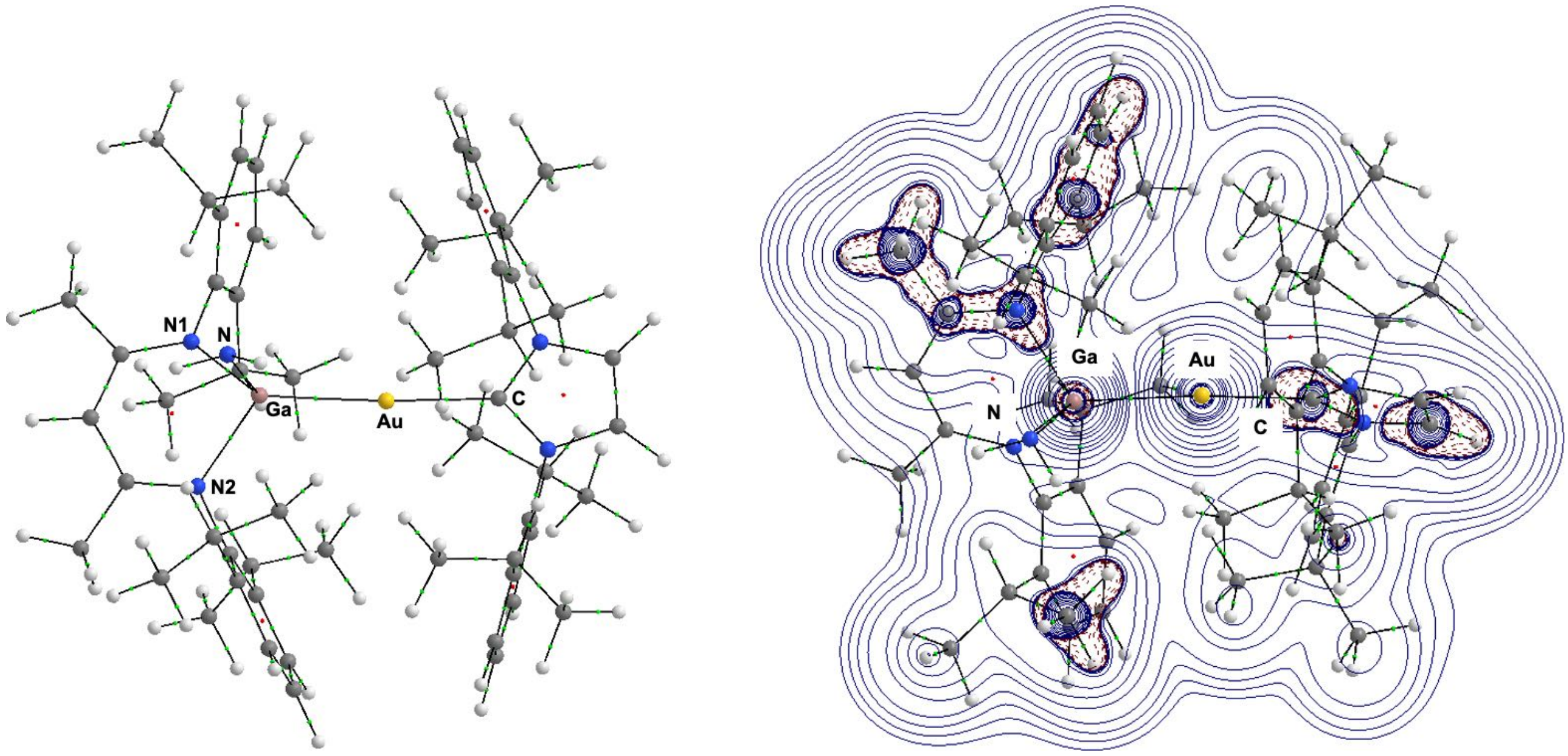

Figure S101. Molecular graph (left) and negative Laplacian $-\nabla^2\rho(r)$ of the electron density in the plane containing N1, Ga and Au (right) for **4**.

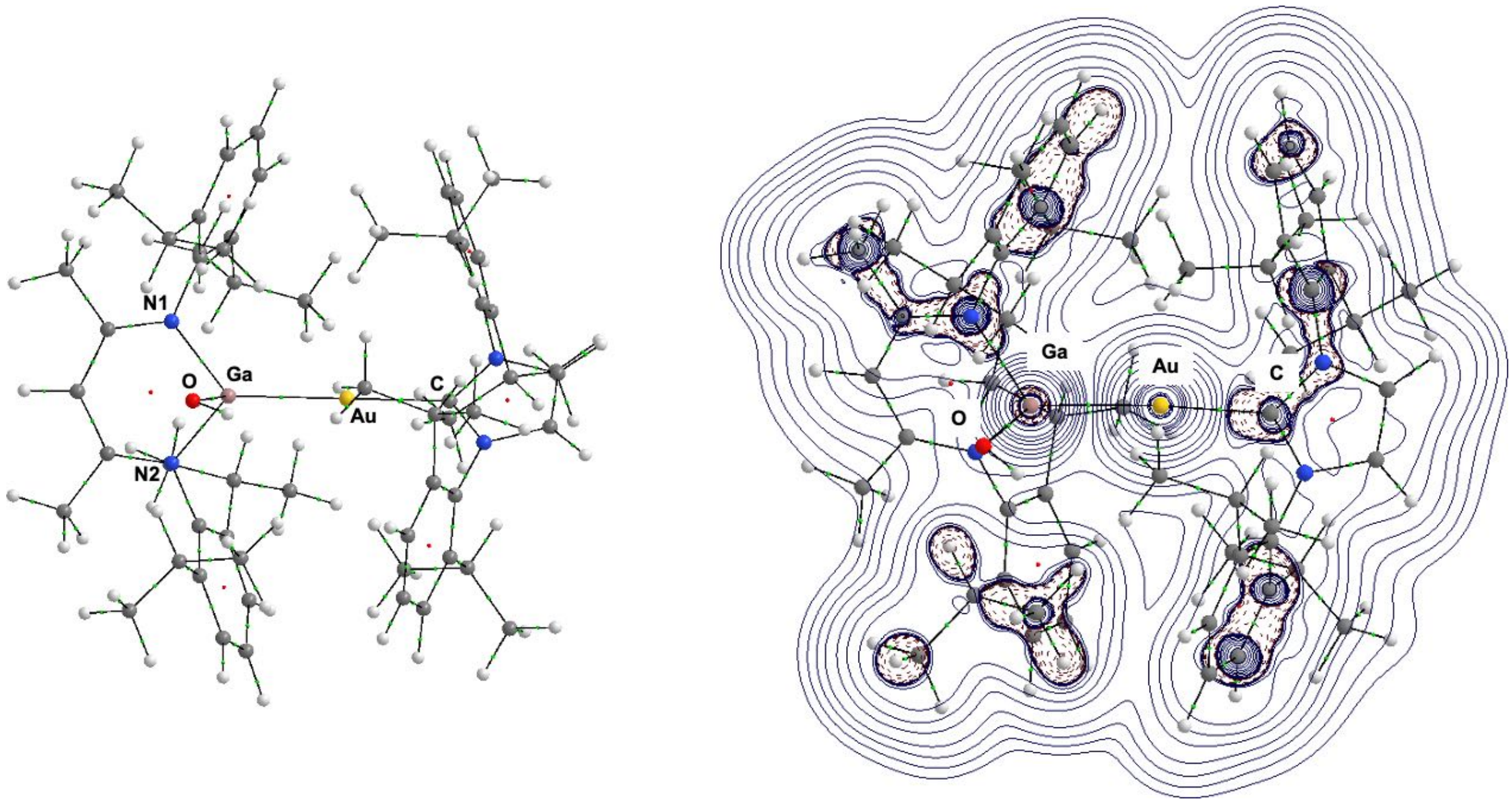

Figure S102. Molecular graph (left) and negative Laplacian $-\nabla^2\rho(r)$ of the electron density in the plane containing N1, Ga and Au (right) for **5**.

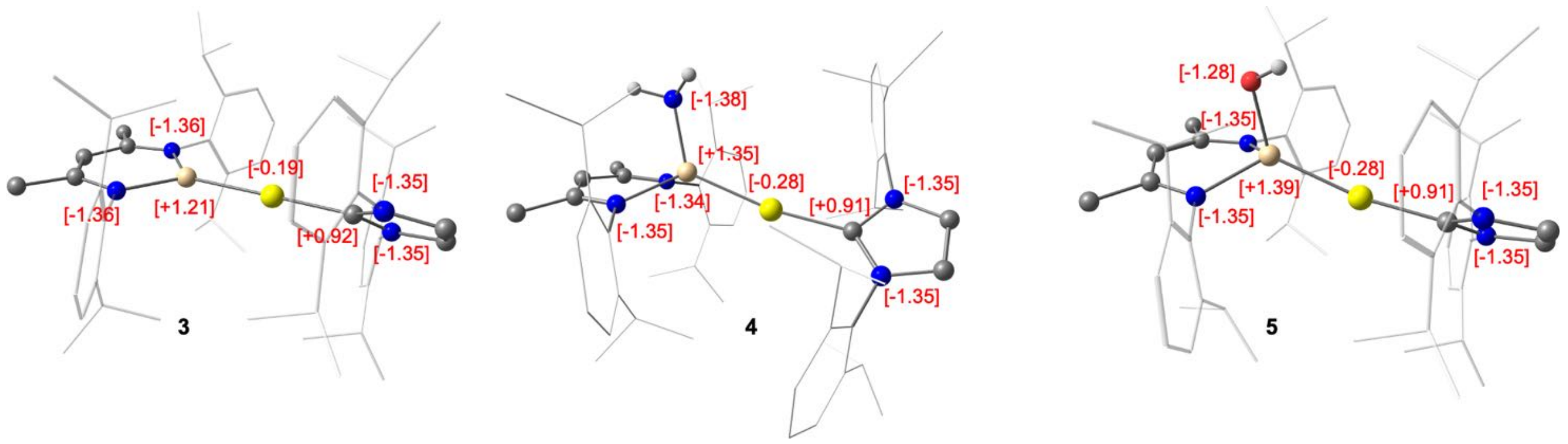

Figure S103. Calculated QTAIM charges (PBE0-D3(BJ)/def2-QZVP/def2-SVP) on key atoms in complexes **3**, **4** and **5**.

S6.1.4 Energy decomposition analysis (EDA)

Detailed insight into the composition of the interaction energy between the gallyl anion and the various cationic partners was obtained from energy decomposition analysis (EDA), the results of which are summarized in Table S29 and Table S30. The gas phase optimized geometries of **2·K(18-c-6)(THF)** and **$2\cdot Cs(18\text{-}c\text{-}6)_2$** displayed the highest disagreement with the experimental geometry, specifically for the Ga···Cs and K···O(THF) distances. Therefore, the solid-state optimized geometries for these two structures were also analyzed using EDA. This analysis revealed that for all complexes, ~60% of the interaction energy is due to electrostatic contribution. Although the orbital and dispersion terms contribute 12-14% and 9-15%, respectively, the repulsive Pauli term (12-17%) essentially cancels either one of these contributions. The contacted complexes, except for the solid state optimized **2·K(18-c-6)(THF)**, are more stabilized than the separated complexes. As a general trend it is observed that the smaller the alkali metal, the more stabilizing the interaction energy. This can be explained by the smaller alkali metals having an increased charge density.

In the solid-state optimized **2·K(18-c-6)(THF)**, the K···O(THF) distance being in better agreement with experiment in comparison to the gas phase geometry leads to a weaker interaction energy between the anion and cation, presumably as the donation from the oxygen atom of THF to the 4s orbital of potassium is now more evenly balanced with the donation from the gallyl anion. In the case of the solid-state optimized **$2\cdot Cs(18\text{-}c\text{-}6)_2$**, the better agreement with experiment with regards to the Ga···Cs distance leads to a weaker interaction energy, as expected.

For the two dimeric complexes, **$(2\cdot K)_2$** and **$(2\cdot Cs)_2$**, the interaction energies between the two alkali metal atoms and the gallyl anions were computed. The electrostatic contribution to the interaction energy is even greater (71% and 69%, for K and Cs, respectively) than is observed for the non-dimeric complexes. The orbital term contributes 18-20%, and perhaps quite surprisingly, the dispersion term only contributes 2% and 6%, for K and Cs, respectively. To shed light on the M···Dipp interactions, these fragments of the structures were isolated for an EDA analysis. It was found that the main stabilizing contributions came from the electrostatic term (39% / 38% for K / Cs) and the orbital term (42% / 39% for K / Cs).

Whilst the fragmentation into anionic and cationic portions for the above complexes ion pairs is chemically most plausible due to lack of direct bonding interactions, two fragmentation schemes were considered for the EDA of complexes **3**–**5** (Table S31). In the donor-acceptor model a heterolytic fragmentation was employed corresponding to units of $\mathbf{2}^{-}$ and $[Au(IPr)]^{+}$. Doublet (S = ½) configurations for $\mathbf{2}^{\bullet}$ and $[Au(IPr)]^{\bullet}$ fragments were used to represent an electron-sharing model. In line with literature the smaller magnitude of the orbital term $\mathbf{\Delta}E_{orb}$ dictates the most realistic bonding model.[48] For all cases considered, the Ga–Au interaction is best described as having donor-acceptor character.

Table S29. Energy decomposition analysis results for all non-dimeric complexes (PBE0D4/TZP, using an all-electron basis set). The structures are sorted from most to least stabilizing interaction energies (top to bottom). The values in rounded brackets are the percentage contribution of each component to the interaction energy. All energy values stated have units of kcal mol^{-1}. [b]Solid state optimized geometry was used for analysis.

Structure	ΔE_{int}	ΔE_{elec}	ΔE_{pauli}	ΔE_{orb}	ΔE_{disp}
**2·Li(crypt-222)**	-71.11	-59.80 (59%)	15.51 (15%)	-14.00 (14%)	-12.82 (13%)
**2·K(crypt-222)**	-68.11	-54.88 (62%)	10.84 (12%)	-10.95 (12%)	-12.13 (14%)
**2·Cs(crypt-222)**	-68.16	-54.89 (61%)	11.04 (12%)	-10.85 (12%)	-13.46 (15%)
**2·Na(18-c-6)**	-87.04	-76.85 (61%)	19.89 (16%)	-16.17 (13%)	-13.90 (11%)
**2·K(18-c-6)**	-80.08	-71.73 (62%)	17.52 (15%)	-13.37 (12%)	-12.49 (11%)
**2·Cs(18-c-6)**	-78.64	-72.51 (61%)	20.51 (17%)	-16.34 (14%)	-10.27 (9%)
**2·K(18-c-6)(THF)**	-78.79	-68.48 (61%)	17.01 (15%)	-14.58 (13%)	-12.74 (11%)
**2·K(18-c-6)(THF)**[b]	-68.98	-61.63 (61%)	16.10 (16%)	-11.96 (12%)	-11.49 (11%)
**2·Cs(18-c-6)$_2$**	-59.59	-49.99 (61%)	11.36 (14%)	-9.97 (12%)	-11.00 (13%)
**2·Cs(18-c-6)$_2$**[b]	-50.33	-42.20 (64%)	7.84 (12%)	-7.60 (12%)	-8.37 (13%)

Table S30. Energy decomposition analysis results for the dimeric complexes (PBE0D4/TZP, using an all-electron basis set). The values in rounded brackets are the percentage contribution of each component to the interaction energy. All energy values stated have units of kcal mol^{-1}.

Structure	ΔE_{int}	ΔE_{elec}	ΔE_{pauli}	ΔE_{orb}	ΔE_{disp}
**(2·K)$_2$**	-349.99	-298.49 (71%)	34.38 (8%)	-83.93 (20%)	-1.95 (0.5%)
K···Dipp(H)	-23.27	-12.36 (39%)	4.10 (13%)	-13.33 (42%)	-1.67 (5%)
Dipp(H)···K···Dipp(H)	-41.66	-22.81 (39%)	8.05 (14%)	-23.49 (41%)	-3.41 (6%)
**(2·Cs)$_2$**	-327.65	-290.49 (69%)	46.07 (11%)	-76.73 (18%)	-6.49 (2%)
Cs···Dipp(H)	-19.38	-11.32 (38%)	5.39 (18%)	-11.75 (39%)	-1.71 (6%)
Dipp(H) ···Cs···Dipp(H)	-35.29	-22.19 (38%)	11.61 (20%)	-21.39 (37%)	-3.32 (6%)

Table S31. Energy decomposition analysis results for the complexes **3**, **4** and **5** (PBE0-D3(BJ)/def2-TZVP, using an all-electron basis set). The values in rounded brackets are the percentage contribution to the *total bonding energy*. All energy values stated have units of kcal mol^{-1}. [a]Heterolytic fragmentation scheme (**2**$^{-}$ + $[Au(IPr)]^{+}$). [a]Homolytic fragmentation scheme (**2**$^{\bullet}$ + $[Au(IPr)]^{\bullet}$).

Structure	ΔE_{int}	ΔE_{elec}	ΔE_{pauli}	ΔE_{orb}	ΔE_{disp}
**3**[a]	-167.67	-180.46 (55%)	157.81	-123.01 (38%)	-22.02 (7%)
**3**[b]	-106.64	-204.47 (44%)	451.01	-242.18 (52%)	-22.02 (5%)
**4**[a]	-192.60	-218.71 (56%)	198.98	-149.81 (38%)	-23.06 (6%)
**4**[b]	-97.35	-204.39 (43%)	381.04	-251.01 (43%)	-23.06 (5%)
**5**[a]	-189.61	-215.23 (56%)	195.57	-146.94 (38%)	-23.01 (6%)
**5**[b]	-97.24	-204.88 (43%)	378.33	-247.72 (52%)	-23.01 (6%)

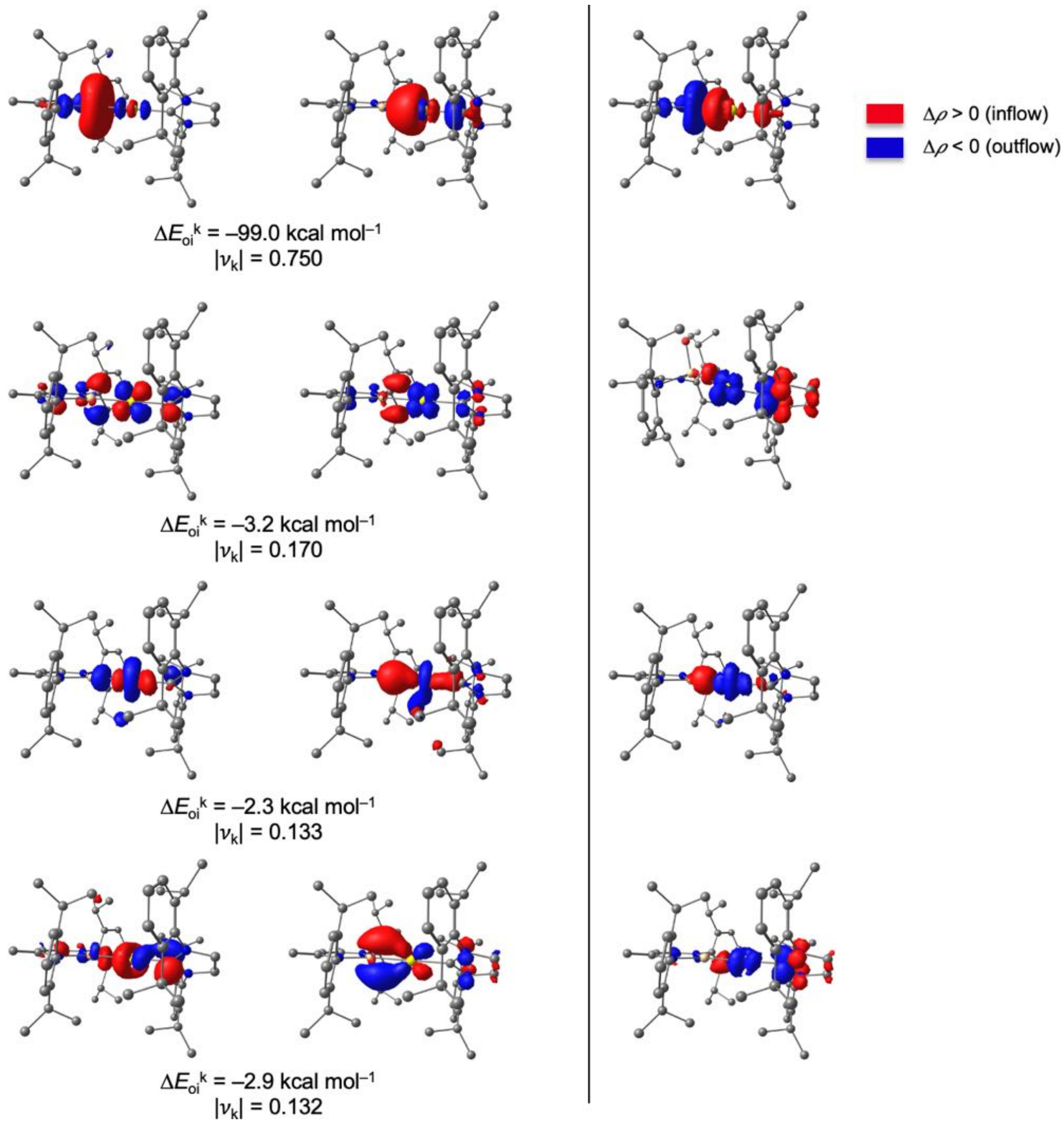

Figure S104. First four ETS-NOCV donor/acceptor orbitals pairs and associated deformation densities for **3** (PBE0-D3(BJ)/def2-TZVP, isovalue = 0.025 au). Blue and red deformation density surfaces indicate regions of charge depletion and accumulation, respectively.

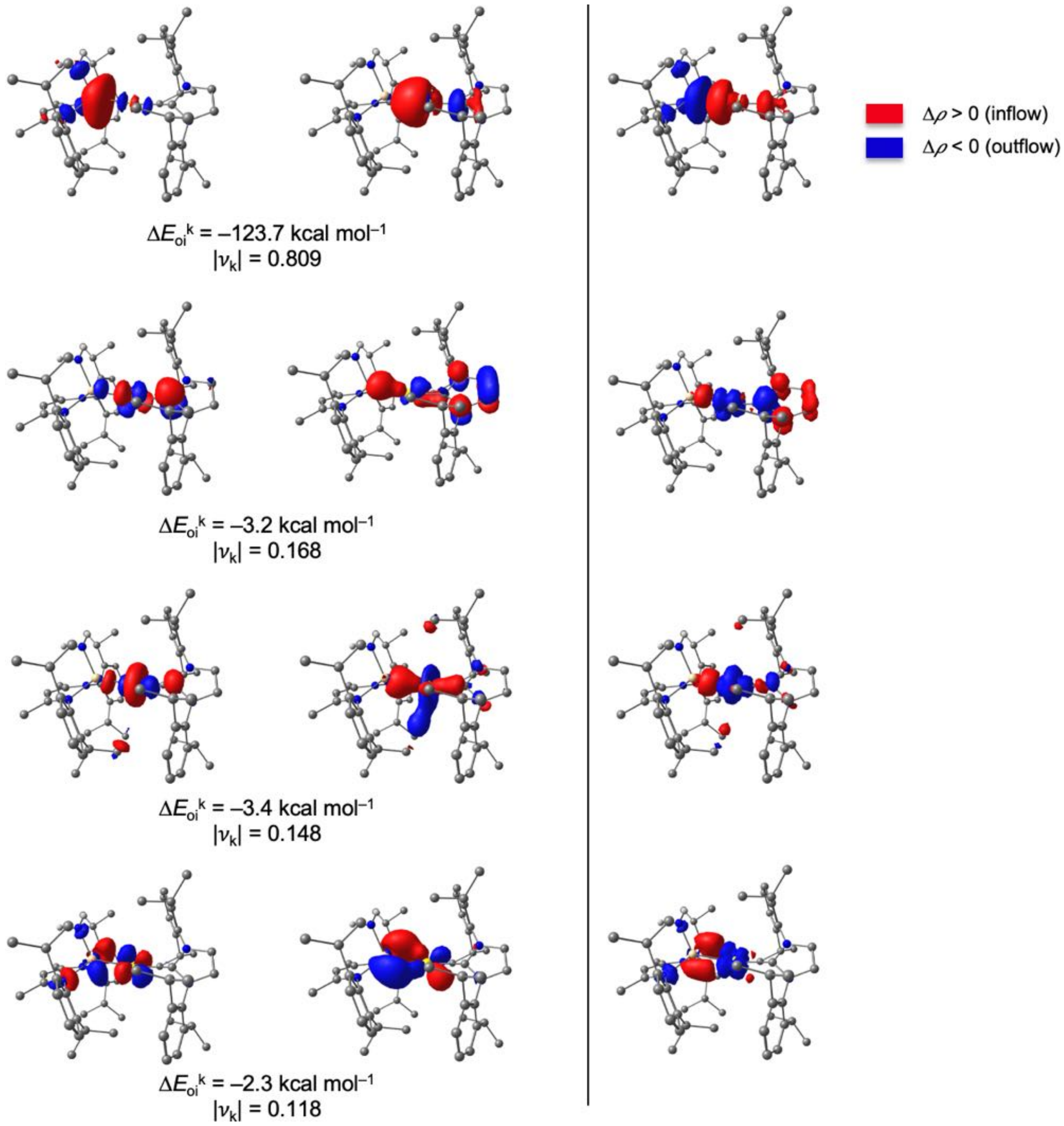

Figure S105. First four ETS-NOCV donor/acceptor orbitals pairs and associated deformation densities for **4** (PBE0-D3(BJ)/def2-TZVP, isovalue = 0.025 au). Blue and red deformation density surfaces indicate regions of charge depletion and accumulation, respectively.

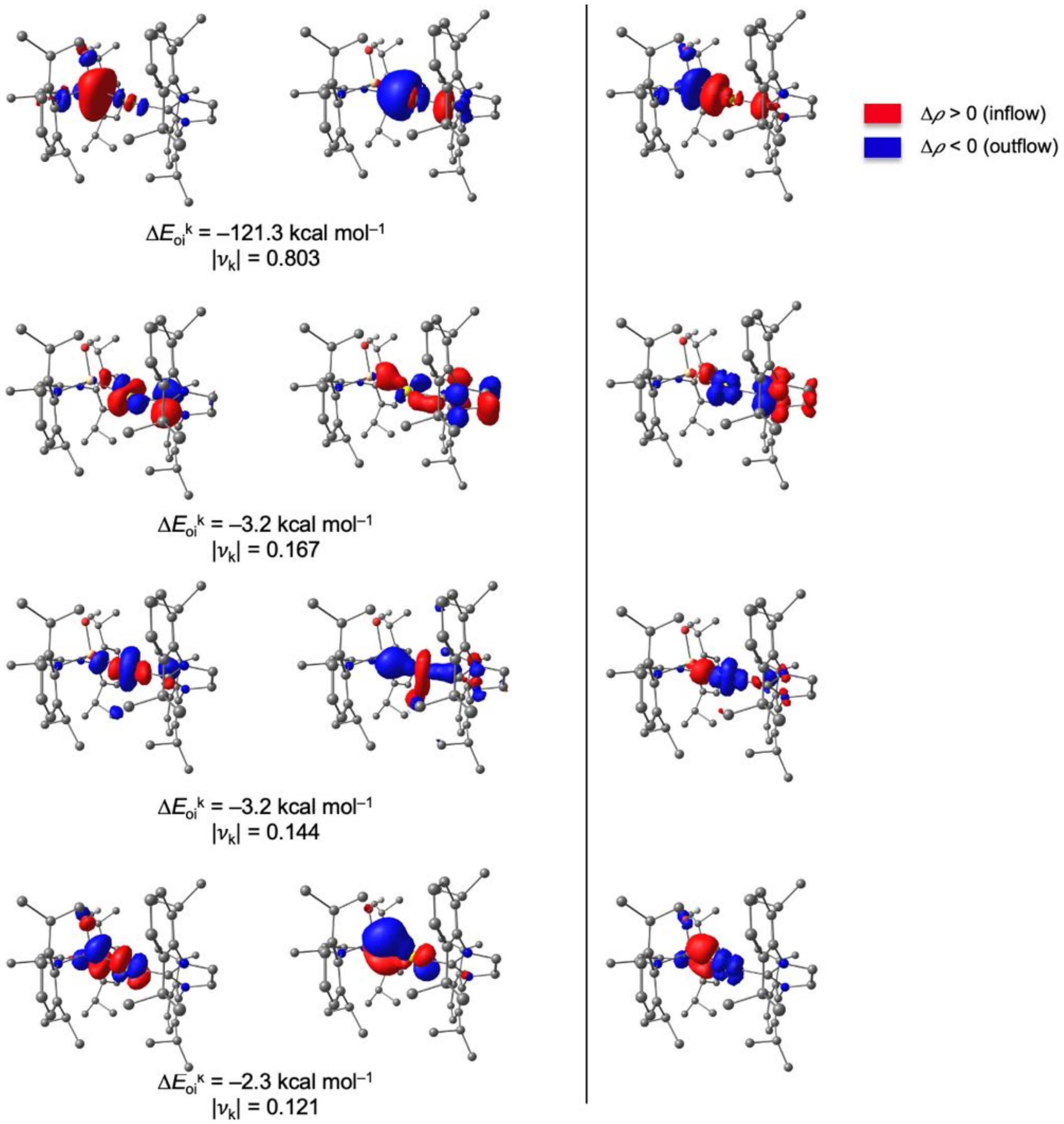

Figure S106. First four ETS-NOCV donor/acceptor orbitals pairs and associated deformation densities for **5** (PBE0-D3(BJ)/def2-TZVP, isovalue = 0.025 au). Blue and red deformation density surfaces indicate regions of charge depletion and accumulation, respectively.

S6.1.5 Molecular Electrostatic Potential Maps

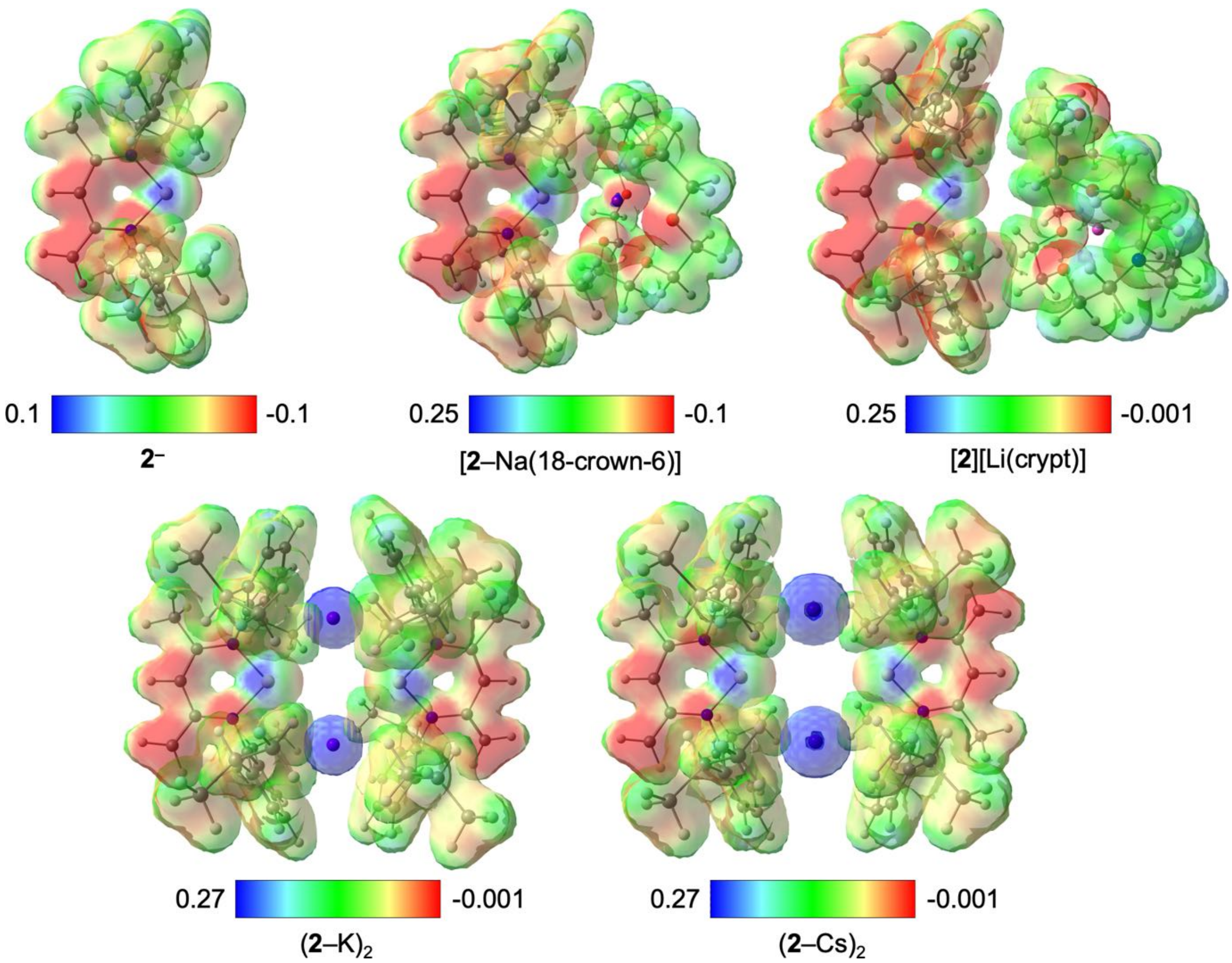

Figure S107. MEP maps for representative structures (isovalue = 0.025 a.u.).

S6.1.6 Non-Covalent Interactions

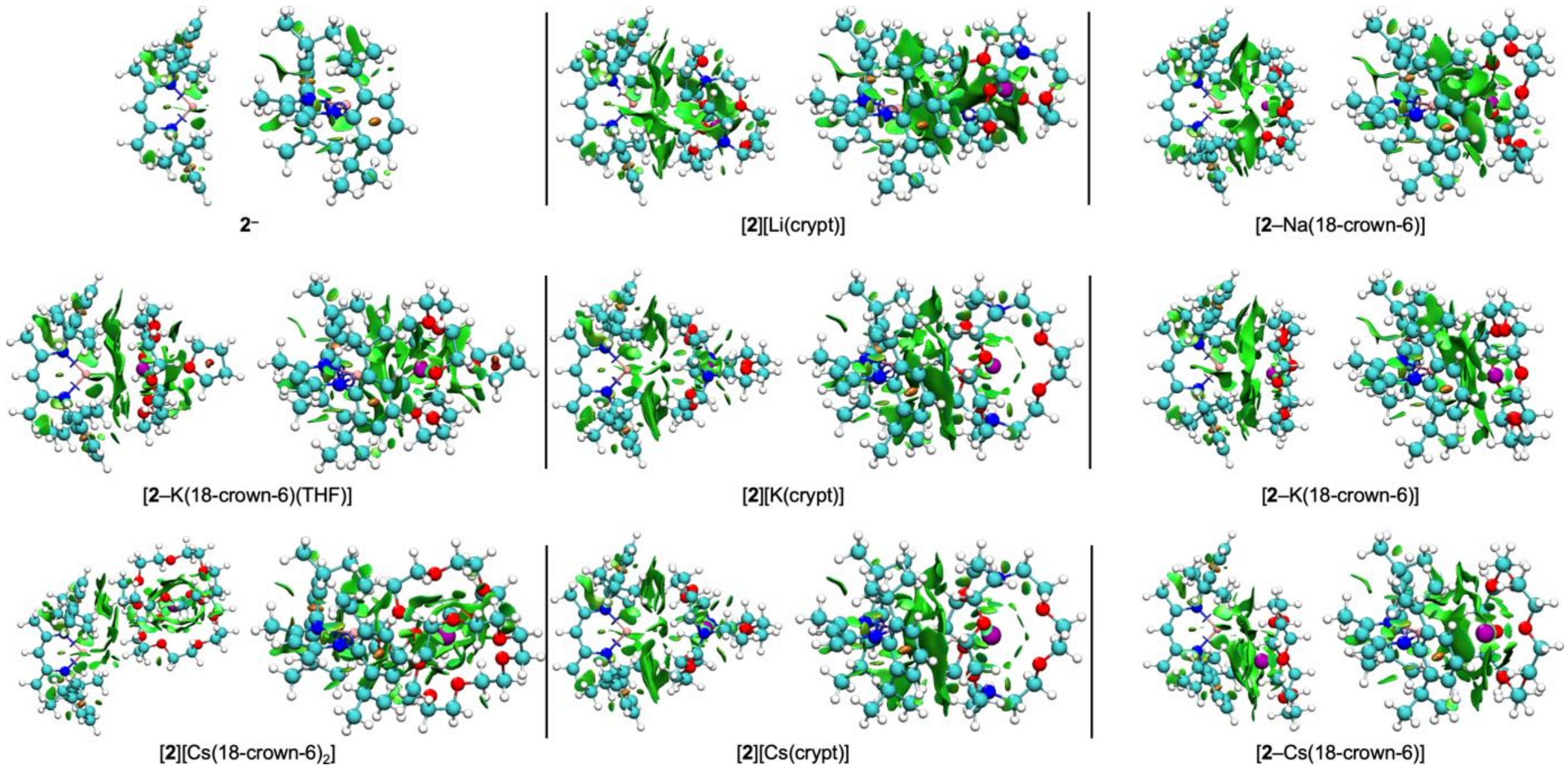

Figure S108. Non-covalent interactions (NCI) plots for all non-dimeric structures. Top and side views for each structure are shown (left and right, respectively)

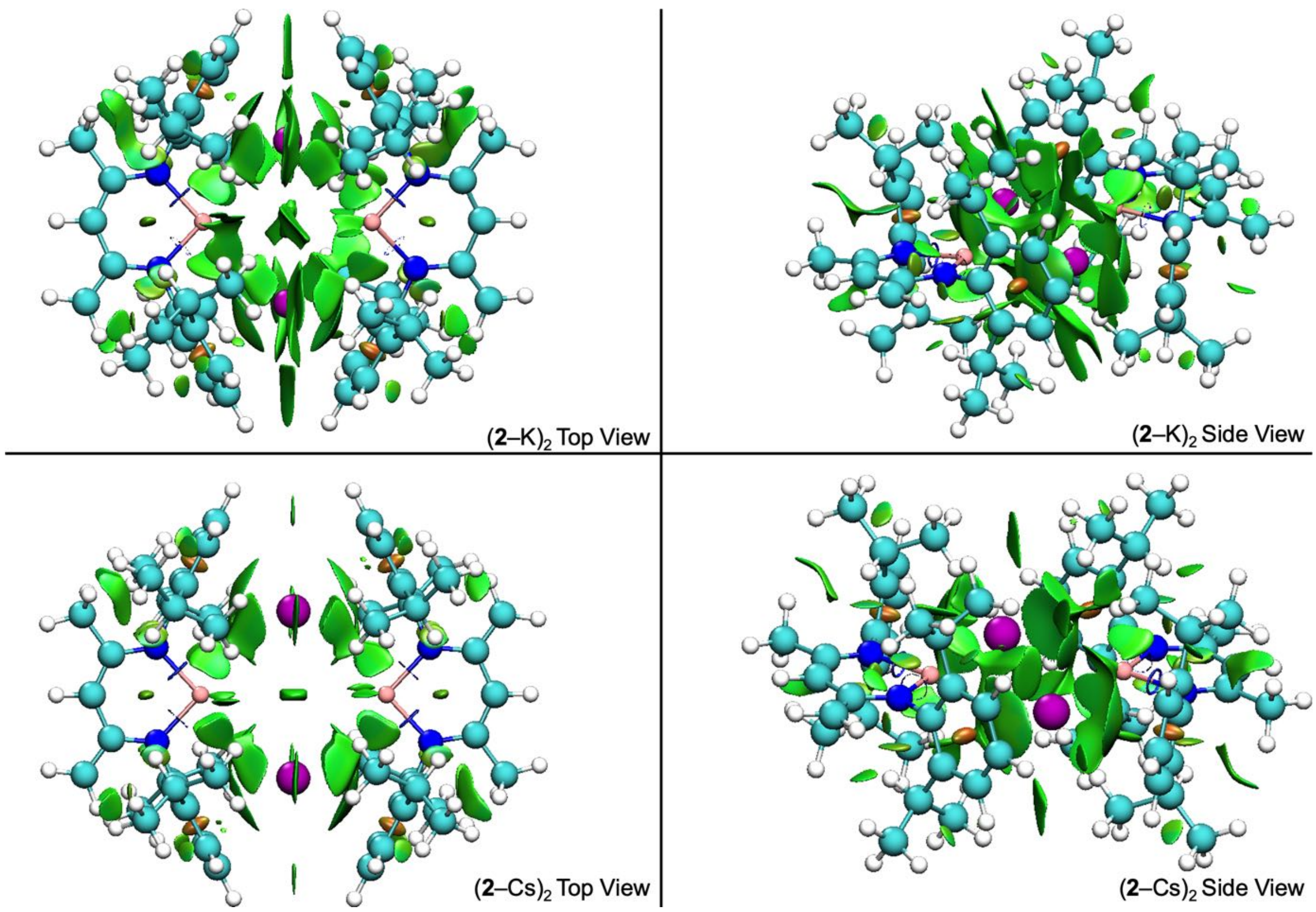

Figure S109. Non-covalent interaction (NCI) plots for the contacted dimeric structures.

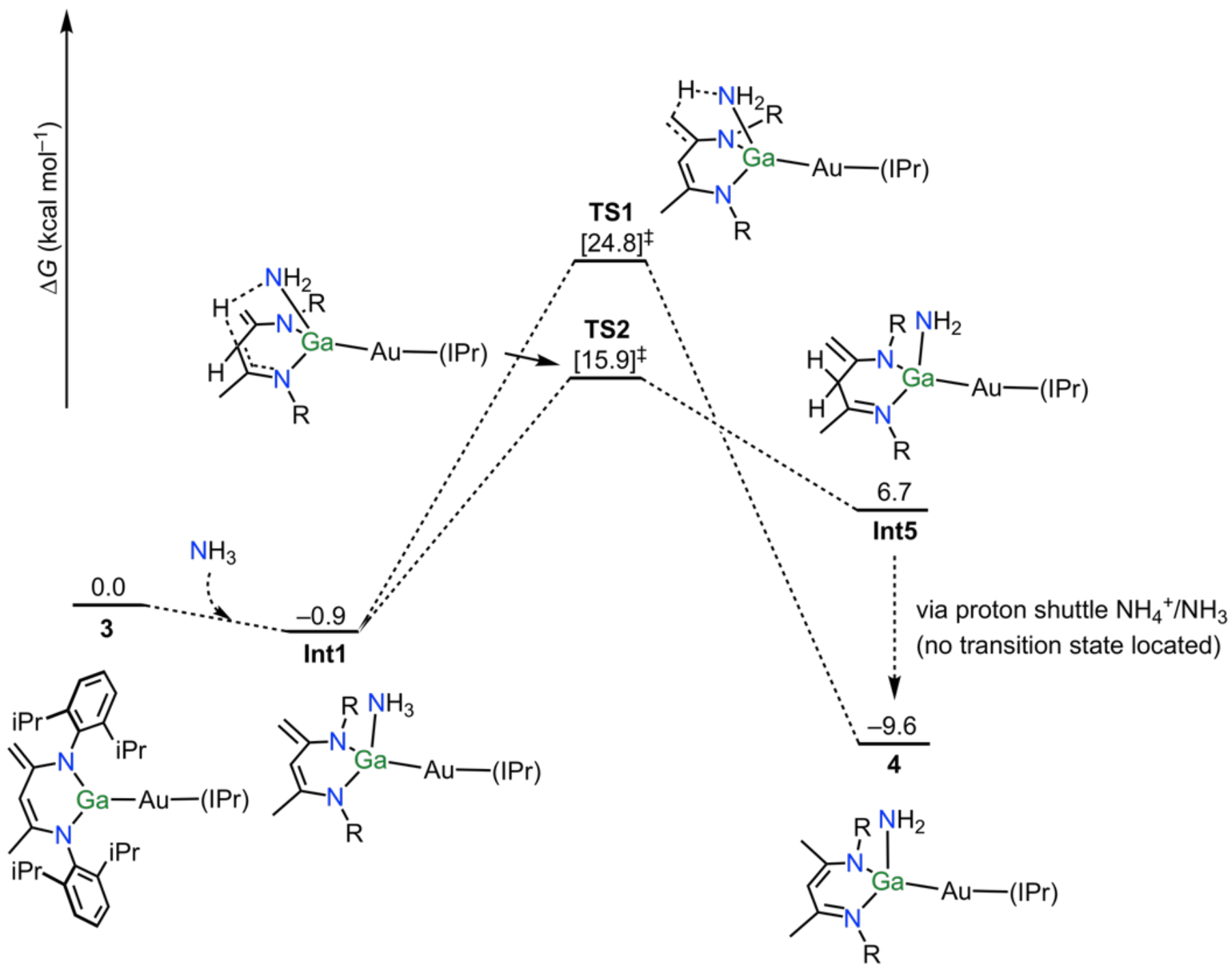

Figure S110. Calculated Gibbs Free energy reaction profile (298 K, in kcal/mol) for direct NH_3 activation at **3** at the PBE0-D3(BJ)/def2-TZVP//PBE-D3(BJ)/def2-TZVP/def2-SV(P) level of theory, corrected for benzene solvent.

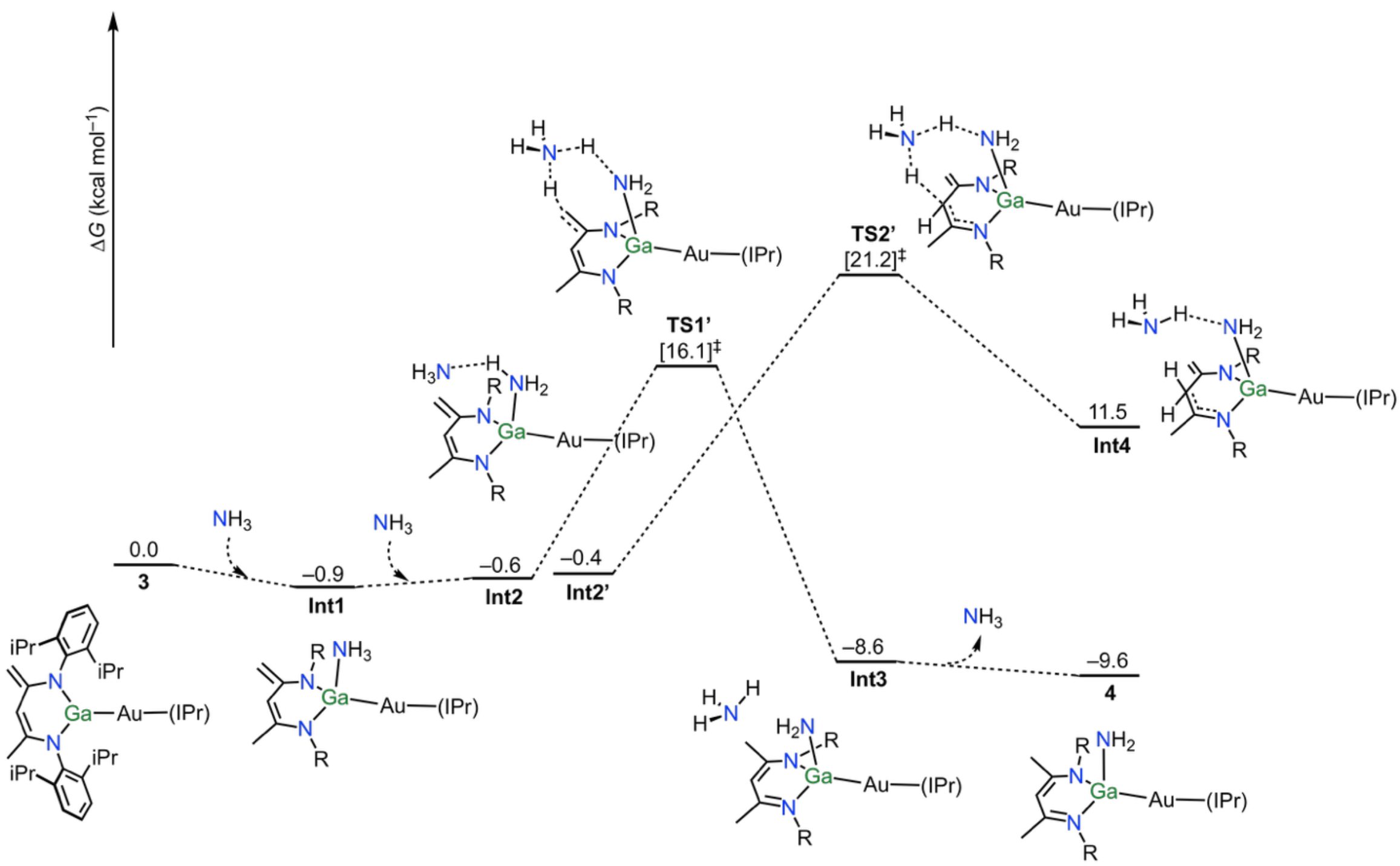

Figure S111. Calculated Gibbs Free energy reaction profile (298 K, in kcal/mol) for NH_3 activation at **3** via proton shuttle at the PBE0-D3(BJ)/def2-TZVP//PBE-D3(BJ)/def2-TZVP/def2-SV(P) level of theory, corrected for benzene solvent.

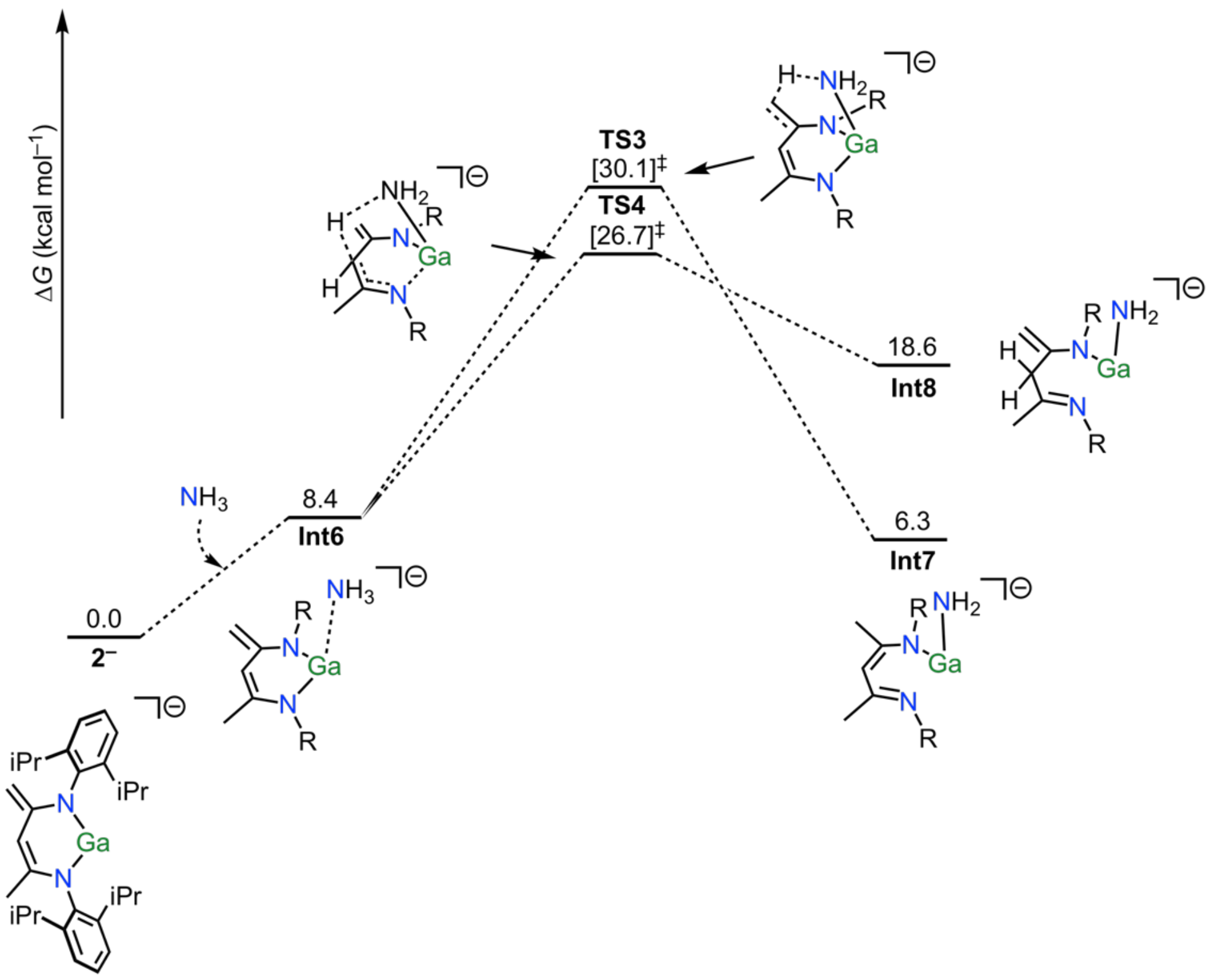

Figure S112. Calculated Gibbs Free energy reaction profile (298 K, in kcal/mol) for direct NH_3 activation at **2**⁻ at the PBE0-D3(BJ)/def2-TZVP//PBE-D3(BJ)/def2-TZVP/def2-SV(P) level of theory, corrected for benzene solvent.

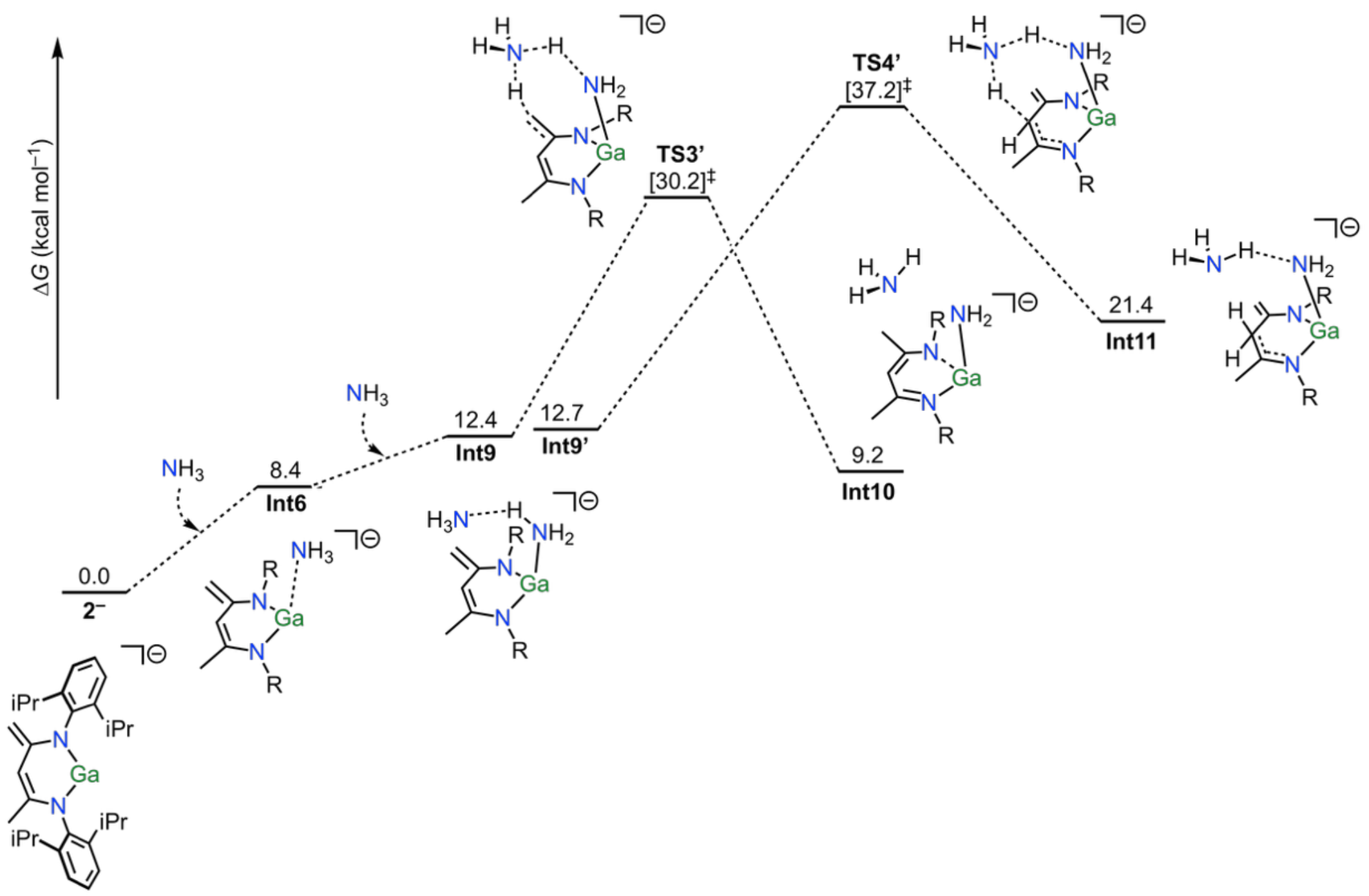

Figure S113. Calculated Gibbs Free energy reaction profile (298 K, in kcal/mol) for NH_3 activation at **2**⁻ via proton shuttle at the PBE0-D3(BJ)/def2-TZVP//PBE-D3(BJ)/def2-TZVP/def2-SV(P) level of theory, corrected for benzene solvent.

S7 References

[1] X. He, Y. Yao, X. Luo, J. Zhang, Y. Liu, L. Zhang, Q. Wu, *Organometallics* **2003**, *22*, 4952-4957.

[2] N. J. Hardman, B. E. Eichler, P. P. Power, *Chem. Commun.* **2000**, 1991-1992.

[3] M. F. Lappert, M. J. Slade, A. Singh, J. L. Atwood, R. D. Rogers, R. Shakir, *J. Am. Chem. Soc.* **1983**, *105*, 302-304.

[4] A. I. Ojeda-Amador, A. J. Martínez-Martínez, A. R. Kennedy, C. T. O'Hara, *Inorg. Chem.* **2016**, *55*, 5719-5728.

[5] P. de Frémont, N. M. Scott, E. D. Stevens, S. P. Nolan, *Organometallics* **2005**, *24*, 2411-2418.

[6] *Organometallics* **2016**, *35*, 3255-3256.

[7] A. C. Aiken, P. F. DeCarlo, J. L. Jimenez, *Anal. Chem.* **2007**, *79*, 8350-8358.

[8] R. E. H. Kuveke, L. Barwise, Y. van Ingen, K. Vashisth, N. Roberts, S. S. Chitnis, J. L. Dutton, C. D. Martin, R. L. Melen, *ACS Cent. Sci.* **2022**, *8*, 855-863.

[9] N. v. Well, C. Klein, F. Ritter, W. Assmus, C. Krellner, M. Bolte, *Acta Crystallogr., Sect. C* **2014**, *70*, 455-459.

[10] A. C. Coxon, J. F. Stoddart, *J. Chem. Soc., Perkin Trans. 1* **1977**, 767-785.

[11] S. Bachmann, R. Neufeld, M. Dzemski, D. Stalke, *Chem. Eur. J.* **2016**, *22*, 8462-8465.

[12] R. Neufeld, D. Stalke, *Chem. Sci.* **2015**, *6*, 3354-3364.

[13] A.-K. Kreyenschmidt, S. Bachmann, T. Niklas, D. Stalke, *ChemistrySelect* **2017**, *2*, 6957-6960.

[14] W. M. Haynes, *CRC Handbook of Chemistry and Physics*, CRC Press, **2014**.

[15] R. Gajda, A. Katrusiak, *Cryst. Growth Des.* **2008**, *8*, 211-214.

[16] J. Donohue, S. H. Goodman, *Acta Crystallogr.* **1967**, *22*, 352-354.

[17] A. Mercier, W. C. Yeo, J. Chou, P. D. Chaudhuri, G. Bernardinelli, E. P. Kündig, *Chem. Comm.* **2009**, 5227-5229.

[18] W. H. Ojala, H. L. Gustafson, C. R. Ojala, *Acta Crystallogr. C* **1994**, *50*, 1602-1604.

[19] S. Alvarez, *Dalton Trans.* **2013**, *42*, 8617-8636.

[20] A. S. Virk, A. M. Torres, S. A. Willis, W. S. Price, *J. Mol. Liq.* **2016**, *214*, 157-161.

[21] J. Cosier, A. M. Glazer, *J. Appl. Crystallogr.* **1986**, *19*, 105-107.

[22] Bruker (2018). APEX3. Bruker AXS Inc., Madison, Wisconsin, USA.

[23] G. M. Sheldrick, *Acta Crystallogr A Found Adv* **2015**, *71*, 3-8.

[24] G. M. Sheldrick, *Acta Crystallogr., Sect. A* **2008**, *64*, 112-122.

[25] O. V. Dolomanov, L. J. Bourhis, R. J. Gildea, J. A. K. Howard, H. Puschmann, *J. Appl. Crystallogr.* **2009**, *42*, 339-341.

[26] M. J. Frisch, G. W. Trucks, H. B. Schlegel, G. E. Scuseria, M. A. Robb, J. R. Cheeseman, G. Scalmani, V. Barone, G. A. Petersson, H. Nakatsuji, X. Li, M. Caricato, A. V. Marenich, J. Bloino, B. G. Janesko, R. Gomperts, B. Mennucci, H. P. Hratchian, J. V. Ortiz, A. F. Izmaylov, J. L. Sonnenberg, Williams, F. Ding, F. Lipparini, F. Egidi, J. Goings, B. Peng, A. Petrone, T. Henderson, D. Ranasinghe, V. G. Zakrzewski, J. Gao, N. Rega, G. Zheng, W. Liang, M. Hada, M. Ehara, K. Toyota, R. Fukuda, J. Hasegawa, M. Ishida, T. Nakajima, Y. Honda, O. Kitao, H. Nakai, T. Vreven, K. Throssell, J. A. Montgomery Jr., J. E. Peralta, F. Ogliaro, M. J. Bearpark, J. J. Heyd, E. N. Brothers, K. N. Kudin, V. N. Staroverov, T. A. Keith, R. Kobayashi,

J. Normand, K. Raghavachari, A. P. Rendell, J. C. Burant, S. S. Iyengar, J. Tomasi, M. Cossi, J. M. Millam, M. Klene, C. Adamo, R. Cammi, J. W. Ochterski, R. L. Martin, K. Morokuma, O. Farkas, J. B. Foresman, D. J. Fox, Wallingford, CT, **2016**.

[27] G. te Velde, F. M. Bickelhaupt, E. J. Baerends, C. Fonseca Guerra, S. J. A. van Gisbergen, J. G. Snijders, T. Ziegler, *J. Comput. Chem.* **2001**, *22*, 931-967.

[28] J. Hutter, M. Iannuzzi, F. Schiffmann, J. VandeVondele, *J. WIREs Comput. Mol. Sci.* **2014**, *4*, 15-25.

[29] J. P. Perdew, K. Burke, M. Ernzerhof, *Phys. Rev. Lett.* **1996**, *77*, 3865-3868.

[30] F. Weigend, R. Ahlrichs, *Phys. Chem. Chem. Phys.* **2005**, *7*, 3297-3305.

[31] F. Weigend, *Phys. Chem. Chem. Phys.* **2006**, *8*, 1057-1065.

[32] S. Grimme, S. Ehrlich, L. Goerigk, *J. Comput. Chem.* **2011**, *32*, 1456-1465.

[33] E. Van Lenthe, E. J. Baerends, *J. Comput. Chem.* **2003**, *24*, 1142-1156.

[34] E. Caldeweyher, S. Ehlert, A. Hansen, H. Neugebauer, S. Spicher, C. Bannwarth, S. Grimme, *J. Chem. Phys.* **2019**, *150*.

[35] E. D. Glendening, C. R. Landis, F. Weinhold, *J. Comput. Chem.* **2013**, *34*, 1429-1437.

[36] R. F. W. Bader, *Acc. Chem. Res.* **1985**, *18*, 9-15.

[37] Keith, T. A. AIMAll (Version 19.10.12), 2019.

[38] J. VandeVondele, M. Krack, F. Mohamed, M. Parrinello, T. Chassaing, J. Hutter, *J. Comput. Phys. Commun.* **2005**, *167*, 103-128.

[39] J. VandeVondele, J. Hutter, *J. Chem. Phys.* **2007**, *127*.

[40] C. Hartwigsen, S. Goedecker, J. Hutter, *J. Phys. Rev. B* **1998**, *58*, 3641-3662.

[41] S. Goedecker, M. Teter, J. Hutter, *J. Phys. Rev. B* **1996**, *54*, 1703-1710.

[42] M. Krack, *Theor. Chem. Acc.* **2005**, *114*, 145-152.

[43] E. R. Johnson, S. Keinan, P. Mori-Sánchez, J. Contreras-García, A. J. Cohen, W. Yang, *J. Am. Chem. Soc.* **2010**, *132*, 6498-6506.

[44] J. Contreras-García, E. R. Johnson, S. Keinan, R. Chaudret, J.-P. Piquemal, D. N. Beratan, W. Yang, *J. Chem. Theory Comput.* **2011**, *7*, 625-632.

[45] W. Humphrey, A. Dalke, K. Schulten, *J. Mol. Graph.* **1996**, *14*, 33-38.

[46] S. Grams, J. Mai, J. Langer, S. Harder, *Dalton Trans.* **2022**, *51*, 12476-12483.
